# SMAD3 promotes expression and activity of the androgen receptor in prostate cancer

**DOI:** 10.1093/nar/gkad043

**Published:** 2023-02-02

**Authors:** Hee-Young Jeon, Majid Pornour, Hyunju Ryu, Sudeep Khadka, Rui Xu, Jihyun Jang, Deqiang Li, Hegang Chen, Arif Hussain, Ladan Fazli, Martin Gleave, Xuesen Dong, Furong Huang, Qianben Wang, Christopher Barbieri, Jianfei Qi

**Affiliations:** Department of Biochemistry and Molecular Biology, University of Maryland, Baltimore, MD, USA; Marlene and Stewart Greenebaum Comprehensive Cancer Center, Baltimore, MD, USA; Department of Biochemistry and Molecular Biology, University of Maryland, Baltimore, MD, USA; Marlene and Stewart Greenebaum Comprehensive Cancer Center, Baltimore, MD, USA; Department of Biochemistry and Molecular Biology, University of Maryland, Baltimore, MD, USA; Marlene and Stewart Greenebaum Comprehensive Cancer Center, Baltimore, MD, USA; Department of Biochemistry and Molecular Biology, University of Maryland, Baltimore, MD, USA; Marlene and Stewart Greenebaum Comprehensive Cancer Center, Baltimore, MD, USA; Department of Biochemistry and Molecular Biology, University of Maryland, Baltimore, MD, USA; Institute of Marine and Environmental Technology, University of Maryland, Baltimore, MD, USA; Department of Cardiac Surgery, University of Maryland, Baltimore, MD, USA; Department of Cardiac Surgery, University of Maryland, Baltimore, MD, USA; Department of Epidemiology and Public Health, University of Maryland, Baltimore, MD, USA; Department of Biochemistry and Molecular Biology, University of Maryland, Baltimore, MD, USA; Marlene and Stewart Greenebaum Comprehensive Cancer Center, Baltimore, MD, USA; Baltimore VA Medical Center, Baltimore, MD, USA; Vancouver Prostate Centre, University of British Columbia, Vancouver, BC, Canada; Vancouver Prostate Centre, University of British Columbia, Vancouver, BC, Canada; Vancouver Prostate Centre, University of British Columbia, Vancouver, BC, Canada; Department of Pathology and Duke Cancer Institute, Duke University School of Medicine, Durham, NC, USA; Department of Pathology and Duke Cancer Institute, Duke University School of Medicine, Durham, NC, USA; Department of Urology, Weill Cornell Medical College, NY, NY, USA; Department of Biochemistry and Molecular Biology, University of Maryland, Baltimore, MD, USA; Marlene and Stewart Greenebaum Comprehensive Cancer Center, Baltimore, MD, USA

## Abstract

Overexpression of androgen receptor (AR) is the primary cause of castration-resistant prostate cancer, although mechanisms upregulating AR transcription in this context are not well understood. Our RNA-seq studies revealed that SMAD3 knockdown decreased levels of AR and AR target genes, whereas SMAD4 or SMAD2 knockdown had little or no effect. ChIP-seq analysis showed that SMAD3 knockdown decreased global binding of AR to chromatin. Mechanistically, we show that SMAD3 binds to intron 3 of the AR gene to promote AR expression. Targeting these binding sites by CRISPRi reduced transcript levels of AR and AR targets. In addition, ∼50% of AR and SMAD3 ChIP-seq peaks overlapped, and SMAD3 may also cooperate with or co-activate AR for AR target expression. Functionally, AR re-expression in SMAD3-knockdown cells partially rescued AR target expression and cell growth defects. The SMAD3 peak in AR intron 3 overlapped with H3K27ac ChIP-seq and ATAC-seq peaks in datasets of prostate cancer. AR and SMAD3 mRNAs were upregulated in datasets of metastatic prostate cancer and CRPC compared with primary prostate cancer. A SMAD3 PROTAC inhibitor reduced levels of AR, AR-V7 and AR targets in prostate cancer cells. This study suggests that SMAD3 could be targeted to inhibit AR in prostate cancer.

## INTRODUCTION

Prostate cancer (PCa) is the second most common cancer in men worldwide. The androgen receptor (AR) is the primary target in treating advanced PCa. Upon androgen binding, AR translocates into nuclei, binds specific genomic locations, and regulates gene expression to promote PCa cell proliferation and survival. Thus, androgen deprivation therapy (ADT), which suppresses AR activity, is the first-line treatment for metastatic PCa. However, despite initial clinical remission after ADT, disease invariably re-occurs in 2–3 years and progresses to lethal castration-resistant prostate cancer (CRPC). Second-generation AR pathway inhibitors such as enzalutamide and abiraterone were developed to treat CRPC by repressing AR signaling. These treatments extend patient survival, but CRPC eventually becomes drug-resistant, driven by reactivated AR signaling (1,2).

Re-activation of AR transcriptional activity in CRPC occurs via multiple mechanisms, such as AR overexpression/mutation/splicing, overexpression of AR co-factors, and/or intratumoral androgen biosynthesis (3). Modest increases in AR expression are reportedly both necessary and sufficient to convert PCa xenograft growth from a castration-sensitive to a castration-resistant stage (4). AR gene amplification is reported in 30–50% of CRPC cases (5,6). Amplification of an AR upstream enhancer (650 kb upstream of the AR gene), which was seen in 20 of 23 CRPC cases, can lead to increased transcription of AR mRNA (7). Transcriptional upregulation of AR could also contribute to the increased AR mRNA expression in CRPC. Although AR is known to be regulated at the transcriptional level, such mechanisms in CRPC are not well understood (8).

SMAD3, a key transcription factor mediating TGF-β signaling, consists of an N-terminal MH1 domain that interacts with the Smad Binding Element (SBE), a linker region, and a C-terminal MH2 domain that interacts with other proteins (9). In canonical TGF-β signaling, TGFBR1 activation phosphorylates SMAD2 and SMAD3, enabling each to heterodimerize with SMAD4. SMAD2-SMAD4 or SMAD3-SMAD4 complexes then translocate to the nucleus to control gene expression and regulate activities such as cell migration, invasion, growth, and apoptosis (10). SMAD4 functions as a tumor suppressor in PCa (11,12). In contrast, SMAD3 is overexpressed in advanced PCa (13) and reportedly promotes PCa progression (13–16). Mechanisms of SMAD3 in PCa progression are not well defined. Several prior studies have demonstrated that SMAD3 interacts physically with AR and functions as an AR co-regulator (17–19). However, the effect of SMAD3 on global expression of AR target genes remains unknown.

Here, we identify AR as a key downstream effector of SMAD3 in gene expression and PCa progression. We reveal a new role for SMAD3 in promoting expression of AR mRNA by binding to an enhancer in AR intron 3. Our results also implicate that SMAD3 cooperates with or co-activates AR in terms of AR target gene expression. Overall, our study supports the idea that SMAD3 may provide new therapeutic opportunity to inhibit AR for potential CRPC therapy.

## MATERIALS AND METHODS

### Antibodies and reagents

Antibodies were purchased from the following companies: AR (06–680), EMD Millipore (Burlington, MA); AR (22089-1-AP), Proteintech (Rosemont, IL); AR-V7 (31-1109-00), RevMAb Biosciences (South San Francisco, CA); SMAD3 (#9523), SMAD2 (#5339), phospho-SMAD3 (#9520) and phospho-SMAD2 (#3108), Cell Signaling Technology (Danvers, MA); SMAD3 (ab28379), Abcam (Cambridge, UK); Cas9 (61957), Active Motif (Carlsbad, CA); Rabbit IgG control (13–0042), EpiCypher (Durham, NC); Mouse IgG control (02–6300), Invitrogen (Waltham, MA); Myc (sc-40, sc-789), Santa Cruz Biotechnology (Dallas, TX); Flag (F7425, F3165) and actin (A5441), Sigma-Aldrich (St. Louis, MO). Trueblot secondary antibodies (18-8816-31, 18-4416-32) were purchased from Rockland Immunochemical (Pottstown, PA). Enzalutamide was purchased from Abmole Bioscience (Kowloon, Hongkong). R1881 was purchased from Sigma-Aldrich (St. Louis, MO). TGF-β was purchased from Cell Signaling Technology (Danvers, MA). SMAD3 phosphorylation inhibitor SIS3 (HY-13013) and SMAD3 PROTAC inhibitor (HY-147025) were purchased from MedChemExpress (Monmouth Junction, NJ). All reagents were used according to manufacturers’ recommendations.

### Cell lines

Rv1 cells (also called CWR22Rv1 cells) were provided by Dr James Jacobberger (Case Western Reserve University, Cleveland, Ohio). C4-2 and C4-2B cells were provided by Dr. Leland Chung (Cedars-Sinai Medical Center, Los Angeles, CA). Enzalutamide-resistant C4-2B cells were generated by serial growth and passaging of parental C4-2B cells in sequentially increasing concentrations of enzalutamide (2–20 μM) for over 1 year. LN95 cells were provided by Dr. Alan Meeker (Johns Hopkins University, Baltimore, MD). PC3 and VCaP cells were purchased from the American Type Culture Collection (ATCC). Rv1, C4-2, C4-2B and PC3 cells were maintained in RPMI 1640 media supplemented with 10% FBS and antibiotics. VCaP cells were maintained in DMEM media supplemented with 10% FBS and antibiotics. LN95 cells were maintained in phenol-red free RPMI 1640 media with 5% charcoal-stripped FBS and antibiotics. Cells were periodically checked for Mycoplasma by PCR analysis, and cells of <20 passages were used for experiments.

### RNA-sequencing analysis

Rv1 cells were transduced with sh-Control, sh-SMAD2, sh-SMAD3, or sh-SMAD4 lentivirus for 48 hours. Total RNAs were extracted using a total RNA miniprep kit (Sigma-Aldrich, St. Louis, MO). Three biological replicates of RNA samples were used for library preparation, sequencing (PE150, 20 million reads per library), and differential expression analysis by Novogene Corporation (Sacramento, CA). In brief, sequenced reads with low quality or adaptor contamination were removed. Clear reads were mapped to the human reference genome (hg19) using Hisat2 v2.0.5 software. To count the read numbers mapped to each gene, featureCounts v1.5.0-p3 was used. The fragments per kilobase of transcript per million mapped reads (FPKM) method was used to estimate expression levels. Differential expression analysis was performed using the DESeq2 R package (1.20.0).

### Cut&run ChIP-sequencing analysis

Rv1 cells were transduced with sh-Control or sh-SMAD3 lentivirus for 48 hours. Two biological replicates (5 × 10^5^ living cells per replicate) were used for ChIP-seq preparation, following the CUT&RUN kit protocol (EpiCypher). In brief, cell nuclei were extracted using nuclear extraction buffer (20 mM HEPES, pH 7.9, 10 mM KCl, 0.1% Triton X-100, 20% glycerol, 1X protease inhibitor cocktail) and incubated with ConA Beads to absorb nuclei onto beads. Antibody buffer and 0.5 μg of antibody were added to samples and incubated overnight at 4°C. After washing beads, 2.5 μl of pAG-NMase was added and incubated for 10 min at RT. After washing, 1 μl of chromatin digest additive was added and incubated for 2 hours at 4°C. Stop buffer and 0.5 ng of Spike-in DNA were then added and incubated for 10 min at 37°C. DNA was purified using the DNA purification kit (EpiCypher). 5 ng of purified DNA was subjected to the library preparation using the Illumina library prep kit (NEB). The library was purified with 1.0× AMPure beads (Beckman) and library fragment size was evaluated using an Agilent Bioanalyzer. Libraries were sequenced (PE150, 20 million reads per library) at Novogene Corporation (Sacramento, CA).

Quality of ChIP-seq reads was checked using FastQC software, and Trim Galore was used to remove adaptor sequences. After trimming, reads with a score >28 were aligned to the human hg19 reference genome and *E. coli* K12, MG1655 reference genome using Bowtie2. Sequencing data was normalized using a normalization factor calculated by sequencing the depth of *E. coli* Spike-in DNA reads. SAM files from reads aligned to hg19 were converted to BAM files, and PCR duplicates were removed. Spike-in normalized bigwig and bedgraph files were generated by deepTools. Bedgraph files were used for peak calling with MACS2 bdgpeakcall. Two replicates of peak files were combined with IDR, and ENCODE blacklist regions were removed. Peaks were annotated by Homer annotatePeaks.pl. The ChIPpeakAnno package in R was used to determine peaks overlapping in two groups. DeepTools was used to create the heatmap and profile plot. Homer v4.11 was used to analyze enrichment of Smad Binding Elements (SBEs), Androgen-Response Elements (AREs) and AR half-sites. For SBE motif scanning, we used FIMO default setting of MEME suite v5.4.1.

### Bioinformatic analysis

Gene ontology (GO) term analysis was performed using the Enrichr web server with differentially expressed genes from RNA-seq or annotated peaks from ChIP-seq. The EnhancedVolano R package was used to create volcano plots. BART web server (20) was used to predict transcriptional regulators underlying differentially expressed genes. GEPIA2 web server was used to analyze the correlation of AR and SMAD3 mRNAs in the TCGA PCa dataset. GEO datasets were downloaded from GEO database to compare the frequency of AR upstream enhancer and AR intron 3 enhancer or evaluate the mRNA levels of AR and SMAD3 in human PCa samples. The H3K27ac ChIP-seq datasets were downloaded as bigwig files. The ATAC-seq datasets were downloaded as raw sequencing files, and adaptor sequences were trimmed with Trim Galore. After trimming, reads with a score > 30 were aligned to the human hg19 reference genome using Bowtie2. SAM files from reads aligned to hg19 were converted to BAM files, and PCR duplicates were removed. For visualization, bigwig files were generated by deepTools.

### Plasmids

Flag-AR, myc-AR, Flag-AR mutants (N-TAD, DBD, LBD) in the pcDNA3 vector, AR or AR-V7 in the pLvx-IRES-zsGreen1 vector, and AR shRNA in pLKO.1 vector targeting the AR N-terminal region were described previously (21–23). The Flag-SMAD3 plasmid was a gift of Dr. Jeff Wrana (Addgene plasmid # 11742). SMAD3 was subcloned into Flag-pcDNA3 or myc-pcDNA3 vector. SMAD3 truncation mutants (ΔN, lacking the MH1 domain (133–425) or ΔC, lacking the MH2 domain (1–225)) were generated by PCR and subcloned into Flag-pcDNA3. pLV hU6-sgRNA hUbC-dCas9-KRAB-T2a-Puro plasmid was a gift of Dr Charles Gersbach (Addgene plasmid #71236). shRNAs in pLKO.1 vector targeting SMAD3 (TRCN0000330127, TRCN0000330128), SMAD2 (TRCN0000010477) and SMAD4 (TRCN0000040031) were from Sigma-Aldrich (St. Louis, MO). Plasmids were confirmed by Sanger sequencing at Genewiz (South Plainfield, NJ).

### Lentiviral vector packaging and transduction

Lentiviral vectors harboring shRNAs, sgRNAs, or AR, AR-V7 cDNAs were packaged in 293T cells using calcium phosphate transfection. The supernatant containing lentiviral particles were collected 48 h after transfection. PCa cells were then transduced with supernatants in the presence of polybrene (8 μg/ml) for 24 h before replacement with fresh growth media. Cells were analyzed 48 hours post transduction. For CRISPRi studies, cells were selected for 7 days in puromycin (1 μg/ml) before analysis.

### Immunoprecipitation and western blotting

Immunoprecipitation and western blotting procedures were detailed previously (21,23).

### Real-time RT-PCR analysis

Total RNA from cells was prepared using a total RNA miniprep kit (Sigma-Aldrich, St. Louis, MO). cDNA was synthesized using random hexamers. SYBR Green qPCR analysis was performed using a Mx3005P QPCR system (Agilent Technologies). Primers for peptidylprolyl isomerase A (PPIA) served as an internal control. Biological triplicate samples were used for qPCR analysis, and independent experiments were repeated at least three times. Primers for qPCR analysis of human gene transcripts were: PPIA: 5’-GACCCAACACAAATGGTTC-3’, 5’-AGTCAGCAATGGTGATCTTC-3’; SMAD3: 5’-TGGACGCAGGTTCTCCAAAC-3’, 5’-CCGGCTCGCAGTAGGTAAC-3’; SMAD4: 5’-CTCATGTGATCTATGCCCGTC-3’, 5’-AGGTGATACAACTCGTTCGTAGT-3’; SMAD2: 5’-CGTCCATCTTGCCATTCACG-3’, 5’-CTCAAGCTCATCTAATCGTCCTG-3’; AR: 5’-CCATCTTGTCGTCTTCGGAAATGTTATGAAGC-3’, 5’-AGCTTCTGGGTTGTCTCCTCAGTGG-3’; AR-V7: 5’-CCATCTTGTCGTCTTCGGAAATGTTATGAAGC-3’, 5’-TTTGAATGAGGCAAGTCAGCCTTTCT-3’; KLK2: 5’-GGTCGGCACAACCTGTTTGA-3’, 5’-GCCCAGGACCTTCACAACAT-3’; KLK3: 5’-ACCAGAGGAGTTCTTGACCCCAAA-3’, 5’-CCCCAGAATCACCCGAGCAG-3’; NKX3-1: 5’-ACTTGGGGTCTTATCTGTTGGA-3’, 5’-CTCGATCACCTGAGTGTGGG-3’; SLC45A3: 5’-GACACTATGATGAAGGCGTTCG-3’, 5’-GAGAAGGTGAACCCGGTGAG-3’. TMPRSS2: 5’-CCTCTAACTGGTGTGATGGCGT-3’, 5’-TGCCAGGACTTCCTCTGAGATG-3’; PMEPA1: 5’-CTGAGCCACTACAAGCTGTCTG-3’, 5’-GGATTCCGTTGCCTGACACTGT-3’; STEAP1: 5’-CCCTTCTACTGGGCACAATACA-3’, 5’-GCATGGCAGGAATAGTATGCTTT-3; STEAP2: 5’-GGTCACTGTAGGTGTGATTGG-3’, 5’-ACCACATGATAGCCGCATCTAA-3’.

### ChIP-PCR

Cells were crosslinked using 1% formaldehyde for 7 min at RT and then quenched with 5 M glycine. Cell nuclei were extracted with lysis buffer 1 (50 mM HEPES–KOH, pH7.5, 140 mM NaCl, 1 mM EDTA, 10% glycerol, 0.5% NP-40, 0.25% Triton X-100, and a 1× protease inhibitor cocktail) and lysis buffer 2 (10 mM Tris-HCl, pH 8.0, 200 mM NaCl, 1 mM EDTA, and 0.5 mM EGTA, and a 1× protease inhibitor cocktail). Nuclear extracts were solubilized in shearing buffer (10 mM Tris-HCl, pH7.6, 1 mM EDTA, 0.5 mM EGTA, 0.1% SDS, and a 1× protease inhibitor cocktail) and sonicated to obtain 500-bp chromatin fragments, using a Covaris M220 sonicator. To prepare antibody-conjugated Protein A/G magnetic beads, beads were washed and incubated with 3 μg primary antibody or control IgG overnight at 4°C. 500 μg of lysate proteins were diluted 10-fold and incubated with antibody-conjugated Protein A/G magnetic beads overnight. After four washes, crosslinking was reversed, and DNA was purified using spin columns and subjected to real-time PCR analysis. ChIP-PCR was performed in biological triplicates, and independent experiments were repeated at least three times. Data were calculated as percentage of input. Real-time PCR primers for various regions of the AR gene were: primer1: 5’-AGGTCACAAGTGCCTAGAAATACA-3’, 5’-TTTGGCAAAGGCAATCTGGG-3’; primer2: 5’-AGAAATTGTGGGGTCTGGGC-3’, 5’-CCAAAAGAAAAGTACAGCCACCC-3’; primer3: 5’-CCCACCTCCACCATTTTCTCT-3’, 5’-CCCTGAGAAGTGGAAAGAAGAGTC-3’; primer4: 5’-CACACAGCCAAGGCCCTATC-3’, 5’-GCTTGCCACTTCGAGAAACA-3’; primer5: 5’-TAATGTGGCTTGGCATTGGC-3’, 5’-GAAAAGCAAGCGACTGAGCC-3’; primer6: 5’-AGCTCCCACAGGGGTCTAAT-3’, 5’-CAAGGGGCTTTCCAGTCCAT-3’; primer7: 5’-AGCAAGCCTCATTGTCCAGG-3’, 5’-ACTCCATTCATTCTGCCGGG-3’; primer8: 5’-GTCAGTTCCAGGTTGTATCTTTTT-3’, 5’-ACCAAAGAGAGAATTTTATTGCTCA-3’; primer9: 5’-CAGGATGCTCTACTTCGCCC-3’, 5’-AGCTTCACTGTCACCCCATC-3’. AR exon 2: 5’-AAGACCTGCCTGATCTGTGG-3’, 5’-TGCATGTGCAAGACCCTTTA-3’. Real-time PCR primers for AR target genes were: KLK3 enhancer: 5’-CCTAGATGAAGTCTCCATGAGCTACA-3’, 5’-GGGAGGGAGAGCTAGCACTTG-3’; KLK2 promoter: 5’-GGGAATGCCTCCAGACTGAT-3’, 5’-CTTGCCCTGTTGGCACCTA-3’.

### CRISPRi targeting of SBE sites

The following oligos targeting SBE sites were synthesized and cloned into the pLV hU6-sgRNA hUbC-dCas9-KRAB-T2a-Puro plasmid: sg1 (GGAGTGGCCAGGAGTGAGAC), sg2 (CCACAGGGGTCTAATGCCCC), sg3 (TGCTCTGACCCAAGACTAAC), sg4 (ACTAGCAAGCCTCATTGTCC), sg5 (CAAGTCGTCTAGCAACATCC), and sg6 (CTCTGCTGCAGACAGAACAG); sg of AR exon 2 (CACTATGGAGCTCTCACATG). Rv1 cells were transduced with lentivirus carrying CRISPRi constructs, either individually or in combination. After 48 h, cells were treated with puromycin (1 μg/ml) and cultured for 7 days to remove non-transduced cells. The remaining cells were used for real-time RT-PCR or ChIP-PCR analysis.

### 2D colony formation assay

PCa cells were seeded at low density into 6-well plates in duplicate or triplicate. After 2 weeks, cells were fixed in 3.5% paraformaldehyde and stained with 0.2% crystal violet. Cell colony images were taken with a scanner. The number of colonies (>100 μm in diameter) was determined in 8 or 12 higher-power fields.

### Soft agar assay

PCa cells were mixed with agar to a final concentration of 0.4% and layered on top of 0.8% agar in six-well plates. Triplicate plates were incubated for 3 weeks at 37°C. The number of colonies (>50 μm in diameter) was scored in 12 high-power fields.

### Xenograft models

Athymic nude mice were purchased from the Jackson Laboratory and housed in the animal facility at the University of Maryland School of Medicine. All experiments were approved by the Institutional Animal Care and Use Committee (IACUC # 0122010) and conducted following university's animal policy in accordance with NIH guidelines. 8-week-old male athymic nude mice (*n* = 10 per group) were subcutaneously injected with Rv1 cells (1 × 10^6^ cells/100 μl of 1:1 PBS:Matrigel). 5 weeks post injection, xenograft tumors were collected, and tumor weights measured using a digital balance.

### Statistical analysis

Experiment was performed in biological triplicate each time. Experiment was independently repeated at least 3 times except RNA-seq, ChIP-seq and xenograft studies. Biological triplicate from one representative experiment was used for quantification and statistical analysis. Quantification was presented as mean ± SD (*n* = 3). Sample size other than triplicate was indicated in the figure legend. Statistical analysis was performed using GraphPad Prism software. Student's *t*-test (2-tailed) was used to compare differences between two groups of datasets. One-way ANOVA was used to compare differences among more than two groups of datasets. For all statistical analyses, differences were labeled as ns, not significant; *P < 0.05; **P < 0.01; ***P < 0.001. *P* values <0.05 were considered statistically significant.

## RESULTS

### SMAD3 promotes expression of AR and AR target genes in PCa cells

**Figure 1.**
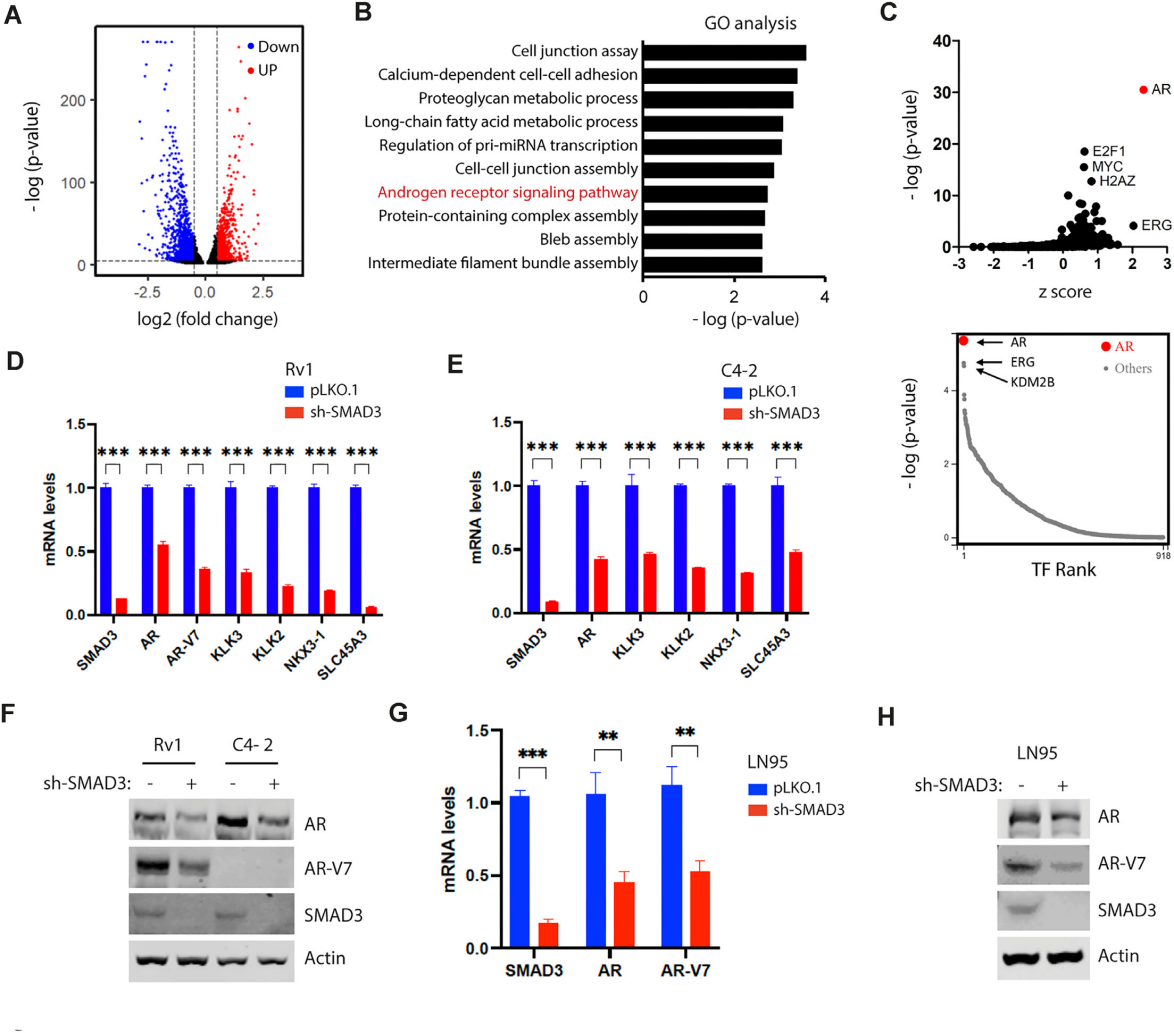
SMAD3 promotes the expression of AR and AR targets. (**A**) Volcano plot showing the differentially expressed genes between control and SMAD3-KD Rv1 cells in the RNA-seq analysis. (**B**) GO analysis of the downregulated genes after SMAD3 KD showing enrichment of the AR signaling pathway. (**C**) BART analysis of the downregulated genes after SMAD3 KD. AR is predicted to be a top transcription factor altered after SMAD3 KD. (**D**, **E**) Real-time RT-PCR results showing the reduced mRNA levels of AR, AR-V7 and example AR targets in the SMAD3-KD Rv1 (D) or C4-2 (E) cells. Quantification was presented as mean ± SD (*n* = 3), and *t* test was used for statistical analysis (***P < 0.001). (**F**) Western blots showing the reduced level of AR and AR-V7 in the SMAD3-KD Rv1 or C4-2 cells. (**G**) Real-time RT-PCR results showing the reduced mRNA level of AR and AR-V7 in the SMAD3-KD LN95 cells. Quantification was presented as mean ± SD (*n* = 3), and t test was used for statistical analysis (**P < 0.01; ***P < 0.001). (**H**) Western blots showing the reduced level of AR and AR-V7 in the SMAD3-KD LN95 cells.

To determine whether loss of SMAD3 function alters gene expression in PCa cells, we knocked down SMAD3 in AR-positive Rv1 cells for RNA-seq analyses. SMAD3 knockdown (KD) was confirmed by both real-time RT-PCR (Supplementary Figure S1A) and RNA-seq analyses (85% KD) (Supplementary Figure S1B). SMAD3 KD decreased levels of 1938 RNAs (log_2_ [fold change] < −0.5, *P*_adj_ < 0.05, Figure 1A, blue color) and upregulated levels of 1267 RNAs (log_2_ [fold change] > 0.5, *P*_adj_ < 0.05, Figure 1A, red color) (Supplemental Table S1). Gene Ontology (GO) analysis of downregulated genes revealed the top GO terms were related to cell adhesion and migration (Figure 1B), which are well-known SMAD3-related functions (10). Interestingly, AR signaling was also a GO pathway altered by SMAD3 KD (Figure 1B). To predict transcription factors regulated by SMAD3, we subjected genes significantly downregulated upon SMAD3 KD (log_2_[fold change] < −1, *P*_adj_ < 0.05) to BART (Binding Analysis for Regulation of Transcription) analysis, an algorithm that can predict key transcriptional regulators underlying altered gene expression. Significantly, AR was predicted to be the most significant transcription regulator altered by SMAD3 KD (Figure 1C).

To validate RNA-seq results, we performed real-time RT-PCR analysis of AR and AR target genes (namely, KLK3, KLK2, NKX3-1, SLC45A3) in Rv1 and C4-2 cells. SMAD3 KD in both lines decreased mRNA levels of both AR and target genes (Figure 1D, E). AR-V7 is a major AR splicing isoform expressed in Rv1 cells (21). SMAD3 KD reduced AR-V7 transcript levels to a similar degree as that of AR (Figure 1D). SMAD3 KD also reduced AR protein levels in Rv1 and C4-2 cells and AR-V7 protein levels in Rv1 cells (Figure 1F). To exclude potential off-target effects of shRNA, we repeated these analyses using a different SMAD3 shRNA (SMAD3 sh-2) and found that it also decreased transcript levels of AR, AR-V7, and AR target genes in Rv1 cells, in the presence or absence of androgen (Supplementary Figure S1C, D). Similarly, in C4-2 cells, transduction of SMAD3 sh-2 reduced transcript levels of AR and AR target genes in the presence or absence of androgen (Supplementary Figure S1E, F), although in the case of target genes that difference was not statistically significant in the absence of androgen (Supplementary Figure S1F). LN95 cells, which are derived from LNCaP cells, express both AR and AR-V7. SMAD3 KD in LN95 cells reduced AR and AR-V7 transcript and protein levels (Figure 1G, H). Together, these observations indicate that SMAD3 promotes expression of AR, AR-V7 and AR targets in PCa cells.

### SMAD3, but not SMAD4 or SMAD2, is a key regulator of AR signaling in PCa cells

**Figure 2.**
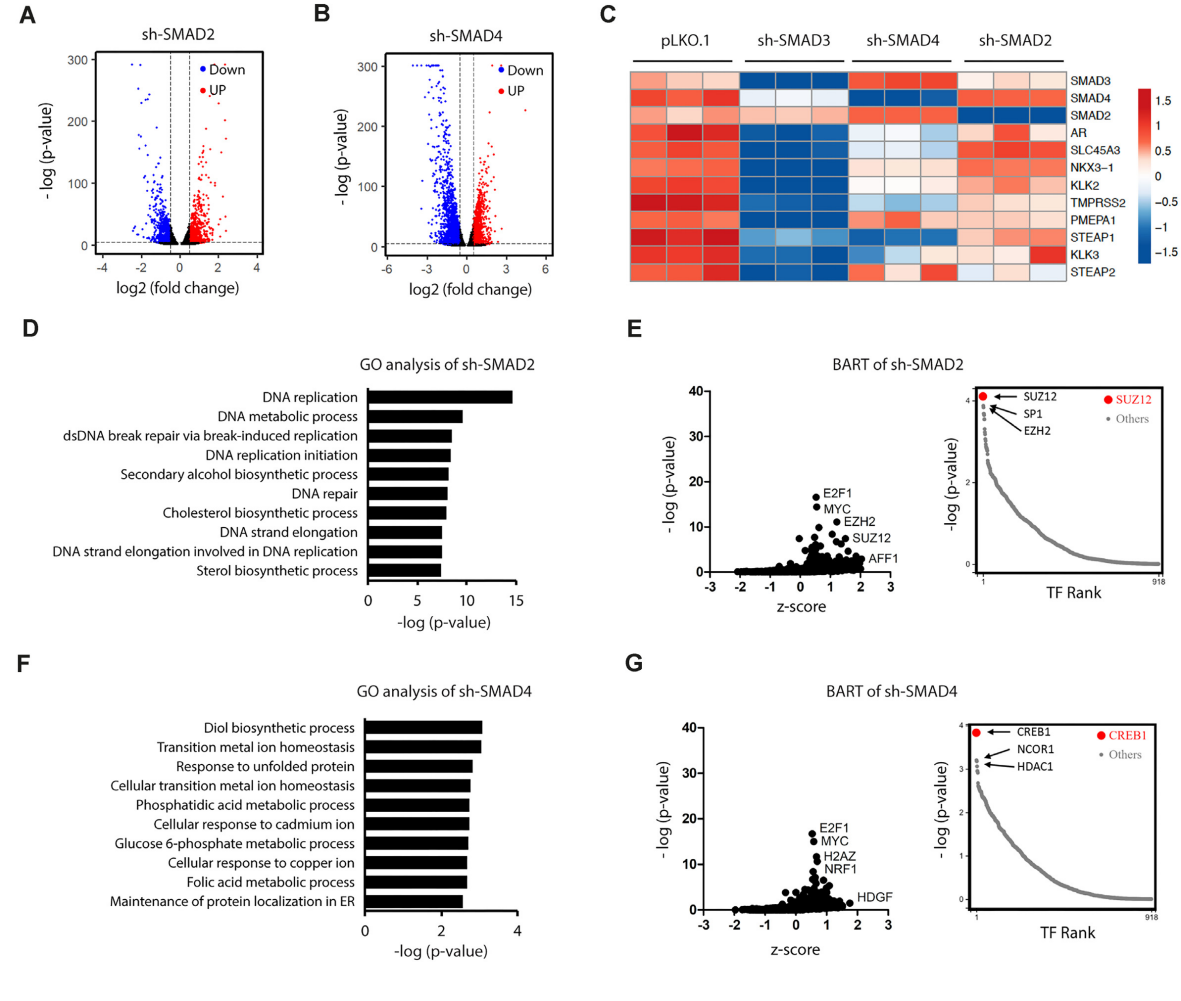
SMAD4 or SMAD2 has little or no effect on the expression of AR and AR targets. (**A**, **B**) Volcano plot of RNA-seq results showing the differentially expressed genes upon KD of SMAD2 (A) or SMAD4 (B) in Rv1 cells. (**C**) Heatmap showing the altered level of AR and classic AR target genes in the RNA-seq analysis of SMAD3-KD, SMAD4-KD or SMAD2-KD Rv1 cells. The FPKM value of RNA-seq results was log_2_ transformed and used for the heatmap preparation using the pheatmap package in R. (**D**) GO analysis of the downregulated genes after SMAD2 KD. (**E**) BART analysis of the downregulated genes after SMAD2 KD to predict the altered transcription factors. (**F**) GO analysis of the downregulated genes after SMAD4 KD. (**G**) BART analysis of the downregulated genes after SMAD4 KD to predict the altered transcription factors.

To evaluate whether other SMADs regulate AR signaling, we knocked down either SMAD2 or SMAD4 in Rv1 cells for RNA-seq analyses. After confirming the knockdown of SMAD2 or SMAD4 by real-time RT-PCR (Supplementary Figure S2A, B), we subjected the triplicate samples for RNA-seq analyses, which showed 77% and 70% KD of SMAD2 and SMAD4, respectively (Supplementary Figure S2C, D). SMAD2 KD decreased levels of 1281 RNAs (log_2_ [fold change] < −0.5, *P*_adj_ < 0.05, Figure 2A blue color) and upregulated levels of 1147 RNAs (log_2_ [fold change] > 0.5, *P*_adj_ < 0.05, Figure 2A red color) (Supplemental Table S2). SMAD4 KD decreased levels of 2698 RNAs (log2 [fold change] < −0.5, *P*_adj_ < 0.05, Figure 2B, blue color) and upregulated levels of 1837 RNAs (log_2_ [fold change] > 0.5, *P*_adj_ < 0.05, Figure 2B, red color) (Supplemental Table S3). Figure 2C shows a heatmap comparing changes in AR and AR target RNAs after KD of SMAD3, SMAD4 or SMAD2. SMAD3 KD significantly reduced mRNA levels of AR and AR targets (Figure 2C), SMAD4 KD had only minimal effects on mRNA levels of AR and some AR targets, and SMAD2 KD had almost no effect on either (Figure 2C). GO and BART analyses showed that neither SMAD2 KD (Figure 2D, E) nor SMAD4 KD (Figure 2F, G) altered AR signaling, results that differed from those seen after SMAD3 KD (Figure 1B, C). These results indicate overall that SMAD3, but not SMAD2 or SMAD4, is a key regulator of AR expression and activity in PCa cells.

### Regulation of AR expression by SMAD3 does not require TGF-β signaling

**Figure 3.**
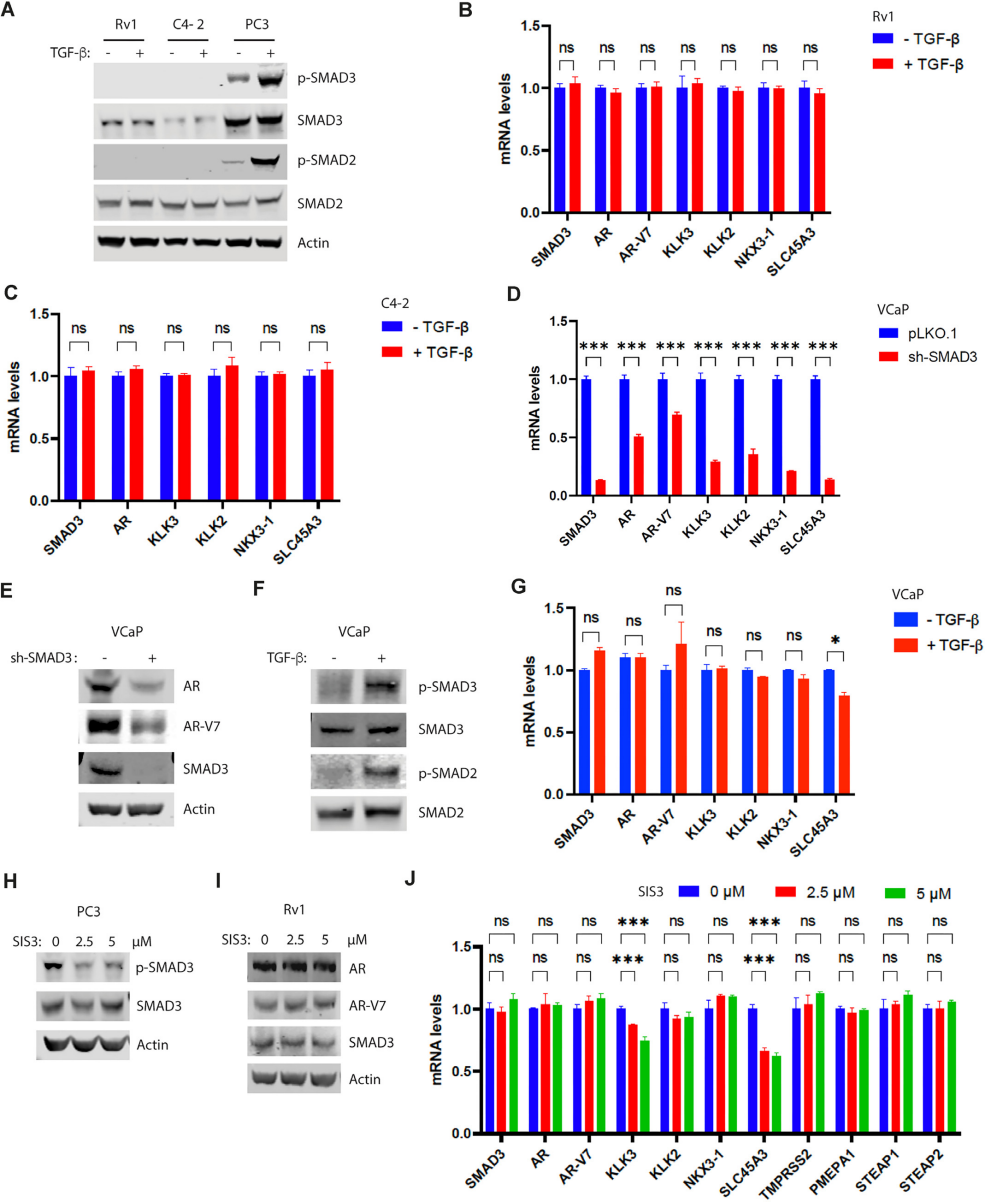
TGF-β treatment has no effect on the expression of AR and AR targets. (**A**) TGF-β did not induce phospho-SMAD3 or phospho-SMAD2 in Rv1 or C4-2 cells. Indicated cells were treated with 10 ng/ml of TGF-β for 1 h. Western blot analysis was performed using the indicated antibodies. (**B** and **C**) Real-time RT-PCR results showing that TGF-β treatment (10 ng/ml for 6 h) of Rv1 (B) or C4-2 (C) cells had no effect on transcript levels of AR, AR-V7 and AR targets. Quantification was presented as mean ± SD (*n* = 3), and t test was used for statistical analysis (ns, not significant). (**D**) Real-time RT-PCR results showing the decreased transcript levels of AR, AR-V7 and AR targets in the SMAD3-KD VCaP cells. Quantification was presented as mean ± SD (*n* = 3), and t test was used for statistical analysis (***P < 0.001). (**E**) Western blots showing the decreased level of AR and AR-V7 in the SMAD3-KD VCaP cells. (**F**) Western blots showing the increased levels of phospho-SMAD3 and phospho-SMAD2 in VCaP cells after TGF-β treatment (10 ng/ml for 1 h). (**G**) Real-time RT-PCR analysis of VCaP cells showing no effect of TGF-β treatment (10 ng/ml for 6 h) on transcript levels of AR, AR-V7 and AR targets relative to untreated cells. Quantification was presented as mean ± SD (*n* = 3), and *t* test was used for statistical analysis (ns, not significant; *P < 0.05). (**H**) Decreased levels of phospho-SMAD3 after treating PC3 cells with SIS3 for 24 h. (**I**) No changes of AR or AR-V7 levels after treating Rv1 cells with SIS3 for 24 h. (**J**) Effect of SIS3 treatment on transcript levels of AR, AR-V7 and AR target genes. Rv1 cells were treated with indicated concentration of SIS3 for 24 h before real-time RT-PCR analysis. Quantification was presented as mean ± SD (*n* = 3), and ANOVA was used for statistical analysis (ns, not significant; ***P < 0.001).

SMAD3 is a mediator of the TGF-β signaling pathway (10). To determine whether TGF-β mediates SMAD3 effects on AR signaling, we treated PCa cells (Rv1, C4-2, or PC3) with TGF-β and performed western blot analysis of phospho-SMAD3 and phospho-SMAD2, which are known markers of TGF-β signaling activation. We detected both phospho-SMAD3 and phospho-SMAD2 in AR-negative PC3 cells in the absence of TGF-β treatment but those levels significantly increased after TGF-β treatment (Figure 3A). By contrast, phospho-SMAD3 and phospho-SMAD2 were undetectable in AR-positive Rv1 and C4-2 cells, regardless of TGF-β treatment (Figure 3A), indicating the lack of detectable TGF-β signaling in Rv1 and C4-2 cells. Real-time RT-PCR analysis indicated comparable levels of AR or AR target gene transcripts in Rv1 or C4-2 cells treated or untreated with TGF-β (Figure 3B, C). TGF-β treatment reportedly activates phospho-SMAD3 in AR-positive VCaP cells (24). SMAD3 KD in VCaP cells decreased levels of AR, AR-V7 and AR target genes (Figure 3D, E). We observed induction of phospho-SMAD3 and phospho-SMAD2 in VCaP cells after TGF-β treatment (Figure 3F). However, we detected comparable levels of AR, AR-V7 or AR target transcripts in VCaP cells treated or untreated with TGF-β (Figure 3G). SIS3 is an inhibitor that blocks TGF-β-induced SMAD3 phosphorylation (25). Consistently, treatment of PC3 cells with 2.5 or 5 μM of SIS3, a commonly used dose, reduced levels of phospho-SMAD3 (Figure 3H). SIS3 treatment of Rv1 cells showed no effect on levels of AR, AR-V7 and most AR target transcripts, while slightly decreasing levels of two AR target transcripts (Figure 3I, J). Together, these results indicate that TGF-β signaling is not required for SMAD3-mediated regulation of AR expression in any PCa lines tested.

### SMAD3 binds to AR gene intron 3 to promote AR mRNA expression

**Figure 4.**
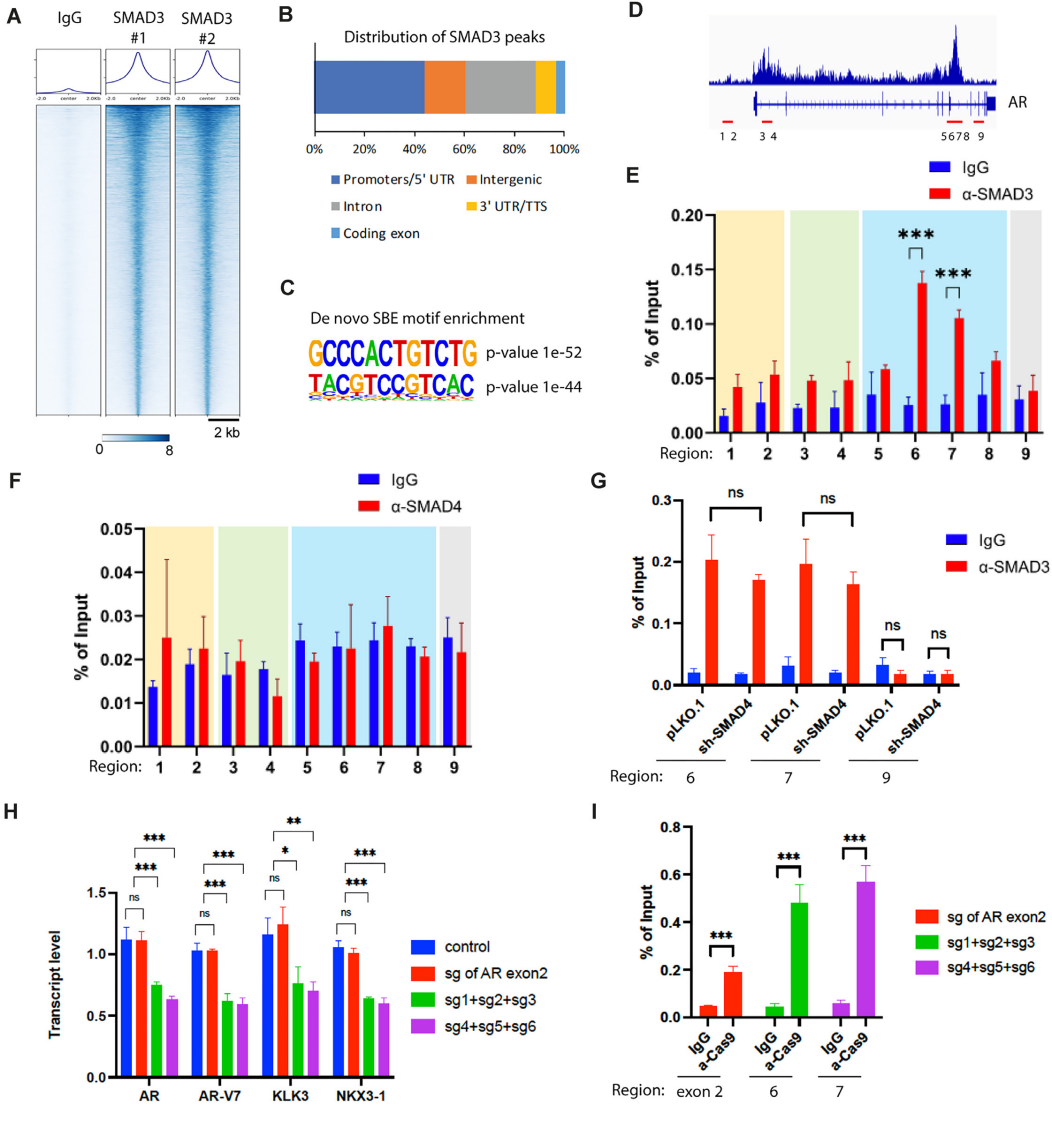
SMAD3 binds to intron 3 of AR gene to promote AR mRNA expression. (**A**) Heatmap showing two replicates of SMAD3 ChIP-seq peaks relative to IgG negative control antibody. (**B**) SMAD3 ChIP-seq peak annotation. (**C**) The Homer motif analysis showing the significant enrichment of SBE motifs on SMAD3 peaks. (**D**) Image showing the SMAD3 ChIP-seq peaks at the AR gene. The red lines and numbers indicate the nine regions analyzed by ChIP-PCR as described in (E). (**E**) ChIP-PCR of SMAD3 showing the enrichment of SMAD3 at regions 6 and 7 that are located at the center of the major SMAD3 ChIP-seq peak. ChIP assays were performed on Rv1 cells with control and SMAD3 antibodies. Precipitated chromatin was analyzed by qPCR for the nine regions of AR gene as indicated in D. % of input was calculated and presented as mean ± SD (*n* = 3), and t test was used for statistical analysis (***P < 0.001). The comparison at other regions (1–5,8,9) was not significant. (**F**) ChIP-PCR of SMAD4 showing no enrichment of SMAD4 at regions 6 and 7. ChIP assays were performed with control and SMAD4 antibodies as described in E. % of input was calculated and presented as mean ± SD (*n* = 3), and *t* test was used for statistical analysis. The comparison at each of the 9 regions was not significant. (**G**) ChIP-PCR of SMAD3 showing the similar SMAD3 enrichment at regions 6 and 7 of AR gene between control and SMAD4-KD Rv1 cells. % of input was calculated and presented as mean ± SD (*n* = 3), and ANOVA was used for statistical analysis (ns, not significant). Region 9 serves as a negative control region for SMAD3 binding. (**H**) Real-time RT-PCR results showing that CRISPRi constructs targeting region 6 or 7 reduced the mRNA levels of AR, AR-V7 and AR targets in Rv1 cells. 3 sgRNAs (sg1,sg2,sg3) were simultaneously used to target the three SBEs at or near region 6. Three sgRNAs (sg4,sg5,sg6) were simultaneously used to target the 4 SBEs at region 7. Rv1 cells expressing the indicated CRISPRi constructs were analyzed by the real-time RT-PCR analysis of indicated genes. Quantification was presented as mean ± SD (*n* = 3), and ANOVA was used for statistical analysis (ns, not significant; *P < 0.05; **P < 0.01; ***P < 0.001). (**I**) ChIP-PCR of Cas9 showing the enrichment of Cas9 at AR exon 2, region 6 or 7 in the CRISPRi-expressing Rv1 cells as described in H. % of input was calculated and presented as mean ± SD (*n* = 3), and t test was used for statistical analysis (ns, not significant; ***P < 0.001).

To identify mechanisms underlying SMAD3 regulation of AR expression, we examined the genome-wide chromatin binding of SMAD3 using Cut&Run ChIP-seq (26). 11779 SMAD3 peaks were detected in the ChIP-seq by SMAD3 antibody, but not by control antibody (Figure 4A). 60% of SMAD3 peaks are located on promoters and intergenic regions (Figure 4B). Homer motif analysis of SMAD3 peaks revealed the significant enrichment of Smad Binding Element (SBE) motifs (Figure 4C). These results demonstrate the success of SMAD3 ChIP-seq studies. Interestingly, this analysis identified a minor SMAD3 peak in AR intron 1 and a major SMAD3 peak in AR intron 3 (Figure 4D). To validate these results and precisely map potential SMAD3 binding sites, we performed SMAD3 ChIP-PCR analysis using primers targeting various regions of the AR gene, as shown in Figure 4D. We observed SMAD3 enrichment at region 6 and 7, which are located near the center of the major SMAD3 ChIP-seq peak (Figure 4E). By contrast, SMAD4 ChIP-PCR showed no SMAD4 enrichment at region 6 and 7 (Figure 4F). SMAD3 ChIP-PCR showed similar levels of SMAD3 enrichment at region 6 and 7 between control and SMAD4-KD Rv1 cells (Figure 4G), indicating that SMAD4 is not required for the binding of SMAD3 to these chromatin sites. Based on previous crystal structure analysis, the SMAD3 MH1 domain recognizes and directly binds to half of palindromic GTCT or AGAC sequences, called the Smad Binding Element (SBE) (9). Multiple SBEs are found at or near region 6 and 7 (Supplementary Figure S3), suggesting that SMAD3 may bind to SBEs in this region to regulate AR expression.

To identify SBEs that may functionally regulate AR mRNA expression, we blocked access to SBEs using the CRISPR interference (CRISPRi) approach (27), in which nuclease-dead Cas9 (dCas9) is fused with the repressive chromatin modifier domain of KRAB (Krüppel associated box) and targeted to a SBE site, similar to the CRISPRd approach used in a previous study (28). Here, we chose to use CRISPRi as it may be more effective than CRISPRd in blocking SMAD3 binding to an SBE not only sterically but also via KRAB-mediated formation of repressive chromatin. For this analysis, we designed 3 sgRNAs separately targeting 3 SBEs at or near region 6, and 3 sgRNAs targeting 4 SBE sites at region 7 (of note: the last two SBE sites at region 7 can be simultaneously targeted by one sgRNA) (Supplementary Figure S3). The CRISPRi constructs without sgRNA or with sgRNA targeting the AR exon 2 were used as controls. We then used lentivirus to transduce Rv1 cells with the CRISPRi constructs and selected transduced cells in puromycin. When used alone, none of the 6 sgRNAs altered AR mRNA expression relative to controls (data not shown). However, simultaneous targeting the 3 SBEs at region 6 and the 4 SBEs at region 7 decreased transcript levels of AR, AR-V7 and AR targets in Rv1 cells, whereas the two control CRISPRi constructs showed no effect (Figure 4H). ChIP-PCR of Cas9 showed the enrichment of Cas9 at AR exon 2, region 6 or 7 in Rv1 cells expressing the corresponding CRISPRi constructs (Figure 4I), demonstrating the recruitment of dCas9-KRAB to the expected chromatin sites. Together, these results strongly suggest that SMAD3 binds to multiple SBE sites within AR intron 3 to activate transcription.

### SMAD3 and AR peaks overlap in ChIP-seq analysis

**Figure 5.**
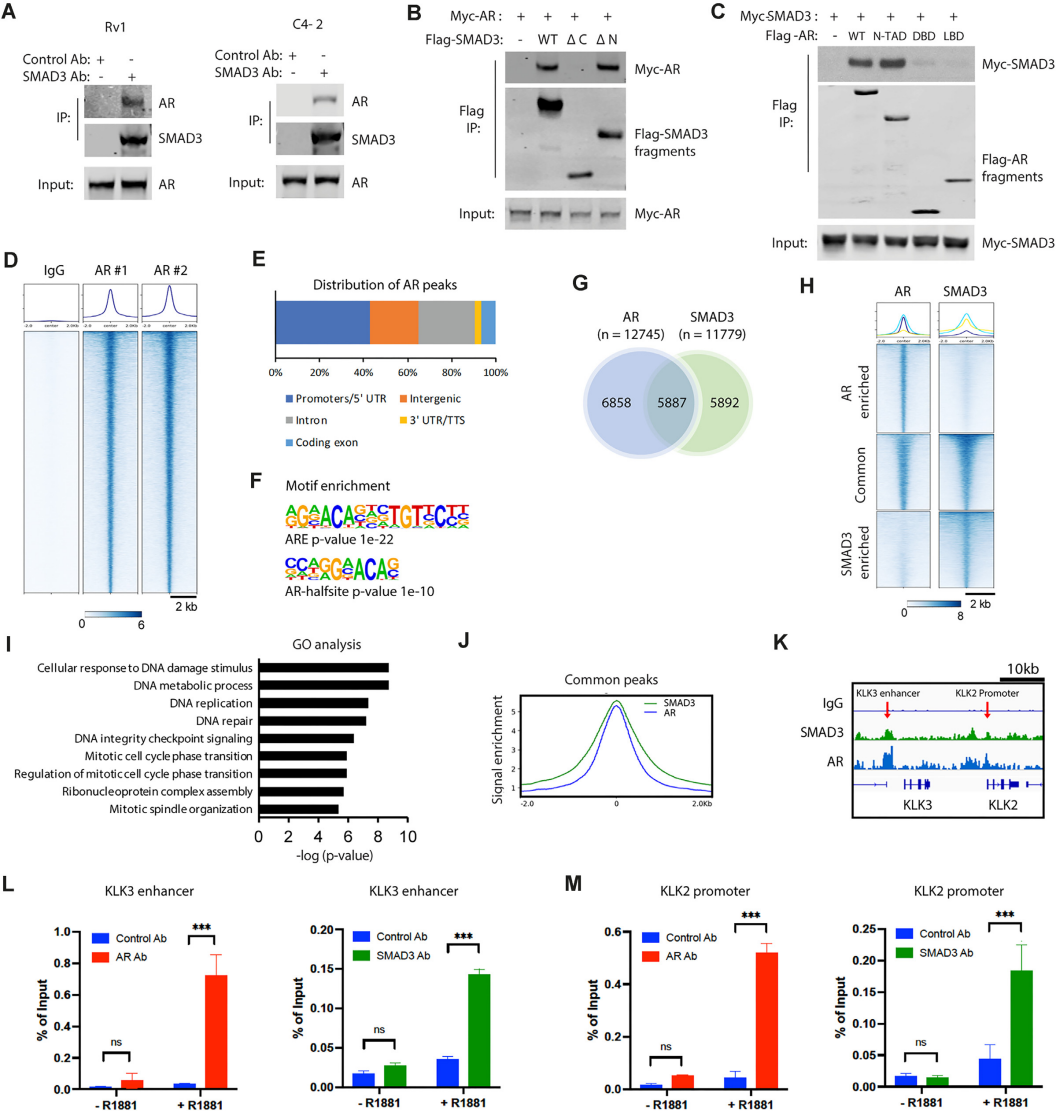
Overlaps of SMAD3 peaks and AR peaks in the ChIP-seq analysis. (**A**) Co-immunoprecipitation (Co-IP) of AR with SMAD3 in Rv1 or C4-2 cells. SMAD3 was immunoprecipitated from cells and analyzed by western blotting for co-precipitation of AR. Trueblot secondary antibodies were used in the western blots. (**B**) The C-terminal MH2 domain of SMAD3 interacted with AR. Myc-AR was co-expressed with Flag-tagged SMAD3 fragments (WT, Δ C mutant lacking the C-terminal MH2 domain, or Δ N mutant lacking the N-terminal MH1 domain) in 293T cells. Flag IP was performed and analyzed by western blotting with Flag or myc antibodies. (**C**) N-TAD domain of AR interacted with SMAD3. Myc-SMAD3 was co-expressed with the Flag-tagged AR fragments (N-terminal transactivation domain, N-TAD; DNA binding domain, DBD; Ligand-binding domain, LBD) in 293T cells, and analyzed as described in (B). (**D**) Heatmap showing two replicates of AR ChIP-seq peaks relative to IgG negative control antibody. (**E**) AR ChIP-seq peak annotation. (**F**) The Homer motif analysis showing the significant enrichment of ARE or AR half-site motifs on AR peaks. (**G**) Venn diagram showing the overlap of AR peaks and SMAD3 peaks in the ChIP-seq analysis. Cut&Run ChIP-seq studies were performed on Rv1 cells using AR or SMAD3 antibodies. Peak calling identified 12745 AR peaks and 11779 SMAD3 peaks. The overlapping of AR and SMAD3 peaks was determined by the ChIPpeakAnno package in R. (**H**) Heatmap showing the AR enriched peaks, common peaks and SMAD3 enriched peaks. (**I**) GO analysis of the genes associated with the common peaks between AR and SMAD3. (**J**) Distribution of AR (blue) and SMAD3 (green) ChIP-seq peak signal near the common peak center. (**K**) Example signal track image showing the SMAD3 peaks and AR peaks at the KLK3 enhancer or KLK2 promoter, which is indicated with an arrow. (**L** and **M**) ChIP-PCR showing the enrichment of AR and SMAD3 at KLK3 enhancer (L) or KLK2 promoter (M) of C4-2 cells after androgen treatment. ChIP assays were performed with control, AR or SMAD3 antibodies. Precipitated chromatin was analyzed by qPCR for KLK3 enhancer or KLK2 promoter. % of input was calculated and presented as mean ± SD (*n* = 3), and ANOVA was used for statistical analysis (ns, not significant; ***P < 0.001).

Previous studies report that ectopically over-expressed SMAD3 and AR proteins interact (17,19). Using co-immunoprecipitation (co-IP), we found that endogenous AR co-precipitated with SMAD3 in Rv1 or C4-2 cells (Figure 5A). Similar levels of AR co-precipitated with SMAD3 between control and SMAD4-KD Rv1 or C4-2 cells (Supplementary Figure S4A), indicating that SMAD4 is not required AR/SMAD3 interaction. Next, we expressed truncated mutants of SMAD3 or AR in 293T cells to determine which domains mediate their interaction. Based on co-IP analysis we found that the SMAD3 MH2 domain interacted with AR (Figure 5B), and the AR N-terminal transactivation domain (N-TAD) interacted with SMAD3 (Figure 5C), results consistent with a previous study mapping AR-SMAD3 interaction by *in vitro* binding of bacterially-generated fusion proteins (17). AR-V7 also co-precipitated with SMAD3 in Rv1 or LN95 cells (Supplementary Figure S4B), results consistent with the interaction of AR N-TAD with SMAD3. AR KD in Rv1 or LN95 cells showed no effect on levels of SMAD3 (Supplementary Figure S4C), indicating that AR/SMAD3 interaction does not affect the SMAD3 protein level.

To determine whether SMAD3-AR interaction occurs on AR target genes, we examined genome-wide chromatin binding of AR using Cut&Run ChIP-seq. 12 745 AR peaks were detected in the ChIP-seq by AR antibody, but not by control antibody (Figure 5D). 65% of AR peaks are located on promoters and intergenic regions (Figure 5E). Homer motif analysis of AR peaks revealed the significant enrichment of Androgen-Response Element (ARE) or AR half-site motifs (Figure 5F). These results demonstrate the success of AR ChIP-seq studies. We found that 46% of AR peaks (5887 of 12 745) overlapped with SMAD3 peaks, and 50% of SMAD3 peaks (5887 of 11779 peaks) overlapped with AR peaks (Figure 5G, H). GO analysis of genes marked by common peaks indicated they were primarily involved in DNA damage response, DNA replication and the cell cycle (Figure 5I). Profiling of common peaks showed that AR and SMAD3 peak centers overlapped (Figure 5J). For example, SMAD3 and AR peaks co-localized on the KLK3 enhancer and KLK2 promoter (Figure 5K). To assess whether the co-localization of AR and SMAD3 depends on androgen, we maintained C4-2 cells in the androgen-depleted media for 3 days and then treated cells with R1881 (5 nM) for 4 hours before ChIP-PCR analysis of AR and SMAD3. Under androgen depletion condition, AR and SMAD3 showed no enrichment at KLK3 enhancer (Figure 5L) or KLK2 promoter (Figure 5M). In contrast, after androgen treatment, AR and SMAD3 were enriched at KLK3 enhancer (Figure 5L) or KLK2 promoter (Figure 5M). These results suggest the requirement of ligand-activated AR for the binding of SMAD3 to KLK3 enhancer or KLK2 promoter in C4-2 cells.

To assess how AR and SMAD3 interact at AR target genes, we performed the SBE motif analysis on the 5887 common peaks that have AR Binding Sites (ARBS). 48.9% of common peaks have at least one SBE motif, while 51.1% of commons peaks have no SBE motif (Supplementary Figure S4D, E). The former result suggests the model that SMAD3 may bind to SBE via its MH1 domain and interact with AR via its MH2 domain, thereby cooperatively facilitating the chromatin binding of AR (Supplementary Figure S4E, left). The latter result suggests the model that SAMD3 may directly bind to AR and potentially function as a co-activator to promote the AR chromatin binding (Supplementary Figure S4E, right).

### SMAD3 knockdown decreases genome-wide AR binding

**Figure 6.**
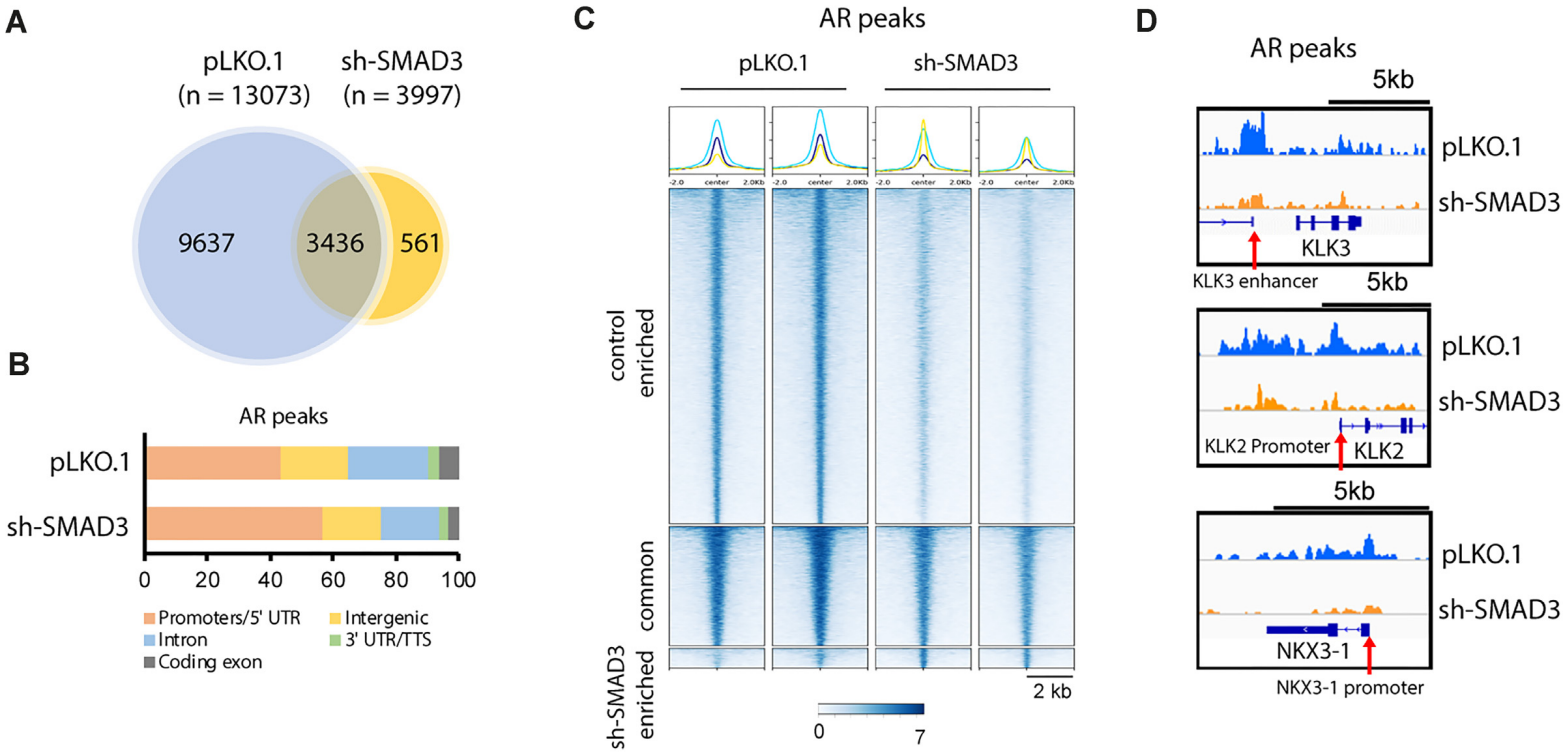
SMAD3 KD decreases the global ChIP-seq signal of AR. (**A**) Alteration of AR ChIP-seq peaks after SMAD3 KD in Rv1 cells. Cut&Run ChIP-seq studies were performed on Rv1 cells (control and SMAD3 KD) using AR antibodies. The alteration of AR peaks is shown in the Venn diagram including the control-enriched peaks, common peaks and SMAD3-KD-enriched peaks. (**B**) The genome distribution of AR peaks in control (*n* = 13073) and SMAD3-KD (*n* = 3997) Rv1 cells. (**C**) Heatmap showing the AR ChIP-seq peaks in control and SMAD3-KD Rv1 cells including the control-enriched peaks, common peaks and SMAD3-KD-enriched peaks. (**D**) Example signal track image showing the AR peak at KLK3 enhancer, KLK2 promoter or NKX3-1 promoter (indicated with an arrow) in control and SMAD3-KD Rv1 cells.

To determine whether SMAD3 loss alters AR binding to chromatin, we knocked down SMAD3 in Rv1 cells and performed Cut&Run ChIP-seq analysis of AR. Among AR peaks, ∼74% (9637 of 13 073) showed a statistically significant reduction in peak size after SMAD3 KD, while 26% (3436 of 13 073) showed reductions in peak size that were not statistically significant (Figure 6A–C). For example, SMAD3 KD decreased AR peak signals on the KLK3 enhancer, KLK2 promoter or NKX3-1 promoter (Figure 6D). We conclude that SMAD3 KD leads to a global decrease in AR binding to chromatin.

### AR re-expression in SMAD3-KD cells partially rescues both AR target gene expression and changes in PCa cell growth

**Figure 7.**
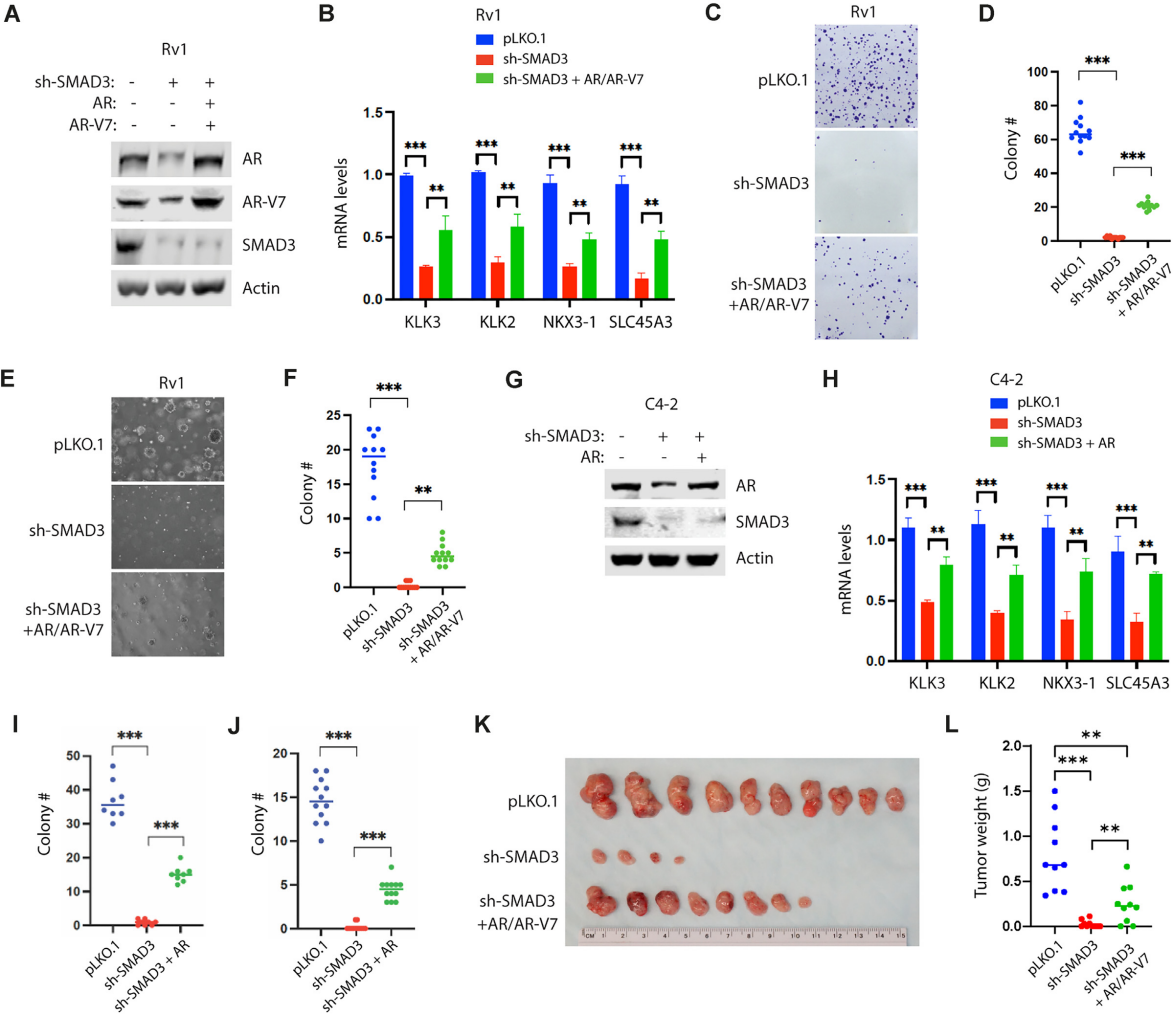
Re-expression of AR partly rescues the AR target expression and growth of SMAD3-KD PCa cells. (**A**) Western blot showing re-expression of AR and AR-V7 in the SMAD3-KD Rv1 cells. Optimal amounts of lentivirus were used to restore the expression of AR and AR-V7 in the SMAD3-KD cells to the levels seen in control cells. (**B**) Real-time RT-PCR results showing that re-expression of AR and AR-V7 in the SMAD3-KD Rv1 cells partly rescued the expression of example AR targets. Quantification was presented as mean ± SD (*n* = 3), and ANOVA was used for statistical analysis (**P < 0.01; ***P < 0.001). (**C** and **D**) Re-expression of AR and AR-V7 in the SMAD3-KD Rv1 cells partly rescued colony formation in cell-culture plates. The indicated cells were seeded at low density and maintained for 2 weeks. Colony numbers were scored in the 12 high-power fields. The example image represents 4 high-power fields. Colony number per field was presented as mean ± SD (*n* = 12), and ANOVA was used for statistical analysis (***P < 0.001). (**E** and **F**) Re-expression of AR and AR-V7 in the SMAD3-KD Rv1 cells partly rescued the colony formation in soft agar. The indicated cells were grown in soft agar for 3 weeks and colony numbers were scored in 12 high-power fields. The example image represents 1 high-power field. Colony number per field was presented as mean ± SD (*n* = 12), and ANOVA was used for statistical analysis (**P < 0.01; ***P < 0.001). (**G**) Western blot showing re-expression of AR in the SMAD3-KD C4-2 cells. (**H**) Real-time RT-PCR results showing that re-expression of AR in the SMAD3-KD C4-2 cells partly rescued the expression of example AR targets. Quantification was presented as mean ± SD (*n* = 3), and ANOVA was used for statistical analysis (**P < 0.01; ***P < 0.001). (**I**) Re-expression of AR in the SMAD3-KD C4-2 cells partly rescued the colony formation in cell-culture plates. The procedure is as described in C and D. Colony numbers were scored in 8 high-power fields. Colony number per field was presented as mean ± SD (*n* = 8), and ANOVA was used for statistical analysis (***P < 0.001). (**J**) Re-expression of AR in the SMAD3-KD C4-2 cells partly rescued the colony formation in soft agar. The procedure is as described in E and F. Colony number per field was presented as mean ± SD (n = 12), and ANOVA was used for statistical analysis (***P < 0.001). (**K** and **L**) Re-expression of AR and AR-V7 in the SMAD3-KD Rv1 cells partly rescued the xenograft tumor formation. The indicated cells (1 × 10^6^) were subcutaneously injected into athymic nude mice (*n* = 10 per group). Xenograft tumors were collected at 5 weeks post injection. The image (K) and weight (L) of xenograft tumors are shown. Tumor weight was presented as mean ± SD (*n* = 10), and ANOVA was used for statistical analysis (**P < 0.01; ***P < 0.001).

We have shown that SMAD3 KD in PCa cells decreases AR transcript expression (Figure 1). To functionally assess effects of SMAD3-mediated AR expression, we restored AR expression in SMAD3-KD PCa cells. To do so, we first transduced SMAD3-KD Rv1 cells with lentivirus encoding AR and AR-V7 sufficient to restore expression to levels comparable to those seen in control cells (Figure 7A). Rescue of AR and AR-V7 expression partially increased levels of transcripts of AR targets in SMAD3-KD Rv1 cells (Figure 7B). Next, we asked whether SMAD3 loss altered colony formation capacity in cell-culture plate or soft agar and observed that SMAD3 KD almost abolished colony formation by Rv1 cells (Figure 7C–F). By contrast, re-expression of AR and AR-V7 in SMAD3-KD Rv1 cells partially restored colony formation (Figure 7C–F). Next, we restored AR expression in SMAD3-KD C4-2 cells (Figure 7G) and observed partial rescue of both AR target gene expression and colony formation (Figure 7H–J). SMAD3 KD in Rv1 cells significantly decreased the frequency and size of xenograft tumor formation upon injection into athymic nude mice (Figure 7K, L). By contrast, re-expression of AR and AR-V7 in the SMAD3-KD Rv1 cells partially restored the frequency and size of xenograft tumor formation (Figure 7K, L). These results support the idea that in PCa cells, SMAD3 plays an important role in tumor cell growth by mediating expression of AR and its targets.

### The SMAD3 peak in AR intron 3 may function as an enhancer of the AR gene in PCa

**Figure 8.**
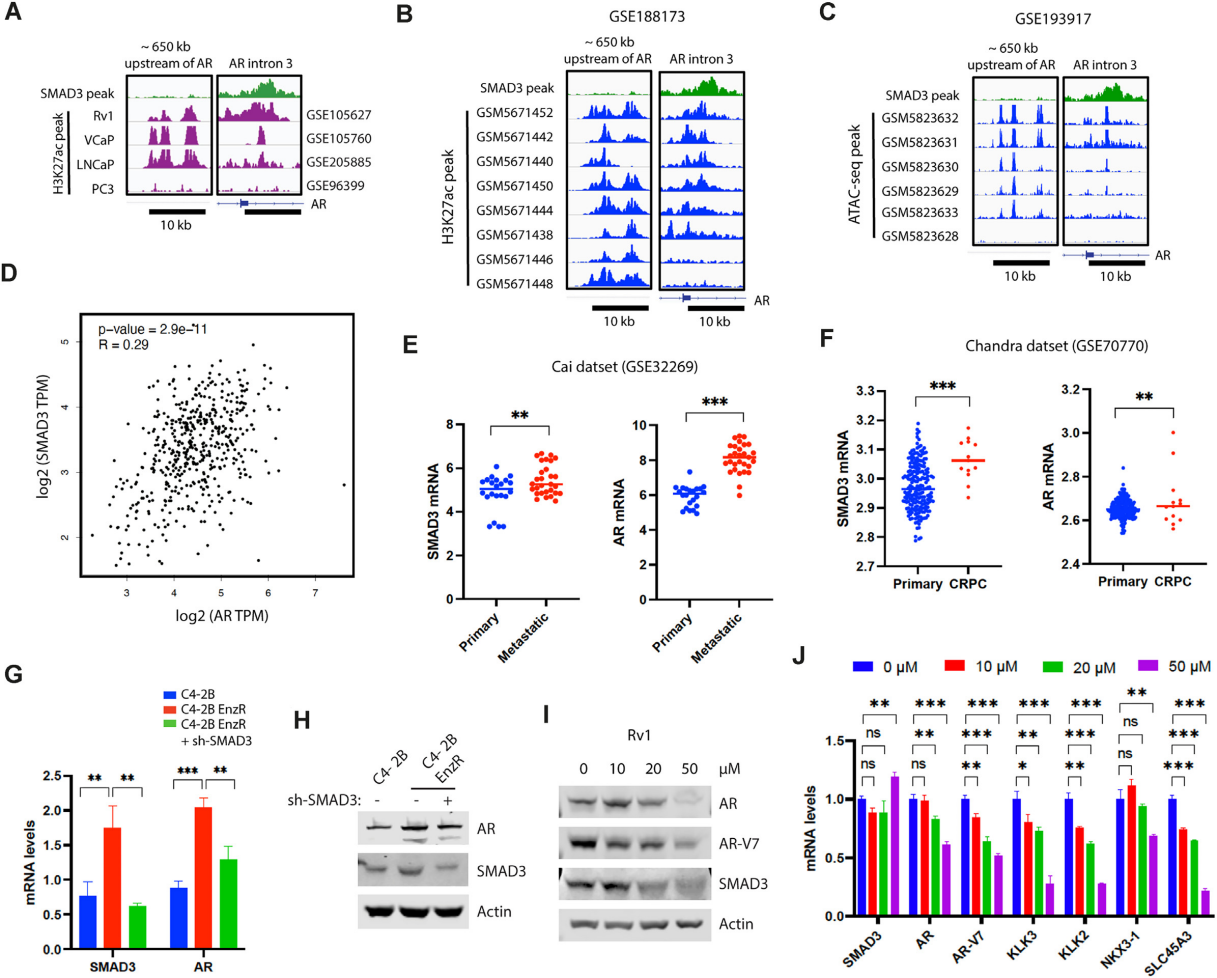
SMAD3 promotes the expression of AR mRNA in human PCa and can be targeted with PROTAC inhibitor. (**A**) The SMAD3 peak in AR intron 3 overlapped with the H3K27ac peak in human PCa cells. The H3K27ac peaks in AR upstream enhancer are also shown. The indicated datasets of H3K27ac ChIP-seq were download from GEO database and visualized by IGV software. (**B**) H3K27ac peaks in AR upstream enhancer and AR intron 3 enhancer in a published dataset of 8 CRPC PDX samples. (**C**) ATAC-seq peaks in AR upstream enhancer and AR intron 3 enhancer in a published dataset of 6 PCa PDX samples. (**D**) Positive correlation (Pearson correlation, *R* = 0.29, *P*-value = 2.9e-11) between AR mRNA and SMAD3 mRNA in the TCGA PCa dataset (n = 492). The correlation analysis was performed using the web server of GEPIA2. (**E** and **F)** Upregulation of AR mRNA and SMAD3 mRNA in metastatic PCa (E) or CRPC (F) relative to the primary PCa. The indicated datasets of profiling array studies on human PCa tissues were downloaded from the GEO database. Cai dataset includes 22 primary PCa and 29 metastatic PCa. Chandra dataset includes 207 primary PCa and 13 CRPC. The intensity values of AR and SMAD3 were Log2 transformed, and the mean values of the indicated PCa groups were compared. Quantification was presented as mean ± SD, and t test was used for statistical analysis (**P < 0.01; ***P < 0.001). (**G** and **H**) Partial knockdown of SMAD3 reduced the levels of AR mRNA (G) and protein (H) in the enzalutamide-resistant C4-2B cells (EnzR). Quantification was presented as mean ± SD (*n* = 3), and ANOVA was used for statistical analysis (**P < 0.01; ***P < 0.001). (**I** and **J**) A SMAD3 PROTAC inhibitor decreased levels of AR, AR-V7 and AR targets. Rv1 cells were treated with the indicated concentration of SMAD3 PROTAC inhibitors for 24 hours before western blot (I) or real-time RT-PCR analysis (J). Quantification was presented as mean ± SD (*n* = 3), and ANOVA was used for statistical analysis (ns, not significant; *P < 0.05; **P < 0.01; ***P < 0.001).

To determine whether the SMAD3 peak in AR intron 3 functions as an enhancer of the AR gene, we analyzed the published datasets of H3K27 acetylation (H3K27ac) ChIP-seq or ATAC-seq, both of which are enhancer markers. The SMAD3 peak in AR intron 3 overlapped with the H3K27ac peak in AR-positive PCa cells (Rv1, VCaP or LNCaP), which also displayed H3K27ac peaks in AR upstream enhancer (650 kb upstream of the AR gene) (Figure 8A). By contrast, AR-negative PC3 cells did not display H3K27ac peaks in either AR intron 3 or AR upstream enhancer (Figure 8A). These results support the idea that the SMAD3 peak in AR intron 3 may function as an enhancer of the AR gene. We next compared the relative abundance of AR intron 3 enhancer and AR upstream enhancer in human PCa samples. In a dataset of CRPC PDX models, 8 out of 8 samples displayed H3K27ac peaks in AR upstream enhancer, while 6 out of 8 samples displayed H3K27ac peaks in AR intron 3 enhancer (Figure 8B). In another dataset of PCa PDX models, 5 out of 6 samples displayed ATAC-seq peaks in AR upstream enhancer, while 4 out of 6 samples displayed ATAC-seq peaks in AR intron 3 enhancer (Figure 8C). Thus, these two datasets of PCa samples showed comparable levels of AR upstream enhancer and AR intron 3 enhancer. By contrast, other datasets of PCa samples showed differential levels of AR upstream enhancer and AR intron 3 enhancer. A dataset of 22 PCa PDX models showed H3K27ac peaks more frequently in AR upstream enhancer (20/22) than AR intron 3 enhancer (7/22) (Supplementary Figure S5A), while another dataset of 22 PCa organoid models showed ATAC-seq peaks more frequently in AR intron 3 enhancer (12/22) than AR upstream enhancer (2/22) (Supplementary Figure S5B). Together, these results support the importance of both AR upstream enhancer and AR intron 3 enhancer in human PCa.

### SMAD3 and AR mRNA levels are upregulated in advanced PCa

To determine the biological significance of SMAD3 regulation of AR expression, we analyzed and correlated SMAD3 and AR mRNA levels in human PCa datasets. SMAD3 and AR mRNA levels were positively correlated in TCGA PCa datasets (Figure 8D). In two GEO datasets, SMAD3 and AR mRNA levels were increased in metastatic PCa (Figure 8E) or CRPC (Figure 8F) relative to primary PCa. We next established enzalutamide-resistant C4-2B PCa cell lines by growing C4-2B cells in the presence of gradually increasing doses of enzalutamide for more than 1 year. We observed increased mRNA and protein levels of AR and SMAD3 in enzalutamide-resistant relative to parental C4-2B cells (Figure 8G, H). Partial SMAD3 KD in enzalutamide-resistant C4-2B cells to levels seen in parental cells partly reduced AR mRNA and protein levels (Figure 8G, H). These results suggest that increased SMAD3 levels may contribute to increased AR levels in enzalutamide-resistant C4-2B cells. Together, these findings suggest that SMAD3 may promote expression of AR mRNA in human PCa and contribute to increased levels of AR mRNA in advanced PCa.

### A SMAD3 PROTAC inhibitor reduces levels of AR and AR targets in PCa cells

SMAD3 PROTAC inhibitors have been developed to induce the degradation of SMAD3 by recruiting VHL ubiquitin ligase (29). We tested the effect of a SMAD3 PROTAC inhibitor on the expression of AR and AR targets in Rv1 cells. The SMAD3 PROTAC inhibitor reduced levels of SMAD3 protein in Rv1 cells with concomitant reduction in the levels of AR, AR-V7 and AR targets (Figure 8I, J). The results indicate that SMAD3 PROTAC inhibitors can be used to repress AR level and activity in PCa cells.

## DISCUSSION

AR overexpression is one of the main drivers in converting castration-sensitive to castration-resistant PCa. However, mechanisms underlying transcriptional upregulation of AR mRNA are not well understood. Several transcription factors reportedly bind to the AR promoter to positively or negatively regulate AR transcription (8). Liganded AR reportedly binds to a site in AR intron 2 to repress AR transcription, a mechanism that may account for increased AR transcription under androgen deprivation conditions (30). Here we find that SMAD3 binds to sites in AR intron 3 to promote expression of AR mRNA to a similar degree in the presence or absence of androgen. SMAD3 and AR mRNAs are positively correlated in the TCGA PCa dataset, and both SMAD3 and AR mRNAs are upregulated in metastatic PCa or CRPC relative to primary PCa. SMAD3 and AR transcripts were upregulated in enzalutamide-resistant PCa cells, and partial SMAD3 knockdown decreased AR mRNA levels. These findings overall support the notion that SMAD3 plays a key role in transcriptional upregulation of AR mRNA in CRPC.

The SMAD3 peak in AR intron 3 overlaps with H3K27ac peaks or ATAC-seq peaks in human PCa cells or PCa tissues. CRISPRi-mediated blocking of SMAD3-binding sites in AR intron 3 inhibited expression of AR mRNA in PCa cells. These findings indicate that the SMAD3 peak in AR intron 3 functions as an enhancer to promote AR mRNA expression. AR intron 3 enhancer and AR upstream enhancer show either comparable or differential pattern in various PCa datasets, supporting the importance and relevance of AR intron 3 enhancer in human PCa.

We also show that SMAD3-mediated expression of AR mRNA is independent of TGF-β signaling in PCa cells. In addition to SMAD3-SMAD4 heterodimers, SMAD3 is known to function as a homodimer (31) or as a heterodimer with structurally unrelated transcription factors such as c-Myc, p53, β-catenin, and STAT3 (32–35). Here, we excluded the possibility that SMAD3-SMAD4 heterodimers regulate AR transcription. However, future work is needed to determine whether SMAD3 regulates transcription of AR alone or in concert with some of the other transcription factors.

Previous studies reported that SMAD3 MH2 domain and AR N-TAD domain interact, overexpression of SMAD3 can increase the expression of AR target gene KLK3, and SMAD3 overexpression can activate or repress activities of androgen-responsive reporters (17–19). Thus, SMAD3 has been established as a positive AR co-regulator through AR/SMAD3 interaction. The established key principles by previous studies are rediscovered in this paper. Our ChIP-seq analysis reveals the co-localization of AR and SMAD3 peaks on chromatin of PCa cells. 48.9% of peaks common to AR and SMAD3 have SBE motifs. Thus, SMAD3 may bind to an SBE via its MH1 domain, while its MH2 domain interacts with AR, to cooperatively facilitate the chromatin binding of AR (Supplementary Figure S4E). On the other hand, 51.1% of peaks common to AR and SMAD3 have no SBE motifs. In this case, SMAD3 may directly interact with AR and co-activate the chromatin binding of AR (Supplementary Figure S4E). Global reduction of AR chromatin binding after SMAD3 KD reflects inhibition of AR expression, while we cannot exclude the possibility that loss of SMAD3-mediated cooperation or co-activation of AR may also contribute to the reduced AR chromatin binding. Although phospho-SMAD3 is below the detection limit in AR-positive PCa cells, we find that the SMAD3 phosphorylation inhibitor SIS3 moderately inhibits expression of selected AR target genes. Future work is needed to determine whether phospho-SMAD3 regulates AR/SMAD3 interaction on a subset of AR target genes.

AR-V7 generation is a key mechanism driving development of enzalutamide resistance. AR-V7 splicing occurs co-transcriptionally with transcription of AR pre-mRNA (36). Thus, increased AR-V7 transcript levels could be due to either increased transcription of AR pre-mRNA or increased AR-V7 splicing. In our study, SMAD3 KD comparably reduced AR and AR-V7 transcript levels. These results support our conclusion that SMAD3 promotes transcription of AR pre-mRNA, leading to simultaneous increases in AR and AR-V7 mRNA levels.

Our study demonstrates a tumor-promoting role of SMAD3 in PCa cell growth dependent on the AR pathway. RNA-seq and subsequent bioinformatic analysis of SMAD3-KD Rv1 cells did not predict changes in SMAD or TGF-β signaling, potentially due to the lack of detectable TGF-β signaling in Rv1 cells. Instead, AR and AR signaling pathways were predicted to be altered by SMAD3 KD. AR re-expression in SMAD3-KD PCa cells partially rescued defects in AR target expression and PCa cell growth *in vitro* and in xenograft models. These results indicate that AR is a key downstream effector in SMAD3-mediated gene expression and PCa cell growth. Neuroendocrine prostate cancer (NEPC) is a lethal form of prostate cancer characterized by loss of AR expression and thus resist ADT. In the AR-negative PC3 cells, SMAD3 was reported to promote HIF-1α chromatin binding under hypoxia to promote neuroendocrine (NE) phenotype (15). Thus, targeting SMAD3 may potentially inhibit both AR-positive CRPC and AR-negative NEPC.

In Summary, we identified a previously uncharacterized function of SMAD3 in regulating transcription of AR mRNA and demonstrated AR as a key downstream effector in SMAD3-dependent PCa progression. We also provide evidence that a SMAD3 PROTAC inhibitor can be used to inhibit expression of AR, AR-V7 and AR target genes. The present findings support that SMAD3 could be targeted to modulate/inhibit AR expression and activity in advanced PCa.

## DATA AVAILABILITY

Raw data related to RNA-seq and ChIP-seq have been deposited in the GEO database with accession number GSE211946.

## Supplementary Material

gkad043_Supplemental_File
