# Supplementary material for: SMAD3 promotes expression and activity of the androgen receptor in prostate cancer
Source: Nucleic Acids Res. 2023 Feb 2;51(6):2655–70. doi: 10.1093/nar/gkad043 (PMC10085708; doi:10.1093/nar/gkad043)
Supplement: gkad043_Supplemental_File [file gkad043_supplemental_file.pdf]

SMAD3 promotes expression and activity of the androgen receptor in prostate cancer

Hee-Young Jeon, Majid Pornour, Hyunju Ryu, Sudeep Khadka, Rui Xu, Jihyun Jang,

Deqiang Li, Hegang Chen, Arif Hussain, Ladan Fazli, Martin Gleave, Xuesen Dong,

Furong Huang, Qianben Wang, Christopher Barbieri, Jianfei Qi

Supporting Online Material

Supplemental Figures and Tables

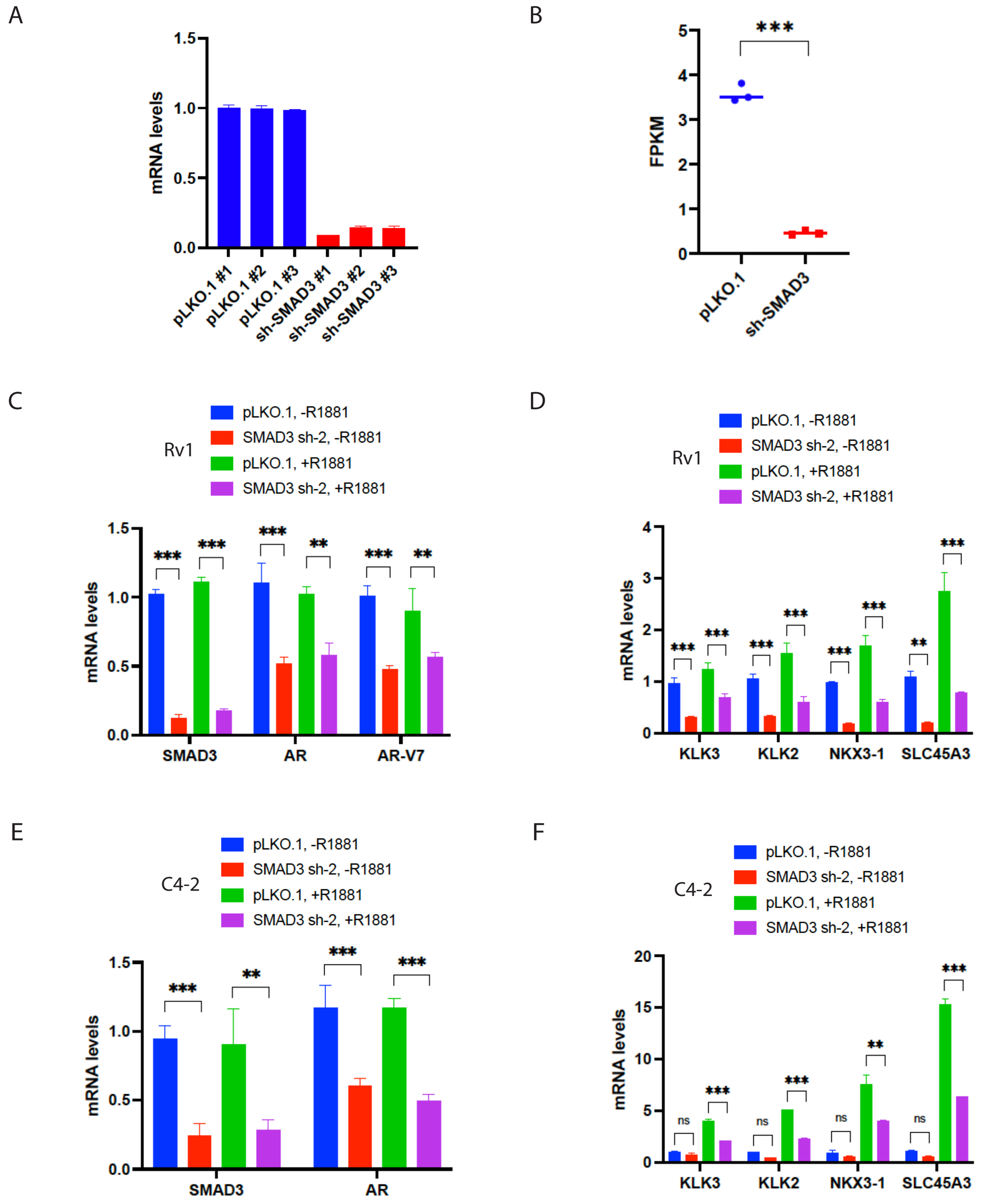

Figure S1. SMAD3 promotes the expression of AR and AR targets in the presence or absence of androgen. A. Real-time RT-PCR results showing the efficiency of SMAD3 KD in the indicated triplicate samples of Rv1 cells before RNA-seq analysis. B. RNA-seq results showing the efficiency of SMAD3 KD in the indicated triplicate samples of Rv1 cells. ***, p<0.001 (t test). C and D. Knockdown of SMAD3 with another shRNA reduced the mRNA levels of AR and AR-V7 (C) or example AR targets (D) in Rv1 cells in the presence or absence of synthetic androgen R1881. Rv1 Cells (control or SMAD3 KD) were maintained in the absence (5% charcoal stripped FBS) or presence (5% charcoal stripped FBS, 1 nM R1881) of androgen for 24 hours before real-time RT-PCR analysis. E and F. Knockdown of SMAD3 with another shRNA reduced the mRNA levels of AR (E) and example AR targets (F) in C4-2 cells in the presence or absence of androgen (R1881). The procedure is as described in C and D. Quantification in C to F was presented as mean ± SD (n=3), and ANOVA was used for statistical analysis (ns, not significant; **, p<0.01; ***, p<0.001).

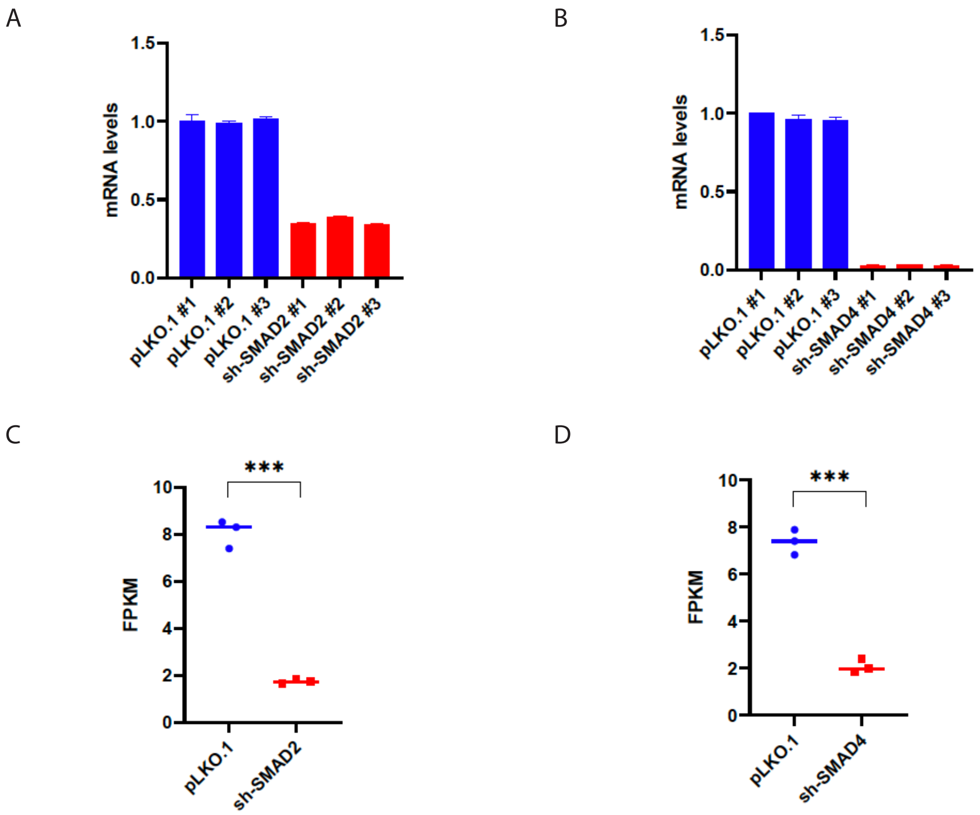

Figure S2. Knockdown efficiency of SMAD2 and SMAD4 in Rv1 cells.
A and B. Real-time RT-PCR results showing the KD of SMAD2 (A) or SMAD4 (B) in the indicated triplicate samples before RNA-seq analysis. C and D. RNA-seq results showing the KD of SMAD2 (C) or SMAD4 (D) in the indicated triplicate samples. ***, $p<0.001$ (t test).

TCACGCCACTTTCACCTTTTCCTTAATCATGGTTGAGAAGGCCTATATCTTGGAGTGGCCAGGAGTG**AGA**
CTGGAACAGTACCTAAAGGTTAAGGACGCTAAAGAAGTTACAGATTGGTTACATCTGCTCCTCCCTAGG
AATGATCCATGGAACCTGATTTGAAATTTTTTTCTCTGGTGCTATAGATAGCTCCCACAGGG**GTCT**AATG
CCCCAGGGCTGAAAAGTTAGTTCCCCATAGGATCCATCCAGGCATGATATCAGGCCAGGTGTTACAATC region 6
TCCTAAAGAGGAGGTATGGACTGGAAAGCCCCTTGCCAATGGCCCTTTCTTGTCACTGCTCTGACCCA**AG**
ACTAACAGGGCAGAGATAGTGAACTCACATACTATTAAAACTATCCACTTATACTTCCCCCTTTCTCTTTG
CTTTATCACTCCATTTAAGTAAACCAATGAGTCTCTGCCTTGACACAGTGGCAAGCTGACCTGTATCTTAT
ATGAAAGAATTAGATTTGACTCTGGGGCTCAGGTGCAGAGGGCAGGAGGGGCATAAGGATGGCCTTCA
TGGAAGAAAAGAAGTCCTTGGATACTGAGTAACAGCTG**AGAC**TAGCAAGCCTCATTGTCCAGGATTCCA
AGTC**GTCT**AGCAACATCCTG**GTCT**CTGCTGC**AGAC**AGAACAGAGGATCCCCCGGCAGAATGAATGGAG region 7
TCTGATTTCAATTACGTTCAGTATAGTCACTCTCTTTAGGCAGAGAAGCCAGAACACCTGGTGCAGCTAG
GGCCACTGTGGTCACAGGGACAAGCACACTACCTGGGTCCTGGAGGCAAGTGGGAATGCAGTTTTTCTT

Figure S3. DNA sequence near regions 6 and region 7 of AR intron 3. The two underlined sequences are region 6 and region 7 that show the highest enrichment of SMAD3 by ChIP-PCR. The SBEs (AGAC or GTCT) are bolded and highlighted with green and yellow, respectively. Three sgRNAs (sg1, sg2, sg3) are designed to target the 3 SBEs at or near region 6. Three sgRNAs (sg4, sg5, sg6) are designed to target the 4 SBEs at region 7.

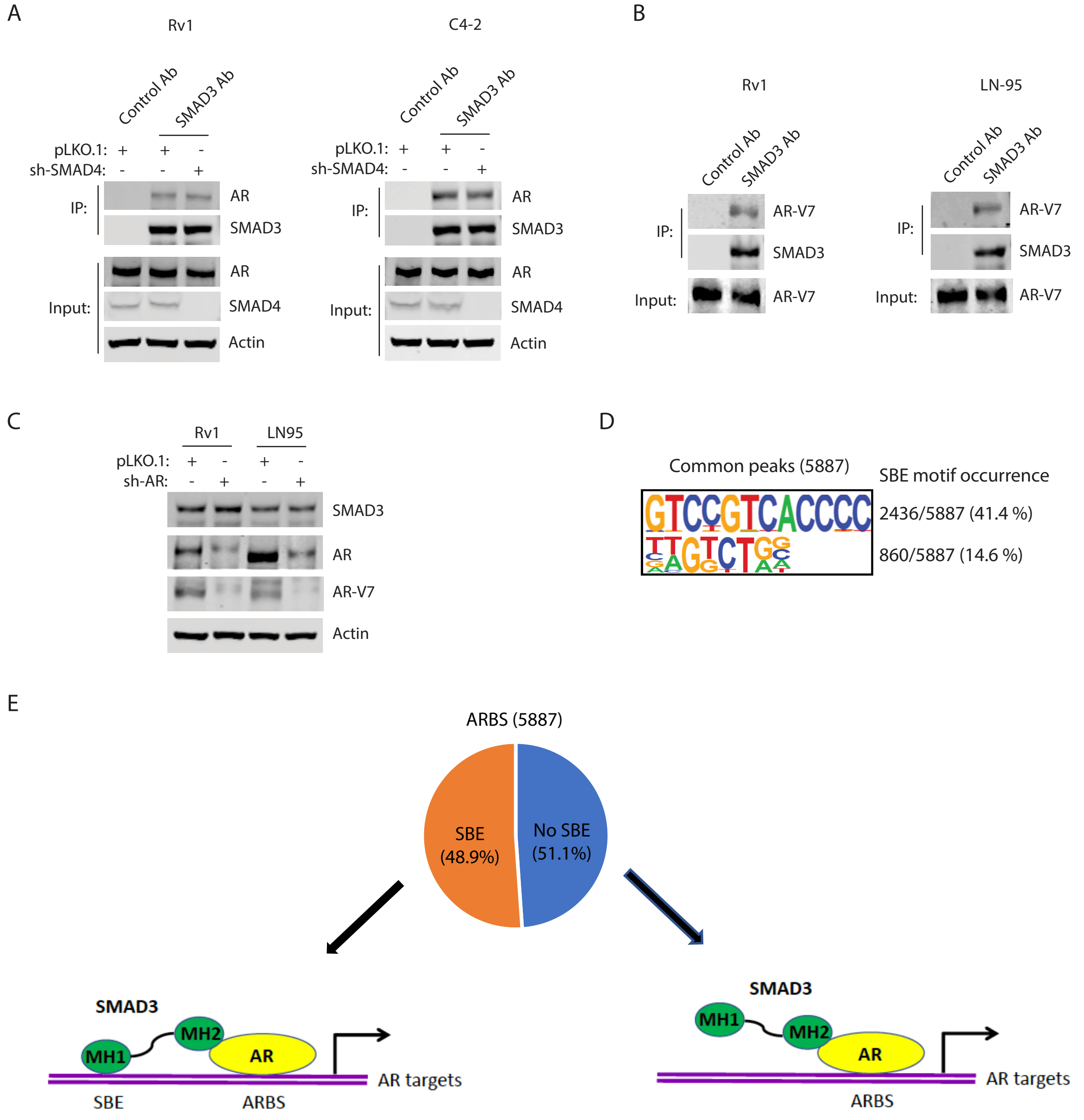

Figure S4. Interaction of SMAD3 with AR or AR-V7 and model of AR-SMAD3 interaction at AR target genes.
A. SMAD4 KD had no effect on the co-IP of AR with SMAD3 in Rv1 or C4-2 cells. B. Co-IP of AR-V7 with SMAD3 in Rv1 or LN95 cells. C. AR KD had no effect on the protein level of SMAD3 in Rv1 or LN95 cells. D. SBE motifs in the common peaks of AR and SMAD3. 41.4% and 14.6% of common peaks have one of two SBE motifs, respectively. Overall, 48.9% of common peaks have at least one SBE motif (2882/5887). E. Model of AR-SMAD3 interaction at AR target sites. Left: among the 5887 peaks having the AR Binding Sites (ARBS), 48.9% have SBE motifs. Under such circumstance, SMAD3 may use its MH1 domain to interact with SBE, and its MH2 domain to interact with N-TAD of AR, to cooperatively facilitate the binding of AR to ARBS. Right: among the 5887 peaks having the ARBS, 51.1% have no SBE motifs. In this case, SMAD3 may directly interact with AR and potentially function as an AR co-activator to promote AR binding to ARBS.

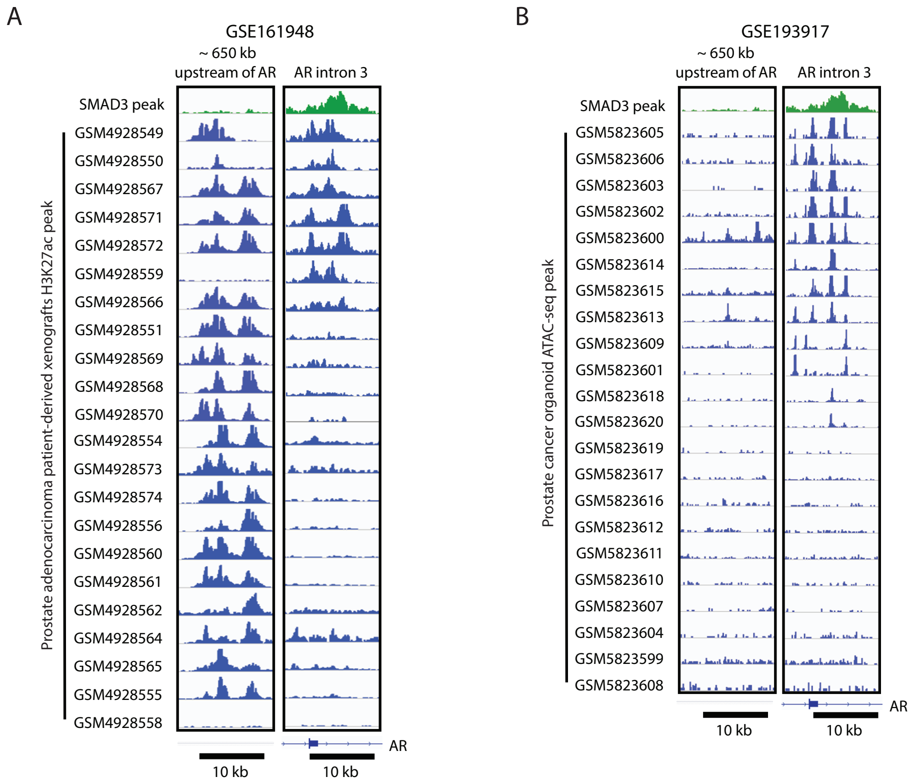

Figure S5. Differential H3K27ac or ATAC-seq peaks at AR upstream enhancer and AR intron 3 enhancer in the published datasets of samples derived from human prostate cancer.
A. H3K27ac peak in a published dataset of human prostate cancer PDX models (n=22). B. ATAC-seq peaks in published dateset of prostate cancer organoid models (n=22). Highlighted regions are AR upstream enhancer (left panel) and AR intron 3 enhancer (right panel).

Table S1. Downregulated and upregulated genes upon SMAD3 KD in Rv1 cells

GeneName	log2FoldChange	pvalue	padj
ADH1B	-2.8415	1.22E-26	2.97E-25
SLC45A3	-2.8194	2.04E-177	1.83E-174
CDS2	-2.739	2.10E-156	1.26E-153
SMAD3	-2.7323	7.28E-102	1.55E-99
MAP4	-2.6803	0	0
NKX3-1	-2.6798	0	0
FAM213B	-2.6587	1.57E-67	1.58E-65
GTF3A	-2.6046	4.90E-232	6.91E-229
BGN	-2.6027	1.94E-21	3.61E-20
RHOC	-2.5544	1.18E-246	2.12E-243
CMTM7	-2.5261	4.17E-105	9.56E-103
NFIX	-2.5006	0	0
CCKBR	-2.4666	6.94E-39	2.92E-37
COL21A1	-2.3975	2.87E-19	4.73E-18
CYP1A2	-2.3039	8.98E-35	3.26E-33
RINL	-2.2995	6.42E-109	1.56E-106
CTNNBIP1	-2.2655	3.81E-111	9.90E-109
SLC25A24	-2.152	4.55E-87	7.17E-85
CYP4F22	-2.1107	2.44E-13	2.66E-12
TMEM100	-2.0978	4.65E-12	4.48E-11
C11orf92	-2.0903	0	0
CP	-2.0609	3.29E-20	5.72E-19
ADRBK2	-2.0602	1.23E-72	1.45E-70
RPL4	-2.0593	0	0
KLK2	-2.0384	3.30E-64	3.00E-62
FEZ2	-2.0299	9.66E-68	9.92E-66
ALDH3B1	-2.0208	2.51E-64	2.32E-62
RP11-521B24.3	-1.9957	9.97E-15	1.22E-13
CHRNA2	-1.9853	1.23E-61	1.01E-59
RBBP9	-1.957	2.28E-111	6.01E-109
PKP1	-1.9268	1.16E-58	9.14E-57
CALD1	-1.9248	0	0
TMPRSS2	-1.9231	1.12E-67	1.14E-65
CD44	-1.902	1.05E-93	1.92E-91
NEFH	-1.9001	3.48E-129	1.25E-126
PMEL	-1.8774	1.67E-13	1.85E-12
IFT81	-1.8605	4.17E-52	2.75E-50
PRUNE2	-1.8548	2.01E-112	5.50E-110
KCNJ8	-1.8543	6.06E-23	1.23E-21
DEFB132	-1.8451	4.04E-09	2.94E-08
PGAP2	-1.8352	1.50E-103	3.26E-101
LSM3	-1.8341	1.04E-154	5.86E-152

PRR16	-1.8204	4.24E-69	4.43E-67
CORO2A	-1.8068	4.48E-37	1.76E-35
APOLD1	-1.8011	1.20E-26	2.94E-25
EHF	-1.7826	1.43E-135	6.00E-133
AKAP5	-1.7716	3.04E-16	4.19E-15
SLBP	-1.7654	6.04E-217	7.45E-214
STX2	-1.7517	8.76E-47	4.93E-45
H2AFV	-1.7289	2.35E-273	5.81E-270
FSTL1	-1.7277	1.36E-25	3.16E-24
FAM64A	-1.7194	4.90E-78	6.41E-76
UCP1	-1.7138	6.85E-09	4.86E-08
PTGFR	-1.7065	2.11E-44	1.08E-42
SYT4	-1.703	8.59E-223	1.13E-219
ATG4C	-1.7027	4.14E-40	1.86E-38
ACER3	-1.6973	5.17E-61	4.22E-59
ISOC1	-1.693	4.85E-86	7.42E-84
PMEPA1	-1.6877	1.74E-170	1.37E-167
RP11-775D22.3	-1.6834	1.09E-09	8.41E-09
ZNF93	-1.683	1.03E-35	3.84E-34
RGS4	-1.682	4.81E-08	3.11E-07
MBOAT2	-1.6783	5.23E-45	2.77E-43
PRX	-1.6613	1.39E-15	1.83E-14
FAM189B	-1.657	3.10E-130	1.16E-127
CCDC160	-1.6341	1.13E-12	1.15E-11
QKI	-1.6332	4.75E-89	7.87E-87
AC073415.2	-1.6327	6.57E-08	4.17E-07
CYB5A	-1.6324	6.02E-85	8.99E-83
BTBD1	-1.6276	2.47E-190	2.44E-187
LRRC8C	-1.6242	5.06E-22	9.75E-21
C10orf82	-1.6198	9.71E-14	1.09E-12
EFNA2	-1.6191	8.02E-24	1.70E-22
TXNDC12	-1.6138	1.43E-104	3.21E-102
C18orf54	-1.6102	5.67E-46	3.09E-44
EPB41L1	-1.5794	9.71E-79	1.28E-76
CCDC43	-1.5781	1.87E-49	1.13E-47
ABCC6P1	-1.5755	1.55E-43	7.82E-42
LL22NC03-86G	-1.5747	2.89E-10	2.37E-09
MEX3A	-1.5745	1.91E-111	5.09E-109
HDGFRP3	-1.5726	1.53E-69	1.64E-67
LPAR3	-1.5715	1.06E-54	7.43E-53
GTF3C2	-1.5681	2.46E-114	7.13E-112
SLC2A4RG	-1.5596	1.66E-114	4.90E-112
RLN2	-1.5568	8.99E-13	9.27E-12
AFAP1L2	-1.5512	9.03E-17	1.28E-15
GBAS	-1.5487	1.68E-105	3.90E-103

KTN1	-1.5483	5.55E-124	1.85E-121
YBX3	-1.5468	1.45E-239	2.19E-236
SLC4A4	-1.5447	2.62E-21	4.84E-20
NR2F1	-1.5433	1.81E-144	8.52E-142
SAMD8	-1.5405	3.23E-82	4.49E-80
AC005077.14	-1.5364	5.05E-08	3.26E-07
CSPG5	-1.5326	4.52E-28	1.20E-26
RHBDD2	-1.5313	1.39E-163	9.80E-161
HMMR	-1.5245	1.39E-104	3.14E-102
PREB	-1.5212	9.70E-136	4.16E-133
LRRCC1	-1.5168	5.86E-74	6.96E-72
ARHGAP6	-1.5124	8.77E-98	1.73E-95
ARHGAP19	-1.505	1.41E-70	1.58E-68
YPEL1	-1.4981	7.82E-09	5.51E-08
CTD-2636A23.2	-1.4977	1.97E-07	1.18E-06
KCNMA1	-1.4973	2.00E-16	2.79E-15
TFDP1	-1.4968	9.73E-171	8.00E-168
PIGW	-1.4949	2.27E-46	1.26E-44
ITGA6	-1.4927	4.98E-22	9.62E-21
TUBB4A	-1.4843	1.96E-08	1.32E-07
BORA	-1.4796	5.88E-29	1.63E-27
RPL14	-1.4737	4.68E-246	7.69E-243
PPM1N	-1.4696	8.42E-10	6.56E-09
GALNT6	-1.4683	5.15E-08	3.32E-07
RP4-594I10.3	-1.4677	1.46E-32	4.81E-31
RP11-500G10.1	-1.4664	7.32E-10	5.72E-09
AIDA	-1.4656	3.64E-81	4.99E-79
MINPP1	-1.4615	1.62E-72	1.89E-70
ACTG1	-1.4564	0	0
SLC2A4	-1.4491	1.47E-07	9.01E-07
KCNMB4	-1.4487	2.64E-47	1.51E-45
C1RL	-1.4481	2.31E-07	1.37E-06
RLN1	-1.4463	7.02E-07	3.93E-06
TOP1MT	-1.446	3.07E-92	5.32E-90
ARNT	-1.4445	5.18E-138	2.27E-135
RAB8B	-1.4441	5.57E-46	3.05E-44
ZNF512	-1.4419	1.66E-37	6.67E-36
AMACR	-1.4418	2.58E-08	1.72E-07
STEAP1	-1.434	4.97E-95	9.26E-93
PSMB2	-1.424	5.55E-159	3.53E-156
TGM2	-1.4203	2.41E-06	1.26E-05
RHOQ	-1.4185	4.73E-22	9.16E-21
RAB28	-1.4149	2.89E-36	1.09E-34
ZNF213	-1.4135	3.63E-40	1.63E-38
SERPINE2	-1.4086	1.43E-38	5.94E-37

ASB9	-1.4033	2.97E-153	1.61E-150
HTRA2	-1.403	5.45E-46	2.99E-44
KIAA1211	-1.4016	5.89E-35	2.16E-33
VIM	-1.3984	2.66E-17	3.94E-16
RCN3	-1.3962	1.08E-09	8.32E-09
DPYSL2	-1.3927	7.15E-164	5.22E-161
LINC00693	-1.3909	1.97E-06	1.04E-05
C12orf43	-1.3904	6.46E-41	3.00E-39
POLR3G	-1.3864	7.54E-17	1.07E-15
ZNF721	-1.3855	2.22E-70	2.44E-68
ZDBF2	-1.3832	1.54E-82	2.17E-80
CERS4	-1.3822	4.68E-06	2.35E-05
C17orf104	-1.3772	4.24E-17	6.18E-16
LANCL2	-1.3768	1.94E-48	1.15E-46
ASH2L	-1.3726	4.40E-121	1.45E-118
HYOU1	-1.361	1.41E-147	7.13E-145
TNFSF4	-1.3604	4.24E-06	2.14E-05
TEX15	-1.3564	1.82E-23	3.80E-22
SREBF1	-1.3542	1.09E-57	8.48E-56
SSR1	-1.3539	1.37E-131	5.22E-129
PNMA2	-1.3532	6.02E-114	1.72E-111
HNF4A	-1.3514	8.36E-08	5.24E-07
SERINC5	-1.351	3.04E-24	6.58E-23
FAM120C	-1.3456	6.89E-30	2.00E-28
NUDT19	-1.3415	2.21E-68	2.28E-66
PDZRN4	-1.3412	1.77E-05	8.15E-05
YIPF2	-1.3406	5.91E-84	8.63E-82
FAM174B	-1.3388	7.88E-08	4.96E-07
SLC18B1	-1.3381	6.23E-43	3.06E-41
TNFRSF10D	-1.3361	4.48E-64	4.03E-62
TTC7B	-1.3329	1.19E-33	4.14E-32
LMOD1	-1.3329	9.04E-08	5.66E-07
ZNF695	-1.3321	1.74E-20	3.06E-19
C7orf63	-1.3315	4.34E-20	7.50E-19
OPRL1	-1.326	2.59E-05	0.00011621
PCDHB11	-1.3256	5.80E-20	9.94E-19
PTK7	-1.3247	7.90E-06	3.84E-05
KLK3	-1.3215	3.78E-08	2.47E-07
RSPO3	-1.3212	1.14E-05	5.44E-05
ZNF716	-1.319	2.56E-13	2.78E-12
RPL4P4	-1.3176	1.78E-06	9.47E-06
TMEM43	-1.316	7.40E-96	1.39E-93
C2orf16	-1.3148	3.88E-08	2.53E-07
LINC00668	-1.3122	1.84E-05	8.42E-05
PAQR4	-1.3114	6.69E-35	2.44E-33

C1QL4	-1.3109	8.23E-26	1.93E-24
DUT	-1.3079	5.76E-104	1.26E-101
DCAF7	-1.3065	3.01E-170	2.28E-167
CCDC112	-1.3005	1.37E-34	4.94E-33
OSMR	-1.2979	3.02E-14	3.56E-13
KLKP1	-1.2974	2.74E-09	2.03E-08
RP11-337C18.8	-1.2964	8.10E-10	6.31E-09
H6PD	-1.2956	6.71E-96	1.27E-93
TDRD6	-1.2949	1.99E-30	5.96E-29
NES	-1.2914	6.20E-24	1.32E-22
EZR	-1.2909	3.37E-138	1.51E-135
SLC16A4	-1.2905	4.06E-16	5.54E-15
MCAM	-1.2899	2.78E-09	2.06E-08
F3	-1.2878	3.09E-24	6.70E-23
C19orf77	-1.2874	2.22E-06	1.17E-05
PODXL	-1.2873	1.72E-113	4.83E-111
DCAF12	-1.286	3.01E-153	1.61E-150
TP53I11	-1.2857	1.63E-85	2.46E-83
LINC00662	-1.2857	3.42E-26	8.19E-25
DMBX1	-1.283	3.98E-05	0.0001731
RBMXL1	-1.2814	2.28E-41	1.07E-39
RBMX	-1.2804	4.66E-120	1.51E-117
PDIA3P	-1.2763	8.56E-14	9.69E-13
PBX1	-1.2761	6.83E-33	2.29E-31
AC005682.5	-1.2752	1.82E-12	1.82E-11
ZFP37	-1.2727	1.31E-19	2.20E-18
AFAP1	-1.2721	7.26E-26	1.70E-24
MRC2	-1.2718	1.05E-08	7.32E-08
PAK3	-1.2709	1.36E-06	7.36E-06
ZNF729	-1.2705	7.17E-12	6.78E-11
CLMN	-1.2704	3.23E-51	2.04E-49
CA3	-1.2669	2.20E-05	9.93E-05
ARHGEF25	-1.2616	8.18E-19	1.32E-17
AC130352.1	-1.2592	6.23E-08	3.97E-07
DNAJB11	-1.2569	1.03E-131	3.98E-129
AC003665.1	-1.2523	4.15E-15	5.23E-14
COL18A1	-1.2505	1.59E-05	7.40E-05
TMEM108	-1.2503	3.68E-07	2.13E-06
DSE	-1.2457	9.58E-10	7.42E-09
CPED1	-1.2386	2.31E-10	1.91E-09
PRICKLE2	-1.2363	1.33E-08	9.14E-08
POU2AF1	-1.2349	2.02E-05	9.19E-05
EMP2	-1.2348	2.09E-18	3.31E-17
RNF168	-1.2343	1.92E-55	1.37E-53
FAM86C2P	-1.2343	6.25E-15	7.79E-14

FAM105A	-1.2327	2.80E-12	2.75E-11
GDAP1	-1.2316	1.69E-37	6.76E-36
P2RX7	-1.2315	1.10E-06	6.02E-06
ADNP2	-1.2313	2.78E-91	4.73E-89
AC010524.2	-1.2302	7.65E-18	1.17E-16
C1orf51	-1.227	1.63E-104	3.61E-102
GPER	-1.2234	1.17E-05	5.56E-05
RP11-195E2.4	-1.2213	1.26E-11	1.17E-10
SYNE3	-1.221	1.72E-07	1.04E-06
SNTB2	-1.2206	8.42E-62	7.04E-60
ATG5	-1.2197	1.67E-44	8.65E-43
C17orf96	-1.2152	1.18E-86	1.83E-84
PRKAG2	-1.2135	7.80E-37	3.03E-35
SLC9A1	-1.2134	5.75E-77	7.42E-75
SCN1A	-1.2106	9.40E-05	0.00038474
YWHAQ	-1.2087	1.39E-145	6.67E-143
NPPC	-1.207	4.70E-07	2.69E-06
COL3A1	-1.2015	1.40E-07	8.57E-07
DNAJC19P5	-1.2001	0.0001403	0.00055601
RP11-549B18.2	-1.1982	6.51E-06	3.21E-05
KIF21B	-1.1958	1.85E-10	1.54E-09
SLC35F1	-1.1935	6.64E-17	9.52E-16
SH2D5	-1.1935	2.86E-05	0.00012736
RPL14P1	-1.1928	3.15E-08	2.08E-07
SOCS2	-1.1923	0.0001325	0.00052815
RP11-318L16.6	-1.1898	3.84E-07	2.22E-06
PLCB1	-1.1895	4.34E-50	2.65E-48
CACNB3	-1.1884	2.48E-55	1.76E-53
KRCC1	-1.184	3.74E-39	1.60E-37
MCMDC2	-1.1802	2.21E-14	2.64E-13
RP11-458D21.1	-1.178	2.56E-12	2.53E-11
SCFD1	-1.1738	1.13E-64	1.05E-62
NUDT17	-1.1732	5.59E-25	1.26E-23
MGAT4C	-1.1707	1.30E-23	2.74E-22
RP11-81M19.3	-1.1688	5.54E-05	0.00023542
RAD50	-1.1643	6.58E-84	9.47E-82
RCAN3	-1.1629	2.12E-43	1.07E-41
CNTD2	-1.1621	1.95E-09	1.47E-08
AP3S2	-1.1619	1.58E-25	3.66E-24
RPRD1A	-1.1608	1.43E-93	2.58E-91
PAQR8	-1.1602	4.56E-17	6.63E-16
CAMKK1	-1.1591	5.15E-14	5.94E-13
FOLR1	-1.1589	0.00015177	0.00059857
RCC1	-1.1577	6.62E-115	1.98E-112
ITGA2	-1.1565	8.79E-40	3.88E-38

SLC35D1	-1.1551	1.14E-56	8.52E-55
CALM1	-1.155	9.01E-128	3.12E-125
ZNF425	-1.1544	1.69E-13	1.87E-12
EIF3EP1	-1.1539	2.07E-06	1.09E-05
CROCCP4	-1.1524	9.97E-07	5.47E-06
RBBP8	-1.1506	1.27E-52	8.56E-51
SLITRK3	-1.1501	1.20E-14	1.46E-13
BATF2	-1.149	3.03E-05	0.00013446
GATAD1	-1.1489	5.58E-64	4.98E-62
RP11-295M18.	-1.1486	0.00023452	0.00089644
GHR	-1.1453	1.56E-30	4.73E-29
CTC-451A6.4	-1.1451	0.0002368	0.00090443
MAPRE2	-1.1432	1.57E-49	9.52E-48
FKBP14	-1.1407	6.57E-28	1.72E-26
EIF5A2	-1.136	3.10E-28	8.32E-27
PALD1	-1.1358	0.00021017	0.00080976
DDX1	-1.1356	5.12E-89	8.43E-87
MESP2	-1.1333	0.00022706	0.00086927
HS3ST4	-1.1327	7.97E-43	3.87E-41
KIF5B	-1.1317	2.47E-138	1.13E-135
LPHN3	-1.1311	2.20E-31	6.90E-30
NFIB	-1.1298	7.67E-101	1.61E-98
LOX	-1.1273	7.47E-09	5.27E-08
FREM3	-1.1262	1.22E-05	5.79E-05
ING3	-1.1256	9.90E-37	3.83E-35
LIX1L	-1.1242	5.83E-11	5.06E-10
GSAP	-1.1209	1.30E-33	4.50E-32
METTL25	-1.117	4.76E-10	3.81E-09
ABCC6	-1.115	1.20E-11	1.12E-10
MTFR1	-1.1146	6.32E-75	7.61E-73
OPN1SW	-1.1141	9.57E-09	6.69E-08
HYLS1	-1.112	8.87E-14	1.00E-12
ABAT	-1.1109	2.57E-36	9.75E-35
LARP4	-1.1108	1.13E-108	2.73E-106
ANKRD27	-1.1103	3.17E-54	2.21E-52
LRG1	-1.1083	0.00039325	0.0014452
GLYATL2	-1.1068	0.00044624	0.0016221
UBLCP1	-1.1055	4.22E-30	1.25E-28
APLF	-1.1053	2.76E-12	2.72E-11
CDCA7	-1.105	2.87E-34	1.02E-32
TMCC3	-1.1036	3.38E-19	5.56E-18
ZC4H2	-1.1026	1.91E-34	6.84E-33
LRRIQ3	-1.1021	2.15E-05	9.73E-05
TFAP4	-1.1004	9.32E-45	4.90E-43
AL449209.1	-1.0996	2.56E-06	1.33E-05

CPSF2	-1.0979	2.73E-70	3.00E-68
NOL8	-1.0977	9.71E-41	4.47E-39
RNF223	-1.0977	0.0004876	0.0017588
NLGN1	-1.0973	1.97E-46	1.10E-44
BRDT	-1.0955	0.00021453	0.00082546
PAQR3	-1.0949	7.17E-25	1.60E-23
BLZF1	-1.0946	1.79E-51	1.15E-49
HSD17B6	-1.0946	5.03E-05	0.00021529
ELOVL6	-1.0945	2.37E-98	4.73E-96
LTBP4	-1.0913	1.63E-36	6.22E-35
DHRS4-AS1	-1.0897	2.45E-15	3.15E-14
AC073043.2	-1.0886	1.11E-07	6.86E-07
SLC44A2	-1.0872	9.09E-80	1.21E-77
KRAS	-1.0857	2.50E-74	2.99E-72
SLFN13	-1.0855	0.00023936	0.00091366
SERINC2	-1.0849	4.16E-36	1.57E-34
DALRD3	-1.0827	3.01E-51	1.91E-49
ADORA2B	-1.0827	9.98E-09	6.96E-08
RP11-151A6.4	-1.0824	0.00058923	0.0020982
C2orf44	-1.0821	7.38E-27	1.82E-25
ULK4P3	-1.0821	0.00045316	0.0016445
SLC25A30	-1.0801	2.69E-31	8.40E-30
ENTPD8	-1.0801	0.00030603	0.0011467
KLHL1	-1.0801	0.00061192	0.0021711
WDFY2	-1.0791	7.31E-17	1.04E-15
CHST14	-1.0779	2.38E-46	1.32E-44
ATCAY	-1.0778	7.18E-05	0.00029947
CTD-2240H23.2	-1.0767	2.64E-06	1.37E-05
MFAP3	-1.0765	7.89E-57	6.01E-55
MSI1	-1.0763	2.31E-43	1.15E-41
PTPLB	-1.0761	5.98E-87	9.36E-85
ADPGK	-1.0757	5.67E-31	1.74E-29
ZNF467	-1.0743	1.49E-13	1.65E-12
SLC25A1P3	-1.0736	0.00033733	0.001253
WRB	-1.0734	4.24E-44	2.17E-42
DCP2	-1.0733	1.73E-66	1.70E-64
CCDC88A	-1.0718	2.60E-15	3.33E-14
RP4-610C12.4	-1.0717	0.00048431	0.0017476
SLC25A44	-1.0692	2.76E-72	3.19E-70
LINC00634	-1.0689	0.00064451	0.0022766
TAF6	-1.0686	2.97E-49	1.79E-47
CAPG	-1.0685	1.75E-20	3.08E-19
GM2A	-1.0672	7.56E-32	2.42E-30
PKP2	-1.0661	2.26E-23	4.69E-22
PRPF18	-1.065	1.95E-07	1.17E-06

C18orf21	-1.0648	5.43E-16	7.35E-15
GRAMD4	-1.0641	1.32E-31	4.18E-30
SEMA4A	-1.0635	3.13E-109	7.71E-107
COPRS	-1.0633	1.95E-40	8.82E-39
SLC5A5	-1.0633	2.08E-26	5.03E-25
HRH3	-1.063	4.25E-05	0.00018389
PRRC1	-1.0628	6.14E-76	7.72E-74
THRAP3	-1.0622	2.20E-107	5.23E-105
WDR72	-1.062	1.60E-52	1.07E-50
C7orf31	-1.0613	1.14E-06	6.22E-06
CDH24	-1.0591	1.41E-51	9.07E-50
ARL2	-1.0581	1.57E-19	2.63E-18
AC015849.16	-1.0581	1.02E-07	6.36E-07
JPH1	-1.0557	1.43E-43	7.25E-42
RASSF9	-1.0555	1.28E-17	1.95E-16
CAMKV	-1.0555	6.44E-14	7.38E-13
CCP110	-1.0546	2.04E-37	8.15E-36
DTX1	-1.0544	0.00047523	0.0017174
AC015849.15	-1.054	1.33E-06	7.17E-06
CLSPN	-1.0532	4.83E-24	1.04E-22
SNAP25	-1.0502	2.23E-11	2.03E-10
CARNS1	-1.0501	3.58E-14	4.19E-13
BEX4	-1.0493	3.11E-31	9.67E-30
PLAT	-1.049	2.00E-07	1.20E-06
GSE1	-1.0486	3.95E-102	8.48E-100
SECISBP2	-1.0483	1.81E-61	1.49E-59
KLHL18	-1.0475	3.33E-49	1.99E-47
SMKR1	-1.0475	8.99E-22	1.71E-20
ZNF849P	-1.046	0.00043828	0.0015955
HEYL	-1.0449	1.51E-05	7.06E-05
DDX53	-1.0448	2.70E-05	0.00012081
EIF4EBP2	-1.0447	2.26E-119	7.08E-117
AGMAT	-1.0447	2.06E-16	2.86E-15
ATOH8	-1.0433	3.75E-06	1.91E-05
TTC9C	-1.0429	1.24E-14	1.51E-13
TSPAN7	-1.0428	0.00029436	0.0011064
PXMP4	-1.0424	3.45E-28	9.22E-27
RP11-517P14.2	-1.0422	0.00016881	0.00066113
AZGP1P1	-1.042	1.50E-06	8.03E-06
LPAR5	-1.0419	0.00023334	0.0008921
FAM149A	-1.0405	9.18E-13	9.45E-12
RARB	-1.0401	2.97E-08	1.96E-07
HS2ST1	-1.0397	3.89E-31	1.20E-29
CELF5	-1.0374	2.23E-07	1.33E-06
EIF4G2	-1.0372	5.13E-129	1.81E-126

XPOT	-1.0366	3.87E-112	1.05E-109
AC091167.3	-1.0363	0.00080156	0.0027747
SLA2	-1.0358	0.0005979	0.0021248
C5orf56	-1.0354	0.00023874	0.00091167
TPMT	-1.0352	9.48E-49	5.62E-47
FTO-IT1	-1.035	0.0003163	0.0011822
CBWD2	-1.0345	1.18E-29	3.40E-28
SCN9A	-1.0337	3.40E-09	2.50E-08
ULBP3	-1.0337	0.00097695	0.0033338
C19orf57	-1.0322	1.93E-05	8.84E-05
MEP1A	-1.0315	9.93E-22	1.88E-20
ACTL8	-1.0305	0.00027971	0.0010569
TLL1	-1.0278	2.57E-17	3.82E-16
CDH26	-1.0275	5.92E-06	2.94E-05
DLX3	-1.0273	7.18E-05	0.00029947
ADH6	-1.0269	4.33E-05	0.00018725
NOTUM	-1.0265	0.00052304	0.0018788
BOD1	-1.0264	2.01E-41	9.46E-40
KRR1	-1.0221	1.88E-59	1.49E-57
MEN1	-1.0219	2.36E-51	1.50E-49
EHD2	-1.019	8.94E-07	4.93E-06
GDPD1	-1.0187	3.58E-13	3.82E-12
SIAE	-1.017	1.96E-40	8.87E-39
RP11-477D19.2	-1.0168	0.00020562	0.00079381
AL050303.1	-1.0163	0.00032895	0.0012249
RP11-154D6.1	-1.015	1.65E-09	1.25E-08
PITHD1	-1.0141	3.62E-49	2.15E-47
NBR1	-1.0138	1.85E-86	2.85E-84
PYGO1	-1.0136	2.73E-23	5.66E-22
MYLK3	-1.0127	0.0013005	0.0043214
CTC-523E23.1	-1.0121	0.00086449	0.0029789
LPL	-1.0117	7.56E-07	4.21E-06
ZDHHC19	-1.0116	0.0012663	0.0042233
MANF	-1.0115	7.34E-97	1.42E-94
RP4-784A16.5	-1.0112	0.0013171	0.00437
ZMIZ1	-1.0108	9.19E-37	3.56E-35
KB-1507C5.4	-1.0106	0.0013028	0.0043281
HTRA3	-1.0099	0.00020622	0.00079548
LAD1	-1.0097	7.77E-100	1.60E-97
C1orf21	-1.0084	3.48E-26	8.33E-25
RP11-830F9.5	-1.0075	0.0013935	0.0045947
TRIM2	-1.0066	4.67E-11	4.10E-10
KCNAB2	-1.0044	2.39E-16	3.31E-15
TM6SF1	-1.0042	2.74E-05	0.00012253
ADAM7	-1.0042	0.0014453	0.0047472

RP4-657E11.1(	-1.0038	2.38E-07	1.41E-06
FCRLB	-1.0033	0.0010254	0.0034852
FBLN7	-1.0029	2.87E-05	0.00012769
PLA1A	-1.0022	1.35E-17	2.04E-16
NT5DC3	-1.0017	6.75E-39	2.85E-37
PDE1B	-1	0.0012449	0.0041577
C11orf85	-0.99908	0.00040563	0.0014887
MDFIC	-0.99879	1.37E-59	1.10E-57
CHST13	-0.9979	3.62E-05	0.00015826
EXOC6	-0.99758	1.39E-35	5.16E-34
HMGCR	-0.99683	2.97E-84	4.37E-82
CAMK2G	-0.99661	7.95E-32	2.54E-30
AGK	-0.99644	4.67E-33	1.57E-31
TOX4	-0.99561	1.25E-60	1.02E-58
CTD-2366F13.1	-0.99536	4.19E-12	4.06E-11
ELOVL2	-0.99503	7.51E-21	1.35E-19
ALG1L6P	-0.99476	4.77E-07	2.73E-06
PLCH2	-0.99362	3.99E-05	0.00017346
TOP2A	-0.99326	8.57E-92	1.47E-89
ENHO	-0.99186	2.70E-06	1.40E-05
DNAH8	-0.99184	0.00062008	0.0021965
RASSF2	-0.99165	0.0013847	0.0045706
FOXM1	-0.99087	1.76E-62	1.53E-60
ASS1	-0.98886	1.98E-16	2.76E-15
LRRTM4	-0.98881	0.00019191	0.00074347
RADIL	-0.98878	1.14E-06	6.22E-06
FAM49A	-0.98864	0.00048465	0.0017485
KCNT2	-0.98747	0.00081322	0.0028125
CREB3L1	-0.98721	0.0007334	0.0025612
TMEM52	-0.984	1.02E-07	6.36E-07
TAPBPL	-0.98318	2.42E-14	2.88E-13
THG1L	-0.9828	1.98E-08	1.33E-07
PPP2R3A	-0.98206	1.68E-13	1.85E-12
PLA2G4A	-0.98031	7.62E-17	1.08E-15
SRP72	-0.98012	2.03E-75	2.51E-73
MYH15	-0.97927	2.54E-09	1.89E-08
AMOT	-0.97551	1.25E-32	4.13E-31
INPP5A	-0.97521	1.16E-26	2.85E-25
HNMT	-0.97397	5.42E-11	4.72E-10
CEBPZ	-0.97382	1.10E-36	4.24E-35
SYCP3	-0.97313	0.00011034	0.00044514
MED21	-0.97229	3.22E-23	6.62E-22
MED30	-0.97152	1.06E-22	2.12E-21
SLC7A5	-0.97137	1.81E-112	5.02E-110
DAPL1	-0.97061	0.0019269	0.006177

NSUN7	-0.96979	0.00010507	0.00042569
C1orf170	-0.96972	0.0001078	0.00043588
PCSK9	-0.96959	0.00047563	0.0017185
CRISPLD2	-0.96957	8.81E-08	5.52E-07
E2F1	-0.96839	9.77E-40	4.29E-38
TSPAN11	-0.96832	6.71E-21	1.21E-19
NRCAM	-0.96682	3.54E-19	5.80E-18
CCNB3	-0.96659	0.00020159	0.00077856
PCDH18	-0.9664	0.00015166	0.00059825
NCMAP	-0.96548	1.47E-06	7.89E-06
RP11-513I15.6	-0.96463	6.08E-62	5.15E-60
NLRP6	-0.96303	0.00022614	0.0008659
BX842568.2	-0.96162	0.0001345	0.00053484
FAM57B	-0.96159	7.10E-06	3.48E-05
SLC35B2	-0.95782	7.26E-84	1.04E-81
DPY19L3	-0.95772	1.51E-34	5.44E-33
EP400	-0.956	2.71E-37	1.07E-35
EMC4	-0.95504	9.78E-60	7.84E-58
PPP1R13L	-0.95435	1.70E-26	4.12E-25
BRMS1L	-0.95338	2.13E-17	3.19E-16
NUDT21	-0.9524	7.45E-70	8.12E-68
PNO1	-0.95196	8.91E-32	2.84E-30
NUP93	-0.95187	2.05E-97	4.01E-95
CDNF	-0.95	1.49E-06	8.02E-06
ENTPD1	-0.94904	3.84E-28	1.03E-26
ASB11	-0.94882	8.92E-07	4.92E-06
ZNF114	-0.94873	2.17E-07	1.30E-06
RP11-108K14.4	-0.94764	0.0008161	0.0028215
AP001372.2	-0.94678	3.46E-06	1.77E-05
BANK1	-0.94621	0.00028856	0.0010878
AC019097.7	-0.94527	0.00024111	0.00092002
UST	-0.9446	1.37E-11	1.27E-10
TCF3	-0.94306	7.10E-47	4.01E-45
LRPPRC	-0.94259	2.29E-65	2.17E-63
CNTRL	-0.94254	7.61E-36	2.85E-34
VEPH1	-0.94062	2.78E-10	2.28E-09
HN1L	-0.94053	1.02E-75	1.26E-73
ERG	-0.93938	0.0025952	0.0081264
IMPACT	-0.93927	1.11E-45	5.99E-44
BET1	-0.93916	2.22E-23	4.62E-22
PLEKHA2	-0.93864	6.85E-13	7.14E-12
RAP1GAP2	-0.93853	2.75E-61	2.25E-59
CMTM4	-0.93828	2.04E-67	2.02E-65
GCHFR	-0.93775	1.26E-42	6.10E-41
RAB31	-0.93775	1.66E-09	1.26E-08

IFNAR1	-0.9373	1.48E-47	8.46E-46
ZNF83	-0.93686	2.07E-16	2.87E-15
AL133216.1	-0.93625	0.00095155	0.0032533
PAQR5	-0.93623	1.46E-05	6.80E-05
GNG7	-0.93616	5.20E-10	4.14E-09
CCDC74A	-0.93591	1.37E-51	8.88E-50
CPNE5	-0.9357	0.0027675	0.0086114
DMC1	-0.93461	6.86E-08	4.35E-07
MAOB	-0.93372	0.0011854	0.0039764
TFAM	-0.93076	2.66E-39	1.14E-37
HSPA2	-0.93071	3.30E-33	1.12E-31
NYAP1	-0.92932	0.0014659	0.0048117
STK36	-0.92869	1.38E-26	3.36E-25
MPND	-0.92769	2.00E-08	1.35E-07
UPF3A	-0.92568	5.33E-27	1.33E-25
C14orf1	-0.92534	2.56E-46	1.41E-44
TCEAL5	-0.92524	0.0015263	0.0049975
GIPC3	-0.92455	4.77E-09	3.45E-08
CDK18	-0.92398	8.10E-27	2.00E-25
SLC25A43	-0.92157	1.44E-23	3.02E-22
BLVRA	-0.92149	4.31E-20	7.45E-19
AK5	-0.92139	1.90E-12	1.89E-11
LEMD2	-0.92118	5.99E-32	1.94E-30
SYNPO2	-0.92107	8.30E-07	4.60E-06
NOV	-0.91956	6.65E-22	1.27E-20
DCT	-0.91931	0.002097	0.0066812
SENP1	-0.91904	1.45E-38	6.01E-37
C16orf74	-0.91883	0.0013529	0.0044797
PAK2	-0.91851	1.99E-71	2.26E-69
EML1	-0.91711	4.38E-10	3.51E-09
CYP2C8	-0.91524	8.58E-07	4.74E-06
NUDT13	-0.91515	4.80E-07	2.74E-06
ZNF140	-0.91425	8.27E-23	1.67E-21
FRG1B	-0.91362	2.66E-22	5.23E-21
ARHGEF1	-0.91332	8.97E-28	2.33E-26
HAPLN3	-0.91299	1.87E-19	3.11E-18
TLR3	-0.91266	0.00075539	0.0026305
EPHX4	-0.91236	1.25E-11	1.16E-10
CTD-3064H18.1	-0.91219	0.0017172	0.0055668
MPZL1	-0.91192	6.84E-86	1.04E-83
DGUOK	-0.91146	6.53E-21	1.18E-19
CAPS2	-0.91026	1.30E-15	1.71E-14
C1R	-0.91013	0.00036942	0.0013635
PTH1R	-0.90994	0.00089223	0.0030654
HDAC11	-0.9096	2.15E-07	1.29E-06

DLG2	-0.90959	5.64E-07	3.20E-06
C6orf25	-0.90929	0.00088931	0.0030575
IL7	-0.90817	7.86E-06	3.83E-05
CBR3	-0.90794	2.39E-05	0.00010743
ZNF77	-0.90765	2.77E-12	2.72E-11
RFC1	-0.90763	9.89E-51	6.14E-49
ATP2B1	-0.90751	8.80E-80	1.18E-77
CDH10	-0.9075	3.00E-13	3.22E-12
FAM133A	-0.90748	0.0019034	0.0061117
DCTN4	-0.90712	9.22E-42	4.39E-40
AP4E1	-0.90693	3.06E-30	9.11E-29
SALL2	-0.90669	1.27E-12	1.30E-11
PRSS16	-0.90659	1.38E-14	1.68E-13
FAM115A	-0.90645	4.80E-47	2.72E-45
CTD-2540F13.2	-0.90613	0.0040207	0.012066
DAG1	-0.90606	2.21E-90	3.69E-88
TMEM185B	-0.90534	2.83E-18	4.44E-17
RNF215	-0.90447	2.42E-16	3.34E-15
LYPD6	-0.90417	7.99E-05	0.00033018
CTC-1337H24.1	-0.90371	0.00083897	0.0028966
NLK	-0.90226	1.35E-19	2.26E-18
LINC00595	-0.90141	0.0026651	0.008323
CTD-3064H18.4	-0.90122	0.0019151	0.0061431
CKAP2L	-0.9007	8.32E-23	1.68E-21
HERC5	-0.90033	6.50E-08	4.13E-07
RP13-36G14.4	-0.90003	0.00095485	0.0032641
RP11-280F2.2	-0.89961	3.84E-05	0.00016731
FBXO48	-0.89959	6.07E-05	0.0002568
ARHGAP33	-0.89919	2.22E-15	2.86E-14
CYP7A1	-0.89826	0.0021324	0.0067885
JAM2	-0.89781	0.0041188	0.012335
ADAMTS12	-0.89778	0.0013811	0.0045607
APC	-0.89724	4.02E-29	1.13E-27
ULBP1	-0.89715	0.002243	0.0071174
TNFAIP2	-0.89713	1.96E-09	1.47E-08
PLEK2	-0.89663	6.70E-09	4.77E-08
DCHS2	-0.89658	0.0013628	0.0045056
CTD-2126E3.1	-0.89533	0.0029	0.0089927
RP11-254F7.2	-0.89505	8.54E-05	0.00035156
BTN3A2	-0.89477	5.44E-22	1.05E-20
SAMD4B	-0.89442	6.34E-39	2.68E-37
CCDC86	-0.89439	6.79E-34	2.37E-32
MRPL50	-0.8929	3.30E-27	8.37E-26
SMTNL2	-0.89234	0.0044628	0.013261
EHD4	-0.89216	7.50E-32	2.40E-30

LSM12	-0.89159	2.79E-33	9.54E-32
ANKRD32	-0.89136	2.25E-16	3.12E-15
HOXB5	-0.89125	2.64E-05	0.00011817
VPS37D	-0.89057	3.82E-07	2.21E-06
OSBPL8	-0.89056	1.39E-62	1.21E-60
TRIM36	-0.8893	5.31E-65	4.99E-63
LRRC2	-0.88865	1.57E-05	7.31E-05
AKAP12	-0.88862	3.54E-16	4.85E-15
MRO	-0.88836	1.81E-09	1.37E-08
C2orf27A	-0.88755	9.35E-09	6.55E-08
SMARCA5	-0.88683	3.73E-54	2.59E-52
ULK4P2	-0.88623	0.0039607	0.011904
REPS2	-0.88603	0.00034632	0.0012854
CTD-2561J22.2	-0.88553	0.0034437	0.010502
NT5M	-0.88539	2.32E-10	1.92E-09
LAMC3	-0.88538	8.94E-15	1.10E-13
PTPRM	-0.88526	0.0049816	0.014662
DPYD	-0.88345	6.25E-06	3.09E-05
GBP1P1	-0.88311	0.0048377	0.014274
PCDHB12	-0.88262	0.0023325	0.0073623
CTD-2516F10.2	-0.88221	0.0051307	0.015056
CBR4	-0.88181	7.21E-17	1.03E-15
MIS18A	-0.88133	1.46E-24	3.23E-23
ANXA6	-0.88124	3.58E-37	1.41E-35
RP13-379L11.2	-0.88115	0.0051869	0.015205
VWA7	-0.88084	4.94E-16	6.71E-15
CCDC50	-0.8808	2.97E-33	1.01E-31
CCSAP	-0.87969	7.41E-46	4.03E-44
RP11-368I7.4	-0.87924	0.00057893	0.0020626
CHST9	-0.87911	0.00432	0.01288
PLIN2	-0.87889	6.30E-10	4.96E-09
RP11-9G1.3	-0.87848	0.00132	0.0043786
KDM4D	-0.87844	0.0013915	0.0045901
SLC6A19	-0.87795	0.0053368	0.015586
KREMEN1	-0.87698	5.69E-13	5.98E-12
RP11-568K15.1	-0.87641	9.71E-25	2.17E-23
KIAA0754	-0.87636	5.77E-06	2.87E-05
RP11-137H2.4	-0.87636	0.005263	0.015403
CCDC15	-0.87627	6.78E-07	3.80E-06
SLC25A21	-0.87443	1.69E-05	7.80E-05
USP21	-0.87442	1.87E-29	5.34E-28
PALLD	-0.87427	1.70E-15	2.20E-14
GLIPR1	-0.87418	3.39E-06	1.74E-05
CRISP3	-0.87368	0.0052432	0.015354
TMEM39B	-0.87307	1.35E-15	1.76E-14

TNFRSF11B	-0.87225	1.44E-10	1.21E-09
SMC1B	-0.87217	3.77E-14	4.40E-13
BTC	-0.87112	6.07E-14	6.98E-13
DKC1	-0.87093	2.76E-64	2.52E-62
EDN2	-0.87083	3.78E-09	2.77E-08
VN1R83P	-0.87068	0.0057394	0.016649
AC003102.3	-0.87036	5.47E-14	6.32E-13
CNKSR1	-0.87031	2.06E-18	3.26E-17
CCDC88B	-0.86977	7.07E-05	0.00029565
FAM90A1	-0.86969	2.99E-06	1.54E-05
DKK2	-0.86868	0.0052131	0.015275
LIMCH1	-0.8686	3.58E-53	2.43E-51
RASGEF1C	-0.86729	0.0025992	0.0081365
INTS4L1	-0.86727	0.0058945	0.017061
ZNF630	-0.86694	4.35E-07	2.50E-06
ZNF525	-0.86683	4.86E-14	5.62E-13
MMP14	-0.86594	0.0004408	0.0016038
ATAD2	-0.86562	3.04E-75	3.70E-73
URI1	-0.86405	5.50E-39	2.33E-37
ASGR1	-0.8635	0.0011326	0.0038176
NRAS	-0.86333	2.83E-48	1.66E-46
AFAP1L1	-0.86315	7.21E-06	3.53E-05
TSPAN12	-0.8629	0.00010225	0.00041522
NIN	-0.86275	1.88E-32	6.15E-31
TMEM216	-0.86231	1.13E-10	9.58E-10
POU6F2	-0.86048	5.10E-07	2.91E-06
CAST	-0.85992	4.20E-43	2.08E-41
DNTTIP1	-0.85905	2.21E-24	4.82E-23
RGS1	-0.85858	0.0064345	0.018469
MLLT6	-0.85833	1.86E-77	2.41E-75
PRPSAP2	-0.85791	2.40E-28	6.47E-27
STARD13	-0.85755	1.78E-17	2.69E-16
PFKFB4	-0.85746	1.93E-12	1.93E-11
PFDN4	-0.85732	2.69E-17	3.98E-16
BRIX1	-0.85653	3.43E-25	7.79E-24
RP11-517B11.2	-0.85633	0.0058593	0.016969
SCNN1A	-0.85595	3.60E-27	9.07E-26
CTD-2521M24.	-0.85548	0.0065053	0.018651
SEMA3G	-0.85484	5.81E-11	5.04E-10
ODF3L2	-0.85412	0.0063915	0.018367
DGKB	-0.8539	0.0031184	0.0096124
ALDH1A2	-0.85372	0.0035299	0.01074
TP53	-0.85275	2.37E-16	3.29E-15
FAM20B	-0.85238	4.80E-64	4.31E-62
SLC38A4	-0.85172	1.14E-16	1.61E-15

PIGK	-0.85125	2.59E-33	8.87E-32
RNF13	-0.85115	4.31E-25	9.76E-24
PATE2	-0.85077	0.0041722	0.012473
KIF14	-0.85074	2.04E-30	6.10E-29
RHBDL2	-0.84994	0.0055589	0.016173
RGS14	-0.84968	3.15E-07	1.84E-06
ISY1	-0.84966	2.48E-14	2.95E-13
C8orf42	-0.84965	7.08E-14	8.08E-13
SDC3	-0.84936	2.43E-33	8.36E-32
SP6	-0.84852	0.0043565	0.012981
CCDC153	-0.84739	0.0070358	0.02
RP11-458D21.2	-0.84697	1.72E-05	7.94E-05
MAPK9	-0.84674	3.29E-38	1.35E-36
TTPA	-0.84656	0.00072506	0.0025334
GHITM	-0.84644	2.26E-82	3.16E-80
NUPL1	-0.84548	2.29E-67	2.26E-65
14-Sep	-0.84532	0.0013091	0.0043478
RP11-436A20.4	-0.84527	0.0014745	0.0048368
ZNF395	-0.84524	6.95E-25	1.55E-23
BMPR1B	-0.84508	1.37E-10	1.15E-09
PLIN5	-0.84498	6.13E-06	3.04E-05
PCDHGA2	-0.84385	0.0035067	0.010681
PTPRB	-0.8435	1.01E-56	7.64E-55
BAI1	-0.84345	2.88E-11	2.58E-10
3-Sep	-0.84232	1.71E-23	3.57E-22
CDH18	-0.84197	0.0036999	0.01119
RP11-359B12.1	-0.84114	0.0010108	0.0034429
NEFM	-0.84109	0.0032527	0.0099704
MDH1B	-0.84102	0.001837	0.0059152
MST4	-0.84093	2.47E-46	1.37E-44
FAM151A	-0.84075	0.0042162	0.012601
GATAD2A	-0.84056	1.52E-60	1.23E-58
ZNF165	-0.8405	2.05E-06	1.08E-05
CHRNB4	-0.84039	0.00072076	0.0025211
FBXO4	-0.84038	1.51E-10	1.27E-09
CTD-2311B13.7	-0.84038	0.0041223	0.012341
DNAJC22	-0.83937	4.89E-37	1.92E-35
EIF3LP3	-0.83922	0.0067335	0.019227
SLC35A1	-0.83876	3.84E-07	2.22E-06
UTP14C	-0.83851	3.18E-27	8.10E-26
CWF19L2	-0.83774	1.72E-25	3.96E-24
ARHGEF17	-0.83764	8.04E-14	9.13E-13
ANO7	-0.83758	0.00010642	0.000431
ATP6AP1L	-0.83699	2.51E-07	1.49E-06
FAM115B	-0.83483	5.93E-10	4.69E-09

RP11-582J16.5	-0.83475	0.0070078	0.019927
INTS4L2	-0.83402	0.0078369	0.022002
CLIC3	-0.83242	8.31E-07	4.60E-06
XKR9	-0.8323	0.0047403	0.01401
DHX58	-0.83185	5.16E-05	0.00022022
TEAD1	-0.83132	5.26E-26	1.25E-24
TNIK	-0.83125	1.70E-16	2.38E-15
SUSD3	-0.83067	0.0035638	0.010826
ZNF564	-0.82978	0.0079639	0.022308
USP13	-0.82928	4.81E-43	2.37E-41
CISD3	-0.82909	2.45E-39	1.06E-37
MLF1IP	-0.82905	1.28E-23	2.69E-22
PDZD3	-0.82903	4.48E-05	0.00019311
ELK3	-0.82831	3.29E-09	2.42E-08
AKT1S1	-0.82825	1.38E-42	6.69E-41
SPNS2	-0.82822	0.00066229	0.002336
ZNF280A	-0.82734	0.0017537	0.0056726
GINS3	-0.82702	5.04E-24	1.08E-22
PPP1R1B	-0.82676	1.76E-08	1.19E-07
C1orf228	-0.82676	0.0066331	0.018971
CTC-523E23.4	-0.82666	0.0084696	0.023518
EXTL1	-0.82646	0.0085056	0.0236
YPEL2	-0.82629	7.05E-11	6.06E-10
CNGB3	-0.82571	0.0061952	0.01785
INSRR	-0.82548	0.007172	0.020332
TPTEP1	-0.82535	6.25E-30	1.82E-28
RP11-66B24.2	-0.82509	0.0036213	0.010977
LRRC37A4P	-0.82465	1.54E-12	1.55E-11
CD47	-0.82421	6.75E-35	2.46E-33
COMMD10	-0.8242	1.18E-14	1.44E-13
GINS2	-0.82331	1.63E-20	2.86E-19
STC1	-0.82311	0.0080264	0.022454
PTGES3L	-0.82273	0.0065652	0.018804
TIMM8AP1	-0.82259	0.0015311	0.0050116
MYLK	-0.82163	2.66E-14	3.15E-13
KIAA1522	-0.82156	2.63E-29	7.43E-28
CRISPLD1	-0.82137	2.08E-09	1.56E-08
OLFM2	-0.82123	3.45E-07	2.01E-06
CTD-3195I5.1	-0.82012	1.12E-09	8.64E-09
BVES	-0.8195	0.0014551	0.0047772
ZBTB10	-0.81941	2.18E-99	4.44E-97
MYBPC1	-0.81909	5.88E-12	5.62E-11
FRS3	-0.81885	1.24E-07	7.60E-07
MKNK1	-0.81875	1.38E-11	1.28E-10
MED12	-0.81858	1.41E-21	2.65E-20

MEIS3P1	-0.81848	0.00036744	0.0013567
GLP1R	-0.81814	0.00079822	0.0027636
MARK2	-0.81748	7.32E-40	3.24E-38
PCBD1	-0.81745	1.79E-57	1.38E-55
HDAC1	-0.81714	1.91E-79	2.53E-77
ATP2A3	-0.81623	0.0090148	0.024853
BAZ2B	-0.81616	4.89E-17	7.08E-16
RP11-15F12.1	-0.81605	0.0066071	0.01891
CFH	-0.81599	0.0086338	0.023906
CEP57	-0.81577	2.00E-25	4.61E-24
GPM6B	-0.81577	1.63E-11	1.50E-10
PDIA4	-0.81445	5.15E-99	1.04E-96
CLIP2	-0.81427	2.99E-17	4.41E-16
ZNF853	-0.81397	0.0097503	0.026609
NFE2L3	-0.81374	2.55E-14	3.03E-13
LHX2	-0.81321	0.0068435	0.019502
DSCR6	-0.81292	0.0093402	0.025621
LYAR	-0.8125	4.42E-26	1.05E-24
SMARCA1	-0.81243	6.37E-12	6.06E-11
PCDHGC3	-0.81187	6.10E-08	3.90E-07
KCTD5	-0.81142	2.20E-40	9.93E-39
PDIA6	-0.81141	1.40E-67	1.42E-65
GPR126	-0.81029	4.06E-62	3.45E-60
CITED2	-0.80959	7.73E-54	5.33E-52
CTC-756D1.1	-0.80931	0.00668	0.019091
PAX5	-0.80852	1.20E-08	8.28E-08
RP11-498D10.6	-0.80805	0.010296	0.027905
RIMS3	-0.80788	1.67E-08	1.14E-07
CDK19	-0.8075	3.87E-52	2.56E-50
STAG3L4	-0.80704	3.02E-14	3.56E-13
SH3RF1	-0.8059	5.67E-34	1.99E-32
UNG	-0.80509	6.49E-62	5.47E-60
TUBA1A	-0.80506	2.73E-31	8.50E-30
RP11-703I16.1	-0.80501	0.0084833	0.023549
PTPN1	-0.80479	2.89E-33	9.85E-32
RP11-321F8.4	-0.80458	7.03E-05	0.00029388
PHF21A	-0.80433	6.39E-30	1.86E-28
B4GALT7	-0.80412	1.49E-15	1.94E-14
C3orf17	-0.80348	7.82E-28	2.04E-26
LINC00504	-0.80262	0.010525	0.028445
IFI44L	-0.80254	0.0085684	0.023745
PPARD	-0.80194	1.04E-15	1.38E-14
CTD-2334D19.1	-0.80182	0.0069436	0.019758
SYT12	-0.80172	0.0010961	0.0037089
UBE2Q1	-0.80068	1.18E-53	8.06E-52

PTCD2	-0.79983	8.33E-13	8.62E-12
STAM2	-0.79879	3.31E-11	2.95E-10
ABCB4	-0.79856	0.00076401	0.0026573
LGALS4	-0.79849	1.13E-06	6.15E-06
CETN3	-0.79843	7.34E-24	1.56E-22
CLDN1	-0.79837	3.18E-06	1.64E-05
NUBP2	-0.79742	6.51E-27	1.61E-25
GOLGA8H	-0.79718	0.0086336	0.023906
CCDC113	-0.7969	1.73E-16	2.41E-15
PCDHB10	-0.79686	5.00E-06	2.50E-05
NEK1	-0.79636	3.52E-09	2.58E-08
NAA40	-0.79615	5.47E-30	1.61E-28
SCD	-0.79602	1.52E-67	1.54E-65
QSER1	-0.79602	6.43E-32	2.07E-30
EPHA3	-0.79486	3.02E-72	3.47E-70
CGNL1	-0.79471	5.97E-05	0.00025268
DHDH	-0.79462	0.011045	0.029653
SPEG	-0.79444	0.010431	0.028213
N4BP3	-0.79404	1.80E-05	8.29E-05
ZNF559	-0.79373	7.19E-10	5.63E-09
CTD-3203P2.2	-0.79362	0.010132	0.027525
SOD3	-0.79357	0.0010006	0.0034104
FIGF	-0.79309	0.0027461	0.0085518
CHCHD4	-0.79277	6.32E-23	1.29E-21
CCDC23	-0.79257	4.66E-05	0.00020032
SYBU	-0.79241	2.69E-21	4.95E-20
ALG1L7P	-0.79201	0.011609	0.030974
GREB1	-0.79172	8.32E-09	5.84E-08
CDKAL1	-0.79153	4.92E-13	5.20E-12
TLE2	-0.79141	1.90E-11	1.73E-10
MAOA	-0.7912	3.75E-80	5.11E-78
SWT1	-0.79059	1.48E-08	1.01E-07
RP3-462E2.5	-0.79046	0.00021104	0.00081279
HPDL	-0.79037	1.60E-09	1.21E-08
MIXL1	-0.78961	0.0055319	0.016108
STX17	-0.78899	3.13E-15	3.98E-14
CTD-2574D22.2	-0.78897	0.00045667	0.0016554
TMC5	-0.78839	2.69E-08	1.78E-07
RP13-401N8.1	-0.78835	0.0073993	0.020883
PCYOX1L	-0.78831	3.75E-22	7.32E-21
DZIP1L	-0.788	0.0019482	0.0062421
ZNF639	-0.78792	4.18E-28	1.11E-26
GMPR	-0.78754	4.95E-05	0.00021198
CPSF4	-0.78744	1.69E-28	4.61E-27
IL34	-0.78718	0.012428	0.032834

SLFN11	-0.78706	1.64E-10	1.37E-09
PLEKHN1	-0.7868	2.81E-07	1.65E-06
NFATC2	-0.78671	0.01086	0.02924
TXLNB	-0.78591	0.01049	0.028362
PEX26	-0.785	5.62E-40	2.50E-38
WAS	-0.78467	0.0069937	0.019895
FAM216A	-0.78441	3.40E-27	8.61E-26
DHRSX	-0.78441	7.80E-08	4.91E-07
WDFY3-AS2	-0.78373	0.00032986	0.0012278
MUC5B	-0.78367	0.010385	0.028107
UBE2K	-0.7832	2.15E-43	1.08E-41
BBS9	-0.78298	6.86E-10	5.39E-09
AL163953.3	-0.78298	0.012992	0.034115
CTBS	-0.7829	3.93E-12	3.82E-11
TRABD2A	-0.78272	0.0095005	0.026017
RECQL4	-0.78258	1.33E-33	4.60E-32
IGFBP5	-0.78255	0.011439	0.030584
NFATC4	-0.78247	5.06E-07	2.89E-06
LINC00471	-0.78242	0.0013916	0.0045901
IL1RAP	-0.78164	0.00056247	0.0020083
AP1S2	-0.78148	1.03E-11	9.60E-11
SLC24A1	-0.78117	3.54E-12	3.46E-11
ACADL	-0.78092	0.00025738	0.00097774
GFI1	-0.7807	3.61E-08	2.36E-07
KLK13	-0.78057	0.012946	0.034005
RBMY1J	-0.77999	0.0094742	0.025963
MYADM	-0.77994	4.33E-16	5.90E-15
MTMR11	-0.77965	1.61E-14	1.94E-13
CCDC102A	-0.77893	0.00013144	0.00052434
GCNT1	-0.77879	4.35E-28	1.15E-26
FCHO1	-0.77879	6.05E-10	4.77E-09
FOXO4	-0.77818	2.14E-11	1.94E-10
DMRT1	-0.77771	0.012161	0.032255
C1GALT1	-0.77765	3.40E-06	1.74E-05
RP11-95D17.1	-0.77749	0.00010704	0.00043313
TCF19	-0.77735	1.89E-13	2.07E-12
ABI3BP	-0.7773	0.0063968	0.018378
FER1L5	-0.77719	0.01367	0.035663
GALM	-0.77666	6.35E-21	1.14E-19
RP11-16P6.1	-0.77501	0.0013848	0.0045706
FSTL3	-0.77433	1.19E-09	9.15E-09
EPN2	-0.77411	9.73E-16	1.30E-14
UNC5A	-0.77375	0.013952	0.036293
AC132872.2	-0.77275	0.0035267	0.010732
CYGB	-0.7725	0.014231	0.036961

ADAMTSL4	-0.77195	0.00054928	0.0019659
VWA2	-0.77113	1.79E-08	1.22E-07
WIF1	-0.7707	1.32E-05	6.22E-05
STARD6	-0.7707	0.014237	0.036973
GTSE1	-0.77044	4.73E-27	1.18E-25
PLCXD3	-0.76987	0.0073874	0.020853
PROB1	-0.76958	8.90E-08	5.57E-07
FAM46D	-0.7695	0.0095731	0.026173
TMEM74	-0.76889	0.01178	0.031364
IL10RB	-0.76887	9.76E-09	6.82E-08
FNBP1P1	-0.76841	5.38E-06	2.68E-05
FAM161A	-0.76791	2.05E-09	1.54E-08
STXBP1	-0.76773	1.19E-13	1.33E-12
RP11-439C15.4	-0.76756	0.0064304	0.01846
GPRASP1	-0.76745	0.004063	0.012178
AP003733.1	-0.76729	0.012238	0.032413
RSPO4	-0.76708	1.14E-15	1.51E-14
RAB40B	-0.76702	2.13E-18	3.37E-17
RIC8B	-0.76645	4.44E-11	3.91E-10
RP11-342K6.1	-0.76637	3.20E-13	3.43E-12
TBC1D19	-0.76607	5.14E-05	0.00021935
SYCE2	-0.76603	0.0099392	0.027065
SCG2	-0.766	0.0012886	0.0042872
SRSF12	-0.76588	6.35E-05	0.00026724
CABP4	-0.76571	4.41E-05	0.00019021
RP11-211C9.1	-0.76556	0.015151	0.039053
CMIP	-0.76495	1.88E-18	2.99E-17
BIVM	-0.76488	2.80E-13	3.03E-12
PLA2G16	-0.7645	1.76E-44	9.10E-43
TMEM198	-0.76439	8.45E-07	4.67E-06
ANKRD18EP	-0.7643	8.61E-13	8.89E-12
ARHGEF37	-0.76352	4.40E-43	2.17E-41
RP11-439C15.2	-0.76319	0.012089	0.032109
PRKD3	-0.76272	6.26E-15	7.79E-14
SCUBE2	-0.76229	2.02E-13	2.22E-12
ZNF107	-0.76227	7.86E-27	1.94E-25
KLHL2	-0.76217	2.92E-17	4.31E-16
DBP	-0.76211	1.02E-06	5.59E-06
C8orf76	-0.76196	0.0055909	0.016256
SH3D19	-0.76172	9.99E-31	3.04E-29
ZCWPW2	-0.76155	0.0058484	0.016945
PCDHA1	-0.76135	0.00011816	0.00047465
RP11-617F23.1	-0.7608	0.0080184	0.022435
SGK3	-0.76064	2.81E-06	1.45E-05
ABLIM3	-0.76064	0.0047576	0.014051

SHD	-0.76022	0.00049231	0.0017748
FAM76A	-0.7599	5.88E-10	4.65E-09
VSIG10L	-0.7599	8.80E-10	6.83E-09
GCA	-0.75988	9.64E-16	1.29E-14
ZNF804A	-0.75987	0.015786	0.040477
NET1	-0.75985	6.26E-55	4.41E-53
GPRIN1	-0.75957	7.39E-17	1.05E-15
SLC7A14	-0.75954	0.0097168	0.026525
MYB	-0.75952	4.83E-06	2.42E-05
GCNT2	-0.75951	0.011246	0.030138
ALDH18A1	-0.75933	9.70E-63	8.58E-61
BOK	-0.75914	1.38E-16	1.94E-15
PGM1	-0.75896	4.10E-16	5.59E-15
CCDC69	-0.75867	0.0085268	0.023656
RP11-1267H10	-0.7579	0.0053782	0.015691
ARHGEF40	-0.75776	1.87E-08	1.26E-07
PLA2G4D	-0.75765	0.0024871	0.0078167
AC005330.2	-0.75737	0.016137	0.041292
STX6	-0.7568	1.19E-36	4.57E-35
TCF7L1	-0.75671	0.0090574	0.024956
CD36	-0.75647	1.45E-08	9.92E-08
LMO3	-0.75621	0.012675	0.033379
ACRBP	-0.75597	1.59E-05	7.37E-05
TMOD2	-0.75593	2.58E-15	3.30E-14
UNC119B	-0.75588	2.62E-64	2.41E-62
TBX6	-0.75561	0.00044348	0.0016125
NOB1	-0.75522	4.90E-28	1.30E-26
ATP5C1	-0.75511	1.81E-40	8.28E-39
ZNF43	-0.75479	3.53E-34	1.24E-32
G3BP2	-0.75472	2.25E-51	1.44E-49
DUSP19	-0.75456	0.00010845	0.00043833
KCNC2	-0.754	1.76E-05	8.13E-05
MTMR6	-0.7524	1.00E-26	2.46E-25
RP11-220I1.1	-0.75232	3.02E-29	8.49E-28
RP11-690D19.3	-0.7521	0.00056098	0.0020038
LINC00341	-0.75155	0.011307	0.030268
PRSS50	-0.75136	1.81E-10	1.51E-09
SMAD9	-0.75132	5.91E-30	1.73E-28
SCGN	-0.75122	0.0072828	0.020605
RP11-412D9.4	-0.75108	0.010362	0.028058
PCDHAC1	-0.75064	0.0007723	0.0026828
RP11-313J2.1	-0.75006	1.92E-06	1.02E-05
DSCC1	-0.74971	3.88E-22	7.56E-21
ATP12A	-0.74955	0.0015485	0.0050644
PHACTR3	-0.74921	0.013938	0.036264

SHMT1	-0.74892	1.08E-32	3.60E-31
CASP3	-0.7477	2.95E-18	4.63E-17
C16orf62	-0.74756	8.34E-08	5.24E-07
PACS1	-0.74729	1.32E-25	3.08E-24
ARSD	-0.74607	6.88E-26	1.62E-24
C9orf142	-0.74585	3.52E-22	6.89E-21
ZNF358	-0.74555	2.64E-06	1.37E-05
MRFAP1L1	-0.74541	1.78E-25	4.11E-24
ORM2	-0.74541	0.017931	0.04537
RHCE	-0.74533	0.015177	0.039109
CWC22	-0.74515	2.54E-17	3.78E-16
RP11-540A21.2	-0.7449	0.012592	0.033214
ZNF606	-0.74473	4.33E-07	2.49E-06
SLC2A1	-0.74467	2.70E-27	6.90E-26
ALPPL2	-0.74449	0.015384	0.039556
USB1	-0.74446	3.08E-23	6.36E-22
JRKL	-0.74425	4.04E-05	0.00017562
SLC1A3	-0.74418	1.92E-16	2.68E-15
RP11-541N10.3	-0.7437	0.00021805	0.00083769
SERPINH1	-0.74347	3.56E-20	6.18E-19
SRCAP	-0.74339	3.21E-34	1.14E-32
KCTD18	-0.74314	6.73E-15	8.37E-14
ABCA2	-0.74265	9.91E-29	2.73E-27
CLCNKB	-0.74261	0.012946	0.034005
LINC00310	-0.74173	0.0046307	0.013707
SAPCD2	-0.74048	4.27E-21	7.77E-20
STRIP2	-0.73991	0.00010919	0.00044112
SLC5A12	-0.73979	0.015604	0.040048
FBXO43	-0.73906	0.0082452	0.022994
LANCL1	-0.73862	6.26E-43	3.06E-41
LRRC37A6P	-0.7381	0.016266	0.041606
TBC1D23	-0.73802	2.49E-12	2.46E-11
NMT2	-0.73779	2.69E-08	1.78E-07
RBMY1A1	-0.73745	0.0021951	0.0069766
ZNF541	-0.73736	0.0091768	0.025229
IL20RA	-0.73717	0.0025937	0.0081232
EEF1DP3	-0.73671	0.011767	0.031332
USP40	-0.73664	1.19E-32	3.93E-31
ZNF189	-0.73648	9.52E-14	1.08E-12
TRAPPC6B	-0.73636	4.68E-18	7.30E-17
AC026202.3	-0.73581	0.019552	0.048867
GPR37	-0.73534	1.09E-20	1.94E-19
HYDIN	-0.73519	0.0013952	0.0045989
SLC47A2	-0.73516	1.58E-05	7.34E-05
RP11-211G3.2	-0.73516	0.018724	0.047099

SGCB	-0.73411	1.21E-29	3.48E-28
CTAGE5	-0.73389	0.014139	0.036747
RP5-940J5.3	-0.73383	0.019256	0.048252
TMC6	-0.73344	2.78E-11	2.50E-10
TMEM194A	-0.73339	9.99E-38	4.05E-36
PLEKHG2	-0.73335	5.18E-11	4.52E-10
GPR116	-0.73319	0.002999	0.0092674
IGDCC3	-0.73307	0.0067641	0.019298
MITD1	-0.73275	1.23E-09	9.42E-09
PHF15	-0.73271	7.49E-31	2.29E-29
SLC3A2	-0.7327	1.19E-56	8.88E-55
CD248	-0.73267	0.0066824	0.019095
ARL6IP6	-0.73256	3.83E-09	2.79E-08
MDC1	-0.732	2.60E-39	1.12E-37
WDHD1	-0.7318	1.32E-19	2.21E-18
OVGP1	-0.73133	1.39E-05	6.53E-05
NDUFAF7	-0.73122	9.95E-11	8.48E-10
PLLP	-0.73112	0.0057546	0.016686
ACOT11	-0.73027	9.98E-05	0.00040632
CTC-455F18.3	-0.73021	0.019125	0.047978
KCNK17	-0.73001	0.01852	0.046639
MICAL2	-0.72956	9.78E-16	1.30E-14
DNAJB12	-0.72895	2.93E-26	7.04E-25
THRA	-0.72866	2.49E-15	3.20E-14
C15orf38	-0.72839	1.12E-19	1.89E-18
BMX	-0.72804	1.72E-07	1.04E-06
LIN9	-0.72755	3.02E-21	5.55E-20
CPQ	-0.72745	9.12E-05	0.00037375
RP11-977B10.[illegible]	-0.7272	0.015437	0.039655
FAXDC2	-0.72705	1.53E-28	4.19E-27
TMCO6	-0.72639	5.56E-10	4.41E-09
RP11-497H16.[illegible]	-0.72568	0.010982	0.029518
PPM1M	-0.72542	1.84E-11	1.68E-10
DDX31	-0.72539	7.79E-19	1.26E-17
CALU	-0.725	1.79E-44	9.24E-43
UQCC	-0.72496	1.37E-28	3.74E-27
NECAB1	-0.72468	0.01172	0.031221
FAM117B	-0.72459	6.52E-25	1.46E-23
NIPAL3	-0.72431	1.34E-41	6.35E-40
MIS18BP1	-0.7243	1.92E-20	3.37E-19
GALNT7	-0.72384	1.47E-23	3.10E-22
TRMT1L	-0.72383	3.86E-22	7.53E-21
RNF2P1	-0.72353	0.012608	0.033249
SOD2	-0.72327	1.51E-58	1.18E-56
GCK	-0.72313	5.68E-08	3.65E-07

PNPLA8	-0.72298	6.68E-17	9.58E-16
LPCAT4	-0.72281	1.02E-11	9.50E-11
FAM65A	-0.72205	7.48E-11	6.42E-10
UPF2	-0.72183	6.69E-29	1.85E-27
RP11-564C4.6	-0.72167	2.65E-06	1.38E-05
PIF1	-0.72149	5.84E-06	2.90E-05
ANKRD20A17P	-0.72149	0.011908	0.031678
CFC1	-0.72126	4.22E-13	4.47E-12
GTF2H2B	-0.72083	9.04E-07	4.98E-06
GRAMD1C	-0.71968	1.33E-08	9.14E-08
PKNOX1	-0.71942	1.45E-15	1.89E-14
FAM198A	-0.71929	0.00066528	0.0023445
ZNF385B	-0.71863	5.90E-24	1.26E-22
RASA4	-0.71843	6.81E-07	3.82E-06
ASAH2	-0.71815	0.0017617	0.0056955
COLEC12	-0.71766	1.12E-10	9.45E-10
SBK1	-0.7172	4.92E-10	3.93E-09
DZIP3	-0.71655	8.44E-20	1.44E-18
PCDHA10	-0.71634	8.20E-07	4.55E-06
CTD-2314B22.3	-0.71631	1.23E-16	1.74E-15
ZNF268	-0.71615	3.03E-15	3.87E-14
AC093642.3	-0.71609	0.0090973	0.025049
LYSMD1	-0.716	4.23E-15	5.31E-14
MTTP	-0.7159	0.0199	0.049627
RP11-163N6.2	-0.71531	0.0016712	0.005436
TSPAN31	-0.71492	1.81E-21	3.37E-20
WT1	-0.71356	3.07E-09	2.27E-08
SCAMP4	-0.71339	8.85E-18	1.35E-16
BRPF3	-0.71301	6.45E-34	2.26E-32
ACSF2	-0.71298	2.12E-08	1.43E-07
BOLL	-0.71273	0.00012993	0.00051873
PDCD6IP	-0.71243	1.76E-48	1.04E-46
ZNF300	-0.7124	3.78E-08	2.47E-07
SMAD4	-0.71217	1.12E-23	2.37E-22
BHLHA15	-0.71201	0.00046991	0.0016988
ANKRD24	-0.71188	1.12E-12	1.14E-11
TTBK2	-0.71186	1.53E-08	1.05E-07
TINCR	-0.71173	2.15E-08	1.44E-07
MDK	-0.71158	2.91E-50	1.79E-48
FAM114A2	-0.71081	7.38E-14	8.40E-13
RP11-13N13.2	-0.71069	0.0086902	0.024042
ZFYVE28	-0.7102	4.28E-05	0.00018484
VTI1B	-0.70947	2.00E-17	2.99E-16
SPOCD1	-0.70946	0.019709	0.04922
CSNK1G3	-0.70943	3.20E-23	6.58E-22

CD99L2	-0.70899	3.44E-20	5.97E-19
CRIM1	-0.70844	1.79E-29	5.11E-28
FSCN1	-0.70832	1.46E-06	7.84E-06
C3orf67	-0.7077	9.31E-05	0.00038115
NBPF2P	-0.70713	0.0024121	0.0076016
TP53BP2	-0.7071	1.44E-24	3.19E-23
PTPRZ1	-0.70709	2.30E-06	1.20E-05
RPH3AL	-0.70704	0.019166	0.048057
ATHL1	-0.7063	1.04E-06	5.72E-06
RWDD1	-0.70625	4.80E-19	7.85E-18
RP11-142L1.1	-0.70616	0.0082275	0.022952
SLCO2A1	-0.70585	0.0087754	0.02425
ARMC4	-0.7055	6.48E-05	0.00027243
MLEC	-0.70548	3.34E-49	1.99E-47
PIK3AP1	-0.70538	0.00064884	0.0022906
CTD-3092A11.2	-0.7051	1.43E-06	7.72E-06
JAKMIP2	-0.70509	2.27E-08	1.52E-07
TIFA	-0.70472	4.74E-09	3.43E-08
HOXA3	-0.70439	0.00013698	0.00054424
TTPAL	-0.70438	3.06E-16	4.21E-15
STARD4	-0.70406	1.24E-25	2.88E-24
VAMP4	-0.70358	4.87E-14	5.63E-13
EPHB2	-0.70352	3.39E-07	1.97E-06
NAT14	-0.70268	5.19E-10	4.14E-09
DCP1A	-0.70254	5.83E-47	3.30E-45
RP11-62J1.3	-0.70246	2.57E-06	1.34E-05
KIF18A	-0.7022	3.60E-12	3.51E-11
SLC1A1	-0.70211	1.15E-05	5.45E-05
TBC1D8B	-0.70154	4.21E-07	2.42E-06
ZNF384	-0.70151	8.32E-28	2.17E-26
KIAA1191	-0.7015	3.85E-39	1.64E-37
VPS37A	-0.70092	2.94E-29	8.28E-28
MT3	-0.70077	0.0039384	0.011846
FMNL1	-0.70054	4.26E-05	0.00018427
MATN1-AS1	-0.69938	0.01319	0.034568
CTD-3105H18.1	-0.69904	0.0081811	0.022835
RP11-705C15.2	-0.69843	0.0030959	0.0095506
MFSD6L	-0.69809	0.0012544	0.0041864
HOGA1	-0.69808	5.57E-05	0.00023668
HECA	-0.69773	5.94E-12	5.67E-11
IKZF5	-0.69731	2.65E-14	3.14E-13
GATA2	-0.6973	9.12E-41	4.20E-39
RNF122	-0.6971	1.65E-05	7.64E-05
PTMAP2	-0.69691	9.14E-10	7.10E-09
RP11-1008C21.	-0.69672	5.07E-05	0.00021672

WNT8B	-0.69669	2.90E-05	0.00012913
ATP11C	-0.69655	5.79E-17	8.35E-16
PPP2R2B	-0.69651	8.08E-07	4.48E-06
IGFBP1	-0.69613	0.017956	0.045409
GTF2H2C	-0.69596	1.10E-09	8.45E-09
NID1	-0.69567	2.57E-05	0.00011511
PCDH17	-0.69551	0.0050604	0.014865
FKBP10	-0.69538	3.97E-43	1.97E-41
RP11-465B22.3	-0.69527	0.001346	0.0044604
PPP1R14D	-0.69491	0.011615	0.030985
FERMT1	-0.69437	3.63E-26	8.68E-25
GTF2H2	-0.69428	4.45E-12	4.31E-11
IGF1R	-0.694	0.0010078	0.0034339
RP11-512M8.3	-0.69398	5.74E-08	3.68E-07
RP11-149A7.2	-0.6934	0.013426	0.03513
FBXO2	-0.69329	0.0011562	0.0038878
AC097500.2	-0.69302	0.01906	0.047829
ACAD9	-0.69263	9.54E-29	2.63E-27
ZSCAN30	-0.69228	1.32E-17	2.00E-16
PPP1R3E	-0.69135	5.91E-05	0.00025043
TTC3P1	-0.69121	9.84E-05	0.00040093
ZNF354C	-0.68967	0.00072295	0.0025278
DPAGT1	-0.68962	1.05E-18	1.68E-17
POPDC3	-0.68931	0.007382	0.020844
AC093323.3	-0.68898	5.03E-29	1.40E-27
CKAP2	-0.68842	2.50E-27	6.42E-26
ZNF688	-0.68765	7.36E-06	3.59E-05
TNFAIP8L1	-0.6876	8.25E-08	5.18E-07
FAM98A	-0.68655	4.44E-27	1.12E-25
RP11-3N2.1	-0.68652	0.00159	0.0051924
GK5	-0.6864	8.90E-18	1.36E-16
LRRC34	-0.6863	0.011679	0.031122
RP11-329L6.1	-0.68592	0.0052917	0.01548
DOCK8	-0.68566	0.00030083	0.0011287
INIP	-0.68554	1.39E-20	2.45E-19
CTDSPL	-0.68542	8.77E-51	5.46E-49
DAXX	-0.68514	2.68E-27	6.85E-26
KATNAL2	-0.68492	7.28E-12	6.88E-11
DARS2	-0.68432	5.66E-48	3.28E-46
PTGDS	-0.68345	0.0027059	0.0084424
MRPS36	-0.68296	2.66E-13	2.87E-12
FICD	-0.6827	5.77E-08	3.70E-07
ABCB10	-0.68258	5.51E-34	1.93E-32
RDM1	-0.68226	0.0001402	0.0005558
COL6A1	-0.68186	4.27E-05	0.00018484

ZNF429	-0.68166	1.78E-11	1.63E-10
AC018737.1	-0.68155	0.0014102	0.004643
ACTR1B	-0.68135	1.44E-31	4.54E-30
ACLY	-0.6813	1.68E-64	1.56E-62
FPGT	-0.68104	5.48E-07	3.11E-06
SHF	-0.67936	0.0045	0.01336
NRXN1	-0.6793	3.22E-07	1.88E-06
DBC1	-0.67928	0.0019479	0.0062421
ZBTB2	-0.67917	5.65E-10	4.48E-09
AP000696.2	-0.67865	0.010008	0.027234
TSNARE1	-0.67835	1.72E-07	1.04E-06
RP3-430N8.8	-0.67788	0.012737	0.033519
PCDHAC2	-0.67753	1.27E-05	5.99E-05
THBS4	-0.67745	0.015915	0.04077
CEP19	-0.67744	0.00049864	0.0017957
PDGFRL	-0.6773	0.00033555	0.0012475
MYEOV	-0.67728	1.52E-07	9.26E-07
SCG3	-0.67724	6.34E-17	9.12E-16
UBTD2	-0.67719	8.24E-14	9.35E-13
SRBD1	-0.67664	6.20E-09	4.43E-08
KBTBD11	-0.67641	1.01E-22	2.03E-21
WDSUB1	-0.67635	0.0013359	0.0044277
C12orf56	-0.67634	0.00043956	0.0015999
CTD-3014M21.	-0.67623	0.0064473	0.018501
MYO15A	-0.67612	0.014721	0.038053
F8	-0.67567	1.93E-05	8.84E-05
EXOSC8	-0.67559	9.67E-15	1.19E-13
NRN1	-0.67554	8.26E-14	9.37E-13
MTBP	-0.67549	1.22E-11	1.14E-10
C12orf65	-0.67545	8.58E-19	1.38E-17
GRID2IP	-0.67541	0.016037	0.041063
CCT8P1	-0.67516	0.0011484	0.0038651
CTC-429P9.1	-0.67445	0.0040014	0.01201
SOSTDC1	-0.67405	0.0043689	0.013013
ZNF99	-0.67384	0.013281	0.034787
AC093724.2	-0.67381	0.00014041	0.00055633
SCN4B	-0.67334	0.0049889	0.014681
GJC1	-0.67311	6.29E-08	4.00E-07
CHRNB1	-0.67287	3.35E-13	3.58E-12
SEC14L1P1	-0.6728	0.00025675	0.0009757
BCL2	-0.67258	1.08E-07	6.66E-07
ZNF501	-0.67253	1.30E-05	6.12E-05
IL27RA	-0.67245	0.019999	0.049817
CHERP	-0.67238	3.57E-19	5.85E-18
CYP2D7P1	-0.67226	2.09E-07	1.25E-06

KLK4	-0.67225	6.33E-43	3.09E-41
RP11-48B3.3	-0.67174	3.93E-06	1.99E-05
LRRC29	-0.67117	0.013487	0.035261
LINC00511	-0.67044	2.69E-07	1.59E-06
NEURL1B	-0.6702	1.71E-21	3.20E-20
TIGD7	-0.6698	0.0099003	0.02697
SYCP2	-0.66973	0.013309	0.034846
RP11-253E3.3	-0.6696	1.55E-11	1.43E-10
ABCC11	-0.66899	0.0070079	0.019927
CAPN12	-0.66864	0.011979	0.031855
PABPC4L	-0.66863	8.73E-05	0.00035826
ANO6	-0.66757	3.58E-33	1.21E-31
METAP1D	-0.6674	1.71E-06	9.10E-06
ADIPOR1	-0.66684	2.23E-51	1.43E-49
DCAKD	-0.66655	4.22E-21	7.68E-20
PLA2G7	-0.66646	1.35E-24	2.99E-23
STAT5A	-0.66607	7.27E-10	5.69E-09
NDUFS6	-0.66603	9.71E-30	2.80E-28
FBXL17	-0.6656	1.59E-20	2.80E-19
MME	-0.66546	2.69E-29	7.59E-28
TNFRSF10C	-0.66523	0.0044289	0.013175
THOC6	-0.66506	7.46E-15	9.24E-14
DRD5P2	-0.66488	0.019476	0.048711
GJB1	-0.66395	3.09E-09	2.28E-08
NEIL2	-0.66394	1.45E-15	1.89E-14
NPHP3	-0.66389	1.85E-08	1.25E-07
CTBP1-AS1	-0.66366	3.19E-17	4.69E-16
IMPA2	-0.66299	5.10E-13	5.38E-12
RP11-393B14.1	-0.66274	0.00013758	0.0005462
ARV1	-0.66266	1.05E-10	8.94E-10
ROBO3	-0.66236	0.011381	0.030444
KLF15	-0.66229	2.02E-13	2.22E-12
AC005481.5	-0.66192	0.019933	0.049692
TUBE1	-0.66177	4.08E-10	3.29E-09
RP11-705C15.3	-0.6617	0.011744	0.031275
EBPL	-0.66148	1.50E-22	2.98E-21
CD109	-0.66136	1.67E-06	8.90E-06
MSX1	-0.66121	0.014623	0.03783
NINL	-0.66106	4.07E-10	3.28E-09
ZNF365	-0.66092	0.0021759	0.006918
BTN1A1	-0.65997	0.0089344	0.024645
ITFG2	-0.6597	5.78E-11	5.02E-10
FAM192A	-0.65969	6.03E-28	1.59E-26
NOX5	-0.65933	6.55E-05	0.00027488
CRYBG3	-0.65903	3.32E-05	0.00014596

EIF2S2	-0.65869	9.36E-40	4.12E-38
ATP6V0E2	-0.65851	1.66E-34	5.94E-33
SAYSD1	-0.65847	1.20E-12	1.22E-11
RP11-710C12.1	-0.65722	0.0074967	0.021125
HSD17B7	-0.65692	2.05E-19	3.40E-18
PTH2	-0.65679	0.018748	0.047147
IL17RD	-0.65575	4.01E-20	6.95E-19
FAM211B	-0.65542	7.35E-05	0.00030566
CCDC12	-0.65537	2.36E-14	2.81E-13
UGT3A2	-0.65507	0.0062685	0.018037
MPP2	-0.655	1.14E-06	6.24E-06
CTTNBP2NL	-0.65411	3.65E-09	2.67E-08
ADAMTS13	-0.65336	5.26E-09	3.78E-08
AC005682.6	-0.65317	0.0040663	0.012184
PYROXD2	-0.65313	8.13E-08	5.11E-07
PC	-0.65295	7.18E-17	1.03E-15
BAIAP3	-0.65278	9.30E-07	5.12E-06
CTD-3074O7.1[illegible]	-0.6522	1.18E-06	6.43E-06
MTMR7	-0.65192	0.0040317	0.012095
DSN1	-0.65181	8.27E-14	9.38E-13
NMNAT1	-0.65125	5.77E-10	4.56E-09
FAM83H	-0.65115	2.78E-24	6.04E-23
LUZP2	-0.65114	8.71E-05	0.00035778
EGFR	-0.65102	1.73E-16	2.41E-15
KANK1	-0.65099	2.86E-30	8.54E-29
GRIK5	-0.65099	0.0036689	0.011105
CRLF1	-0.65086	0.0082322	0.022962
TNC	-0.65075	1.98E-26	4.79E-25
SRRM4	-0.65069	5.42E-26	1.29E-24
ZNF28	-0.64968	4.87E-15	6.11E-14
KLF12	-0.64899	0.0004622	0.001674
WNT10B	-0.64884	4.38E-15	5.50E-14
PRTFDC1	-0.64876	1.10E-05	5.24E-05
RIF1	-0.64828	4.43E-19	7.24E-18
KLHL30	-0.6482	3.39E-05	0.00014902
KCND2	-0.6482	0.0059626	0.01724
ZNF844	-0.64816	1.10E-13	1.23E-12
AC012512.1	-0.64782	0.013556	0.035408
PIGF	-0.64737	0.00075748	0.0026369
PLEKHA5	-0.64721	2.49E-15	3.20E-14
STAT3	-0.64685	5.28E-31	1.62E-29
FN1	-0.64683	1.22E-37	4.91E-36
DPPA5	-0.64658	0.010234	0.027753
CCDC88C	-0.64631	4.14E-10	3.33E-09
EPOR	-0.64631	3.02E-05	0.00013386

CTSL2	-0.64628	0.0018292	0.0058916
NRM	-0.64586	3.66E-12	3.56E-11
LPP	-0.64544	7.71E-22	1.47E-20
KCNT1	-0.64516	0.0009461	0.003237
ZNF568	-0.64365	2.66E-07	1.57E-06
C15orf52	-0.64342	0.010948	0.029443
ZNF229	-0.64341	0.0012853	0.0042779
TRIP12	-0.64314	1.24E-30	3.77E-29
HCG15	-0.6431	0.0045179	0.013411
OGFRL1	-0.64271	6.70E-13	6.99E-12
HMGCL	-0.64247	2.71E-26	6.52E-25
AIM1L	-0.64235	0.017467	0.044298
PCYT1B	-0.64214	0.017391	0.044145
CTD-2382E5.3	-0.64196	0.011332	0.030326
PDZK1IP1	-0.64178	6.45E-07	3.63E-06
SPOCK3	-0.64168	1.00E-05	4.80E-05
CHRM2	-0.64166	0.0141	0.036655
MAT1A	-0.64119	0.0020394	0.0065123
DCAF4	-0.64085	2.28E-06	1.20E-05
OGG1	-0.64061	4.17E-12	4.05E-11
PDE9A	-0.63987	4.33E-10	3.47E-09
M6PR	-0.63943	6.72E-22	1.28E-20
G2E3	-0.63934	1.31E-12	1.33E-11
AP001816.1	-0.63889	2.50E-12	2.47E-11
BDP1	-0.63867	6.24E-26	1.48E-24
ITGB5	-0.6381	7.83E-44	4.00E-42
C1orf122	-0.63764	1.04E-15	1.38E-14
ANAPC7	-0.63724	6.37E-24	1.36E-22
CANX	-0.63708	1.69E-43	8.55E-42
MRPS10	-0.63696	2.68E-19	4.42E-18
FER	-0.6365	2.96E-12	2.90E-11
TMEM136	-0.6364	3.09E-05	0.00013663
AC004540.5	-0.63633	0.0083303	0.023183
ZMYND8	-0.636	8.03E-18	1.23E-16
GRM3	-0.63562	0.0031568	0.0097172
RARG	-0.63501	2.60E-22	5.12E-21
TASP1	-0.63501	6.32E-08	4.02E-07
STX12	-0.63491	1.25E-23	2.65E-22
AGA	-0.63476	2.69E-06	1.40E-05
CCNG2	-0.63397	1.47E-21	2.77E-20
STEAP2	-0.63383	1.92E-30	5.77E-29
CBWD1	-0.63357	7.40E-22	1.41E-20
RAB9B	-0.63327	0.00081088	0.002805
CDH12	-0.63312	1.67E-13	1.84E-12
VASN	-0.63312	0.018832	0.047341

ZNF485	-0.63286	1.65E-05	7.65E-05
MPP3	-0.63253	4.44E-07	2.55E-06
HNRNPK	-0.63118	5.86E-52	3.83E-50
CNRIP1	-0.63083	0.018881	0.047443
SARDH	-0.63054	4.78E-06	2.40E-05
TTLL7	-0.63028	1.00E-07	6.24E-07
SMC3	-0.63008	1.87E-25	4.32E-24
C6orf170	-0.62948	7.29E-06	3.57E-05
PPM1J	-0.62899	0.0023262	0.007346
MAP3K14-AS1	-0.62895	0.0042886	0.012796
APAF1	-0.62885	2.87E-14	3.40E-13
HOMER2	-0.62881	5.81E-24	1.25E-22
RNF214	-0.62875	3.29E-09	2.42E-08
DHX8	-0.62864	5.69E-27	1.41E-25
KB-1507C5.2	-0.62812	0.001109	0.003748
ELFN1	-0.62807	6.29E-06	3.11E-05
SPDYC	-0.62796	0.015801	0.040505
HEATR3	-0.62712	6.39E-17	9.18E-16
TBX20	-0.62712	0.0091766	0.025229
CCDC122	-0.6269	0.00059397	0.0021128
MYRIP	-0.62686	3.51E-15	4.44E-14
LRRC48	-0.62682	0.00042274	0.0015466
DDIT4	-0.6268	3.81E-10	3.08E-09
COQ7	-0.6261	4.96E-18	7.72E-17
MITF	-0.62513	0.0017343	0.0056145
ZNF836	-0.62506	0.0044536	0.013238
SBNO2	-0.62444	1.12E-19	1.90E-18
TMPO	-0.62415	2.90E-30	8.64E-29
FUT2	-0.62372	0.0076141	0.021431
RP11-649A18.7	-0.62331	0.019414	0.048606
VGF	-0.62309	7.74E-07	4.31E-06
RP1-80N2.3	-0.62298	0.0067561	0.019278
REEP6	-0.62212	0.010472	0.028316
TIMELESS	-0.62205	2.05E-37	8.16E-36
MIB1	-0.62183	5.01E-23	1.02E-21
FAM175A	-0.62165	0.00071272	0.0024956
ASF1B	-0.62164	3.14E-23	6.47E-22
COPZ2	-0.62155	0.0043466	0.012953
GABRB3	-0.62125	1.27E-16	1.78E-15
ZNF19	-0.62104	0.01422	0.036948
BTN3A3	-0.62101	0.0038549	0.011614
MMS22L	-0.62097	9.85E-20	1.67E-18
ZNF277	-0.62035	2.04E-09	1.53E-08
SLC46A3	-0.62022	3.16E-05	0.00013962
SMURF2	-0.62021	2.26E-14	2.70E-13

ZNF280C	-0.62013	2.53E-08	1.69E-07
EML2	-0.61973	8.83E-12	8.30E-11
WASF2	-0.61874	4.80E-21	8.73E-20
RP1-180E22.3	-0.61849	0.00021055	0.00081106
PSRC1	-0.61822	2.80E-09	2.07E-08
TRIP6	-0.61815	4.00E-07	2.31E-06
DEK	-0.6176	1.90E-29	5.42E-28
RP11-767N6.7	-0.61673	0.0071653	0.020316
FAM86B3P	-0.61656	0.0068473	0.019507
HIF1AN	-0.61637	2.06E-32	6.72E-31
EPSTI1	-0.61635	0.017843	0.045164
SWAP70	-0.6161	3.41E-12	3.34E-11
ZNF146	-0.61598	7.28E-21	1.31E-19
PHF11	-0.61577	0.00035371	0.0013101
NRBF2	-0.6157	3.55E-08	2.33E-07
RP11-713C5.1	-0.61553	0.0081135	0.022669
ADCK3	-0.61543	7.25E-38	2.95E-36
BANF1	-0.61504	1.47E-29	4.23E-28
TRUB1	-0.61485	1.83E-21	3.42E-20
AR	-0.61462	2.73E-33	9.36E-32
DLX4	-0.61425	0.0174	0.044162
USO1	-0.61411	6.54E-30	1.90E-28
MAGT1	-0.61352	1.22E-31	3.89E-30
ZFAND3	-0.61329	6.45E-25	1.45E-23
RGS10	-0.61312	1.02E-05	4.91E-05
SLC13A3	-0.61269	1.59E-12	1.60E-11
SHKBP1	-0.61257	1.54E-15	2.00E-14
CENPN	-0.61248	5.17E-16	7.01E-15
NRIP2	-0.61214	0.0033217	0.01016
LYSMD2	-0.61198	2.12E-10	1.76E-09
ABLIM1	-0.61166	1.60E-26	3.90E-25
NDE1	-0.61166	3.89E-10	3.15E-09
RBM43	-0.61133	0.0001796	0.00070021
ANXA9	-0.61039	2.85E-08	1.89E-07
THAP9	-0.61013	1.94E-05	8.86E-05
CDC16	-0.61007	4.92E-21	8.94E-20
ILDR1	-0.60993	1.02E-06	5.58E-06
GPD2	-0.60992	2.32E-19	3.84E-18
ZFP28	-0.60976	0.00018621	0.00072339
HDAC7	-0.60955	1.81E-18	2.88E-17
TMEM168	-0.60943	1.59E-17	2.39E-16
POMT2	-0.60925	8.14E-09	5.73E-08
HCG18	-0.60916	1.07E-10	9.08E-10
PCDH20	-0.60856	2.98E-23	6.17E-22
AP3D1	-0.6084	6.18E-37	2.42E-35

KLK1	-0.60817	2.96E-11	2.65E-10
METTL20	-0.60785	7.30E-06	3.57E-05
QRICH1	-0.60686	1.70E-30	5.14E-29
TRIM25	-0.60657	1.75E-23	3.67E-22
CATSPER2P1	-0.60621	0.0079709	0.022318
GLIS2	-0.60616	0.00012008	0.00048166
RP11-983P16.4	-0.60577	3.35E-11	2.98E-10
NBN	-0.60572	3.39E-22	6.66E-21
UGDH	-0.6051	1.10E-36	4.25E-35
FAM189A2	-0.6047	0.018922	0.047531
ZBTB44	-0.60461	5.58E-18	8.65E-17
ZBTB12	-0.60461	0.0001244	0.00049788
DERL3	-0.60412	6.15E-08	3.92E-07
PIGV	-0.60366	1.78E-12	1.78E-11
GOSR1	-0.60327	1.90E-28	5.16E-27
LGALSL	-0.603	3.87E-22	7.55E-21
TNFAIP8	-0.60299	0.00089494	0.0030736
FAM101B	-0.60295	9.02E-06	4.35E-05
TBC1D10B	-0.6021	1.24E-24	2.77E-23
APH1B	-0.60185	0.00033264	0.0012374
PLCD3	-0.60179	5.79E-08	3.71E-07
SLX4IP	-0.60131	0.0020539	0.0065554
HOOK3	-0.60119	1.72E-19	2.87E-18
TGFBRAP1	-0.60065	2.17E-17	3.25E-16
ZNF681	-0.60054	1.72E-17	2.60E-16
YARS2	-0.60038	2.35E-17	3.50E-16
CASP7	-0.59993	6.24E-12	5.94E-11
EXTL2	-0.59956	1.46E-10	1.23E-09
ANKLE1	-0.59955	0.00097931	0.0033413
KIAA1551	-0.59926	1.07E-13	1.21E-12
C6orf183	-0.59902	0.012598	0.033227
CHRM1	-0.59896	0.014335	0.037194
GRIP2	-0.59848	5.39E-05	0.00022939
B3GALT4	-0.59836	0.00083499	0.0028843
LRRC3	-0.59834	0.0070562	0.020047
NOL10	-0.59806	2.44E-15	3.13E-14
CHM	-0.59784	3.11E-14	3.66E-13
SEC14L4	-0.59748	0.00010449	0.00042351
MAT2B	-0.59728	1.66E-24	3.67E-23
CDKN2C	-0.59723	1.66E-10	1.39E-09
TARBP1	-0.59652	5.84E-15	7.29E-14
MCPH1	-0.59648	1.29E-11	1.19E-10
SS18L2	-0.59612	1.07E-08	7.47E-08
TREH	-0.59606	0.0011372	0.0038317
PPAP2C	-0.59578	0.019458	0.048679

GGACT	-0.59571	2.27E-06	1.19E-05
THNSL2	-0.59499	3.04E-09	2.25E-08
CASP6	-0.59424	1.85E-12	1.85E-11
SMPDL3B	-0.59326	1.04E-07	6.48E-07
KIF4A	-0.59317	2.70E-18	4.25E-17
NREP	-0.59317	0.00010349	0.00041989
AMER3	-0.59253	1.45E-05	6.76E-05
TOR1A	-0.59239	1.92E-21	3.57E-20
AATK	-0.59209	0.00021713	0.00083479
CCDC53	-0.59208	3.27E-10	2.65E-09
CC2D2A	-0.59116	6.90E-07	3.87E-06
AP000525.9	-0.59029	7.52E-19	1.22E-17
FMO4	-0.58987	0.0051285	0.015052
ARHGAP35	-0.58986	9.24E-22	1.75E-20
PLXNC1	-0.58943	0.01375	0.035847
SSR3	-0.5894	8.97E-35	3.26E-33
OAS3	-0.5878	2.36E-10	1.95E-09
MAP2	-0.58684	5.21E-26	1.24E-24
KCTD15	-0.58661	1.35E-16	1.90E-15
RP11-303E16.2	-0.58631	1.51E-06	8.08E-06
ALOX15	-0.58591	1.38E-12	1.39E-11
FAM203A	-0.5858	1.31E-05	6.17E-05
SOLH	-0.58561	7.22E-14	8.23E-13
LRRC58	-0.58541	4.58E-29	1.28E-27
RYR1	-0.58539	9.63E-07	5.29E-06
CD2AP	-0.58445	4.73E-25	1.07E-23
AC016747.3	-0.58445	1.90E-07	1.14E-06
MYO7A	-0.58442	0.0012012	0.0040212
IQGAP2	-0.58428	6.82E-05	0.00028589
ZNF468	-0.58408	1.91E-09	1.44E-08
ZNF470	-0.58383	7.43E-05	0.00030908
NAP1L3	-0.58371	1.36E-13	1.51E-12
CDH2	-0.58357	6.48E-10	5.10E-09
RAD54L	-0.58347	1.86E-06	9.88E-06
RP11-498C9.15	-0.58342	0.019269	0.048273
SLC16A7	-0.58313	6.53E-09	4.65E-08
INPP5B	-0.58313	6.56E-07	3.69E-06
SMC4	-0.58311	9.98E-26	2.33E-24
MBNL3	-0.58281	1.48E-11	1.36E-10
PRDM7	-0.5821	0.015908	0.040759
RP11-1277A3.2	-0.58201	0.020016	0.049853
LOXL3	-0.58179	0.00068349	0.0024022
AEBP1	-0.58168	0.0006483	0.0022891
RP1-193H18.2	-0.58149	0.0031032	0.0095716
PFKFB3	-0.58138	6.70E-17	9.60E-16

AAED1	-0.58116	0.0012887	0.0042872
ADAM1A	-0.58045	4.51E-07	2.59E-06
HERC6	-0.58045	0.0028557	0.0088665
MYBL1	-0.58038	0.00010152	0.00041257
PRKD1	-0.58008	2.13E-12	2.12E-11
NKAIN1	-0.57996	0.00017078	0.0006679
UCP2	-0.57918	6.88E-05	0.00028803
MANEA	-0.5788	2.57E-20	4.48E-19
RIMS1	-0.57851	9.44E-05	0.00038594
HLCS	-0.57847	7.81E-14	8.88E-13
RAB40A	-0.57794	0.011538	0.030806
TM2D1	-0.57791	8.55E-11	7.32E-10
WIPF3	-0.57772	0.0034794	0.010604
PREX1	-0.57715	0.012077	0.032084
DNAJC18	-0.5769	4.45E-05	0.00019213
HDAC6	-0.57678	6.48E-08	4.12E-07
TRIM16L	-0.5766	2.48E-06	1.30E-05
CILP2	-0.57549	4.28E-11	3.77E-10
PPP1R26	-0.57525	2.79E-12	2.74E-11
FAM86B1	-0.57498	0.00010741	0.00043454
MYOF	-0.57475	0.00012062	0.00048352
TRIM66	-0.57443	4.41E-07	2.53E-06
DOC2A	-0.57438	2.94E-05	0.00013071
HMGCS1	-0.57315	8.96E-37	3.48E-35
SOX9	-0.57273	1.07E-26	2.62E-25
TMEM241	-0.57263	9.38E-07	5.16E-06
BRI3BP	-0.57238	3.12E-38	1.29E-36
TMEM87A	-0.57224	3.24E-16	4.46E-15
IDNK	-0.5721	0.00042134	0.0015418
FMO5	-0.57206	4.44E-06	2.24E-05
ATRNL1	-0.57201	0.019282	0.0483
ACOT4	-0.57194	0.0055795	0.016228
FAM83G	-0.57182	0.00069455	0.0024398
SIRT3	-0.57132	1.44E-09	1.09E-08
DNAJB14	-0.57128	3.41E-17	4.99E-16
LNX1	-0.571	5.86E-08	3.75E-07
LZTFL1	-0.57083	4.62E-12	4.46E-11
PAXBP1-AS1	-0.57066	0.0074282	0.02095
RP11-216F19.2	-0.5704	5.89E-05	0.00024974
SLC25A32	-0.57007	1.68E-14	2.02E-13
ZMAT1	-0.56952	0.016155	0.041332
PCDHB7	-0.56944	0.012547	0.033116
BATF3	-0.56926	0.00060339	0.0021421
FAM111A	-0.56922	2.99E-22	5.87E-21
MGAT3	-0.5692	0.010196	0.027669

FEN1	-0.5691	1.07E-22	2.15E-21
NBPF3	-0.56905	2.77E-07	1.63E-06
PCDHA13	-0.56854	0.00037246	0.0013731
RAB3D	-0.56826	3.60E-14	4.20E-13
CENPE	-0.56796	1.42E-22	2.82E-21
TRAF5	-0.5678	7.87E-10	6.15E-09
NXPH4	-0.5675	2.69E-06	1.40E-05
HERC2P4	-0.56737	0.014045	0.036531
ZNF816	-0.56732	3.52E-05	0.00015425
TMEM194B	-0.56729	2.16E-06	1.14E-05
GOLPH3L	-0.56728	2.34E-17	3.49E-16
DDX10	-0.56718	6.12E-19	9.97E-18
TRPC3	-0.56702	0.017284	0.043897
TACC2	-0.56694	9.52E-27	2.34E-25
EME1	-0.56683	2.92E-05	0.00012971
IPO11	-0.56667	3.22E-10	2.62E-09
SMIM19	-0.56656	7.06E-09	5.01E-08
MLKL	-0.56597	0.0044273	0.013172
PCDH7	-0.56587	1.03E-10	8.78E-10
ZNF678	-0.56573	7.46E-16	1.00E-14
CDH6	-0.56556	3.42E-17	5.00E-16
RP11-345J4.8	-0.56553	0.0077434	0.021767
TRA2A	-0.56537	7.18E-18	1.11E-16
SKAP2	-0.56516	7.04E-19	1.14E-17
COPS3	-0.56436	4.50E-22	8.71E-21
NKX2-8	-0.564	0.014565	0.037701
FHDC1	-0.56384	9.67E-07	5.32E-06
MEGF6	-0.56342	0.0010616	0.0036016
GS1-251I9.4	-0.56321	1.76E-14	2.12E-13
TCOF1	-0.56207	3.35E-27	8.49E-26
PALMD	-0.56084	0.0066234	0.018948
LINC00630	-0.56074	0.005431	0.015838
SIMC1	-0.56069	1.52E-07	9.26E-07
THAP9-AS1	-0.56061	2.84E-12	2.79E-11
CECR2	-0.56058	0.018286	0.046153
CTD-2554C21.3	-0.56026	0.01364	0.035599
PELI1	-0.55984	1.58E-06	8.47E-06
POLI	-0.55947	2.56E-13	2.78E-12
SAGE1	-0.55916	1.72E-05	7.93E-05
UNC80	-0.55915	0.0019875	0.0063609
RASIP1	-0.55913	0.00015429	0.00060766
ARRDC4	-0.55863	1.08E-07	6.69E-07
CREB3L4	-0.55819	4.65E-15	5.83E-14
KREMEN2	-0.55731	1.37E-08	9.43E-08
ENPEP	-0.55689	0.011487	0.030678

AKAP6	-0.55652	0.00029015	0.0010926
DHCR24	-0.5563	1.82E-35	6.76E-34
SPTBN4	-0.55627	0.0042215	0.012615
ERICH1	-0.55612	1.09E-10	9.23E-10
COPS8	-0.55578	3.85E-15	4.87E-14
FAM13A	-0.55568	2.26E-11	2.05E-10
ING2	-0.5552	6.28E-06	3.11E-05
RP11-217H19.1	-0.55506	0.013506	0.035297
RAD54B	-0.5548	7.22E-06	3.53E-05
ZNF813	-0.55465	1.96E-05	8.95E-05
PTPN2	-0.55386	1.99E-07	1.19E-06
ZDHHC8	-0.55374	6.00E-11	5.20E-10
MNS1	-0.55357	0.0011774	0.0039536
FAM126A	-0.55267	2.48E-13	2.70E-12
NGEF	-0.55263	1.06E-07	6.56E-07
MFSD4	-0.55257	9.09E-11	7.76E-10
ZNF569	-0.55214	1.79E-05	8.25E-05
CBWD3	-0.55207	0.00035234	0.0013055
ZNF273	-0.55178	7.12E-09	5.04E-08
POLR3GL	-0.55118	7.06E-12	6.68E-11
SHOX2	-0.55046	0.0014301	0.0046996
NCKAP5L	-0.54902	0.0075525	0.021276
TMEM45A	-0.54821	8.65E-10	6.73E-09
RP11-632K20.7	-0.54802	0.0032243	0.0098971
LETM1	-0.54719	2.21E-23	4.60E-22
ARL13B	-0.54706	6.13E-08	3.91E-07
LOXL1-AS1	-0.54693	7.35E-06	3.59E-05
TCF7L2	-0.54659	2.31E-07	1.37E-06
SP3	-0.54647	5.58E-21	1.01E-19
VBP1	-0.54647	2.44E-18	3.83E-17
CDKN2D	-0.54646	1.73E-07	1.05E-06
ENTPD2	-0.5461	0.020056	0.049946
HECTD2	-0.54609	4.69E-08	3.04E-07
SLC25A37	-0.54578	5.97E-18	9.25E-17
THAP6	-0.54549	6.42E-08	4.09E-07
EXOSC2	-0.54501	1.95E-18	3.09E-17
KCNA6	-0.54491	0.001401	0.004615
FAM86JP	-0.54488	1.28E-05	6.05E-05
ATG10	-0.54479	7.38E-10	5.77E-09
GARNL3	-0.54472	0.0017189	0.0055709
RGL3	-0.54468	2.84E-07	1.67E-06
CNPY4	-0.54467	6.65E-05	0.00027918
PLEKHH1	-0.54459	8.10E-11	6.94E-10
ANO10	-0.54457	2.42E-06	1.26E-05
FGFBP3	-0.54445	5.12E-05	0.00021876

SH3PXD2A	-0.54425	7.09E-05	0.00029619
GFRA1	-0.54383	0.00033559	0.0012475
KLHL5	-0.54374	1.49E-06	7.99E-06
CAMK4	-0.54318	6.29E-08	4.01E-07
PYCRL	-0.54308	2.47E-17	3.68E-16
ZBTB16	-0.54302	1.16E-07	7.17E-07
CDK5	-0.54258	2.95E-11	2.64E-10
PNPLA7	-0.54184	0.00093193	0.0031918
SCP2	-0.54175	2.60E-22	5.12E-21
ILF3	-0.54169	9.11E-40	4.01E-38
MANBAL	-0.54146	1.61E-14	1.94E-13
PAX9	-0.54084	1.57E-05	7.29E-05
RP11-390F4.3	-0.54079	6.60E-07	3.71E-06
ALDH6A1	-0.54076	4.80E-29	1.34E-27
AC009237.8	-0.54076	1.27E-05	6.01E-05
ZNF599	-0.54042	0.012023	0.031968
PPP2R3B	-0.54033	8.01E-10	6.25E-09
WDTC1	-0.54023	7.60E-19	1.23E-17
PCDHB13	-0.53996	0.00021635	0.00083197
RP4-694B14.5	-0.53966	0.00017643	0.00068832
WHAMMP3	-0.53918	0.012807	0.033684
TNNI1	-0.53809	0.001677	0.0054511
DLGAP5	-0.53791	1.33E-12	1.34E-11
MBLAC2	-0.5377	3.03E-06	1.56E-05
PIK3R5	-0.5376	0.01819	0.045938
ATP6V1E2	-0.53714	0.0040559	0.012164
CTC-428G20.3	-0.53647	0.0032684	0.010011
CROCC	-0.53643	7.30E-11	6.28E-10
CCDC138	-0.53633	1.98E-06	1.05E-05
BCAT1	-0.53632	9.27E-06	4.46E-05
MAGOH	-0.53626	1.99E-10	1.66E-09
SNX18P7	-0.53622	0.017442	0.044252
LIN7A	-0.53593	2.20E-19	3.65E-18
SLC4A8	-0.53589	0.003754	0.01134
PAPSS1	-0.53521	1.21E-36	4.64E-35
DDX5	-0.53498	2.03E-33	6.99E-32
BCAS3	-0.53476	2.87E-05	0.00012782
PARP11	-0.53459	0.0032752	0.01003
RAB7L1	-0.53399	4.35E-23	8.89E-22
GOLGA4	-0.53398	4.13E-24	8.89E-23
LINC00654	-0.53393	1.24E-05	5.85E-05
F2R	-0.5337	0.012373	0.032721
SH3BGR	-0.53364	0.0011551	0.0038847
RPL22L1	-0.53333	7.22E-18	1.11E-16
POMZP3	-0.53307	7.12E-05	0.00029727

LNPEP	-0.53303	1.84E-07	1.11E-06
TEC	-0.53302	0.018165	0.045885
TMEM180	-0.53274	6.55E-11	5.65E-10
NUCB1	-0.53273	1.12E-23	2.37E-22
C1orf159	-0.53257	6.72E-07	3.77E-06
ACADM	-0.53249	3.15E-24	6.82E-23
RP11-671C19.2	-0.53239	4.40E-05	0.00019017
TCF7	-0.5323	0.0082791	0.023073
NMNAT3	-0.5322	0.0027337	0.0085184
PKN2	-0.53205	1.99E-21	3.69E-20
ZC2HC1A	-0.53154	0.0010539	0.0035768
C8orf22	-0.53152	0.0034922	0.01064
LARP7	-0.53118	1.11E-12	1.13E-11
MAFB	-0.5311	1.64E-09	1.24E-08
MPI	-0.53066	5.46E-18	8.49E-17
ATP6V0E2-AS1	-0.53028	0.0010167	0.0034593
HPSE	-0.53025	2.20E-05	9.92E-05
PORCN	-0.53013	0.0010438	0.0035453
HSPA12A	-0.53007	0.002566	0.0080452
DECR2	-0.52933	1.79E-13	1.97E-12
PRAME	-0.52881	1.30E-15	1.71E-14
RP11-48B3.4	-0.52876	0.00016966	0.0006642
KIAA1377	-0.52875	0.0073382	0.020732
SCAI	-0.52842	0.0013655	0.0045131
RAB22A	-0.52816	3.39E-17	4.97E-16
CAND2	-0.52807	2.19E-09	1.64E-08
HOXB13	-0.52789	7.46E-32	2.40E-30
TTF2	-0.52779	6.04E-17	8.70E-16
ASAH2B	-0.52774	1.25E-05	5.90E-05
FCF1	-0.5274	2.36E-14	2.82E-13
WBSCR27	-0.52693	0.0045732	0.013561
ZNF317	-0.52631	5.51E-12	5.29E-11
PDCD7	-0.52609	7.15E-12	6.77E-11
NOP16	-0.52606	1.07E-09	8.27E-09
ICAM5	-0.52605	4.12E-11	3.63E-10
FAM114A1	-0.52529	9.59E-08	5.97E-07
COCH	-0.5248	0.0032918	0.010076
RAB11B	-0.52469	1.19E-12	1.21E-11
PACSIN1	-0.52445	1.32E-12	1.33E-11
LPXN	-0.52442	3.53E-07	2.06E-06
LDHD	-0.52347	8.92E-07	4.92E-06
6-Sep	-0.52345	0.017605	0.0446
MIR210HG	-0.52294	0.017693	0.044797
NPR1	-0.52271	0.0093382	0.025619
TMEM106B	-0.52264	1.14E-16	1.61E-15

MSN	-0.52226	1.35E-10	1.14E-09
ACSL6	-0.52217	0.00025629	0.00097417
ARHGEF39	-0.52193	1.08E-07	6.67E-07
C12orf23	-0.52175	9.50E-13	9.77E-12
PDLIM2	-0.52169	0.0049427	0.01456
PLTP	-0.52155	1.50E-12	1.51E-11
GNG4	-0.52151	6.20E-11	5.37E-10
KCNN2	-0.52151	7.80E-05	0.00032315
AP3B2	-0.52128	5.35E-05	0.00022784
METTL7B	-0.52127	2.37E-17	3.53E-16
EFNB3	-0.52049	0.0019107	0.0061312
CCNE2	-0.5204	1.55E-06	8.32E-06
PDPK2	-0.52039	0.0083571	0.023251
DBF4B	-0.52008	6.10E-06	3.02E-05
ZFP90	-0.51982	0.0095964	0.026229
FADS1	-0.51969	1.66E-26	4.02E-25
C7orf55-LUC7L	-0.51954	8.82E-15	1.09E-13
RP11-488C13.5	-0.51942	0.013939	0.036264
QSOX1	-0.51926	1.47E-19	2.46E-18
BRCA1	-0.51878	8.45E-13	8.74E-12
PCDHB8	-0.51868	2.93E-05	0.00013042
RP11-566E18.3	-0.51824	0.00026232	0.00099472
DTD1	-0.51817	5.49E-19	8.95E-18
GTF2H3	-0.5179	2.33E-21	4.29E-20
CCDC111	-0.51737	1.21E-07	7.45E-07
CPT1C	-0.51724	1.75E-06	9.30E-06
RAD51D	-0.5171	3.84E-05	0.00016731
MOSPD3	-0.51688	1.01E-05	4.86E-05
ZCCHC11	-0.51686	3.43E-16	4.71E-15
LDLRAD3	-0.51674	1.52E-08	1.04E-07
AIFM3	-0.51645	0.00016727	0.00065563
TBC1D4	-0.51611	1.51E-12	1.52E-11
PAPOLA	-0.51608	5.06E-26	1.20E-24
SVEP1	-0.51607	0.009601	0.026238
RAB15	-0.51586	0.00011409	0.00045913
PARP3	-0.51573	7.67E-06	3.74E-05
CTD-3064H18.6	-0.51534	0.0031081	0.0095852
ANGEL1	-0.51503	4.20E-10	3.37E-09
INO80C	-0.51365	0.0003213	0.0011991
LOXL1	-0.51331	0.0080589	0.022536
TMEM145	-0.51307	6.07E-06	3.01E-05
DTWD1	-0.51291	3.31E-06	1.70E-05
PACS2	-0.51267	3.32E-11	2.96E-10
SLC27A3	-0.5126	1.64E-12	1.65E-11
EFCAB11	-0.5122	3.78E-06	1.92E-05

ZMYND11	-0.51162	1.13E-21	2.13E-20
DDX50	-0.51158	1.17E-14	1.43E-13
FAM81A	-0.51143	3.26E-06	1.67E-05
ZNF415	-0.51084	0.0037756	0.011398
TMEM254	-0.51059	4.70E-14	5.44E-13
DHRS11	-0.51031	2.04E-07	1.22E-06
KIAA1549	-0.50975	3.73E-10	3.01E-09
AL589743.1	-0.5093	1.39E-08	9.54E-08
TCERG1L	-0.50929	0.017292	0.043912
SCAPER	-0.50922	8.34E-05	0.00034376
RASEF	-0.50897	1.22E-07	7.49E-07
DERA	-0.50827	6.89E-08	4.37E-07
RIBC2	-0.50813	0.0011469	0.0038616
KIF11	-0.50769	1.34E-13	1.50E-12
TFDP2	-0.50767	9.74E-19	1.56E-17
DDN	-0.50743	1.40E-14	1.70E-13
MAPK1	-0.50728	3.12E-31	9.71E-30
HOOK2	-0.50695	3.09E-10	2.52E-09
CYP2D6	-0.50689	0.0018235	0.0058752
GLTSCR1L	-0.50671	1.48E-12	1.49E-11
RP11-293B20.1	-0.50637	0.013129	0.03442
NUFIP1	-0.50636	2.50E-07	1.48E-06
NFATC1	-0.50605	0.0021722	0.0069074
IFIH1	-0.50578	9.06E-17	1.28E-15
SPTSSA	-0.50563	9.92E-12	9.29E-11
ALPK1	-0.50549	0.00043585	0.0015884
TMEM14A	-0.505	1.01E-11	9.43E-11
TAF4B	-0.50492	3.50E-07	2.04E-06
BEND3	-0.50482	6.85E-10	5.37E-09
LGR5	-0.50459	1.05E-08	7.31E-08
PRR14L	-0.50449	6.85E-19	1.11E-17
TRMT11	-0.50427	4.43E-06	2.23E-05
CRYBG3	-0.50413	6.63E-07	3.72E-06
PCDHB2	-0.5038	5.64E-10	4.47E-09
NOXA1	-0.50368	0.0010722	0.003635
KCTD17	-0.50339	9.85E-09	6.88E-08
BMP8B	-0.50314	0.00061715	0.0021869
SDK1	-0.50305	0.0085047	0.0236
EMID1	-0.50294	4.35E-07	2.50E-06
KIAA1715	-0.50283	5.78E-11	5.02E-10
INCENP	-0.50174	3.75E-13	3.99E-12
HAUS5	-0.50142	2.48E-10	2.05E-09
TRIP10	-0.50108	0.0010014	0.0034125
ARHGAP15	-0.50098	0.016312	0.041701
BUB3	-0.5009	2.95E-25	6.74E-24

SBDSP1	-0.50087	2.09E-12	2.08E-11
CBFA2T2	-0.50068	6.44E-09	4.59E-08
ZC3H4	-0.50063	2.00E-12	1.99E-11
ZNF326	-0.50059	3.04E-14	3.58E-13
NASP	-0.50036	2.20E-20	3.86E-19
NICN1	-0.50023	4.69E-07	2.69E-06
MBP	0.50033	5.98E-07	3.38E-06
SSBP2	0.50036	2.49E-11	2.25E-10
C6orf120	0.50078	5.29E-16	7.16E-15
TBC1D2	0.50086	0.012894	0.033884
GPX7	0.50095	4.00E-05	0.00017371
ST13	0.50106	7.15E-26	1.68E-24
MAN1C1	0.50125	6.11E-05	0.00025817
RPS6KA4	0.50161	7.93E-15	9.80E-14
TRIM23	0.50168	3.22E-14	3.78E-13
MYO5B	0.5023	3.16E-12	3.09E-11
EIF4EBP1	0.50232	1.06E-15	1.40E-14
B4GALT1	0.50297	3.00E-21	5.51E-20
DMWD	0.50306	1.26E-09	9.63E-09
SOWAHA	0.50344	0.0053192	0.015542
TJP2	0.5048	1.56E-18	2.48E-17
BSDC1	0.50496	7.63E-23	1.54E-21
SYPL1	0.50523	1.02E-15	1.36E-14
POLH	0.50528	4.16E-15	5.24E-14
NADK	0.50534	6.59E-18	1.02E-16
ETNK1	0.50537	1.30E-23	2.74E-22
PPP1R1C	0.50543	0.018462	0.046534
C12orf45	0.50548	7.47E-11	6.41E-10
NMD3	0.50575	5.09E-20	8.75E-19
UPP1	0.50597	0.00012026	0.0004822
C11orf82	0.50602	1.04E-07	6.43E-07
NHLRC3	0.50605	8.73E-07	4.82E-06
CXADR	0.50641	1.46E-13	1.63E-12
ZDHHC8P1	0.50663	1.84E-08	1.25E-07
CREB3	0.5069	7.74E-15	9.57E-14
DOM3Z	0.50727	3.29E-08	2.17E-07
CTD-2196E14.4	0.50738	0.0076392	0.021496
CCDC144NL	0.50754	8.62E-06	4.17E-05
ENOX1	0.50771	1.20E-06	6.49E-06
SLC6A6	0.50817	2.14E-22	4.24E-21
TCP1	0.50819	2.46E-34	8.78E-33
SLC10A7	0.50829	3.19E-08	2.10E-07
ALOXE3	0.50843	4.16E-06	2.11E-05
KIAA0907	0.50854	2.71E-17	4.01E-16
SYNJ1	0.50864	1.22E-12	1.25E-11

RP11-46B11.5	0.50867	4.76E-05	0.00020435
ADAM15	0.50871	1.29E-31	4.09E-30
MED26	0.50878	3.68E-05	0.00016082
RP11-473M20.	0.50894	8.20E-06	3.98E-05
PCDHA4	0.50904	0.0012237	0.0040916
CRB3	0.50916	1.73E-12	1.73E-11
XK	0.5093	5.99E-12	5.72E-11
UHRF1BP1	0.50977	4.74E-20	8.16E-19
GCH1	0.51062	2.16E-14	2.58E-13
TJAP1	0.51066	2.82E-11	2.53E-10
COX5A	0.51159	4.73E-31	1.46E-29
UBE2D3	0.51175	9.15E-26	2.14E-24
ARIH2OS	0.51206	0.0090191	0.024861
ZNF697	0.51269	0.00014969	0.00059131
MLL2	0.51317	0.00051951	0.0018664
SRPR	0.51319	5.12E-35	1.89E-33
ARID4B	0.51335	1.13E-19	1.91E-18
RP11-435B5.4	0.51345	3.27E-07	1.91E-06
NME1	0.51356	6.30E-09	4.50E-08
CMTM8	0.51367	2.34E-06	1.23E-05
CLU	0.51399	6.08E-41	2.83E-39
C12orf49	0.51401	6.75E-21	1.21E-19
WDR1	0.51405	5.88E-39	2.49E-37
C6orf226	0.51432	1.56E-05	7.25E-05
CUTA	0.51434	3.34E-25	7.60E-24
CNOT11	0.51484	2.39E-23	4.97E-22
DYNC1H1	0.51539	1.25E-14	1.53E-13
HOXC10	0.516	0.0084592	0.023495
PTEN	0.51624	5.97E-28	1.57E-26
PSENEN	0.51645	0.0029138	0.0090326
DUSP18	0.51691	0.0041333	0.012367
C9orf40	0.51695	1.03E-11	9.63E-11
ATP5O	0.51702	0.00060767	0.0021565
RUFY3	0.51706	4.60E-12	4.44E-11
GAL3ST1	0.5171	1.34E-05	6.32E-05
UBE3C	0.51725	6.19E-40	2.75E-38
AMER1	0.51772	5.19E-08	3.34E-07
GPATCH3	0.51791	1.32E-07	8.10E-07
GRAMD1A	0.51808	2.86E-11	2.57E-10
SLC43A2	0.51812	1.93E-13	2.12E-12
IQCE	0.51815	1.28E-24	2.84E-23
RP11-18H7.1	0.51815	0.0029795	0.009216
RPL35P2	0.51885	0.00072692	0.0025394
HIGD1A	0.51902	8.00E-21	1.43E-19
SLC25A14	0.51902	5.67E-05	0.00024071

TMEM62	0.51937	1.36E-10	1.14E-09
TMPPE	0.51962	6.41E-06	3.17E-05
TULP4	0.51978	1.44E-12	1.45E-11
ZCCHC3	0.51994	0.009889	0.026947
FOXP1	0.51995	1.14E-11	1.06E-10
NUDT4	0.52046	1.43E-30	4.34E-29
RIPK4	0.52046	4.46E-05	0.00019238
EIF3K	0.52078	9.53E-38	3.87E-36
TLE1	0.52079	7.06E-07	3.95E-06
TDG	0.52107	6.04E-30	1.76E-28
BOLA3	0.52107	2.01E-14	2.41E-13
PTAR1	0.52153	6.57E-15	8.17E-14
LRFN5	0.52179	3.88E-13	4.13E-12
PLBD2	0.52187	6.84E-14	7.82E-13
ABCA1	0.52199	0.007271	0.02058
LRFN4	0.52218	2.16E-05	9.77E-05
SC5D	0.52302	5.03E-18	7.84E-17
SAMD10	0.52305	1.45E-09	1.11E-08
RP11-11N9.4	0.52312	3.27E-05	0.00014379
TMEM199	0.52399	0.0018573	0.0059732
GGA3	0.52416	1.68E-13	1.85E-12
PQLC2	0.52424	1.32E-07	8.11E-07
FOXL2	0.52429	0.0095416	0.026108
CNKSR3	0.52453	2.66E-06	1.38E-05
MAP3K1	0.52459	6.29E-13	6.59E-12
IL1R2	0.5246	0.00086872	0.0029924
AC156455.1	0.52481	1.35E-10	1.14E-09
MEX3C	0.52502	1.14E-20	2.02E-19
RPL39P3	0.52517	0.0075698	0.02132
RP11-21N3.1	0.52604	7.41E-14	8.43E-13
C17orf59	0.52607	2.48E-10	2.05E-09
ASZ1	0.52622	0.019801	0.049411
ARF4	0.52629	8.16E-23	1.65E-21
HGD	0.52654	2.30E-06	1.20E-05
AGBL3	0.52668	0.002529	0.0079356
CDC42	0.52726	4.57E-30	1.35E-28
JPH3	0.52741	5.03E-12	4.84E-11
NBEA	0.52751	6.50E-11	5.61E-10
SLC9A6	0.52772	1.57E-08	1.07E-07
CYB5R3	0.52784	1.99E-24	4.35E-23
TMEM243	0.52801	0.00014756	0.0005836
PBLD	0.52805	2.55E-26	6.15E-25
MDM2	0.52812	3.18E-25	7.26E-24
ST8SIA4	0.52826	1.99E-10	1.65E-09
PRADC1	0.52828	3.39E-16	4.65E-15

LINC00338	0.52851	5.25E-05	0.0002235
FRS2	0.52857	4.01E-22	7.80E-21
PLA2G4F	0.52862	1.36E-12	1.37E-11
ACOT7	0.52874	9.09E-19	1.46E-17
NPAS2	0.52901	0.00030196	0.0011327
FAM63B	0.52902	3.39E-17	4.97E-16
WDR44	0.52917	1.09E-19	1.84E-18
C9orf91	0.52921	5.86E-13	6.14E-12
NCOR2	0.52976	4.50E-05	0.00019374
C6orf203	0.52992	4.96E-09	3.58E-08
UTRN	0.53041	1.89E-05	8.66E-05
SNTB1	0.53052	0.001179	0.0039579
NDUFA4	0.5308	5.87E-24	1.26E-22
SLC1A4	0.53088	8.51E-12	8.02E-11
RICTOR	0.53106	1.17E-10	9.94E-10
HSPA8	0.5311	4.75E-25	1.07E-23
MAPK8IP1	0.53111	6.10E-16	8.24E-15
MORF4L1P1	0.53155	2.17E-07	1.29E-06
EXOC5	0.5317	2.94E-24	6.40E-23
MAPRE3	0.5318	1.29E-09	9.89E-09
UBR3	0.53184	3.56E-16	4.88E-15
C12orf10	0.53194	4.64E-09	3.37E-08
HLA-C	0.53211	1.66E-08	1.13E-07
CNIH	0.5323	3.97E-24	8.55E-23
EIF3C	0.53232	3.08E-06	1.59E-05
HBEGF	0.5324	0.0018531	0.0059628
ZNF689	0.53241	8.67E-10	6.74E-09
ASB14	0.53253	0.017545	0.044468
PDZD11	0.53265	2.21E-10	1.83E-09
GSN	0.53278	5.62E-09	4.03E-08
ATP5G3	0.53308	3.19E-27	8.11E-26
HMGCS2	0.53315	0.003222	0.0098917
LINC00116	0.53316	3.36E-07	1.96E-06
CPOX	0.53381	3.87E-21	7.06E-20
MT-CO3	0.53409	2.76E-07	1.63E-06
RAB30	0.53423	1.20E-07	7.41E-07
SUV420H2	0.53453	2.80E-07	1.65E-06
FLJ27365	0.53459	0.0042465	0.012678
ERBB4	0.53478	9.93E-06	4.76E-05
MTAP	0.53562	1.51E-17	2.29E-16
RP11-694I15.7	0.53562	0.012429	0.032834
ADAM9	0.53589	8.22E-33	2.74E-31
ISG15	0.53605	1.86E-17	2.78E-16
TBC1D9	0.53606	1.40E-16	1.97E-15
TNPO1	0.53608	8.08E-32	2.57E-30

NF2	0.53713	3.19E-28	8.56E-27
RP11-159H10.3	0.53713	0.0028287	0.0087935
PPTC7	0.5372	1.94E-23	4.06E-22
IER2	0.53751	4.10E-14	4.77E-13
PROM2	0.53787	2.61E-28	7.03E-27
DENND4B	0.53791	7.67E-24	1.63E-22
RP11-30J20.1	0.53795	9.24E-07	5.09E-06
OTUD7B	0.53827	1.10E-22	2.19E-21
ACTB	0.53852	1.10E-45	5.94E-44
CDC42SE2	0.53869	1.05E-20	1.87E-19
TRNP1	0.53884	4.39E-22	8.50E-21
SEMA3E	0.5393	1.22E-05	5.76E-05
ATPAF1	0.53958	2.35E-22	4.65E-21
C4orf46	0.53959	2.06E-11	1.87E-10
ADAMTS7	0.53988	0.00042362	0.001549
Z83851.3	0.54031	0.008918	0.02461
C17orf58	0.54042	1.60E-06	8.55E-06
EZH1	0.54043	5.74E-13	6.03E-12
BCLAF1	0.54124	3.21E-25	7.34E-24
GLG1	0.54136	7.97E-18	1.22E-16
CTB-13H5.1	0.54159	0.00011417	0.00045934
ATXN7L3B	0.54206	2.80E-37	1.11E-35
C2orf72	0.54255	2.43E-13	2.64E-12
MIA3	0.54303	4.62E-30	1.36E-28
GALNT18	0.54306	3.97E-06	2.01E-05
CTA-217C2.1	0.54348	1.45E-05	6.80E-05
LMBR1L	0.54374	2.41E-10	1.99E-09
GATC	0.54378	5.14E-14	5.93E-13
C19orf52	0.54378	4.70E-10	3.76E-09
HABP4	0.54382	7.63E-09	5.39E-08
AMDHD2	0.54386	0.0016891	0.0054862
SH3BP5-AS1	0.54409	0.00048049	0.0017344
MT-ATP6	0.5441	5.77E-21	1.04E-19
ANKRD49	0.54414	3.06E-06	1.58E-05
LRPAP1	0.54439	2.14E-29	6.09E-28
BRAF	0.54485	1.45E-15	1.89E-14
SLC45A4	0.54489	1.04E-12	1.07E-11
FAM189A1	0.54501	2.92E-13	3.14E-12
SIAH2	0.54529	2.29E-13	2.50E-12
FBXL18	0.54576	1.71E-10	1.43E-09
GALNT2	0.54599	1.04E-22	2.09E-21
NEDD9	0.54614	6.36E-08	4.04E-07
EIF4E2	0.54647	1.66E-19	2.77E-18
AP5B1	0.54739	8.01E-09	5.64E-08
LANCL3	0.54764	1.05E-06	5.74E-06

SERHL	0.54767	0.013737	0.035823
CLDN15	0.5478	1.19E-08	8.21E-08
RP4-756H11.3	0.54791	0.00015055	0.0005941
PDE12	0.54797	1.58E-33	5.48E-32
DNAJB2	0.54834	1.30E-21	2.45E-20
CCND3	0.54872	9.22E-15	1.13E-13
STS	0.54913	1.54E-08	1.05E-07
C12orf60	0.54922	0.0013584	0.0044948
RECK	0.54927	5.90E-05	0.00025011
CBFB	0.5494	2.18E-21	4.04E-20
MFSD9	0.54981	7.16E-09	5.07E-08
ITGA9	0.54982	0.00032484	0.0012116
MPC1	0.55007	3.08E-09	2.27E-08
NDUFAF6	0.55025	2.13E-14	2.55E-13
MT-ND2	0.55054	9.45E-21	1.68E-19
RP11-122C9.1	0.55073	0.00053428	0.0019146
SLC6A8	0.5508	3.86E-33	1.30E-31
UQCRC1	0.55086	9.86E-50	5.99E-48
RP11-325F22.3	0.55089	0.013813	0.035984
PRPS1	0.55096	5.31E-30	1.56E-28
PTGS2	0.55104	6.40E-10	5.04E-09
DPCD	0.55171	7.99E-13	8.30E-12
ENC1	0.5519	1.21E-16	1.71E-15
PARM1	0.55228	4.39E-19	7.18E-18
PKDCC	0.55246	0.0085546	0.023713
AC009403.2	0.55323	0.00059524	0.0021161
RP11-467D6.1	0.55345	0.0090747	0.025
PDK1	0.55362	2.24E-07	1.33E-06
C14orf2	0.55415	1.31E-19	2.21E-18
ACBD5	0.55421	1.94E-25	4.46E-24
UFD1L	0.55429	9.54E-26	2.23E-24
MAN1A1	0.55477	3.94E-24	8.49E-23
MAMDC4	0.55498	4.94E-07	2.82E-06
RPSAP15	0.55554	0.00018829	0.00073073
RP11-295G20.2	0.55582	0.00015182	0.00059865
CCDC107	0.55618	0.005268	0.015415
PGPEP1	0.55635	4.72E-09	3.42E-08
GATS	0.55716	0.00011334	0.00045647
IQCD	0.5572	0.0047541	0.014045
NEK6	0.55729	1.64E-18	2.62E-17
WBSCR22	0.55732	1.45E-45	7.76E-44
S100PBP	0.55743	2.78E-11	2.50E-10
GIPC1	0.55749	1.38E-24	3.07E-23
TMEM9B	0.55749	5.07E-18	7.89E-17
PSME4	0.55763	2.12E-19	3.52E-18

CD164	0.55768	2.01E-24	4.40E-23
SAT1	0.55803	8.21E-09	5.77E-08
AC025335.1	0.55803	3.01E-05	0.00013348
TMEM68	0.55807	2.14E-18	3.38E-17
SLIRP	0.55829	7.85E-17	1.12E-15
RP11-466H18.1	0.55853	0.00045915	0.0016638
ENTPD7	0.55878	1.77E-10	1.48E-09
UGCG	0.55934	6.58E-14	7.53E-13
ANAPC13	0.55949	1.18E-18	1.88E-17
ANKRD29	0.55968	5.94E-05	0.00025129
CRNDE	0.55989	2.29E-10	1.90E-09
SSX2	0.56003	0.00055427	0.0019819
TNKS2	0.56008	3.16E-30	9.38E-29
PIFO	0.56009	0.0094251	0.025839
HECTD4	0.56128	3.72E-11	3.29E-10
SNHG3	0.56136	1.44E-09	1.10E-08
MGST2	0.56147	2.08E-24	4.54E-23
AUP1	0.56169	2.97E-24	6.45E-23
PLA2G15	0.56216	2.03E-05	9.26E-05
ST8SIA6-AS1	0.56293	8.59E-16	1.15E-14
PDZD8	0.56301	8.03E-19	1.30E-17
RP11-361F15.2	0.5633	0.0090879	0.02503
PRR15L	0.56398	4.56E-11	4.01E-10
SCOC	0.56432	4.99E-20	8.58E-19
LENG8-AS1	0.5644	0.0059231	0.017131
CDH1	0.56444	9.72E-35	3.51E-33
RP11-14N7.2	0.56463	3.41E-13	3.64E-12
HOPX	0.56466	6.65E-07	3.74E-06
GNG5	0.5647	5.48E-15	6.85E-14
ZNF71	0.56472	1.61E-08	1.10E-07
CACUL1	0.5648	5.66E-27	1.41E-25
CEP85L	0.56493	4.84E-06	2.43E-05
GINM1	0.56494	1.40E-20	2.47E-19
CCRN4L	0.56509	1.64E-07	9.92E-07
GMFB	0.5654	3.24E-24	6.99E-23
ATP6V1F	0.56555	1.44E-17	2.18E-16
SMCR8	0.56596	1.06E-11	9.90E-11
E2F7	0.56611	1.52E-16	2.14E-15
PHC3	0.56683	9.66E-16	1.29E-14
CCNG1	0.56702	2.93E-20	5.10E-19
CTC-471F3.4	0.56743	2.23E-05	0.0001007
DANCR	0.5679	2.25E-28	6.11E-27
APOM	0.56806	0.0088148	0.024352
API5	0.5687	2.95E-29	8.29E-28
FOS	0.56919	6.20E-09	4.43E-08

PDXP	0.56959	0.00028295	0.0010679
FAM83F	0.56976	2.63E-06	1.37E-05
ST3GAL1	0.57001	5.48E-36	2.06E-34
PRRT1	0.57014	0.0012709	0.0042373
PRELID1	0.57024	1.34E-15	1.75E-14
FAM134A	0.57026	2.08E-31	6.52E-30
CTD-2287O16.1	0.57045	0.0019949	0.0063835
A2M	0.5708	0.0003263	0.0012159
RNF19B	0.57084	1.55E-10	1.30E-09
SYT15	0.5712	2.45E-07	1.46E-06
AC007318.5	0.57215	0.0017574	0.0056836
RPL18AP3	0.57244	0.00010758	0.00043512
BBIP1	0.5727	1.28E-06	6.92E-06
ZFP3	0.57281	1.17E-06	6.37E-06
RP11-111M22.	0.57446	2.93E-07	1.72E-06
EHD3	0.57452	1.38E-07	8.47E-07
PANK1	0.5746	5.87E-12	5.62E-11
FOLH1B	0.57476	0.00045896	0.0016634
CTD-2196E14.9	0.5749	4.43E-07	2.54E-06
MAP2K3	0.57587	2.95E-32	9.63E-31
GPC6	0.57592	3.00E-17	4.41E-16
SDCBP2	0.5764	0.0041191	0.012335
ATG9A	0.57661	4.12E-21	7.52E-20
MVB12B	0.57671	7.19E-14	8.20E-13
EVI5	0.57678	5.96E-12	5.70E-11
CTD-3065J16.9	0.57679	0.01284	0.033759
C8orf44	0.5768	0.0074082	0.020903
NDUFA2	0.5769	1.97E-05	8.97E-05
MYCBP	0.57692	1.97E-12	1.96E-11
KIAA1919	0.57696	3.82E-07	2.21E-06
EVI5L	0.57699	3.99E-16	5.45E-15
TMEM86A	0.57701	0.00057816	0.0020602
ACSL1	0.57736	7.34E-15	9.10E-14
MORN2	0.57788	1.31E-06	7.10E-06
GEMIN7	0.57802	5.21E-05	0.0002219
KDELC1	0.57828	1.04E-07	6.48E-07
UNKL	0.57859	4.07E-17	5.93E-16
MYPOP	0.57888	1.33E-08	9.17E-08
MCTS1	0.57909	3.04E-14	3.57E-13
SLC19A2	0.57954	3.09E-23	6.39E-22
BRCC3	0.57997	3.03E-16	4.19E-15
CYSTM1	0.58008	1.58E-17	2.39E-16
ZNF276	0.58009	3.20E-16	4.40E-15
DNAJB6	0.58049	1.77E-37	7.10E-36
HHEX	0.58053	0.010247	0.02778

RP11-304L19.5	0.58065	3.43E-06	1.76E-05
CC2D1A	0.58089	7.15E-26	1.68E-24
FRAS1	0.58096	5.95E-16	8.05E-15
ZNF608	0.58096	3.27E-08	2.15E-07
ZDHHC12	0.58153	8.79E-17	1.25E-15
RNASEH1	0.58165	8.61E-16	1.15E-14
RPS6KA3	0.58204	1.03E-28	2.84E-27
NR2F2	0.58217	4.28E-08	2.78E-07
SMCR7	0.58301	1.28E-11	1.19E-10
TUBB2A	0.58313	7.44E-11	6.39E-10
RPS3AP26	0.58315	3.77E-05	0.00016446
RP11-572P18.1	0.58329	0.00059439	0.0021135
EEF1A1P13	0.58331	0.0045778	0.01357
RAMP1	0.58384	2.60E-23	5.38E-22
FGFR1OP	0.58388	1.81E-13	1.99E-12
PNKD	0.58391	1.92E-28	5.20E-27
CEBPD	0.58408	6.91E-16	9.33E-15
TMEM147	0.58561	7.40E-22	1.41E-20
NDUFB1	0.58579	1.50E-17	2.28E-16
SMPD2	0.58582	5.01E-09	3.61E-08
DTX2	0.5865	5.09E-05	0.00021756
PCTP	0.58675	1.47E-15	1.92E-14
GPRC5A	0.58698	1.81E-09	1.36E-08
PRKAB2	0.58724	6.28E-24	1.34E-22
VMP1	0.58769	6.43E-19	1.05E-17
DMPK	0.58844	4.94E-09	3.56E-08
SEC14L1	0.58887	4.02E-15	5.06E-14
DNA2	0.58898	9.79E-13	1.01E-11
HMGA1	0.58995	6.16E-20	1.06E-18
ADCY9	0.5903	1.16E-12	1.18E-11
NEU1	0.59117	1.35E-22	2.69E-21
RP11-680F20.1	0.59139	0.00096585	0.0032982
PCBP4	0.59319	1.02E-26	2.51E-25
ZC3HAV1L	0.59364	2.30E-08	1.54E-07
UBE2G2	0.59409	4.70E-41	2.19E-39
NUTF2	0.5944	3.07E-12	3.00E-11
RP11-157B13.6	0.59448	0.012839	0.033759
HOXC6	0.59456	0.00031653	0.0011828
P4HA2	0.59461	2.80E-07	1.65E-06
RP11-115D19.1	0.59463	0.0020177	0.0064483
RP11-1094M14	0.59469	0.00078944	0.0027351
ABT1	0.59549	4.03E-22	7.83E-21
SHISA5	0.59642	1.02E-40	4.66E-39
RP11-629O1.2	0.5971	0.0087657	0.024227
ATP6V0E1	0.59711	4.30E-23	8.79E-22

APOH	0.59767	1.57E-06	8.39E-06
BCAT2	0.59817	2.44E-26	5.88E-25
PIM2	0.59831	1.42E-14	1.72E-13
ZNF79	0.59979	3.16E-10	2.58E-09
C22orf26	0.59995	0.014875	0.038411
VDAC1	0.60008	1.12E-43	5.70E-42
ZFP62	0.60039	1.99E-24	4.35E-23
NKIRAS1	0.60072	6.31E-06	3.12E-05
C21orf90	0.60084	9.33E-07	5.13E-06
ALG3	0.60164	1.31E-35	4.87E-34
TOLLIP	0.6022	4.17E-19	6.83E-18
NDUFS5	0.60235	1.74E-38	7.20E-37
CDHR3	0.60236	0.0083275	0.023178
DOT1L	0.60282	4.10E-22	7.96E-21
SLC5A6	0.603	3.49E-22	6.84E-21
XBP1	0.60301	1.87E-39	8.14E-38
RP11-169K16.9	0.60349	6.21E-07	3.50E-06
AGO4	0.6037	7.86E-22	1.49E-20
BCR	0.60392	2.49E-17	3.70E-16
ODC1	0.60427	4.08E-54	2.83E-52
MFSD1	0.60428	3.91E-28	1.04E-26
CTD-2006C1.2	0.60439	1.92E-07	1.15E-06
MCFD2	0.60514	1.96E-24	4.31E-23
GOLGA1	0.60518	1.02E-22	2.04E-21
FASTK	0.60541	1.23E-29	3.54E-28
CDC34	0.60661	5.90E-21	1.07E-19
CCNJ	0.60685	4.72E-13	4.99E-12
EMC3	0.60704	2.08E-12	2.06E-11
EMC10	0.60723	3.36E-30	9.94E-29
PPIC	0.60728	2.66E-12	2.62E-11
AP001053.11	0.60804	0.0023257	0.007346
HN1	0.60888	4.38E-33	1.48E-31
FBXL5	0.60919	6.52E-19	1.06E-17
UHMK1	0.61015	1.20E-40	5.49E-39
OCLN	0.61057	1.30E-15	1.70E-14
TMEM234	0.61123	0.00074459	0.0025961
TUSC3	0.61141	1.47E-39	6.41E-38
SMIM13	0.61161	1.85E-17	2.77E-16
GFPT1	0.61214	3.71E-31	1.15E-29
RAB11FIP4	0.61238	4.35E-25	9.83E-24
KLHDC7A	0.61252	0.0016043	0.0052372
ACOT8	0.61374	7.26E-16	9.78E-15
CTD-2017D11.1	0.61409	0.0014884	0.00488
SLC6A9	0.61518	1.62E-07	9.83E-07
PPM1D	0.61543	3.70E-22	7.23E-21

RNF44	0.61555	4.12E-31	1.27E-29
MAPKAPK3	0.61561	8.72E-30	2.53E-28
EIF5AL1	0.61573	9.26E-05	0.00037925
CLTB	0.61597	2.20E-29	6.24E-28
C17orf89	0.61606	8.40E-19	1.35E-17
C3orf58	0.61628	9.60E-28	2.49E-26
RNA28S5	0.61663	1.79E-18	2.85E-17
EMB	0.61729	3.41E-25	7.77E-24
SLC30A1	0.61743	1.62E-28	4.44E-27
KCTD8	0.61746	0.018789	0.047238
SMOC1	0.61756	1.67E-07	1.01E-06
POTEF	0.61756	7.38E-07	4.12E-06
ENPP4	0.61824	3.94E-11	3.49E-10
SDC4	0.6184	2.04E-11	1.86E-10
C5orf15	0.61925	1.74E-21	3.24E-20
RRM2	0.62035	5.14E-40	2.29E-38
ANXA1	0.62082	2.25E-05	0.00010135
FAM109A	0.62086	2.13E-15	2.75E-14
CBR3-AS1	0.62101	0.016784	0.042768
AC004166.7	0.62163	1.53E-11	1.41E-10
SQSTM1	0.62397	7.76E-40	3.43E-38
SLC40A1	0.62403	1.40E-25	3.25E-24
FAM219A	0.62418	2.31E-13	2.52E-12
GUCY1A3	0.62458	3.16E-10	2.58E-09
RNF128	0.62488	9.79E-06	4.70E-05
EXOC2	0.62491	1.06E-20	1.87E-19
RP11-254B13.1	0.62531	0.0086132	0.023859
SLC25A22	0.6254	4.51E-31	1.39E-29
SWI5	0.6258	4.97E-16	6.74E-15
BCL9	0.62611	1.60E-32	5.26E-31
TRHDE	0.62663	1.05E-12	1.08E-11
KIAA1324	0.62695	3.33E-17	4.90E-16
YBX1P10	0.62709	0.007644	0.021506
TMEM45B	0.62711	3.09E-11	2.76E-10
TNFAIP1	0.62782	9.54E-22	1.81E-20
RP11-330M19.	0.62782	0.017043	0.043374
AC128709.1	0.62783	0.00094842	0.0032438
ABHD17AP5	0.62816	0.0036451	0.011043
CYR61	0.6289	2.01E-06	1.06E-05
C17orf82	0.62939	0.008017	0.022434
ERP29	0.62952	4.78E-47	2.72E-45
FAM3C	0.62959	1.40E-27	3.62E-26
DCAF15	0.63046	1.61E-16	2.26E-15
GUSB	0.63073	6.81E-33	2.28E-31
RP11-701H24.7	0.63105	0.0031579	0.0097189

ROBO2	0.63109	2.84E-06	1.47E-05
SARNP	0.63193	7.85E-05	0.00032486
HECTD3	0.63206	7.82E-27	1.93E-25
P2RY11	0.63272	0.00026186	0.00099322
ADM2	0.63275	1.56E-27	4.03E-26
TIMM8B	0.63284	1.02E-32	3.39E-31
ABHD12	0.63327	1.34E-32	4.41E-31
TNFRSF10B	0.63363	3.96E-48	2.31E-46
ARHGEF38	0.63366	0.012533	0.033091
HADHB	0.63436	3.83E-39	1.63E-37
RP11-244J10.1	0.63466	0.012765	0.033577
ATP8A1	0.6349	7.96E-11	6.82E-10
FAM212B	0.63581	1.17E-06	6.37E-06
TPCN1	0.63584	8.67E-35	3.15E-33
YPEL3	0.63604	1.01E-14	1.24E-13
MT-ND5	0.63654	2.04E-10	1.70E-09
PCF11	0.63693	8.78E-23	1.77E-21
RP11-1006G14	0.63694	0.0022714	0.0071959
NCOA1	0.63738	3.54E-15	4.48E-14
SPATA31C2	0.63779	0.0079362	0.022236
PIP4K2A	0.63843	6.50E-12	6.18E-11
FNDC3A	0.63854	1.63E-28	4.46E-27
LRRN1	0.63857	1.15E-05	5.46E-05
PGAM1	0.63895	2.43E-62	2.09E-60
TMED4	0.63924	8.73E-62	7.27E-60
GNRHR2	0.64099	0.00069572	0.0024435
SGK223	0.641	1.00E-16	1.42E-15
ZBTB47	0.64115	1.46E-13	1.62E-12
CHD9	0.64185	5.57E-20	9.56E-19
TM4SF20	0.64382	0.00039387	0.0014472
EOGT	0.64395	5.89E-18	9.12E-17
RP11-15H20.5	0.64439	1.27E-09	9.72E-09
AC009245.3	0.6445	0.019943	0.049709
LINC00638	0.64475	6.84E-05	0.00028649
IER3IP1	0.64533	2.72E-11	2.45E-10
BSCL2	0.64543	0.012355	0.032682
THSD4	0.64581	8.66E-05	0.00035594
SLCO5A1	0.64602	5.46E-15	6.83E-14
DUSP7	0.64732	1.64E-26	3.99E-25
REEP3	0.64744	1.80E-31	5.65E-30
RP11-366L5.1	0.64745	0.019049	0.047805
RP1-265C24.9	0.64816	0.0085367	0.02368
RP11-73M18.8	0.64848	0.00055877	0.0019966
AVPI1	0.64907	0.00020718	0.00079904
NPM1P29	0.65033	0.011095	0.029777

RP11-486A14.1	0.65054	0.0012743	0.0042463
SFXN1	0.65173	6.81E-42	3.26E-40
STK4	0.65216	2.91E-25	6.67E-24
KLC2	0.65284	6.26E-32	2.02E-30
CCDC85A	0.65333	0.0057533	0.016684
PON2	0.65377	3.19E-33	1.08E-31
SIDT2	0.65377	1.06E-22	2.13E-21
DUSP3	0.6539	3.26E-32	1.06E-30
PIKFYVE	0.65495	4.74E-29	1.32E-27
RHOB	0.65501	5.43E-27	1.35E-25
ATF3	0.65508	6.71E-22	1.28E-20
HS3ST3A1	0.65521	0.015673	0.040203
FAM91A1	0.65528	3.04E-39	1.30E-37
KRT18	0.65559	3.71E-43	1.85E-41
RP11-297D21.4	0.65604	0.009569	0.026172
RGPD8	0.65773	1.53E-08	1.05E-07
UGT2B15	0.65827	3.42E-35	1.27E-33
CPM	0.65832	0.00026154	0.00099221
KCTD2	0.65867	5.66E-27	1.41E-25
SEMA7A	0.65946	0.0022606	0.007171
UBN2	0.65995	1.80E-24	3.97E-23
KIAA0101	0.66007	1.23E-32	4.05E-31
TRGC1	0.66041	1.83E-10	1.53E-09
C1orf85	0.66043	2.79E-36	1.06E-34
RP11-15H20.6	0.6605	3.26E-05	0.00014353
MED6	0.66073	1.05E-11	9.82E-11
AGPAT6	0.66119	3.28E-38	1.35E-36
KPNA2	0.66139	4.11E-43	2.04E-41
LEPROTL1	0.66157	1.51E-25	3.51E-24
SETD5-AS1	0.66278	3.77E-14	4.40E-13
KLHL20	0.66415	6.69E-22	1.28E-20
TSSK1A	0.66558	0.019555	0.048867
PRKAR1A	0.66607	1.04E-56	7.83E-55
MYCBP2	0.6664	4.56E-15	5.72E-14
ABHD13	0.66662	2.91E-14	3.44E-13
TNFSF18	0.66677	0.01273	0.033503
FUT4	0.66834	7.67E-07	4.27E-06
ITPRIP	0.66859	0.00021786	0.00083713
GLB1	0.66881	1.66E-36	6.31E-35
C20orf24	0.67001	8.49E-09	5.96E-08
TMED9	0.67008	1.67E-39	7.28E-38
GLA	0.67114	1.03E-20	1.84E-19
TRIAP1	0.67116	6.96E-29	1.92E-27
FEM1C	0.67156	6.89E-29	1.91E-27
RP11-102F4.3	0.67205	3.43E-10	2.78E-09

ATP6V0D1	0.67243	9.64E-35	3.49E-33
RABGAP1	0.67253	5.85E-33	1.96E-31
NDEL1	0.67284	9.80E-20	1.67E-18
GPCPD1	0.67288	1.88E-15	2.43E-14
PIGS	0.67446	2.81E-47	1.60E-45
RP11-159D12.9	0.67446	4.65E-20	8.01E-19
OSBPL5	0.67509	2.12E-18	3.36E-17
ITGB8	0.67514	2.66E-14	3.15E-13
HSPB8	0.67523	1.23E-13	1.37E-12
ABCB9	0.6753	4.27E-13	4.52E-12
C19orf47	0.67532	1.43E-08	9.80E-08
PBX3	0.67538	1.72E-13	1.89E-12
RP11-119B16.2	0.67639	0.0052387	0.015343
RP11-793H13.1	0.67685	0.0001751	0.00068345
ST5	0.67698	1.05E-15	1.39E-14
CHIC1	0.67783	3.23E-10	2.63E-09
ST6GALNAC6	0.67857	1.74E-15	2.26E-14
OST4	0.67882	6.76E-23	1.37E-21
ATF4	0.67909	8.75E-54	6.02E-52
C5orf50	0.67913	0.013393	0.035053
FLRT3	0.68022	7.28E-15	9.03E-14
RP11-553L6.5	0.6805	1.98E-05	9.03E-05
SBF1	0.68067	3.11E-32	1.01E-30
FSTL4	0.68084	0.0055495	0.01615
RP11-160E2.6	0.68127	0.014503	0.037562
CHCHD10	0.68169	2.56E-22	5.06E-21
AQP11	0.68187	0.0046066	0.013645
MAP3K9	0.6821	1.12E-21	2.12E-20
FAM3C2	0.68223	3.33E-08	2.19E-07
BTF3L4	0.6825	5.61E-35	2.07E-33
RP11-502I4.3	0.6825	0.00014835	0.00058623
RP11-379K17.1	0.68251	1.31E-06	7.10E-06
B4GALNT1	0.68259	2.42E-08	1.62E-07
CECR6	0.68282	1.73E-09	1.31E-08
RTCA	0.6831	1.57E-21	2.95E-20
AP006621.1	0.6836	0.003282	0.010049
RNF6	0.68397	3.31E-25	7.54E-24
AHR	0.68404	3.75E-23	7.69E-22
ID3	0.68441	5.20E-42	2.50E-40
LACC1	0.68503	3.40E-06	1.74E-05
PRR24	0.68507	3.21E-05	0.00014141
RP11-588K22.2	0.68599	3.28E-05	0.00014426
RBM38	0.68669	6.60E-23	1.34E-21
PRKAR2A	0.68673	2.25E-50	1.38E-48
RP3-337H4.8	0.68712	1.18E-07	7.29E-07

SPRY4	0.68808	2.02E-13	2.21E-12
TMEM138	0.68826	3.48E-15	4.41E-14
NCOA3	0.68841	3.02E-26	7.26E-25
EEF2K	0.68973	5.73E-28	1.51E-26
CYP2W1	0.6904	0.012095	0.032118
PCGF3	0.69051	1.24E-39	5.41E-38
CTB-92J24.2	0.69072	0.0070132	0.019939
HCN2	0.69092	6.60E-10	5.19E-09
C20orf112	0.69168	1.74E-09	1.31E-08
METRNL	0.69276	0.0028998	0.0089927
SNRK	0.6928	8.19E-22	1.56E-20
AC034193.5	0.69324	1.13E-07	6.95E-07
HSPD1P1	0.69562	0.00075351	0.0026245
AC004057.1	0.69594	4.91E-06	2.46E-05
ELMOD1	0.69632	2.11E-20	3.69E-19
KCTD20	0.69633	1.69E-41	7.99E-40
SPOPL	0.69689	6.99E-19	1.13E-17
GPR157	0.69724	2.17E-09	1.62E-08
STX3	0.69823	3.24E-30	9.62E-29
CTC-559E9.6	0.69824	0.010809	0.029115
ATP6V0A1	0.69844	1.80E-30	5.43E-29
LEF1	0.69859	1.08E-15	1.42E-14
AC058791.2	0.70044	0.0010343	0.0035149
IL18BP	0.70046	2.43E-05	0.00010924
SRM	0.70156	5.18E-38	2.12E-36
RP11-51O6.1	0.70176	0.00040797	0.001496
C2CD4D	0.70275	0.00078725	0.002729
LRP1B	0.70328	0.0027533	0.0085699
ABCC2	0.70347	6.47E-12	6.15E-11
LINC00657	0.70455	9.94E-45	5.22E-43
MAPK6	0.70455	7.94E-32	2.54E-30
AL121578.7	0.70459	0.016947	0.043152
DHRS3	0.70475	4.31E-25	9.76E-24
WEE1	0.70506	9.78E-31	2.98E-29
RP11-334C17.5	0.70576	4.24E-05	0.00018336
DNAJB4	0.70588	6.78E-13	7.08E-12
TMEM133	0.70593	0.0011471	0.0038616
YRDC	0.70594	2.79E-25	6.39E-24
S100A11	0.70647	1.81E-55	1.29E-53
PRDM2	0.7067	6.86E-25	1.54E-23
PPP2CB	0.70678	7.97E-31	2.44E-29
TBC1D20	0.70847	1.97E-32	6.45E-31
BZW1	0.70888	4.23E-41	1.98E-39
IPPK	0.70906	3.56E-10	2.88E-09
MFSD7	0.70941	0.00096051	0.0032823

UGT2B28	0.7098	0.0014336	0.0047104
TMEM189	0.71018	1.79E-16	2.50E-15
CHPF2	0.71027	2.51E-25	5.77E-24
SYNJ2BP	0.71075	2.91E-22	5.72E-21
CES5AP1	0.71145	0.0086367	0.023911
ARHGDIA	0.71155	7.33E-33	2.45E-31
RAPGEF1	0.71299	7.96E-38	3.24E-36
RP11-676M6.1	0.71306	0.0051428	0.015085
DNAH7	0.71313	0.0046124	0.013661
HYAL3	0.71454	6.22E-12	5.93E-11
RPL7AP34	0.71579	0.013429	0.035132
ZMAT3	0.71652	3.60E-45	1.91E-43
DOK3	0.71678	0.00014982	0.0005917
C14orf142	0.71695	9.37E-11	8.00E-10
RP3-322G13.5	0.71752	0.011504	0.030721
PPP1R37	0.71771	3.26E-27	8.28E-26
RP11-655M14.	0.71801	0.0098954	0.02696
USP54	0.71818	4.30E-48	2.50E-46
PLEKHB2	0.71923	1.87E-67	1.87E-65
AC112229.1	0.71931	0.015327	0.039439
RP11-797A18.4	0.71974	0.019745	0.049295
TAGLN	0.72044	7.57E-06	3.69E-05
RP11-872J21.3	0.72221	0.0011544	0.0038837
TLE4	0.72318	1.81E-06	9.59E-06
MAP7D1	0.72447	1.82E-28	4.95E-27
CHD7	0.72632	8.43E-28	2.20E-26
GDF11	0.72736	4.87E-27	1.22E-25
TSPAN5	0.72822	5.26E-08	3.39E-07
ZFAND5	0.72863	1.58E-70	1.76E-68
RP11-887P2.3	0.72926	0.00087976	0.0030268
RP11-294O2.2	0.73029	0.00043825	0.0015955
ZNF280B	0.73044	5.60E-08	3.60E-07
PKD2	0.73094	9.73E-23	1.96E-21
TAC3	0.73144	0.008578	0.023768
RNF103	0.732	9.56E-13	9.83E-12
EEF1A1P20	0.73211	0.011281	0.030211
CRYZ	0.73224	7.69E-39	3.23E-37
RP11-175P13.2	0.73277	0.019934	0.049692
GPRC5D	0.73342	0.00031627	0.0011822
PGAM1P7	0.73371	0.019435	0.048633
ISG20	0.734	5.27E-12	5.06E-11
BLCAP	0.73461	1.20E-43	6.11E-42
RP11-283G6.3	0.73491	0.018636	0.046909
SLC26A7	0.73547	0.012756	0.033561
RP11-616K22.1	0.73575	0.018983	0.047653

MAGEA2	0.73578	0.0003595	0.0013293
RP4-625H18.2	0.73631	0.017004	0.04328
EFCAB14	0.73693	8.54E-57	6.48E-55
ERRFI1	0.73722	2.86E-34	1.02E-32
CDV3	0.73729	8.78E-66	8.37E-64
TPP1	0.73771	1.10E-45	5.96E-44
ORAI3	0.73827	1.55E-10	1.30E-09
NPTN	0.73895	1.04E-37	4.21E-36
LHFP	0.73916	3.81E-12	3.70E-11
SMCR7L	0.73932	1.98E-39	8.58E-38
ARL5B	0.74015	1.85E-40	8.42E-39
FREM2	0.74027	1.17E-11	1.08E-10
PAFAH2	0.74042	4.30E-40	1.92E-38
RP11-175B9.3	0.74127	0.002288	0.0072428
NTSR1	0.74167	0.015875	0.04069
AC004449.6	0.74215	0.017207	0.043739
TCTEX1D4	0.74321	0.00039761	0.0014604
MPZL3	0.7433	4.21E-25	9.56E-24
HIPK3	0.74366	7.27E-41	3.36E-39
AOC4	0.74421	0.011145	0.029887
PRSS23	0.74672	1.34E-55	9.63E-54
RP1-90J20.7	0.74759	0.00052944	0.0018993
CSMD3	0.74788	0.0094493	0.025902
CCDC96	0.74806	0.010419	0.028184
CTC-329D1.2	0.74818	0.0027135	0.0084647
TMEM127	0.74819	1.65E-44	8.58E-43
SMPD1	0.74821	1.06E-18	1.70E-17
ATP6V1B1	0.74841	0.017508	0.044385
GNS	0.7486	1.56E-59	1.24E-57
CTD-2545H1.2	0.74948	0.01528	0.039339
CADM2	0.75086	7.03E-06	3.45E-05
HSD17B1	0.75109	0.00076802	0.0026698
FAM160B1	0.75132	2.61E-27	6.69E-26
C18orf56	0.75162	0.0050636	0.014872
GLUD1P3	0.75201	0.0029064	0.0090112
C9orf37	0.7534	8.99E-16	1.20E-14
PI15	0.75468	0.011744	0.031275
CMPK1	0.75479	1.29E-38	5.38E-37
RP4-730K3.3	0.75484	0.016645	0.042442
ID2	0.7551	3.80E-33	1.28E-31
LINC00470	0.75519	0.0078258	0.021977
SSFA2	0.75548	1.79E-67	1.79E-65
SLC25A20	0.75627	2.78E-29	7.84E-28
UQCR11	0.75687	0.016245	0.041557
FAM86HP	0.75712	3.03E-05	0.00013442

ACHE	0.75727	0.0073112	0.020673
SLC7A6	0.75787	4.14E-11	3.65E-10
EID2B	0.75882	0.00068214	0.0023983
POTEI	0.75902	0.014947	0.038569
BCL2L11	0.75946	2.40E-23	4.97E-22
SLC48A1	0.76021	2.40E-28	6.48E-27
FAM127A	0.76045	2.70E-24	5.88E-23
HIST2H2AA4	0.76075	0.012127	0.032183
KHNYN	0.76166	8.20E-19	1.32E-17
ANKRD9	0.76178	7.18E-05	0.00029947
CTC-459F4.1	0.76275	0.00071606	0.002506
AKR1C4	0.76313	0.0064704	0.018556
MYCN	0.76401	0.0077379	0.021755
SLC35D2	0.76422	2.64E-20	4.60E-19
SREK1IP1	0.7644	9.97E-34	3.48E-32
DUSP2	0.76468	4.42E-29	1.23E-27
GRHL3	0.76562	4.97E-05	0.0002129
MAP2K4	0.76576	1.83E-40	8.32E-39
ATP6V0B	0.76633	1.44E-56	1.06E-54
CTD-2341M24.	0.76648	0.0022953	0.0072614
SDHAF1	0.76649	1.21E-18	1.94E-17
VLDLR	0.76654	3.23E-07	1.89E-06
RP11-398F12.1	0.76685	4.70E-09	3.41E-08
NDN	0.76866	2.72E-28	7.31E-27
RP11-654E17.2	0.7688	0.0067974	0.019381
ABCC5	0.76885	1.40E-45	7.53E-44
RP11-223I10.1	0.76887	0.014352	0.037227
MAGEA12	0.76899	1.26E-34	4.54E-33
TPPP3	0.76902	5.46E-15	6.83E-14
SCN1B	0.76978	0.0063254	0.018196
DSTYK	0.76998	1.97E-39	8.54E-38
LSM11	0.7702	1.76E-19	2.93E-18
C2CD2L	0.77154	6.53E-35	2.39E-33
ZNF154	0.77266	0.013553	0.035404
C2orf69	0.77314	6.45E-27	1.60E-25
RGS22	0.77352	0.013878	0.03613
RP11-456K23.1	0.77373	0.0063461	0.018247
CTB-157D17.1	0.77505	0.00093878	0.0032125
TP53INP2	0.77684	3.80E-28	1.02E-26
MTATP6P1	0.77695	4.65E-07	2.67E-06
AGTR1	0.77735	3.10E-38	1.28E-36
CDH17	0.77794	5.91E-30	1.73E-28
CPT1A	0.77838	3.84E-46	2.11E-44
CLCN5	0.77845	4.91E-32	1.59E-30
SLC16A14	0.77851	8.75E-29	2.41E-27

RP1-59D14.6	0.77911	0.00055435	0.0019819
TMEM64	0.77927	4.92E-42	2.37E-40
BAK1	0.78003	1.73E-41	8.19E-40
SLAIN1	0.78021	3.94E-56	2.86E-54
GYLTL1B	0.78055	8.18E-18	1.25E-16
IGF2BP2	0.78096	7.15E-27	1.77E-25
RPRM	0.78115	0.010074	0.027394
RAB36	0.78117	3.15E-21	5.77E-20
ADRA2A	0.78124	6.58E-20	1.12E-18
DOCK11	0.78142	1.91E-06	1.01E-05
PARD6B	0.78246	9.53E-12	8.94E-11
HIST1H3J	0.78257	0.01298	0.034089
IL6R	0.7838	6.83E-42	3.26E-40
BCL2L12	0.78384	2.51E-37	9.98E-36
SOBP	0.78401	4.71E-12	4.54E-11
AL020996.1	0.78424	0.012391	0.03276
RTEL1-TNFRSF	0.7859	0.00029132	0.0010965
RC3H2	0.78607	1.09E-50	6.72E-49
AGPAT9	0.78679	0.00096542	0.0032973
ST13P6	0.78755	0.01227	0.032479
RHOBTB1	0.78756	1.04E-38	4.35E-37
APOF	0.78798	0.0082599	0.023026
TRAPPC1	0.78851	3.77E-34	1.33E-32
C15orf37	0.78893	0.010752	0.028994
RP11-434B12.1	0.78928	0.005377	0.01569
C16orf52	0.78945	1.56E-15	2.04E-14
AC027763.2	0.7899	0.0032995	0.010098
RAB11FIP5	0.78993	1.60E-17	2.41E-16
FRMD4B	0.78998	6.55E-11	5.65E-10
MAP3K3	0.79017	1.58E-12	1.59E-11
RP11-805L22.3	0.79048	0.010626	0.028693
SPRYD4	0.79083	2.26E-31	7.07E-30
DNHD1	0.79126	1.79E-08	1.21E-07
RP6-109B7.3	0.79146	5.46E-05	0.00023203
HIST2H2BF	0.79222	0.00086891	0.0029926
RP5-1148A21.3	0.79268	0.0004228	0.0015466
RP11-593F23.1	0.79282	0.0069146	0.019681
U47924.27	0.79289	0.00029016	0.0010926
CEACAM19	0.7931	0.01186	0.031568
FOXO1	0.79486	2.99E-11	2.67E-10
BRWD1	0.79488	5.66E-45	2.99E-43
RP11-363E7.4	0.79575	1.58E-13	1.75E-12
CORO7	0.79663	0.0011899	0.0039895
VCAN	0.79666	2.96E-27	7.54E-26
CNIH3	0.7967	0.003313	0.010135

PLAGL2	0.79738	1.06E-28	2.91E-27
SLIT2	0.7976	3.91E-06	1.99E-05
TNFRSF21	0.79787	3.39E-96	6.49E-94
EYS	0.79899	0.0037085	0.011213
GARS	0.79902	3.09E-71	3.49E-69
TACR2	0.79913	0.010078	0.027397
LIMD1-AS1	0.8011	0.011033	0.029635
HIST1H2AE	0.8018	0.0095898	0.026215
BMF	0.80186	2.57E-17	3.82E-16
KIAA0319	0.80246	0.0057085	0.016569
HIST2H3D	0.8026	0.0073243	0.020701
NECAP1	0.8033	4.92E-59	3.89E-57
LRRC73	0.80351	3.17E-09	2.34E-08
HIC2	0.80373	1.91E-08	1.29E-07
AC104135.3	0.80451	4.94E-11	4.32E-10
MTND2P28	0.80533	2.79E-27	7.11E-26
RP13-1032I1.7	0.80544	1.20E-05	5.69E-05
RP11-262H14.5	0.80566	7.38E-06	3.61E-05
BTG3	0.80583	1.62E-29	4.64E-28
PLXNA2	0.80728	4.01E-27	1.01E-25
HS3ST5	0.8076	0.010402	0.028143
CCDC178	0.80876	0.0093515	0.025645
RANBP6	0.81055	5.06E-28	1.34E-26
GIGYF1	0.81338	3.06E-34	1.09E-32
RP11-715J22.2	0.81452	0.0091206	0.025099
RTN4	0.81456	1.97E-60	1.58E-58
HIST1H3E	0.81571	0.00023562	0.00090009
KCNQ1OT1	0.8175	0.00029233	0.0010995
TMEM27	0.81791	6.58E-14	7.53E-13
SENP5	0.8182	1.65E-45	8.81E-44
DYRK2	0.81862	1.60E-37	6.44E-36
EN2	0.81911	0.0031957	0.0098215
RIMKLA	0.81995	1.79E-05	8.25E-05
GLIS3	0.82012	4.07E-09	2.96E-08
FAM46A	0.8204	9.86E-76	1.23E-73
TMEM245	0.82127	4.08E-55	2.89E-53
ISL2	0.82127	0.0091575	0.025193
NR4A1	0.82186	3.31E-15	4.21E-14
TMEM55B	0.82257	1.52E-21	2.86E-20
HS6ST2	0.82263	6.88E-28	1.80E-26
DACH1	0.82272	4.91E-16	6.67E-15
AC110619.1	0.8231	0.00056966	0.0020318
IRGQ	0.82365	2.70E-53	1.84E-51
ARHGAP29	0.824	5.66E-14	6.51E-13
AL513477.1	0.82562	0.0030331	0.0093684

SNCG	0.82632	4.28E-64	3.87E-62
ALDH1A1	0.82638	6.40E-77	8.20E-75
LARP1P1	0.82688	0.0025117	0.0078852
MYLIP	0.82691	7.18E-18	1.11E-16
UQCRFS1P1	0.82693	3.69E-11	3.27E-10
ITGA5	0.82704	1.81E-24	3.99E-23
HTT	0.83083	4.43E-38	1.81E-36
RP4-794H19.2	0.83147	0.0082859	0.023082
HOTAIR	0.83171	0.0017294	0.0056004
DUSP5	0.83233	2.66E-06	1.38E-05
FANK1	0.83248	0.0082695	0.023049
PLEKHF2	0.83323	3.28E-34	1.16E-32
RP5-827C21.4	0.83522	0.0014782	0.0048472
VPS25	0.83535	4.12E-51	2.60E-49
CNEP1R1	0.83628	2.94E-17	4.33E-16
RP1-151F17.1	0.83655	0.0022718	0.0071962
ACVR2B-AS1	0.83673	7.14E-05	0.0002981
PYGM	0.83681	0.0078665	0.022066
RP11-461A8.4	0.83765	0.0024579	0.0077313
HDHD1	0.83861	1.68E-27	4.32E-26
C21orf88	0.83953	0.0028823	0.0089432
ASB5	0.83988	0.0042903	0.012797
PSAT1	0.84073	8.85E-70	9.60E-68
CACNB2	0.84173	6.13E-12	5.85E-11
EXT1	0.84246	3.42E-27	8.64E-26
MCOLN1	0.84322	1.21E-22	2.40E-21
SYNGR2	0.84372	2.62E-72	3.04E-70
ABHD14B	0.84391	5.37E-66	5.14E-64
SLC38A7	0.84438	2.56E-30	7.65E-29
DOK6	0.84496	6.64E-41	3.07E-39
AP1S3	0.84541	4.90E-32	1.59E-30
MCL1	0.84584	1.33E-90	2.24E-88
LHX9	0.84651	3.43E-13	3.66E-12
RP11-453F18_	0.84661	0.00015464	0.00060879
NLRC4	0.84745	0.00086066	0.0029668
MSTN	0.84919	0.0051984	0.015236
AC074138.3	0.85039	2.50E-05	0.00011228
LXN	0.85052	1.27E-36	4.88E-35
CFL1P1	0.85289	0.00084501	0.0029164
JUND	0.85301	8.40E-31	2.57E-29
CREB5	0.85361	0.0043251	0.012893
FTLP14	0.85404	0.0001641	0.00064421
RP11-1109M24	0.85419	8.72E-06	4.22E-05
ANTXR2	0.85536	2.98E-21	5.47E-20
RFX2	0.85538	2.01E-07	1.20E-06

ASH1L-AS1	0.85612	3.79E-07	2.20E-06
FOSL2	0.85667	6.42E-13	6.71E-12
TOB1	0.85748	6.13E-71	6.87E-69
CXorf67	0.85755	0.0007403	0.0025835
DTNA	0.85763	1.09E-20	1.94E-19
FIGN	0.85766	1.93E-08	1.30E-07
AXDND1	0.85844	0.0037147	0.01123
ZFYVE26	0.85892	7.34E-23	1.49E-21
RASD1	0.85925	0.0032017	0.0098337
ATP11A	0.86047	0.0009964	0.0033973
RP11-552M11.	0.86226	0.0044325	0.013183
MAN2A1	0.86307	4.98E-56	3.59E-54
ARHGAP42	0.86314	1.32E-12	1.34E-11
SMIM4	0.86371	2.89E-21	5.33E-20
SH3TC1	0.86388	1.21E-10	1.02E-09
AC005003.1	0.86403	1.58E-15	2.05E-14
CTGF	0.86458	1.55E-11	1.43E-10
DCTN5	0.86574	9.66E-47	5.41E-45
SGPP1	0.86667	8.42E-48	4.86E-46
MKNK2	0.86771	3.73E-75	4.52E-73
GABARAPL1	0.86898	4.82E-17	6.99E-16
NAGPA	0.86988	5.77E-17	8.32E-16
ROR1	0.87006	0.0024441	0.0076962
IFRD1	0.87052	5.72E-65	5.35E-63
BCO2	0.87176	0.0056795	0.016492
C9orf41	0.87187	6.68E-69	6.93E-67
RP11-1191J2.2	0.87239	0.005386	0.015711
ATP8B5P	0.87281	0.005613	0.016313
SMLR1	0.87412	0.005534	0.016112
RNF43	0.87495	9.18E-36	3.44E-34
ATP6V1G1	0.87523	4.08E-53	2.77E-51
HIST1H2AI	0.87635	1.62E-05	7.53E-05
KBTBD8	0.87689	3.26E-14	3.82E-13
FAT1	0.87879	1.69E-25	3.91E-24
GPR63	0.88049	6.97E-11	6.00E-10
HIST1H4E	0.88114	0.0050114	0.014733
TRPV3	0.88144	2.91E-15	3.72E-14
NR4A2	0.88157	1.69E-13	1.86E-12
TVP23B	0.88241	3.31E-46	1.83E-44
ARSJ	0.88254	2.60E-06	1.35E-05
PEX5L	0.88619	0.00014709	0.00058185
RPS6KA2	0.88671	1.94E-17	2.91E-16
AC026740.1	0.887	0.00028092	0.0010606
POU6F2-AS2	0.88793	0.0044653	0.013265
DOCK4	0.88939	9.70E-11	8.28E-10

DUSP1	0.88962	3.94E-15	4.98E-14
NR1H2	0.89055	7.01E-48	4.06E-46
FYCO1	0.89121	1.94E-52	1.30E-50
PRTG	0.893	1.16E-13	1.30E-12
BMP6P1	0.89482	0.0033992	0.010378
SEPT7P9	0.89492	0.0044454	0.013218
ASNA1	0.89514	1.42E-69	1.53E-67
PLCXD1	0.89536	2.21E-65	2.10E-63
YBX1P1	0.89825	1.26E-05	5.95E-05
KLHL15	0.90081	3.18E-34	1.12E-32
CDK8	0.90436	1.82E-37	7.25E-36
FURIN	0.9061	2.92E-56	2.14E-54
ACER2	0.90758	2.83E-19	4.66E-18
MTMR9	0.9109	8.57E-30	2.49E-28
ATP10B	0.911	0.0035208	0.010716
RP13-317D12.3	0.91219	0.0033031	0.010108
KIAA0513	0.91231	2.97E-17	4.37E-16
RGS16	0.91306	2.55E-19	4.22E-18
RP6-206I17.1	0.9134	0.00011965	0.00048012
AC006538.1	0.91396	3.77E-06	1.92E-05
RP5-1057J7.6	0.91439	0.0032128	0.0098665
LYPLA2	0.91446	1.09E-71	1.24E-69
UGT2B7	0.91466	2.56E-10	2.11E-09
ZBTB5	0.91525	4.36E-50	2.66E-48
LTB4R	0.91647	1.02E-06	5.59E-06
RP11-381O7.1	0.91727	0.0035784	0.010864
PNPLA6	0.91784	1.96E-48	1.15E-46
TGFBR3	0.9197	2.74E-10	2.25E-09
CAPN2	0.92025	7.54E-21	1.35E-19
SLC30A7	0.92055	6.68E-52	4.35E-50
B3GAT3	0.92069	4.89E-56	3.53E-54
CTC-534A2.2	0.92183	2.31E-12	2.29E-11
PSD3	0.92574	7.72E-43	3.76E-41
CDK5R2	0.93122	7.47E-05	0.00031033
LCOR	0.9318	7.64E-52	4.96E-50
RP11-790I12.1	0.93205	0.0029793	0.009216
CTC-505O3.2	0.93246	0.0027238	0.0084944
ATP6V1D	0.93382	1.54E-44	8.05E-43
SHCBP1	0.93423	5.39E-52	3.54E-50
RP1-140K8.5	0.93472	0.0018541	0.005965
AC007038.7	0.93539	0.0025711	0.0080588
INTS6-AS1	0.93674	0.0011791	0.0039579
ADAM19	0.93958	0.00096723	0.0033024
PPP2R5A	0.93988	9.84E-63	8.67E-61
KRT18P5	0.94002	0.0027666	0.00861

CPEB4	0.9412	6.01E-53	4.06E-51
FOXC1	0.94191	6.21E-05	0.00026188
AC022532.1	0.94218	0.0027508	0.0085636
ERO1L	0.94431	6.85E-80	9.26E-78
CRY2	0.94473	1.31E-38	5.47E-37
RP4-635E18.6	0.945	0.0025116	0.0078852
SECISBP2L	0.94696	2.50E-69	2.64E-67
KCND1	0.9492	1.08E-05	5.13E-05
FGD6	0.95023	7.73E-24	1.64E-22
IER5L	0.95044	2.97E-14	3.50E-13
RP11-164P12.4	0.95058	3.03E-07	1.77E-06
FGB	0.95097	0.0013136	0.0043604
SCRT2	0.95194	0.0012218	0.0040866
RP11-465N4.4	0.95268	6.30E-05	0.00026548
AURKC	0.95277	0.0024627	0.007745
ULK1	0.95289	5.88E-51	3.70E-49
RASSF6	0.95441	2.33E-05	0.00010512
NR1I3	0.95524	3.92E-08	2.55E-07
ANKRD52	0.95633	2.25E-62	1.95E-60
EIF3CL	0.95674	3.61E-06	1.84E-05
SLC23A3	0.959	0.00014652	0.00057984
ITM2A	0.96056	3.17E-05	0.00014009
GCNT3	0.96116	1.97E-08	1.33E-07
LGR4	0.96296	8.21E-55	5.76E-53
STOM	0.9631	8.05E-111	2.06E-108
VMA21	0.96454	3.32E-49	1.99E-47
GALNTL6	0.96568	0.00032908	0.0012251
PKD1L1	0.96578	0.0016337	0.0053254
POLQ	0.96615	1.49E-45	7.95E-44
SESN1	0.96621	2.79E-66	2.72E-64
LINC00869	0.9666	0.0013266	0.0043991
CTNND2	0.96718	6.58E-26	1.55E-24
BICC1	0.97179	1.27E-20	2.25E-19
NODAL	0.97336	0.0016309	0.005319
ZBTB34	0.97642	1.36E-23	2.85E-22
GDA	0.97643	0.001692	0.0054937
RP11-403I13.8	0.97652	7.40E-07	4.13E-06
CTD-2104P17.1	0.97659	1.79E-05	8.22E-05
AC093162.5	0.97664	1.65E-05	7.65E-05
MFAP3L	0.97725	1.64E-09	1.24E-08
REC8	0.97991	0.00014691	0.00058124
LRRC66	0.98013	0.0018636	0.0059897
MIER2	0.98112	1.85E-19	3.09E-18
CA15P1	0.98141	0.0017491	0.0056595
RP11-169K16.6	0.98205	3.49E-08	2.29E-07

DUSP6	0.98463	3.80E-56	2.77E-54
BZW1P2	0.98489	5.82E-06	2.89E-05
AGR2	0.98597	3.24E-29	9.08E-28
MLANA	0.98622	0.00035603	0.0013175
LATS2	0.98801	7.85E-39	3.29E-37
PARP8	0.98952	3.33E-26	7.99E-25
WBP1LP2	0.99133	0.00071701	0.0025089
SLC11A2	0.9931	6.01E-84	8.71E-82
TCF24	0.99553	0.00078227	0.0027136
CD24P4	0.99835	4.75E-09	3.44E-08
RP11-199F11.2	0.99882	0.0002452	0.00093508
FAM195B	0.99985	0.00053455	0.0019152
ZNFX1	1.0012	1.71E-35	6.34E-34
LAMB3	1.0018	6.13E-17	8.83E-16
LINC00324	1.0027	1.54E-12	1.54E-11
TREX2	1.0027	0.0011365	0.00383
FAM177B	1.0041	2.04E-05	9.26E-05
PRSS22	1.0044	0.0012737	0.0042458
MKRN3	1.0057	2.91E-17	4.30E-16
FAM154B	1.01	0.001298	0.0043138
RP11-59D5__B	1.0106	1.69E-05	7.81E-05
ACTA2	1.0115	6.94E-14	7.92E-13
C12orf76	1.0124	6.54E-22	1.25E-20
AC009133.15	1.0131	0.0011841	0.0039734
KLF4	1.0133	1.20E-27	3.09E-26
PTP4A1	1.0164	6.30E-82	8.69E-80
DNAJC27	1.0167	2.21E-18	3.49E-17
ARID3B	1.0207	7.72E-15	9.54E-14
KIF1C	1.0221	1.13E-92	2.00E-90
SPRYD3	1.0237	2.35E-76	2.98E-74
LRRC10B	1.0243	0.00020895	0.00080555
HIST1H2BE	1.0252	0.0007796	0.0027058
DCUN1D3	1.0258	7.08E-20	1.21E-18
DUSP16	1.0262	6.64E-83	9.43E-81
HIST3H2BB	1.0264	8.49E-05	0.00034968
SLC38A9	1.0276	8.12E-21	1.45E-19
ZNF367	1.0277	2.85E-52	1.90E-50
TNFRSF12A	1.0278	1.45E-22	2.89E-21
RPS6KB2	1.0299	7.80E-62	6.55E-60
AC093673.5	1.0302	1.06E-05	5.06E-05
MAPK4	1.0326	5.35E-28	1.41E-26
CTSA	1.0351	6.14E-120	1.95E-117
ESR2	1.0376	0.00016281	0.00063942
AMOTL2	1.0379	1.58E-31	4.96E-30
MIR4720	1.0389	3.07E-39	1.31E-37

JUN	1.0398	9.99E-48	5.75E-46
RP11-395P17.3	1.0424	3.60E-16	4.93E-15
RP11-83N9.5	1.0428	1.65E-07	1.00E-06
QRFP	1.0438	0.00078426	0.00272
CROT	1.0449	3.82E-88	6.12E-86
SESN3	1.0452	1.00E-84	1.49E-82
PHLDA3	1.0455	2.93E-66	2.84E-64
FAM179A	1.0476	0.00087697	0.0030187
LRRC31	1.0507	6.26E-07	3.53E-06
RP11-399O19.8	1.0511	5.03E-05	0.00021548
B3GALT5	1.0587	6.90E-06	3.39E-05
RP1-102E24.8	1.0593	1.57E-05	7.32E-05
RP11-147I3.1	1.0606	0.00019731	0.00076336
FAM160A1	1.0659	2.27E-41	1.06E-39
MTF2	1.0662	1.85E-66	1.80E-64
AKIRIN1	1.0687	1.77E-88	2.86E-86
STEAP4	1.0717	2.81E-07	1.65E-06
CCND1	1.0718	2.22E-191	2.30E-188
AC074117.10	1.0723	7.72E-22	1.47E-20
TSPAN3	1.0724	1.48E-134	5.97E-132
CPEB2	1.0725	5.60E-23	1.14E-21
TP53INP1	1.074	5.53E-124	1.85E-121
RP11-134G8.8	1.0761	1.01E-27	2.61E-26
SLC22A23	1.0794	2.54E-62	2.18E-60
RP11-235E17.5	1.0845	0.00025001	0.00095196
BCMO1	1.0874	6.82E-11	5.87E-10
RP11-98I9.4	1.0951	3.39E-05	0.00014885
SLC17A1	1.0954	0.00045364	0.001646
ATP8B1	1.0964	1.61E-57	1.24E-55
FAM135A	1.0991	1.10E-56	8.28E-55
ARID3A	1.0997	3.85E-37	1.52E-35
SPATA2	1.1019	3.65E-40	1.64E-38
SIPA1L2	1.1024	3.61E-100	7.51E-98
IGLV11-55	1.1026	0.00036738	0.0013567
HIST1H2BC	1.105	7.82E-05	0.00032378
DDX3Y	1.1081	3.00E-110	7.58E-108
SCML2	1.1103	3.58E-66	3.44E-64
RP3-388M5.9	1.1106	0.00017178	0.0006717
TPRG1L	1.1119	6.33E-89	1.03E-86
FGF22	1.1125	7.81E-06	3.80E-05
CDC25A	1.113	3.10E-93	5.57E-91
HIST2H2AC	1.1135	8.65E-09	6.06E-08
HCN4	1.1156	0.00019748	0.00076387
ZCCHC24	1.117	6.39E-25	1.44E-23
OTUD3	1.1186	2.07E-56	1.52E-54

COL4A2	1.1189	1.88E-14	2.26E-13
HIST1H3H	1.1206	1.66E-12	1.66E-11
RFTN1	1.1219	5.20E-08	3.35E-07
PPP2R1B	1.1221	2.60E-92	4.54E-90
RP11-490H24.5	1.1274	0.00013033	0.00052024
RP11-21L23.2	1.131	1.52E-10	1.27E-09
RP1-34B20.4	1.1321	0.00032243	0.0012031
AGO2	1.1337	1.58E-42	7.64E-41
CYP3A5	1.1366	6.95E-05	0.00029084
TBX2	1.1386	1.24E-11	1.15E-10
IGF2BP3	1.1408	6.43E-58	5.02E-56
FST	1.1499	1.30E-36	4.97E-35
PLEKHA1	1.1524	2.56E-69	2.69E-67
UGT2B11	1.1585	1.96E-24	4.30E-23
SIK1	1.1649	7.28E-37	2.84E-35
CLN8	1.1674	5.08E-94	9.37E-92
CDC37L1	1.169	7.10E-51	4.45E-49
FAS	1.1732	1.46E-76	1.86E-74
ABCA12	1.1822	6.24E-11	5.40E-10
GPR137B	1.1888	1.27E-56	9.44E-55
GAN	1.1986	1.75E-33	6.04E-32
PHLPP2	1.2006	6.44E-73	7.60E-71
ERP27	1.2036	5.66E-05	0.00024021
NCEH1	1.2058	9.06E-62	7.51E-60
C11orf95	1.2096	5.54E-52	3.63E-50
FAM102A	1.2171	2.48E-57	1.90E-55
PCDH19	1.2273	3.18E-10	2.59E-09
SESN2	1.2291	5.98E-115	1.82E-112
PGM2L1	1.2346	3.53E-15	4.47E-14
PLK2	1.2351	4.97E-57	3.80E-55
GCNT4	1.2357	6.66E-05	0.00027937
HIST3H2A	1.2378	7.49E-51	4.68E-49
HIST1H2BK	1.238	7.03E-64	6.25E-62
UBASH3B	1.2389	6.29E-08	4.01E-07
MIR22HG	1.2447	9.40E-22	1.78E-20
TMC7	1.2454	5.52E-21	1.00E-19
RP11-297L17.2	1.258	4.35E-07	2.50E-06
CTA-204B4.6	1.2613	1.18E-158	7.27E-156
CUBN	1.2657	9.69E-07	5.32E-06
NEAT1	1.2745	3.67E-133	1.45E-130
SNORD3A	1.295	3.98E-05	0.00017308
ENPP5	1.2961	2.26E-45	1.20E-43
PMAIP1	1.2971	2.46E-56	1.81E-54
HIST1H2BJ	1.3115	1.96E-07	1.18E-06
TMEM2	1.3143	2.28E-135	9.37E-133

DNAJB9	1.3178	1.60E-50	9.84E-49
ELF3	1.3215	5.92E-107	1.39E-104
NR6A1	1.3223	8.91E-18	1.36E-16
RP11-24B21.1	1.3267	5.71E-06	2.84E-05
RNF216P1	1.3333	2.01E-43	1.01E-41
KLHL28	1.3384	1.89E-44	9.73E-43
HIST1H2BF	1.3448	2.98E-06	1.54E-05
AL022341.3	1.3544	2.91E-06	1.50E-05
NR4A3	1.3713	5.30E-10	4.22E-09
XKR8	1.3816	2.05E-30	6.13E-29
DICER1	1.3858	1.32E-192	1.45E-189
CGN	1.388	1.19E-189	1.12E-186
DHRS2	1.3931	3.24E-115	9.99E-113
HIC1	1.4045	6.87E-10	5.39E-09
PANX2	1.4184	2.33E-69	2.48E-67
HIST1H2AG	1.424	2.35E-28	6.35E-27
CPEB3	1.4432	2.43E-35	8.99E-34
TUSC2	1.453	2.52E-159	1.66E-156
BMP2	1.4534	1.66E-129	6.07E-127
AC005281.1	1.4555	1.70E-06	9.07E-06
CDKN1A	1.4666	3.11E-268	6.82E-265
RP11-540O11.1	1.4729	2.53E-09	1.88E-08
AOC3	1.4754	2.62E-14	3.11E-13
IQCJ-SCHIP1	1.4763	7.85E-16	1.05E-14
LINC00622	1.4823	4.84E-07	2.76E-06
NRBP1	1.4854	4.81E-146	2.37E-143
DAPK1	1.49	1.31E-10	1.10E-09
RP11-469M7.1	1.4955	6.09E-22	1.17E-20
EDIL3	1.5352	2.00E-69	2.13E-67
CTD-2561B21.5	1.5368	5.83E-07	3.30E-06
FAM84A	1.5381	8.76E-251	1.73E-247
VGLL3	1.5414	6.93E-12	6.56E-11
HIST2H2BE	1.5617	1.72E-155	9.99E-153
PPT2	1.5652	1.21E-62	1.06E-60
KCNG2	1.5731	8.16E-09	5.74E-08
CD74	1.5798	3.08E-07	1.81E-06
AOC2	1.5985	1.86E-27	4.78E-26
DUSP8	1.6089	2.05E-75	2.51E-73
GDF15	1.6093	8.06E-160	5.49E-157
ADM	1.6736	2.88E-14	3.40E-13
ANKRD46	1.6741	1.11E-109	2.78E-107
PLSCR4	1.69	1.25E-41	5.95E-40
UGT2B4	1.7002	6.58E-28	1.72E-26
HSD3BP5	1.732	7.46E-09	5.27E-08
YOD1	1.7452	1.17E-205	1.36E-202

DUSP4	1.7667	3.47E-23	7.12E-22
IL8	1.7829	6.48E-09	4.61E-08
HIST1H2BG	1.8249	8.36E-12	7.88E-11
HIST1H4H	1.8264	1.55E-17	2.34E-16
HIST1H2BD	1.8869	1.89E-36	7.19E-35
C7orf43	1.915	7.53E-175	6.46E-172
HIST1H2AC	2.027	2.97E-149	1.54E-146
HMGA2	2.0917	1.72E-92	3.04E-90
BASP1	2.1029	1.50E-44	7.85E-43
HIST1H1C	2.1112	5.42E-88	8.63E-86
CGA	2.176	4.68E-27	1.17E-25
CHAC1	2.2612	2.79E-62	2.38E-60
C8orf4	2.306	3.63E-52	2.41E-50

Table S2. Downregulatd and upregulated genes upon SMAD2 KD in Rv1 cells

GeneName	log2FoldChange	pvalue	padj
UBE2Q1	-2.4703	0	0
CLRN3	-2.4095	6.74E-25	2.01E-23
HHLA2	-2.364	5.61E-29	2.09E-27
SLC26A3	-2.3219	2.54E-32	1.16E-30
MIR210HG	-2.1957	4.95E-19	1.08E-17
TIMP2	-2.1578	6.67E-19	1.44E-17
B4GALT3	-2.1478	1.52E-256	5.68E-253
SMAD2	-2.1315	5.72E-181	6.66E-178
RPIA	-2.082	5.95E-159	5.28E-156
NIPSNAP1	-2.0283	7.30E-295	3.40E-291
MFN1	-2.0025	1.61E-178	1.77E-175
RAB14	-1.9813	5.38E-233	9.12E-230
CDC26	-1.9302	9.72E-28	3.41E-26
TM4SF20	-1.8791	3.32E-14	4.93E-13
TMEM9B	-1.8515	1.05E-111	4.24E-109
FOPNL	-1.8028	7.96E-115	3.62E-112
FAF1	-1.7717	3.15E-164	3.09E-161
MFN2	-1.766	1.77E-238	3.29E-235
CAV1	-1.7469	1.33E-37	8.12E-36
DSCC1	-1.6809	1.70E-90	5.01E-88
SLCO2B1	-1.674	1.55E-12	2.00E-11
PDCL3	-1.6665	5.56E-105	2.03E-102
CKS1B	-1.6618	1.92E-148	1.32E-145
TPM3	-1.6485	7.19E-239	1.49E-235
CHRNA2	-1.6405	5.45E-51	5.77E-49
C1orf53	-1.5914	1.51E-13	2.14E-12
SCD	-1.5841	4.42E-247	1.18E-243
C7orf31	-1.5711	1.29E-11	1.53E-10
APEX2	-1.5609	1.98E-61	2.77E-59
TMEM19	-1.5531	1.33E-66	2.15E-64
OAS3	-1.5097	8.28E-49	8.04E-47
SH2D5	-1.5065	2.43E-08	2.00E-07
TNFSF4	-1.5062	5.46E-08	4.31E-07
SLC7A8	-1.4573	3.94E-43	3.06E-41
WDR76	-1.4461	1.10E-41	8.01E-40
ENHO	-1.4444	1.98E-11	2.31E-10
GPD2	-1.4411	1.61E-80	3.75E-78
GLRX5	-1.4403	5.63E-91	1.69E-88
ZBTB9	-1.4363	1.07E-39	7.14E-38
AGPAT2	-1.433	6.32E-39	4.13E-37
PPM1N	-1.4287	3.87E-10	3.89E-09
WNT7B	-1.4151	3.14E-14	4.68E-13

WWC3	-1.4084	4.90E-43	3.79E-41
MT1G	-1.3925	3.01E-42	2.25E-40
SESN1	-1.3925	2.00E-87	5.39E-85
TP53I11	-1.3815	8.47E-93	2.63E-90
MYH14	-1.3678	9.14E-65	1.36E-62
BIRC5	-1.367	8.82E-43	6.68E-41
KIF21B	-1.3629	2.03E-12	2.59E-11
CTA-445C9.14	-1.3621	3.10E-14	4.64E-13
ATP6V0B	-1.3591	1.22E-116	5.97E-114
CYP2C19	-1.3452	1.89E-06	1.18E-05
CAND2	-1.3398	6.50E-37	3.77E-35
SIGLEC10	-1.3382	2.14E-06	1.33E-05
HRH3	-1.3334	2.27E-07	1.64E-06
MT1F	-1.3308	2.23E-07	1.61E-06
THEM6	-1.3301	1.07E-58	1.42E-56
CUL5	-1.3294	5.11E-104	1.83E-101
IGIP	-1.3198	1.09E-10	1.17E-09
RP11-204M4.2	-1.3174	1.34E-17	2.59E-16
FITM2	-1.3158	2.22E-40	1.52E-38
TRHDE	-1.3128	7.76E-31	3.28E-29
LINC00339	-1.3091	7.46E-19	1.60E-17
DDX60	-1.3024	8.12E-11	8.79E-10
TDG	-1.3003	6.25E-106	2.33E-103
PXMP4	-1.2971	7.91E-44	6.38E-42
JDP2	-1.2914	1.89E-22	5.02E-21
PFAS	-1.2901	1.18E-75	2.42E-73
NLRP6	-1.2862	5.14E-07	3.53E-06
SLC39A10	-1.2801	4.92E-77	1.08E-74
TCF19	-1.2762	1.97E-29	7.49E-28
TMEM164	-1.2728	1.98E-65	3.04E-63
CAPN5	-1.2669	5.20E-23	1.43E-21
SMCO4	-1.2639	3.51E-66	5.59E-64
OSTM1	-1.2638	2.90E-40	1.98E-38
ANKS4B	-1.2624	2.95E-07	2.09E-06
ESRRA	-1.2611	9.33E-80	2.15E-77
SHD	-1.2571	3.53E-08	2.85E-07
FAM122B	-1.2487	3.07E-55	3.82E-53
LMOD1	-1.2436	6.83E-07	4.59E-06
PPFIA4	-1.2324	7.51E-06	4.29E-05
GIPC1	-1.2301	3.67E-68	6.27E-66
ZC3H6	-1.2291	5.25E-35	2.79E-33
ZNF512	-1.2245	3.10E-26	1.02E-24
GIPC3	-1.223	3.21E-12	4.02E-11
SLC2A10	-1.2224	2.28E-06	1.41E-05
CPNE5	-1.222	1.29E-05	7.13E-05

CPNE1	-1.2138	1.45E-60	2.02E-58
C20orf27	-1.2129	1.15E-68	2.00E-66
UHMK1	-1.2114	6.23E-129	3.14E-126
HMGN2	-1.2074	8.34E-193	1.11E-189
MNX1	-1.2024	2.04E-14	3.09E-13
CCDC134	-1.202	8.23E-16	1.41E-14
B4GALT6	-1.1846	1.18E-12	1.54E-11
AC093323.3	-1.1836	3.53E-78	7.82E-76
MYO19	-1.1812	4.34E-53	4.90E-51
CYP2C8	-1.1804	2.38E-12	3.02E-11
LINC00341	-1.1747	2.42E-05	0.00012835
CHMP1A	-1.1708	2.42E-90	6.94E-88
MFSD3	-1.1667	5.00E-50	5.12E-48
PPP1CA	-1.1617	5.20E-82	1.31E-79
MSR1	-1.1543	6.40E-06	3.71E-05
DOCK9	-1.1523	1.14E-33	5.57E-32
MYRF	-1.15	7.18E-39	4.66E-37
MYO1A	-1.1424	9.12E-33	4.27E-31
PPP1R3F	-1.141	3.64E-15	5.97E-14
PAQR8	-1.1356	6.80E-18	1.35E-16
DIAPH1	-1.1342	4.59E-101	1.55E-98
CELSR2	-1.1326	1.25E-30	5.18E-29
C10orf32	-1.1313	1.52E-30	6.19E-29
BMX	-1.1251	2.90E-14	4.34E-13
CTD-2008P7.1	-1.122	4.84E-05	0.00024549
HMGCS1	-1.1211	2.20E-135	1.24E-132
CDCA7	-1.1209	2.59E-29	9.83E-28
VAPB	-1.1171	1.19E-81	2.96E-79
HSD3B7	-1.1132	2.54E-52	2.80E-50
PLA2G15	-1.1117	6.72E-12	8.20E-11
ASF1B	-1.0992	2.18E-58	2.86E-56
PIF1	-1.0981	2.66E-11	3.05E-10
ACTL8	-1.0976	2.29E-05	0.0001218
PPP1R3G	-1.0973	8.02E-05	0.00039246
USH1C	-1.093	3.38E-05	0.00017578
ZBTB10	-1.092	1.36E-134	7.47E-132
FGFR4	-1.0911	1.44E-34	7.46E-33
ADH6	-1.0876	8.17E-06	4.65E-05
RP11-1094M14	-1.0859	2.20E-07	1.59E-06
CTD-2008P7.6	-1.0846	0.0001202	0.00056832
ARL6IP6	-1.0825	1.77E-17	3.37E-16
EXOSC6	-1.0761	3.10E-38	1.97E-36
HTRA1	-1.0743	1.85E-18	3.85E-17
AKT2	-1.073	1.95E-88	5.43E-86
FAM213B	-1.0673	2.50E-16	4.45E-15

OTOGL	-1.0669	3.32E-05	0.00017314
RP13-379L11.2	-1.061	0.00014694	0.00067955
HMGN2P5	-1.0506	6.93E-10	6.80E-09
C19orf12	-1.045	6.06E-28	2.16E-26
RNF157	-1.0428	9.80E-22	2.49E-20
ANAPC11	-1.0426	3.00E-31	1.31E-29
CISD3	-1.0418	1.32E-55	1.65E-53
SLC6A17	-1.0396	3.46E-05	0.00018001
KHK	-1.0392	8.45E-09	7.35E-08
CARD11	-1.0388	2.43E-05	0.00012848
TMEM82	-1.0385	0.00022925	0.0010192
PTH2	-1.0365	0.00014704	0.00067975
SLC19A1	-1.0362	5.13E-30	2.03E-28
PANK1	-1.0354	1.14E-24	3.35E-23
TGFBR1	-1.0353	1.48E-20	3.56E-19
SPAG5	-1.0341	3.95E-29	1.48E-27
NOL3	-1.0319	1.41E-18	2.97E-17
DCAF12	-1.0314	3.44E-94	1.09E-91
HRNR	-1.0286	1.32E-05	7.27E-05
KCNJ14	-1.0261	1.43E-07	1.07E-06
ZBTB18	-1.0252	1.04E-06	6.83E-06
FANCC	-1.0223	4.01E-33	1.90E-31
RP11-61A14.3	-1.0198	1.06E-13	1.53E-12
CCDC126	-1.0159	2.14E-14	3.25E-13
RASA1	-1.0136	2.91E-37	1.73E-35
ULBP1	-1.0122	0.00025257	0.0011125
CHAF1A	-1.0118	8.16E-45	6.94E-43
CHP1	-1.0117	1.04E-76	2.26E-74
C7orf55-LUC7L	-1.0105	2.92E-43	2.29E-41
MVK	-1.0097	8.69E-43	6.61E-41
BVES	-1.0093	0.0001071	0.00051062
RP5-1129J21.3	-1.0086	4.10E-11	4.60E-10
C1orf106	-1.0072	1.03E-34	5.37E-33
HSD17B6	-1.0069	9.23E-05	0.00044599
MARS	-1.0065	9.26E-101	3.08E-98
DMBX1	-1.006	0.00033667	0.0014391
PLXND1	-1.0014	1.06E-30	4.42E-29
C16orf80	-0.99957	6.98E-25	2.08E-23
PCNXL3	-0.99835	6.77E-47	6.15E-45
HTRA3	-0.99634	0.00012109	0.00057192
UBFD1	-0.9948	2.13E-69	3.78E-67
TAPBP	-0.99425	2.42E-37	1.45E-35
CHAC1	-0.99323	6.32E-06	3.67E-05
FAM89B	-0.99297	0.00043629	0.0018244
TAGLN2	-0.99208	3.11E-144	1.93E-141

DENND4B	-0.99204	7.80E-53	8.65E-51
SSRP1	-0.98979	2.60E-95	8.35E-93
DARS2	-0.98899	6.48E-88	1.78E-85
SLC17A4	-0.98862	2.70E-08	2.21E-07
EBP	-0.98817	5.59E-70	1.01E-67
TYRO3	-0.98815	4.00E-34	2.00E-32
RP11-318L16.6	-0.98651	2.41E-06	1.49E-05
MESP2	-0.98462	0.00044943	0.001874
TWF2	-0.98283	5.38E-16	9.32E-15
BNIP3	-0.98181	1.56E-53	1.85E-51
GNG13	-0.98072	0.0002153	0.00096158
C16orf59	-0.97991	3.19E-26	1.04E-24
TELO2	-0.97968	1.07E-29	4.15E-28
WEE1	-0.97748	7.14E-36	3.90E-34
RP11-500G10.[illegible]	-0.97721	1.07E-05	5.97E-05
ACBD7	-0.9763	6.05E-11	6.66E-10
HBA2	-0.97624	0.00031002	0.0013356
LIG3	-0.97614	9.02E-60	1.21E-57
CDC25B	-0.97335	2.64E-50	2.75E-48
PKMYT1	-0.96996	2.87E-27	9.89E-26
PPIL3	-0.96966	1.70E-14	2.62E-13
SLC7A9	-0.96696	0.00060452	0.0024423
PIK3R6	-0.9658	0.00033101	0.0014169
COL1A1	-0.96541	2.44E-08	2.00E-07
ZNF551	-0.9637	7.95E-18	1.56E-16
BCAT1	-0.96298	1.04E-14	1.64E-13
POGLUT1	-0.96273	9.67E-20	2.21E-18
JPH1	-0.96036	3.48E-43	2.72E-41
PLA2G4D	-0.95935	8.73E-05	0.00042349
C17orf51	-0.95899	2.69E-10	2.76E-09
SCFD2	-0.9588	1.96E-22	5.21E-21
DBP	-0.95451	2.60E-09	2.40E-08
RP11-509E16.1	-0.9523	0.00045861	0.0019091
NELFB	-0.94655	1.08E-43	8.68E-42
SQLE	-0.94588	7.11E-55	8.72E-53
MSN	-0.94422	2.00E-27	6.92E-26
DOCK1	-0.94417	5.66E-38	3.57E-36
SREBF1	-0.94397	1.62E-30	6.59E-29
AP1S3	-0.94183	3.10E-22	8.12E-21
CTC-429P9.1	-0.94117	4.44E-05	0.00022684
RP11-498C9.15	-0.94098	0.00012123	0.00057246
HPS6	-0.94058	2.75E-26	9.01E-25
GINS2	-0.93962	3.69E-27	1.26E-25
DPM1	-0.93944	3.27E-32	1.50E-30
RP11-280F2.2	-0.93715	1.13E-05	6.31E-05

PRELID2	-0.93699	0.0006519	0.0026122
CTD-3074O7.1[illegible]	-0.93571	8.79E-12	1.06E-10
PPAP2C	-0.93433	0.0001587	0.00072796
NUDCD3	-0.93387	9.09E-75	1.82E-72
INMT	-0.93373	0.00015892	0.00072862
AOX1	-0.93319	1.52E-07	1.13E-06
ALOX15	-0.93319	2.88E-25	8.81E-24
ARL10	-0.92929	5.04E-13	6.82E-12
RRP1B	-0.92851	3.93E-48	3.76E-46
TRIM6	-0.92726	1.20E-05	6.65E-05
APMAP	-0.92658	3.30E-30	1.32E-28
CYP2U1	-0.9258	2.33E-23	6.50E-22
TBC1D25	-0.92554	6.86E-13	9.13E-12
FAM109B	-0.92521	3.79E-08	3.04E-07
TNS4	-0.92496	9.82E-06	5.54E-05
MAP2K1	-0.92208	6.30E-41	4.41E-39
LOH12CR2	-0.92127	0.00023131	0.0010265
RAB39A	-0.92016	2.41E-14	3.63E-13
UCK2	-0.91841	4.20E-92	1.28E-89
DHCR24	-0.9177	2.38E-112	1.03E-109
JAKMIP3	-0.91548	0.00090737	0.0035297
HPCAL4	-0.91476	0.00096312	0.0037178
KCNQ1	-0.91459	0.00026756	0.0011725
C1QL4	-0.91432	1.68E-13	2.38E-12
SLC5A12	-0.91428	0.0011943	0.0045058
RFXAP	-0.91352	4.53E-09	4.08E-08
IDNK	-0.90997	6.18E-07	4.19E-06
NXPH4	-0.90908	6.42E-11	7.03E-10
TIMELESS	-0.90902	3.73E-72	7.01E-70
F11R	-0.90874	1.13E-44	9.54E-43
SART3	-0.90592	4.20E-57	5.40E-55
DNPEP	-0.90435	8.59E-28	3.02E-26
VCP	-0.90266	2.53E-114	1.12E-111
RP11-573D15.[illegible]	-0.90263	5.74E-05	0.00028816
PLK4	-0.90127	1.19E-23	3.38E-22
C8orf22	-0.9007	1.23E-05	6.82E-05
PPP1R3E	-0.90024	6.65E-07	4.49E-06
KCNQ2	-0.89923	4.10E-06	2.45E-05
AKR1C1	-0.89834	1.09E-33	5.33E-32
PPOX	-0.89753	4.42E-13	6.04E-12
ARL6IP1	-0.89723	2.74E-53	3.15E-51
NUP93	-0.89647	7.34E-74	1.44E-71
RP11-649A18.[illegible]	-0.89603	4.41E-05	0.00022552
GNAZ	-0.8952	5.28E-15	8.53E-14
VPS25	-0.89349	1.87E-30	7.58E-29

NHSL1	-0.89342	3.55E-50	3.68E-48
RSAD2	-0.89284	8.61E-05	0.00041807
C9orf78	-0.89214	1.02E-44	8.66E-43
PGLS	-0.89133	5.82E-31	2.49E-29
OGDH	-0.89013	2.89E-74	5.73E-72
SNAP25	-0.88856	3.68E-08	2.96E-07
EFR3B	-0.88821	0.00054151	0.0022132
CMTM8	-0.8845	6.32E-10	6.25E-09
PARP11	-0.88316	1.12E-05	6.27E-05
ESAM	-0.88071	0.001115	0.0042442
AIFM2	-0.88057	0.00031178	0.0013425
RP4-659J6.2	-0.88042	3.78E-08	3.04E-07
AC018696.4	-0.87808	0.0016778	0.0061032
MPP2	-0.87732	2.41E-13	3.35E-12
NELFE	-0.87721	3.17E-49	3.11E-47
OIP5	-0.87634	1.10E-09	1.06E-08
RP11-541N10.3	-0.87584	1.97E-05	0.00010586
RP5-1085F17.3	-0.87573	0.00027114	0.0011854
PGBD5	-0.87501	4.75E-11	5.27E-10
LGALS3BP	-0.87369	7.56E-05	0.00037137
KISS1R	-0.87363	1.55E-08	1.31E-07
KIF1A	-0.87342	6.99E-32	3.14E-30
NUDT19	-0.87324	1.86E-32	8.61E-31
KCNH6	-0.87223	1.89E-14	2.89E-13
FTSJ2	-0.87173	5.39E-44	4.42E-42
FBXO43	-0.8712	0.0010019	0.0038484
ELP6	-0.86922	5.82E-34	2.91E-32
IQGAP2	-0.86878	1.09E-07	8.24E-07
PPP1R14A	-0.86799	0.001906	0.0068231
C14orf1	-0.86727	2.29E-45	2.01E-43
IPO9	-0.86401	1.03E-65	1.62E-63
RBPJ	-0.86317	1.72E-53	2.03E-51
KIF18B	-0.86249	5.93E-32	2.69E-30
C19orf57	-0.86208	1.54E-05	8.37E-05
LSS	-0.86086	1.74E-39	1.16E-37
DNAJB2	-0.86061	2.34E-32	1.08E-30
POLD3	-0.86035	3.72E-18	7.55E-17
POMT2	-0.85916	1.63E-17	3.13E-16
SKA2	-0.85661	4.35E-28	1.56E-26
UTP18	-0.85581	2.98E-37	1.76E-35
STUB1	-0.85398	3.22E-30	1.29E-28
PDP2	-0.85206	4.04E-27	1.38E-25
SENP2	-0.85156	8.44E-38	5.20E-36
SLC27A3	-0.85148	4.85E-31	2.09E-29
CHCHD7	-0.85099	1.17E-22	3.14E-21

FAM174B	-0.85095	0.00013233	0.00061905
SEMA4F	-0.85076	2.19E-09	2.03E-08
LRRTM4	-0.85071	0.00090902	0.0035353
RASA4	-0.85014	7.11E-09	6.25E-08
CDCA5	-0.84978	2.32E-34	1.19E-32
RP11-488L18.1	-0.84804	4.87E-07	3.36E-06
TRMT11	-0.84785	1.33E-16	2.40E-15
WNT10A	-0.8476	0.0012351	0.0046465
FANCD2	-0.84727	1.03E-26	3.44E-25
CTD-3184A7.4	-0.84719	4.30E-05	0.0002205
CCDC15	-0.84702	2.11E-06	1.32E-05
SEC61A2	-0.84589	1.11E-09	1.06E-08
NT5M	-0.84539	5.81E-10	5.77E-09
UBE2D3	-0.84447	6.23E-65	9.36E-63
CTB-36H16.2	-0.84369	2.00E-05	0.000107
WDR62	-0.84338	2.38E-11	2.75E-10
SPHK1	-0.84206	4.08E-06	2.44E-05
CTC-429P9.5	-0.84192	0.00013088	0.00061319
CENPM	-0.84089	2.12E-25	6.55E-24
MX1	-0.84043	1.51E-10	1.59E-09
B4GALT7	-0.8402	1.12E-15	1.91E-14
PPP2R1A	-0.83891	1.49E-60	2.06E-58
TCTEX1D2	-0.83845	0.00043433	0.0018178
MYBL2	-0.83687	6.46E-34	3.21E-32
AC091801.1	-0.83458	0.0010725	0.0040941
PRR15L	-0.83448	1.59E-12	2.04E-11
USP37	-0.83365	3.20E-17	6.00E-16
CHRM4	-0.8333	0.0030658	0.010445
RASL11A	-0.8321	7.06E-08	5.48E-07
DCDC2	-0.83192	0.00066862	0.0026735
RP11-305N23.1	-0.83177	5.71E-07	3.89E-06
MT1E	-0.8307	7.35E-29	2.71E-27
C15orf38	-0.83065	4.13E-22	1.08E-20
NES	-0.82988	6.98E-11	7.63E-10
ZFAND2B	-0.82864	6.16E-15	9.86E-14
FGFR3	-0.82864	4.66E-30	1.86E-28
RP3-462E2.5	-0.8285	5.24E-05	0.00026455
RNF141	-0.82722	6.37E-34	3.17E-32
CTD-2196E14.4	-0.82665	0.00073558	0.0029155
EME1	-0.82661	1.26E-09	1.20E-08
CCKBR	-0.82625	3.27E-07	2.31E-06
TMEM65	-0.82469	4.28E-36	2.37E-34
CDC6	-0.82417	1.37E-30	5.65E-29
C18orf56	-0.82403	0.0033584	0.011273
RP5-837J1.2	-0.82349	6.56E-06	3.79E-05

PNMA2	-0.82344	9.06E-40	6.07E-38
RNF168	-0.82203	6.70E-32	3.02E-30
TRPV4	-0.82101	2.12E-08	1.76E-07
RADIL	-0.82055	3.09E-05	0.00016144
RASIP1	-0.82001	1.33E-07	9.90E-07
MPHOSPH9	-0.81972	4.74E-25	1.43E-23
ANKRD32	-0.81922	2.67E-13	3.70E-12
DLGAP5	-0.81817	5.58E-25	1.68E-23
PIM1	-0.81724	6.60E-06	3.81E-05
ARFGAP2	-0.81718	1.77E-34	9.15E-33
WDR37	-0.81686	1.29E-25	4.04E-24
RP11-395I6.3	-0.81661	0.0026049	0.0090167
TMEM104	-0.81568	3.59E-11	4.06E-10
RP11-61A14.2	-0.81562	2.02E-07	1.47E-06
1-Mar	-0.81402	8.32E-34	4.11E-32
SHMT1	-0.81316	2.91E-38	1.85E-36
PI4K2B	-0.81292	3.46E-31	1.50E-29
RP11-134G8.7	-0.81283	0.0028837	0.0098881
ZNF467	-0.81166	7.89E-09	6.90E-08
COPG2	-0.80944	1.02E-10	1.09E-09
SAE1	-0.80892	1.82E-56	2.32E-54
MVD	-0.80889	2.67E-33	1.28E-31
TNFAIP2	-0.808	7.01E-08	5.44E-07
NRBP2	-0.80773	3.09E-28	1.12E-26
MMAB	-0.8068	1.49E-42	1.12E-40
GCK	-0.80672	3.57E-08	2.88E-07
LHX2	-0.8066	0.0036099	0.012018
RAB27A	-0.80657	1.47E-11	1.74E-10
INO80C	-0.80611	8.07E-08	6.21E-07
MORN4	-0.80532	9.78E-14	1.41E-12
CEBPG	-0.8052	2.24E-34	1.16E-32
BATF2	-0.80424	0.0010028	0.0038512
CCDC160	-0.80366	9.98E-05	0.00047896
SLC25A1P3	-0.80313	0.001892	0.00678
TMCC3	-0.80283	7.16E-11	7.83E-10
RP11-566E18.3	-0.80126	3.30E-08	2.68E-07
SLC35A4	-0.8009	4.24E-52	4.65E-50
TMEM160	-0.80064	1.46E-10	1.54E-09
RP11-468E2.9	-0.79773	0.0030675	0.010447
PNP	-0.79554	1.24E-26	4.11E-25
LINC00668	-0.7953	0.0034177	0.011454
CHAF1B	-0.79475	3.38E-17	6.32E-16
SLC7A5	-0.79427	9.68E-65	1.43E-62
RP11-343N15.5	-0.79359	1.84E-13	2.59E-12
SHKBP1	-0.79349	6.03E-29	2.24E-27

RFC5	-0.79097	7.58E-28	2.66E-26
ATG4D	-0.78994	1.19E-25	3.75E-24
RP11-156E6.1	-0.78896	1.23E-13	1.76E-12
IDI1	-0.78842	7.70E-38	4.76E-36
SLC7A10	-0.78789	0.0035979	0.011986
LINC00263	-0.78784	8.03E-10	7.84E-09
PPP2R3C	-0.78612	5.17E-13	6.99E-12
PFKFB4	-0.78535	5.68E-12	6.98E-11
CD151	-0.78531	1.86E-24	5.43E-23
KCTD14	-0.78489	0.0050845	0.016315
TRAF5	-0.78488	1.81E-15	3.04E-14
4-Sep	-0.78382	0.0035819	0.01194
COA3	-0.78296	3.76E-35	2.01E-33
ZNF674-AS1	-0.78295	8.38E-05	0.0004079
SCAMP3	-0.7828	5.49E-56	6.95E-54
GLT8D2	-0.78252	0.0046933	0.015164
IFNAR1	-0.7824	2.98E-33	1.42E-31
PTH1R	-0.78175	0.0022805	0.0079995
TBL1XR1	-0.78066	1.80E-45	1.58E-43
PLEKHO1	-0.7806	1.39E-11	1.64E-10
ACAD9	-0.77995	1.77E-34	9.15E-33
FANCA	-0.77961	5.76E-16	9.96E-15
GPT2	-0.77957	3.72E-43	2.90E-41
GTSE1	-0.7792	8.24E-30	3.24E-28
RAD51	-0.77866	3.38E-16	5.97E-15
SLC1A5	-0.77829	1.10E-41	8.01E-40
TMEM107	-0.77823	2.65E-16	4.71E-15
TGM2	-0.77816	0.0037007	0.012284
MT2A	-0.77801	6.15E-43	4.73E-41
CERS4	-0.77703	0.0039404	0.012993
NUB1	-0.77663	1.47E-32	6.83E-31
TMEM216	-0.77654	2.90E-11	3.31E-10
ZNF723	-0.77588	0.00072619	0.0028851
RABGGTB	-0.77582	6.27E-26	2.01E-24
PCSK7	-0.77555	7.41E-25	2.19E-23
RPF2	-0.77508	3.59E-27	1.23E-25
PRRC2C	-0.77439	3.41E-34	1.72E-32
CYP2C9	-0.77389	0.0014415	0.0053199
RBFOX2	-0.77273	1.51E-45	1.33E-43
RP11-167H9.4	-0.77257	0.0062039	0.019399
TRIM33	-0.77224	6.36E-31	2.71E-29
SAR1A	-0.77224	1.42E-43	1.13E-41
RFC2	-0.77206	5.38E-42	3.96E-40
DTX3L	-0.77105	2.15E-37	1.29E-35
TMEM179	-0.77045	6.10E-09	5.42E-08

RP11-677M14.	-0.77023	0.0032854	0.011066
CARM1	-0.76951	8.76E-27	2.93E-25
NDUFC2	-0.76823	6.36E-07	4.30E-06
MCM6	-0.76704	6.54E-38	4.09E-36
HMGN2P3	-0.7658	0.0022305	0.0078402
MPC2	-0.76539	1.74E-25	5.39E-24
ASB11	-0.76492	3.92E-05	0.00020231
FANCI	-0.76454	3.88E-26	1.26E-24
BLZF1	-0.76406	4.50E-28	1.61E-26
RPL39L	-0.76387	6.14E-17	1.13E-15
G3BP2	-0.76289	5.55E-47	5.12E-45
SPIN4	-0.7623	1.86E-17	3.55E-16
PRSS36	-0.76226	0.0063812	0.019885
RP11-571M6.1	-0.76219	0.0061499	0.019253
SYNE3	-0.76198	0.00050264	0.0020725
NRTN	-0.76128	0.0012343	0.0046445
YARS	-0.76123	2.80E-41	2.00E-39
SLC6A3	-0.761	0.00575	0.018158
PTK7	-0.76019	0.0037813	0.012519
SRI	-0.75977	8.12E-25	2.40E-23
MYD88	-0.75938	7.44E-26	2.39E-24
3-Sep	-0.75904	2.11E-18	4.38E-17
HOXB6	-0.7587	4.35E-06	2.59E-05
STX2	-0.75792	1.41E-12	1.82E-11
IRF2	-0.75785	3.62E-09	3.30E-08
FXYD4	-0.75711	0.0067025	0.020759
TRIP13	-0.75676	6.48E-28	2.29E-26
ARHGEF26-AS1	-0.75638	1.39E-06	8.92E-06
KIF18A	-0.75601	5.93E-15	9.53E-14
ABTB2	-0.75508	1.08E-16	1.96E-15
C15orf56	-0.75492	0.0067839	0.02099
IER3IP1	-0.75439	1.23E-10	1.30E-09
TTC13	-0.75327	4.08E-18	8.21E-17
EIF5A	-0.75231	3.07E-34	1.56E-32
KIF14	-0.75154	1.44E-19	3.25E-18
SMIM8	-0.75114	2.08E-07	1.51E-06
SHH	-0.75101	0.0067314	0.020842
SLC35F5	-0.75098	5.97E-19	1.29E-17
SLC6A9	-0.75053	4.83E-08	3.83E-07
SPC24	-0.7504	7.37E-19	1.58E-17
SYK	-0.75038	2.01E-30	8.09E-29
ZNF99	-0.75023	0.0055025	0.017469
AC004447.2	-0.7499	0.0024237	0.0084522
IFI44	-0.74919	0.0068976	0.021289
RP11-890B15.3	-0.74725	5.34E-05	0.00026951

RP1-39G22.7	-0.74651	1.19E-07	8.99E-07
RNASEH2A	-0.74637	1.06E-29	4.13E-28
GABRD	-0.7458	0.0081675	0.024738
TMEM194B	-0.74533	2.39E-09	2.22E-08
TK2	-0.74514	1.01E-15	1.72E-14
WDR4	-0.74493	1.09E-16	1.98E-15
BMP8B	-0.74466	3.70E-07	2.59E-06
ACSBG1	-0.74404	0.0050847	0.016315
PLK1	-0.74315	6.62E-44	5.38E-42
RAC3	-0.7421	1.64E-09	1.55E-08
RP11-167H9.6	-0.74142	0.0020456	0.0072532
AOC2	-0.74045	0.00026815	0.0011743
ISYNA1	-0.74045	1.15E-29	4.47E-28
RP11-122G18.!	-0.74034	1.60E-12	2.06E-11
AMN	-0.74026	4.69E-11	5.21E-10
RP11-436A20.4	-0.73899	0.0033107	0.011135
EFHD1	-0.73891	0.0020733	0.0073375
SKP2	-0.73863	6.67E-34	3.30E-32
PBK	-0.73853	1.86E-31	8.16E-30
CAMTA1	-0.738	4.69E-13	6.37E-12
CHMP1B	-0.73777	1.89E-20	4.50E-19
TPBGL	-0.73746	0.00882	0.026485
TTC28-AS1	-0.73679	1.23E-07	9.23E-07
AK7	-0.73629	5.36E-07	3.67E-06
EPS8L3	-0.73604	0.00025171	0.001109
RFC4	-0.7357	4.05E-23	1.12E-21
DNAJC27-AS1	-0.73499	1.76E-07	1.29E-06
MSH6	-0.73474	4.26E-25	1.29E-23
PORCN	-0.73465	9.85E-06	5.55E-05
RNF144A	-0.73425	7.41E-10	7.25E-09
STARD5	-0.73407	0.0070196	0.021637
SMPD3	-0.7339	3.15E-07	2.22E-06
TTC7A	-0.73385	1.18E-05	6.58E-05
LETMD1	-0.73379	7.35E-43	5.64E-41
MMGT1	-0.7337	7.12E-23	1.93E-21
ALDH1L1	-0.73328	0.0077394	0.023602
MGAT3	-0.73287	0.00082503	0.0032371
TCEA2	-0.73218	5.96E-15	9.57E-14
DHFRP1	-0.73109	0.0015636	0.0057239
CDKN2D	-0.7308	2.83E-11	3.24E-10
KRCC1	-0.7308	3.58E-16	6.31E-15
DOM3Z	-0.73079	1.88E-12	2.40E-11
PTPLB	-0.73076	1.74E-41	1.24E-39
DICER1-AS1	-0.73025	0.00023821	0.0010545
GNB1	-0.72985	7.61E-63	1.10E-60

BOLA1	-0.72952	1.04E-18	2.22E-17
IFITM1	-0.72905	0.0096429	0.028652
HOXB5	-0.72882	0.00057328	0.0023293
PRRT3-AS1	-0.72828	0.0023094	0.0080899
MARVELD1	-0.72767	8.85E-34	4.36E-32
CTD-2196E14.9	-0.72727	5.27E-07	3.61E-06
CDIPT	-0.72666	6.48E-28	2.29E-26
PEA15	-0.72641	4.39E-46	3.93E-44
NCAPH	-0.72638	1.66E-26	5.46E-25
SLC5A5	-0.72625	2.49E-10	2.57E-09
BCAS4	-0.72599	3.27E-06	1.98E-05
IFRD2	-0.72596	9.34E-33	4.36E-31
RP11-378A13.1	-0.7257	0.010072	0.02972
RARG	-0.72538	1.36E-26	4.48E-25
FIGF	-0.72487	0.0035562	0.011867
ARF1	-0.72469	7.18E-75	1.45E-72
MSANTD4	-0.72433	9.26E-11	9.98E-10
LRR1	-0.7241	3.30E-08	2.68E-07
TK1	-0.72401	5.36E-27	1.82E-25
CCT8	-0.72394	1.67E-41	1.20E-39
NFATC1	-0.72381	3.21E-05	0.00016787
RP11-89C3.4	-0.72287	0.0096915	0.028778
CTD-2523D13.2	-0.7228	0.0042072	0.01378
C2orf27A	-0.72176	2.61E-06	1.61E-05
KLHL1	-0.72167	0.010578	0.030997
TAP2	-0.72157	3.33E-12	4.16E-11
RP11-181C3.1	-0.71904	0.0011172	0.0042491
TPRG1L	-0.71821	4.49E-24	1.30E-22
MPV17L2	-0.71787	4.54E-18	9.10E-17
WDR5	-0.71711	1.33E-41	9.67E-40
KIF26A	-0.71661	0.0098273	0.029125
MCAT	-0.71561	3.09E-18	6.33E-17
NRM	-0.71548	1.01E-13	1.45E-12
KLHL11	-0.71471	1.79E-11	2.10E-10
RP11-760D2.5	-0.71463	0.0083769	0.02531
RP11-16P6.1	-0.7124	0.0022476	0.0078957
KCNH2	-0.71235	3.06E-10	3.11E-09
SLC25A23	-0.71225	2.10E-40	1.45E-38
ARHGAP33	-0.71217	1.18E-10	1.26E-09
DKC1	-0.71128	2.87E-41	2.04E-39
EPB41L4A-AS1	-0.71098	5.29E-15	8.54E-14
LARP4	-0.71077	1.82E-44	1.53E-42
PYGO2	-0.70976	5.96E-47	5.45E-45
ANKRD13A	-0.70968	9.68E-20	2.21E-18
IGLON5	-0.70965	0.0089437	0.026827

MCM5	-0.70941	6.42E-35	3.39E-33
BCAM	-0.70906	1.45E-14	2.24E-13
NEURL	-0.70895	6.13E-08	4.81E-07
CPPED1	-0.70846	2.65E-13	3.68E-12
NCBP2	-0.70788	7.12E-37	4.11E-35
LINC00338	-0.7066	3.26E-06	1.98E-05
SIGMAR1	-0.70632	1.44E-49	1.43E-47
RP11-480D4.3	-0.70616	4.72E-05	0.0002395
CACNA1G	-0.70612	0.0090187	0.027026
SERPINE2	-0.70595	6.57E-14	9.54E-13
RP11-66B24.2	-0.70566	0.010419	0.030596
DOK1	-0.70558	2.31E-05	0.00012301
CNFN	-0.70512	0.0011582	0.004389
SLC1A4	-0.70506	1.83E-13	2.56E-12
TMEM145	-0.70482	4.68E-09	4.20E-08
TMPRSS2	-0.7047	5.42E-14	7.93E-13
DHX58	-0.7042	0.0011175	0.0042496
ZFAND3	-0.70343	2.84E-33	1.36E-31
CSPG4	-0.70342	0.012669	0.036267
CELF5	-0.70243	0.0003055	0.0013183
PLSCR1	-0.7021	7.21E-16	1.24E-14
DANCR	-0.70047	5.31E-33	2.51E-31
RP11-187C18.4	-0.70024	0.0010059	0.0038604
PDLIM5	-0.69921	3.96E-34	1.99E-32
H2AFX	-0.69899	1.13E-25	3.59E-24
AKR1C2	-0.69869	3.75E-26	1.22E-24
ZWINT	-0.69834	3.01E-41	2.13E-39
SNX27	-0.69784	2.33E-45	2.03E-43
SREBF2	-0.69752	3.72E-40	2.53E-38
PTPN18	-0.69738	4.01E-28	1.44E-26
AC016629.8	-0.69701	0.00073625	0.0029176
ATXN10	-0.69696	3.67E-42	2.72E-40
MAPK3	-0.69564	7.42E-19	1.59E-17
CCBL2	-0.69552	3.31E-10	3.36E-09
C4orf27	-0.6955	1.01E-08	8.70E-08
C16orf74	-0.69467	0.006584	0.020436
TMEM184C	-0.69296	7.64E-23	2.07E-21
GCLM	-0.69295	1.19E-20	2.88E-19
OR51B5	-0.69287	0.014116	0.039852
SLC30A9	-0.69282	8.07E-37	4.64E-35
ACOT9	-0.69267	0.00034116	0.0014567
FAM72C	-0.69256	0.00040159	0.0016952
TLN2	-0.69049	0.014358	0.040431
NDUFB8	-0.68995	0.014202	0.040065
STRIP2	-0.68994	1.16E-05	6.43E-05

MROH6	-0.68953	1.48E-17	2.85E-16
CTD-2636A23.2	-0.68944	0.006337	0.019755
APEH	-0.68942	5.24E-50	5.34E-48
FAM73B	-0.68929	1.37E-17	2.65E-16
RP11-477D19.2	-0.68924	0.0042609	0.013943
RP11-622O11.1	-0.68917	0.0094987	0.02826
AC010504.2	-0.68874	0.01449	0.040753
SMC2	-0.68852	7.54E-23	2.05E-21
SLC25A22	-0.68771	8.77E-29	3.22E-27
SPTBN4	-0.68638	0.00042604	0.0017867
MYH15	-0.68629	1.87E-05	0.00010072
ZNF726	-0.68628	1.28E-06	8.27E-06
EMILIN3	-0.68602	0.012934	0.036928
B9D2	-0.68595	0.00061274	0.0024707
SLC31A1	-0.68583	1.02E-29	4.00E-28
OXSM	-0.68559	4.12E-20	9.60E-19
TRIM47	-0.68503	1.17E-09	1.12E-08
RP5-1120P11.1	-0.68468	0.014884	0.041704
EXO1	-0.68457	1.32E-17	2.55E-16
HAUS4	-0.68403	3.26E-07	2.30E-06
LINC00870	-0.68384	0.013604	0.038575
SLC47A1	-0.68326	1.45E-13	2.05E-12
SLC39A6	-0.68306	1.77E-42	1.33E-40
HFM1	-0.68209	0.012225	0.035132
RPL17P50	-0.68192	0.014272	0.040221
AURKAIP1	-0.68186	2.26E-25	6.95E-24
ILF3-AS1	-0.68182	2.10E-23	5.90E-22
RP11-159D12.9	-0.68165	9.01E-14	1.30E-12
BRCA1	-0.68132	2.76E-20	6.49E-19
HES4	-0.68125	4.68E-08	3.72E-07
LL22NC03-86G	-0.68061	0.0020822	0.0073662
KCNJ11	-0.68026	0.0001256	0.00059048
MCM3	-0.67946	3.33E-35	1.78E-33
RP11-43F13.3	-0.67941	0.00046993	0.0019532
WNT9A	-0.67915	0.00065961	0.0026397
TREX1	-0.67912	3.97E-18	8.01E-17
DNAL4	-0.67892	1.02E-11	1.22E-10
CENPI	-0.67883	6.38E-08	4.98E-07
CTC-429P9.3	-0.67865	0.00074789	0.0029612
GART	-0.6781	1.12E-46	1.01E-44
RP11-321N4.3	-0.67809	0.013291	0.037828
CTD-2245F17.2	-0.67787	0.01109	0.032287
KIF4A	-0.67787	6.74E-21	1.65E-19
GOLGA6L7P	-0.67759	0.013317	0.037877
TIFA	-0.67744	1.24E-08	1.05E-07

FAM49A	-0.67736	0.010012	0.029568
ZNF728	-0.67703	4.29E-07	2.98E-06
SEC23A	-0.67639	2.83E-37	1.68E-35
TMA7	-0.67635	3.59E-10	3.63E-09
CTD-3195I5.1	-0.67605	9.52E-08	7.27E-07
RPP25	-0.67604	2.95E-06	1.80E-05
CNTNAP2	-0.67589	2.52E-05	0.0001334
MLF1IP	-0.67502	7.68E-15	1.22E-13
DDX11-AS1	-0.67453	0.0068873	0.021264
TMEM219	-0.67275	2.71E-16	4.82E-15
HSDL1	-0.67247	2.58E-17	4.89E-16
EMG1	-0.67224	9.52E-05	0.00045858
COQ10A	-0.67133	1.06E-06	6.91E-06
PAQR4	-0.67122	1.38E-11	1.63E-10
TMEM136	-0.67104	9.59E-06	5.41E-05
DCAF4	-0.6709	7.02E-07	4.72E-06
POLA1	-0.67066	7.63E-13	1.01E-11
DDC	-0.67059	8.97E-18	1.76E-16
INCENP	-0.67055	6.18E-20	1.43E-18
RPRML	-0.67048	0.010616	0.031093
LRRC16B	-0.67005	0.016208	0.044868
C8orf82	-0.66968	1.40E-20	3.38E-19
HESX1	-0.66825	0.015131	0.042263
ACY3	-0.66804	0.0023016	0.0080689
ACOXL	-0.66684	0.00020112	0.00090277
PRSS27	-0.66679	0.018074	0.049357
SLC9A3R1	-0.66673	5.12E-11	5.67E-10
C14orf142	-0.66609	1.15E-07	8.71E-07
NUP210	-0.66604	7.02E-31	2.98E-29
C5orf15	-0.66603	5.91E-20	1.37E-18
HPDL	-0.66576	2.17E-07	1.57E-06
FADS1	-0.66576	9.83E-43	7.41E-41
HAUS2	-0.66553	1.36E-14	2.11E-13
NAPA	-0.66552	7.35E-38	4.58E-36
ABCC6P2	-0.66483	0.013321	0.037882
WDR16	-0.66466	0.0081999	0.024823
FLCN	-0.66457	2.10E-11	2.44E-10
NCMAP	-0.66416	0.00035569	0.0015131
PYCR1	-0.66405	2.81E-24	8.17E-23
KCTD17	-0.66344	4.36E-12	5.40E-11
CDC20	-0.66306	1.86E-44	1.55E-42
8-Sep	-0.6628	8.32E-33	3.90E-31
AC009133.12	-0.66241	0.0028724	0.0098548
INSIG1	-0.66186	1.81E-25	5.60E-24
AC006538.1	-0.66159	0.013943	0.039446

SLC30A8	-0.66089	0.018221	0.049659
ALDH4A1	-0.66041	7.82E-17	1.43E-15
RP5-1057J7.7	-0.66033	0.0066401	0.0206
AC005943.5	-0.6602	1.41E-07	1.05E-06
C5orf49	-0.66015	0.018315	0.049879
KB-1507C5.2	-0.65991	0.00092566	0.0035903
BIN1	-0.6592	0.010994	0.032072
RP11-46H11.3	-0.65919	0.01809	0.049386
ENO2	-0.65869	1.63E-37	9.88E-36
SULT1E1	-0.65857	0.0095328	0.028352
HNF4G	-0.65793	1.59E-19	3.57E-18
TRAIP	-0.65789	2.49E-09	2.30E-08
TMEM51	-0.6578	4.99E-07	3.43E-06
MFI2	-0.65777	2.97E-10	3.03E-09
RP11-326I11.3	-0.65712	0.0015517	0.0056859
TRIB3	-0.65658	1.51E-22	4.03E-21
POLR2I	-0.65656	0.0025803	0.0089416
KCNT1	-0.65643	0.0011475	0.0043545
RAB31	-0.65596	1.29E-05	7.14E-05
FEM1AP2	-0.65519	0.011528	0.033417
PIP5K1A	-0.65519	2.51E-45	2.18E-43
RP1-239B22.5	-0.65507	2.28E-05	0.00012141
LMNB2	-0.65502	7.61E-38	4.73E-36
PDE4DIP	-0.65491	1.04E-29	4.06E-28
ST6GALNAC5	-0.65445	0.00016727	0.00076258
MCM2	-0.6539	2.29E-43	1.81E-41
DMC1	-0.65371	7.12E-05	0.0003518
KIF22	-0.65368	5.81E-29	2.16E-27
DIAPH3	-0.65366	1.36E-17	2.63E-16
ELOVL6	-0.65356	9.78E-37	5.59E-35
LETM1	-0.65282	1.18E-31	5.27E-30
POU6F1	-0.65232	4.55E-13	6.19E-12
UHRF1	-0.65211	3.05E-17	5.74E-16
RP11-598P20.3	-0.65185	7.67E-09	6.71E-08
ENTPD3-AS1	-0.65151	3.83E-05	0.00019746
TLR3	-0.65133	0.010098	0.029775
FAM225B	-0.6505	0.017434	0.047893
MCM4	-0.65033	9.14E-31	3.84E-29
VILL	-0.65012	0.016906	0.046569
DCLRE1B	-0.64958	2.38E-11	2.75E-10
POLRMT	-0.6491	1.12E-19	2.56E-18
MOSPD2	-0.64818	2.50E-08	2.05E-07
GSDMD	-0.64815	6.82E-22	1.74E-20
AL645728.1	-0.64773	0.017803	0.048746
DHX37	-0.64763	4.57E-21	1.13E-19

DPP10	-0.64728	0.00078322	0.0030899
VWA5B2	-0.64675	3.65E-12	4.54E-11
SLCO2A1	-0.64653	0.0099919	0.029524
PRODH	-0.64639	0.0011931	0.0045019
STAU1	-0.64584	1.55E-37	9.46E-36
TRIM60P18	-0.6451	0.003959	0.013049
ZNF789	-0.64497	2.93E-19	6.45E-18
DPYSL5	-0.64461	0.00028741	0.0012489
AC034193.5	-0.64403	7.44E-05	0.00036635
ADM2	-0.6439	4.15E-22	1.08E-20
PRR5L	-0.64369	0.0074718	0.022902
HNRNPUL2	-0.6423	8.38E-15	1.33E-13
RP11-600F24.7	-0.64229	2.22E-05	0.00011854
APLP1	-0.64204	3.46E-15	5.68E-14
ARFIP2	-0.64176	3.46E-25	1.05E-23
GCHFR	-0.64167	2.83E-19	6.23E-18
RP11-16E18.4	-0.64142	0.0047187	0.015238
DEGS2	-0.64081	0.0076755	0.023456
KRT19	-0.64029	3.28E-06	1.98E-05
COPS7A	-0.64016	3.34E-32	1.53E-30
NSUN5	-0.63981	4.71E-19	1.03E-17
GLB1L2	-0.63913	1.73E-18	3.62E-17
C1orf112	-0.63864	9.25E-11	9.98E-10
SPR	-0.6386	1.46E-29	5.64E-28
NKD2	-0.63833	0.00011586	0.00054948
AGT	-0.6375	0.0098746	0.029233
POLR2M	-0.6364	3.29E-18	6.73E-17
KLF11	-0.63614	5.51E-08	4.34E-07
TINCR	-0.63576	2.92E-06	1.78E-05
AC004463.6	-0.63574	0.01724	0.047406
CDC25A	-0.63488	4.36E-14	6.42E-13
RP3-341D10.4	-0.63447	0.0052641	0.016804
RP11-496H1.1	-0.63428	0.010325	0.030354
CTC-429P9.2	-0.63321	0.01053	0.030887
DTL	-0.63316	2.98E-18	6.12E-17
BRD8	-0.63312	1.38E-19	3.13E-18
ZNF98	-0.63303	1.99E-05	0.00010696
PALM	-0.63215	0.0061245	0.019179
ASPHD1	-0.63196	8.01E-14	1.16E-12
RP11-389G6.3	-0.63102	0.0028849	0.0098905
MTFR2	-0.63012	2.68E-06	1.65E-05
ALDH16A1	-0.62934	3.40E-11	3.85E-10
FBXO2	-0.62926	0.0019472	0.0069466
FAM72D	-0.62906	7.43E-11	8.09E-10
TRAPPC9	-0.62855	8.29E-16	1.42E-14

CTD-2525I3.3	-0.62853	0.017358	0.047708
AC130352.1	-0.62808	0.002324	0.0081382
LYSMD2	-0.62783	9.26E-11	9.98E-10
RP11-660L16.2	-0.62732	0.0015108	0.0055491
HIST1H2AE	-0.62697	0.01533	0.042767
RANGAP1	-0.62647	2.99E-36	1.68E-34
NIF3L1	-0.62572	4.27E-13	5.84E-12
CSPG5	-0.62568	2.69E-06	1.65E-05
APRT	-0.62537	5.63E-28	2.01E-26
STMN3	-0.62504	4.32E-27	1.47E-25
LIN9	-0.6245	4.89E-16	8.49E-15
STAT5A	-0.62446	2.21E-08	1.84E-07
KLK1	-0.62426	9.04E-13	1.19E-11
PCK2	-0.62421	1.55E-18	3.26E-17
NGRN	-0.62414	4.60E-09	4.14E-08
DHFR	-0.62397	1.60E-29	6.15E-28
SWAP70	-0.62255	2.42E-10	2.50E-09
UCP1	-0.62242	0.014209	0.040078
NAT14	-0.62234	1.74E-07	1.28E-06
PRC1	-0.622	3.36E-29	1.27E-27
RP11-849H4.2	-0.62199	0.002963	0.010127
MMEL1	-0.6217	0.013399	0.038059
RPL7L1	-0.62111	4.46E-40	3.01E-38
CHTOP	-0.6208	7.45E-24	2.14E-22
KIF2C	-0.62028	7.05E-19	1.52E-17
ITPRIPL1	-0.62026	0.00058422	0.0023685
RAB23	-0.62	4.23E-09	3.83E-08
BCL2L13	-0.61972	1.27E-30	5.25E-29
DONSON	-0.61876	7.34E-09	6.45E-08
ADAM1A	-0.61857	7.44E-08	5.75E-07
POPDC2	-0.61768	0.01545	0.043038
OSMR	-0.61719	0.00012764	0.00059906
GPAT2	-0.61684	2.71E-14	4.07E-13
CKB	-0.61657	7.60E-27	2.55E-25
METTL21A	-0.61637	2.40E-08	1.97E-07
SLC3A2	-0.61622	1.38E-43	1.10E-41
C18orf54	-0.61621	1.42E-08	1.20E-07
C2CD2	-0.61585	1.17E-11	1.40E-10
UMPS	-0.61569	5.79E-25	1.74E-23
POP7	-0.61562	6.12E-16	1.06E-14
ZNF665	-0.61535	0.0019603	0.0069892
B3GNTL1	-0.61519	1.02E-05	5.72E-05
PSMC3IP	-0.61364	1.14E-05	6.36E-05
RP11-44N21.1	-0.61359	0.0083937	0.025357
SNAP29	-0.61319	2.04E-15	3.41E-14

HMG20B	-0.61307	3.63E-29	1.37E-27
ZCCHC10	-0.61264	3.75E-08	3.01E-07
PPP1R16A	-0.6126	2.26E-14	3.41E-13
UBE2K	-0.61204	1.20E-32	5.59E-31
SNHG10	-0.61162	0.0031279	0.010612
CCDC85B	-0.61138	1.06E-11	1.27E-10
CHMP4B	-0.61078	3.19E-18	6.54E-17
DGUOK	-0.61072	5.39E-10	5.37E-09
PHF11	-0.61069	0.0006688	0.0026736
MAP3K1	-0.61008	5.33E-11	5.89E-10
MAD2L1	-0.61008	1.01E-20	2.45E-19
CCDC88B	-0.60998	0.0044855	0.014586
NRXN2	-0.60966	1.27E-06	8.21E-06
ALYREF	-0.60965	5.91E-24	1.70E-22
ZBTB38	-0.60955	1.71E-11	2.01E-10
FAM81A	-0.60934	9.01E-08	6.90E-07
DNM1P35	-0.60854	0.013855	0.039222
CENPL	-0.6079	6.06E-14	8.83E-13
EMC10	-0.60717	6.47E-23	1.77E-21
PRSS16	-0.60706	2.54E-07	1.82E-06
TM4SF5	-0.60635	0.00070341	0.002803
SMIM19	-0.60567	5.79E-14	8.46E-13
TMEM88	-0.60551	7.59E-07	5.07E-06
TIMM10B	-0.60532	8.89E-11	9.60E-10
H2AFV	-0.60499	1.45E-36	8.21E-35
PNCK	-0.60482	4.06E-17	7.55E-16
ORC1	-0.60448	7.27E-11	7.93E-10
RBM8A	-0.60385	7.44E-20	1.71E-18
ALDH1L2	-0.60369	1.98E-28	7.24E-27
MTRR	-0.60337	2.03E-17	3.86E-16
NR3C2	-0.60281	8.48E-14	1.23E-12
CDC45	-0.60241	1.11E-13	1.59E-12
TEF	-0.60238	1.19E-08	1.02E-07
ATP6AP1L	-0.60237	0.00013324	0.00062283
BBS2	-0.60224	3.31E-17	6.21E-16
P2RX6	-0.60189	2.26E-05	0.00012061
SLC35G1	-0.60184	3.60E-08	2.91E-07
HAPLN2	-0.60107	0.016175	0.04479
BAMBI	-0.60087	1.21E-07	9.11E-07
CLEC11A	-0.59952	0.0031917	0.010803
PNMA1	-0.59911	9.20E-13	1.21E-11
CAPG	-0.59895	1.90E-08	1.59E-07
STYX	-0.59867	1.30E-18	2.75E-17
CDC7	-0.59863	2.29E-11	2.65E-10
KCTD15	-0.59841	1.92E-16	3.43E-15

CCNB1	-0.59803	1.70E-25	5.30E-24
TECPR1	-0.598	2.00E-13	2.80E-12
GYG2	-0.59793	0.014893	0.041724
ABCF3	-0.59785	1.55E-22	4.15E-21
ASNS	-0.59755	3.51E-24	1.02E-22
AL807752.1	-0.59651	0.0042311	0.013853
MCM7	-0.59642	5.69E-53	6.39E-51
FEN1	-0.59609	1.25E-24	3.64E-23
TONSL	-0.59561	5.80E-17	1.07E-15
RIMKLA	-0.59546	0.006228	0.019464
XRCC3	-0.59531	1.65E-09	1.55E-08
LRRC45	-0.59522	4.47E-12	5.53E-11
SLC43A3	-0.5949	8.31E-09	7.24E-08
IMPA2	-0.59465	3.84E-10	3.87E-09
SRSF12	-0.59431	0.00082383	0.003233
PAGR1	-0.59428	4.20E-11	4.70E-10
FBXW11	-0.59385	9.43E-26	3.01E-24
GALNT6	-0.59381	0.011588	0.033559
TMC6	-0.59381	6.39E-07	4.32E-06
NCAPG	-0.59348	2.52E-16	4.49E-15
C3orf14	-0.593	8.78E-05	0.00042565
PPP2R4	-0.5927	3.06E-28	1.11E-26
TMEM200B	-0.59231	0.00021525	0.00096158
FANCE	-0.59228	2.59E-05	0.0001365
NEURL3	-0.59199	0.009497	0.028259
KANK2	-0.5916	1.50E-16	2.70E-15
MEIS3	-0.59117	0.013373	0.037991
PFKL	-0.59081	7.94E-20	1.82E-18
ALDH3B1	-0.59074	8.94E-09	7.76E-08
FKBP4	-0.59049	5.60E-36	3.07E-34
RP11-115C21.2	-0.59037	1.23E-05	6.82E-05
CARS	-0.59018	2.97E-17	5.60E-16
TMEM243	-0.58995	0.0006097	0.0024606
PLGRKT	-0.58976	1.82E-11	2.13E-10
CYBB	-0.58946	0.0013245	0.004941
MFF	-0.58944	3.59E-19	7.87E-18
DGCR5	-0.58938	0.00033718	0.0014407
AIF1L	-0.58926	5.09E-26	1.65E-24
GPR153	-0.58911	0.0012502	0.0046976
LINGO3	-0.58879	0.0055531	0.017609
TOR4A	-0.58836	3.40E-07	2.39E-06
SRSF4	-0.58806	7.09E-21	1.74E-19
RIBC2	-0.58786	0.00015082	0.00069524
ANKRD30B	-0.58776	0.007652	0.023397
TSPAN17	-0.58768	3.00E-16	5.30E-15

HNF4A	-0.58745	0.0078352	0.023851
BORA	-0.58644	2.42E-08	1.99E-07
COMMD10	-0.58594	2.79E-07	1.99E-06
AC066593.1	-0.58562	6.82E-06	3.93E-05
POLD1	-0.5846	1.39E-16	2.50E-15
HMGCR	-0.58433	5.09E-37	2.96E-35
CLIC3	-0.58404	0.00052129	0.0021385
ZNF837	-0.58359	7.19E-05	0.00035484
ECT2	-0.58339	1.19E-25	3.76E-24
KIAA1462	-0.58332	0.0027984	0.0096222
CTD-2292M16.	-0.58323	0.017664	0.048403
YBX2	-0.58317	4.48E-11	4.99E-10
CTSL2	-0.58292	0.0042042	0.013772
RP11-760H22.2	-0.58285	0.0052115	0.016659
ACAT2	-0.58271	2.63E-17	4.97E-16
RP11-342K6.4	-0.58257	0.013562	0.038476
MRPL2	-0.58254	3.12E-11	3.56E-10
SYP	-0.58251	5.93E-11	6.54E-10
METTL1	-0.58198	1.71E-13	2.41E-12
RPS6KA4	-0.58179	6.23E-13	8.34E-12
MLH1	-0.58082	1.30E-23	3.67E-22
BUB1B	-0.58076	1.48E-20	3.56E-19
VRK1	-0.58069	1.02E-07	7.77E-07
ILVBL	-0.57963	6.19E-14	9.01E-13
NADKD1	-0.57963	5.42E-18	1.08E-16
RP11-73B2.6	-0.57931	0.016759	0.046222
DCAKD	-0.57919	1.97E-16	3.53E-15
SMARCD2	-0.57905	5.11E-42	3.78E-40
SWSAP1	-0.57884	0.011322	0.03288
RCOR2	-0.57837	2.39E-07	1.71E-06
RNFT2	-0.57809	5.43E-13	7.32E-12
GGCX	-0.57801	5.55E-09	4.95E-08
RP11-392A22.2	-0.57789	4.41E-05	0.00022566
EIF2B5	-0.57768	3.94E-21	9.77E-20
STOX1	-0.57731	0.012455	0.035737
BRI3BP	-0.57717	7.68E-36	4.18E-34
RABGGTA	-0.57699	3.55E-14	5.28E-13
CD6	-0.57685	8.03E-05	0.00039279
ACOT1	-0.57641	0.010597	0.031045
NXT2	-0.57624	1.72E-07	1.27E-06
C1orf192	-0.57623	1.01E-11	1.21E-10
TBCD	-0.57605	1.50E-18	3.16E-17
XRCC2	-0.57599	6.66E-13	8.87E-12
E2F8	-0.57576	1.50E-05	8.21E-05
POLR3A	-0.57553	9.76E-21	2.37E-19

FAM72B	-0.57482	2.23E-07	1.61E-06
FANCG	-0.57459	1.65E-16	2.96E-15
HOXB4	-0.57452	0.0013934	0.0051668
C16orf55	-0.57439	2.13E-05	0.00011391
ADH1B	-0.57415	0.0044883	0.014593
C18orf8	-0.57409	1.22E-09	1.17E-08
SOCS7	-0.57407	3.13E-07	2.21E-06
RP11-195E2.4	-0.57379	0.00033625	0.0014377
MCM8	-0.57352	8.18E-16	1.40E-14
MLK4	-0.57347	2.54E-10	2.62E-09
COPG1	-0.57341	2.26E-34	1.16E-32
TCTA	-0.57273	1.34E-25	4.20E-24
PIK3R5	-0.57242	0.011612	0.033605
AC017116.8	-0.57231	1.18E-05	6.58E-05
WDR34	-0.57217	5.64E-18	1.12E-16
RP11-498E2.7	-0.57213	0.005715	0.018058
SPATA6	-0.5721	0.0033947	0.011383
ZNF653	-0.57207	0.00087569	0.0034173
RSAD1	-0.57188	1.70E-19	3.81E-18
KCNC2	-0.57143	0.00062524	0.0025135
PC	-0.57138	6.60E-15	1.05E-13
KCNMA1	-0.57094	0.00093068	0.0036053
SPTBN5	-0.57085	0.0046056	0.014919
RP11-73M18.8	-0.57084	0.0071947	0.022136
PARP12	-0.57037	1.88E-08	1.57E-07
NRCAM	-0.57024	3.83E-08	3.07E-07
TRMT2B	-0.56993	3.28E-08	2.66E-07
LPCAT1	-0.56921	3.60E-23	9.96E-22
ZDHHC18	-0.56901	2.63E-17	4.97E-16
DAAM2	-0.56843	0.0063368	0.019755
AFAP1L2	-0.56831	0.00047687	0.001979
H2AFZ	-0.56826	2.16E-23	6.03E-22
LMAN1	-0.56808	3.74E-26	1.22E-24
ABCA7	-0.56805	1.79E-12	2.29E-11
TBXA2R	-0.56733	0.0020482	0.0072595
MRPL41	-0.56698	5.13E-13	6.94E-12
LGALS4	-0.5669	0.00027176	0.001187
MERTK	-0.56625	0.00037003	0.0015706
ARID3A	-0.56622	4.11E-07	2.86E-06
RAD54L	-0.56595	9.19E-06	5.19E-05
DZIP3	-0.5651	4.69E-17	8.68E-16
ASAP3	-0.565	7.53E-08	5.82E-07
EPS8	-0.56488	2.04E-30	8.18E-29
PTMAP2	-0.5647	2.41E-06	1.49E-05
FARP2	-0.5643	8.54E-08	6.55E-07

KCNK5	-0.56414	2.55E-05	0.00013468
RFX2	-0.56387	0.0037475	0.012416
RP11-21N3.1	-0.56378	3.75E-10	3.78E-09
WDR89	-0.5632	1.36E-07	1.02E-06
MELK	-0.56269	1.18E-12	1.55E-11
NASP	-0.56245	7.02E-25	2.08E-23
ARV1	-0.56224	3.59E-07	2.52E-06
KIF1C	-0.56212	5.58E-26	1.80E-24
NELFCD	-0.56182	3.49E-29	1.32E-27
GINS3	-0.56151	1.10E-11	1.31E-10
TATDN3	-0.56141	3.58E-16	6.31E-15
C1orf229	-0.56123	0.006364	0.019836
RAD54B	-0.56076	6.90E-06	3.96E-05
PKN1	-0.5607	7.95E-20	1.82E-18
ARHGAP15	-0.56031	0.00979	0.029024
ARHGEF6	-0.56011	3.52E-06	2.12E-05
RP11-247I13.7	-0.55974	0.012877	0.036788
AP1G1	-0.55963	1.51E-26	4.98E-25
ATP5J2	-0.55899	0.0118	0.034062
VPS33A	-0.55899	1.94E-08	1.62E-07
PRMT6	-0.55885	8.80E-20	2.02E-18
GEM	-0.55869	0.0079616	0.024201
NCR3LG1	-0.55818	1.40E-10	1.49E-09
ULK3	-0.55814	3.58E-17	6.67E-16
TUBE1	-0.55812	5.49E-07	3.75E-06
DENND1A	-0.55781	1.19E-11	1.42E-10
KBTBD6	-0.5575	5.13E-19	1.12E-17
CCDC135	-0.5566	0.0087126	0.026188
DOK4	-0.55656	6.04E-15	9.68E-14
CEP135	-0.55459	9.64E-09	8.33E-08
SNX4	-0.55438	6.69E-25	2.00E-23
RP11-568K15.1	-0.55425	2.57E-10	2.65E-09
C7orf63	-0.55354	1.71E-05	9.25E-05
CLIP2	-0.55342	3.02E-10	3.07E-09
HOXA3	-0.55303	0.0031045	0.010554
IFT27	-0.55302	1.26E-08	1.07E-07
WDHD1	-0.55243	1.26E-11	1.50E-10
DSN1	-0.5523	7.72E-12	9.37E-11
AC133528.2	-0.5521	0.0019934	0.0070885
RFC3	-0.55196	1.80E-15	3.03E-14
RNF187	-0.5518	1.81E-29	6.94E-28
UBE2G1	-0.55154	3.92E-24	1.13E-22
LLGL1	-0.55046	3.17E-11	3.61E-10
AC069282.6	-0.55037	0.0030513	0.010406
SLC25A4	-0.54995	2.46E-19	5.47E-18

SLC24A6	-0.54974	0.001137	0.0043167
FDFT1	-0.54969	2.34E-43	1.85E-41
CNTROB	-0.54919	7.27E-16	1.25E-14
TRAPPC4	-0.54852	1.50E-17	2.89E-16
TRIM45	-0.54832	0.0029392	0.010053
VAMP2	-0.54829	5.59E-16	9.68E-15
TATDN1	-0.54826	1.94E-09	1.81E-08
ZBTB12	-0.54806	0.00044485	0.0018564
R3HDM1	-0.54746	5.63E-13	7.60E-12
TNS1	-0.54722	6.04E-06	3.52E-05
TMEFF2	-0.54679	6.74E-44	5.46E-42
LRRC26	-0.54673	0.0055932	0.017724
TMEM30A	-0.54662	5.05E-20	1.17E-18
RNLS	-0.54658	0.0016979	0.0061707
EEF1E1	-0.54654	0.002325	0.0081401
MAPK8IP2	-0.54652	3.24E-19	7.12E-18
ALDH3B2	-0.54646	5.81E-05	0.00029127
CLSPN	-0.54644	2.78E-07	1.98E-06
ARL5A	-0.54637	2.13E-14	3.23E-13
RAD18	-0.54608	8.12E-12	9.85E-11
BARD1	-0.54605	2.56E-09	2.36E-08
ZNF736	-0.54556	3.02E-07	2.14E-06
MTCH1	-0.54549	1.48E-27	5.15E-26
FLG	-0.54546	1.28E-07	9.61E-07
RP11-396C23.2	-0.54515	0.0013225	0.0049344
ALDOC	-0.54511	4.15E-11	4.66E-10
TBL1Y	-0.54481	0.010003	0.029546
PHGDH	-0.54479	1.78E-19	3.97E-18
EXOSC8	-0.54459	7.18E-12	8.74E-11
KIFC1	-0.54437	3.50E-18	7.14E-17
ZNF257	-0.54427	6.29E-06	3.65E-05
SPINT1	-0.54254	2.00E-30	8.05E-29
RGS14	-0.54245	0.00070483	0.002808
RP11-181G12.2	-0.54185	0.007138	0.021973
MCM10	-0.54178	1.27E-15	2.15E-14
TIPIN	-0.54169	5.80E-09	5.16E-08
RP11-18I14.10	-0.54155	8.42E-09	7.32E-08
RASA4CP	-0.5412	2.93E-08	2.39E-07
BRCA2	-0.54061	1.32E-07	9.90E-07
AAED1	-0.54039	0.0015742	0.0057571
AUH	-0.53973	5.13E-09	4.59E-08
C5orf54	-0.5394	0.00056659	0.0023056
CPEB4	-0.53938	1.74E-13	2.45E-12
C1orf21	-0.53928	6.17E-09	5.47E-08
MTBP	-0.53918	8.24E-09	7.18E-08

PRTFDC1	-0.53889	6.57E-05	0.00032674
SLC11A1	-0.53821	0.016876	0.046501
PMF1	-0.5381	6.11E-07	4.14E-06
MIS18A	-0.53809	9.92E-13	1.30E-11
TFG	-0.53804	3.48E-32	1.58E-30
GABRQ	-0.53797	3.20E-14	4.77E-13
GEMIN2	-0.53774	2.64E-08	2.16E-07
LYSMD1	-0.53745	1.84E-09	1.73E-08
CPS1	-0.5374	0.00013113	0.00061422
SCO2	-0.53736	1.76E-13	2.48E-12
IMPA1	-0.53702	6.33E-12	7.73E-11
VMA21	-0.53637	2.29E-13	3.19E-12
PSMA2	-0.53612	0.0086375	0.025988
SKA3	-0.536	3.86E-08	3.09E-07
CIAPIN1	-0.53598	9.64E-22	2.46E-20
HAGHL	-0.53572	5.47E-10	5.43E-09
SLC22A18	-0.53564	6.92E-06	3.98E-05
RP11-132A1.4	-0.53561	0.0017413	0.0063125
TMEM192	-0.53521	2.80E-16	4.96E-15
RP11-983P16.4	-0.53516	1.26E-09	1.20E-08
FAM3A	-0.53495	4.05E-12	5.03E-11
HES6	-0.53477	0.0054091	0.017193
AC016683.6	-0.53466	1.14E-14	1.79E-13
MYBPC1	-0.53464	6.60E-06	3.81E-05
TCIRG1	-0.53432	2.06E-07	1.50E-06
DDN	-0.5343	2.96E-16	5.23E-15
RP5-1086D14.6	-0.5334	0.00023438	0.0010386
MCCC2	-0.53328	2.42E-40	1.66E-38
GARNL3	-0.53307	0.00091673	0.0035623
LRRC37A16P	-0.53293	8.30E-16	1.42E-14
PLEKHG2	-0.53292	2.05E-06	1.28E-05
ERCC2	-0.532	4.31E-15	7.02E-14
SUMO3	-0.53191	1.40E-23	3.94E-22
ZNF729	-0.53172	0.0017511	0.0063392
MARK4	-0.53168	6.79E-10	6.68E-09
NAPB	-0.53147	2.31E-08	1.91E-07
C14orf119	-0.53129	7.98E-07	5.31E-06
CECR6	-0.5312	0.00010387	0.00049704
C9orf72	-0.53078	1.21E-08	1.03E-07
GP1BA	-0.52956	0.0030598	0.01043
POLE2	-0.52933	2.03E-05	0.00010869
STEAP1	-0.52902	6.70E-16	1.16E-14
RRNAD1	-0.52898	1.34E-11	1.58E-10
FAM72A	-0.52877	1.90E-07	1.38E-06
GPRIN1	-0.52877	1.88E-09	1.76E-08

NEK4	-0.52834	3.03E-15	5.00E-14
H6PD	-0.52834	1.90E-17	3.62E-16
C17orf53	-0.52821	8.21E-05	0.00040065
LDHAP4	-0.52811	0.0039706	0.013085
NUDT15	-0.52804	3.10E-09	2.84E-08
RP4-740C4.6	-0.5279	0.00051188	0.0021041
RP5-1121A15.1	-0.52764	0.00081374	0.0031981
KLF2	-0.52751	0.0018676	0.0066999
USP12	-0.52745	7.83E-13	1.03E-11
MBOAT2	-0.52724	1.16E-07	8.77E-07
UPP1	-0.52679	0.00051631	0.0021214
SUCLG1	-0.52676	1.69E-25	5.27E-24
APBB2	-0.52669	4.45E-08	3.54E-07
ACOT2	-0.5266	1.02E-10	1.09E-09
PDE8B	-0.52506	0.0059425	0.018691
AKAP5	-0.52476	0.008293	0.025089
FBXO17	-0.52457	0.00040837	0.0017215
C5orf34	-0.52421	0.00044225	0.0018468
KHDC1	-0.52363	0.016787	0.046285
GPNMB	-0.52355	0.009871	0.029232
RP11-163N6.2	-0.52349	0.016689	0.046062
COPS4	-0.52342	2.57E-11	2.96E-10
WDR67	-0.52282	5.29E-08	4.17E-07
CDHR5	-0.52277	0.014237	0.040132
KIF12	-0.52179	2.54E-12	3.21E-11
EFCAB11	-0.52162	5.71E-07	3.89E-06
MBLAC2	-0.52122	1.72E-05	9.29E-05
VPS26B	-0.52077	5.07E-15	8.20E-14
BAIAP3	-0.5201	6.93E-06	3.98E-05
TNNT1	-0.51994	2.14E-10	2.23E-09
NUPR1	-0.51981	2.97E-21	7.42E-20
SLC39A3	-0.51967	1.97E-08	1.64E-07
PROB1	-0.51963	0.00019975	0.00089687
CTC-444N24.11	-0.51857	3.19E-05	0.00016671
TUBB4B	-0.51851	4.26E-31	1.84E-29
SLC7A2	-0.51821	4.97E-20	1.15E-18
LEMD3	-0.51792	5.37E-17	9.93E-16
CUL7	-0.5179	2.63E-15	4.35E-14
MYLK	-0.51752	2.05E-07	1.49E-06
HOXB-AS5	-0.51709	2.69E-05	0.00014176
RAPGEFL1	-0.51708	0.0015215	0.005583
THNSL2	-0.5166	1.42E-08	1.20E-07
CISD1	-0.51652	5.87E-14	8.57E-13
NIP7	-0.5163	1.11E-12	1.45E-11
BSPRY	-0.51619	1.10E-08	9.48E-08

CCNA2	-0.51562	1.19E-21	3.02E-20
DCTPP1	-0.51545	1.04E-14	1.63E-13
MGLL	-0.51522	1.42E-07	1.06E-06
AFAP1L1	-0.515	0.0032671	0.011018
C11orf68	-0.51492	9.62E-08	7.34E-07
LGALSL	-0.51432	5.49E-17	1.01E-15
TMED8	-0.51429	8.14E-14	1.18E-12
E2F2	-0.514	5.67E-15	9.13E-14
ACOT7	-0.51316	6.96E-13	9.23E-12
PIGV	-0.51283	1.55E-10	1.63E-09
ATRIP	-0.51267	4.24E-06	2.53E-05
RP4-594I10.3	-0.51263	1.19E-05	6.59E-05
7-Mar	-0.51244	7.59E-11	8.25E-10
ZFYVE28	-0.51242	0.0018635	0.0066876
GSTZ1	-0.51162	1.99E-11	2.32E-10
GDI1	-0.51142	8.95E-23	2.41E-21
PER2	-0.511	3.35E-09	3.06E-08
PTP4A2	-0.51062	7.70E-26	2.46E-24
PRPF40A	-0.51059	2.12E-17	4.03E-16
TMBIM4	-0.51052	2.21E-06	1.37E-05
C11orf71	-0.50936	0.00057768	0.0023461
TMEM180	-0.50866	1.80E-10	1.88E-09
PFKFB3	-0.5085	8.14E-12	9.87E-11
SLCO4A1	-0.50835	0.00010783	0.00051347
ABCC6P1	-0.50822	1.61E-07	1.19E-06
CNTFR	-0.50819	0.00063814	0.0025604
ORC6	-0.5081	6.39E-13	8.54E-12
ERCC6L	-0.50783	1.14E-05	6.35E-05
ZDHHC13	-0.50751	5.04E-09	4.51E-08
CGNL1	-0.50739	0.0081515	0.024699
LINC00094	-0.50708	1.28E-08	1.09E-07
PPP3R1	-0.50698	7.87E-17	1.44E-15
EFNB3	-0.50697	0.0031263	0.010611
KNOP1	-0.50685	1.11E-07	8.43E-07
FN3KRP	-0.50671	1.25E-10	1.33E-09
FDPS	-0.5066	0.0005846	0.0023696
FAH	-0.50651	2.93E-12	3.67E-11
COX5B	-0.5062	2.16E-20	5.13E-19
HMMR	-0.50578	9.42E-16	1.61E-14
HPS5	-0.50546	1.39E-08	1.17E-07
POMZP3	-0.50517	0.00016655	0.00075968
TBL3	-0.50506	2.13E-10	2.21E-09
N6AMT2	-0.50504	4.32E-05	0.00022139
RPS10P7	-0.50501	0.00029159	0.0012644
RAPH1	-0.50501	3.69E-08	2.97E-07

UNC13A	-0.50501	3.88E-20	9.06E-19
MAN1C1	-0.50496	0.00044947	0.001874
RAD51D	-0.50488	0.00014729	0.00068017
CTD-2037K23.2	-0.50479	0.0037203	0.012337
PARP16	-0.50438	2.29E-05	0.00012183
NDUFC1	-0.50389	1.38E-11	1.63E-10
DPM2	-0.5038	2.01E-13	2.81E-12
GNB1L	-0.50375	0.0018999	0.0068051
SLC46A3	-0.50369	0.00068241	0.0027222
MAGEF1	-0.50301	4.11E-20	9.58E-19
RPUSD1	-0.50289	1.21E-11	1.44E-10
ARHGEF25	-0.50284	0.0001548	0.00071221
GSTM4	-0.50178	1.86E-10	1.94E-09
MB	-0.50176	2.08E-08	1.73E-07
ATP6V0A2	-0.5017	1.43E-10	1.51E-09
DTNBP1	-0.50168	1.88E-06	1.18E-05
MAD1L1	-0.50098	7.16E-11	7.82E-10
LRRC47	-0.50098	2.76E-16	4.89E-15
C17orf76-AS1	-0.50065	1.35E-14	2.10E-13
EIF5AL1	-0.50046	0.0084896	0.025605
NCAPD3	-0.50037	2.67E-12	3.38E-11
RP1-193H18.2	-0.50012	0.012655	0.036242
HMHA1	-0.50005	1.44E-12	1.86E-11
ZNF215	-0.50002	2.77E-06	1.70E-05
GUCY1B3	0.5001	1.12E-07	8.51E-07
ZNF260	0.50049	2.13E-15	3.55E-14
RP11-295G20.2	0.50117	0.00010786	0.00051347
FAM83B	0.50141	0.00050286	0.002073
TIPARP	0.502	1.83E-10	1.91E-09
MBD5	0.50213	0.00011988	0.00056694
PLEKHF2	0.50297	1.79E-13	2.52E-12
FUT10	0.50299	1.05E-08	9.03E-08
AKAP13	0.50328	2.09E-08	1.73E-07
RP11-15H20.6	0.5033	0.0016411	0.0059771
EFCAB7	0.50395	0.00098034	0.0037788
RAB30	0.50402	8.19E-07	5.44E-06
PRKX	0.50413	2.81E-19	6.21E-18
QSER1	0.50427	7.70E-16	1.32E-14
GOSR2	0.50451	0.00037164	0.001577
TNFRSF10B	0.50489	1.72E-29	6.60E-28
COL4A5	0.50521	7.54E-13	9.99E-12
ZNF333	0.50527	0.0022357	0.007857
SIK3	0.50534	8.32E-09	7.24E-08
CDK6	0.50544	1.53E-12	1.97E-11
BIRC3	0.50587	0.0025479	0.0088408

CRISPLD2	0.50591	0.00044016	0.0018397
PCBP4	0.50591	5.02E-18	1.00E-16
AFAP1	0.50613	1.16E-07	8.76E-07
C8orf58	0.50647	0.014406	0.040542
SLC16A13	0.50673	0.017727	0.048553
COQ10B	0.50673	1.42E-17	2.74E-16
ZNF200	0.50743	4.33E-05	0.00022195
KANK1	0.50762	3.73E-20	8.72E-19
ABT1	0.50763	2.74E-12	3.45E-11
MT-ND1	0.50794	3.33E-23	9.26E-22
PRKD1	0.50796	1.68E-12	2.15E-11
ZFP36L1	0.50815	3.39E-20	7.94E-19
EBF2	0.50824	0.0040325	0.013263
C7orf43	0.50834	3.11E-08	2.53E-07
ARAP2	0.50875	6.70E-07	4.51E-06
MINPP1	0.50888	4.31E-16	7.56E-15
MAP4K4	0.50888	5.79E-27	1.96E-25
C9orf41	0.50895	6.49E-23	1.77E-21
NPC1	0.5091	2.45E-08	2.01E-07
DCP1B	0.50937	1.47E-15	2.48E-14
GRB14	0.50998	6.81E-07	4.58E-06
PSME4	0.51029	4.93E-19	1.08E-17
PRRG2	0.51074	7.19E-06	4.13E-05
SPATA18	0.51113	6.90E-16	1.19E-14
YIPF5	0.51169	3.53E-23	9.79E-22
KLLN	0.5122	1.43E-07	1.06E-06
TMEM184B	0.51243	6.27E-09	5.55E-08
ZBTB26	0.51288	4.43E-05	0.00022626
RP1-228H13.5	0.51296	0.0016701	0.0060778
ANKRD34A	0.51301	0.00021727	0.00096944
PRDM2	0.51302	2.70E-13	3.73E-12
MLF1	0.51313	1.62E-14	2.50E-13
IDUA	0.51372	0.0012512	0.0046983
SERTAD1	0.51385	1.80E-06	1.13E-05
KIF5C	0.51388	5.20E-08	4.10E-07
JUP	0.51406	7.82E-24	2.24E-22
ADA	0.51424	0.0028589	0.009817
TEX15	0.51461	6.45E-06	3.73E-05
PRAC	0.51483	1.56E-05	8.49E-05
HMG20A	0.51521	5.95E-12	7.28E-11
SORBS2	0.51545	9.49E-08	7.26E-07
PNRC2	0.51553	1.42E-25	4.45E-24
PPIB	0.51589	1.69E-15	2.84E-14
CFL2	0.51592	1.33E-21	3.38E-20
IBTK	0.51622	1.72E-20	4.12E-19

TTPAL	0.51652	4.81E-12	5.94E-11
FAM111A	0.51653	2.73E-20	6.44E-19
CEP170	0.51708	1.97E-13	2.77E-12
SASH1	0.5171	3.60E-09	3.28E-08
SLC44A5	0.51737	0.00093287	0.0036108
CCDC117	0.51783	4.96E-20	1.15E-18
RP11-706O15.1	0.51814	1.60E-07	1.19E-06
MPDZ	0.51863	2.24E-09	2.08E-08
CATSPER2	0.51875	0.0041617	0.013643
STXBP5L	0.51918	1.75E-06	1.10E-05
TMEM87A	0.51935	4.42E-16	7.72E-15
XXbac-BPG308	0.51969	0.0051036	0.016373
SSFA2	0.51969	1.29E-30	5.33E-29
TTC6	0.51992	6.36E-09	5.61E-08
GRHL1	0.51998	1.48E-06	9.46E-06
MAN2A1	0.52036	1.49E-19	3.35E-18
TCP11L2	0.5205	3.69E-05	0.00019065
DUSP10	0.52143	1.71E-17	3.28E-16
RP11-325F22.3	0.52179	0.012496	0.03585
RNF125	0.52182	0.00014208	0.00065903
C12orf49	0.52198	2.73E-22	7.17E-21
SLC2A12	0.5221	1.32E-08	1.12E-07
NCEH1	0.52239	1.23E-10	1.31E-09
ZFHX3	0.52247	1.58E-09	1.50E-08
ZNF74	0.52261	3.25E-11	3.70E-10
IPO7	0.52284	1.53E-31	6.77E-30
CDC14A	0.52288	1.61E-13	2.28E-12
ME1	0.52299	1.04E-09	1.01E-08
PTPRK	0.52299	1.15E-16	2.08E-15
TMEM117	0.52313	3.40E-08	2.75E-07
PVRL4	0.52319	2.42E-07	1.73E-06
ZNF69	0.52365	0.00091537	0.0035586
ARHGAP18	0.52398	1.78E-11	2.09E-10
FAM220A	0.52449	3.19E-13	4.40E-12
DNAJC24	0.52471	1.24E-07	9.31E-07
CACNB2	0.52482	0.00011258	0.0005347
RNF139	0.52514	4.33E-17	8.04E-16
LHX9	0.52531	1.22E-05	6.75E-05
POU5F1	0.52565	0.005371	0.01709
OTUD3	0.52612	1.29E-11	1.53E-10
GLCCI1	0.52623	3.73E-09	3.39E-08
SMAD6	0.52652	2.88E-06	1.76E-05
CTD-2008P7.9	0.52668	0.013106	0.037368
ATP6V1G1	0.52725	2.78E-21	6.95E-20
FRMPD2	0.52739	0.00097974	0.0037773

ZNF175	0.52777	2.40E-05	0.00012685
GLTSCR2	0.52783	3.15E-21	7.85E-20
KIF27	0.52824	0.0011811	0.004465
TRPS1	0.52824	1.82E-11	2.13E-10
QSOX1	0.52831	3.25E-25	9.89E-24
NPNT	0.52836	1.40E-31	6.21E-30
RBM38	0.52843	2.22E-10	2.31E-09
TAP1	0.52942	1.19E-12	1.55E-11
CES2	0.5296	1.42E-19	3.21E-18
HELB	0.53046	0.0027904	0.0095999
JAZF1	0.53119	0.001928	0.0068861
HLA-B	0.53136	0.012879	0.036789
CERKL	0.53167	0.0019842	0.0070639
TMEM25	0.53171	4.09E-19	8.97E-18
THBS1	0.53178	7.11E-23	1.93E-21
ZNF697	0.53187	8.50E-05	0.00041328
MT-ND6	0.53271	4.22E-05	0.00021638
PLCB1	0.53335	1.17E-14	1.83E-13
SNHG16	0.53351	1.51E-21	3.81E-20
ZNF81	0.53373	6.20E-07	4.20E-06
HOPX	0.53418	2.23E-06	1.39E-05
AMMECR1	0.53443	5.30E-19	1.15E-17
PCDH20	0.53444	6.64E-23	1.81E-21
NME5	0.53506	0.00040267	0.0016994
THBS3	0.53572	4.80E-05	0.00024378
SERPINI1	0.53613	6.16E-11	6.77E-10
DNAJB5	0.53672	9.05E-06	5.12E-05
PDPK2	0.53766	0.0017146	0.0062242
CYTH1	0.53791	6.80E-10	6.68E-09
ADAMTS3	0.53797	1.26E-09	1.20E-08
SKIDA1	0.53814	4.84E-06	2.85E-05
MST1P2	0.53833	0.0012722	0.0047705
IER5L	0.53915	0.00014726	0.00068017
TRIM23	0.53947	7.70E-17	1.41E-15
FAM73A	0.53987	1.83E-14	2.81E-13
TNFRSF10D	0.54002	7.92E-18	1.56E-16
RPS6KB2	0.54084	2.22E-13	3.10E-12
HERC5	0.54125	0.00010246	0.00049055
FAM107B	0.54125	1.90E-15	3.18E-14
SIX4	0.54174	3.29E-13	4.54E-12
MYOF	0.54182	3.60E-05	0.00018638
HLA-DQB2	0.54196	1.36E-06	8.78E-06
RNF149	0.54233	5.12E-14	7.51E-13
FTL	0.54235	4.04E-31	1.75E-29
SATB2	0.54237	3.37E-08	2.73E-07

TET3	0.54261	1.16E-19	2.63E-18
TECPR2	0.54289	4.56E-14	6.72E-13
UNC5B	0.54322	0.00013671	0.00063683
ZFHX2	0.54328	1.58E-05	8.59E-05
ZNF493	0.54335	1.37E-06	8.83E-06
WWP1	0.54363	5.00E-20	1.16E-18
AASS	0.54406	3.40E-09	3.11E-08
BTC	0.54467	6.85E-08	5.32E-07
RCN2	0.54501	2.45E-27	8.46E-26
SQSTM1	0.54698	2.51E-19	5.56E-18
GREB1L	0.54771	1.61E-06	1.02E-05
CPNE4	0.54809	0.0141	0.03982
RP5-894A10.6	0.54809	0.011065	0.032241
SPTAN1	0.54886	2.55E-16	4.54E-15
TAGLN	0.54939	0.00059547	0.0024089
AVPI1	0.5497	0.0014049	0.0052032
PLCD3	0.54973	8.35E-09	7.27E-08
CTD-2086O20.3	0.55004	0.016908	0.046569
FURIN	0.55086	1.28E-18	2.71E-17
WASF3	0.55096	6.29E-18	1.25E-16
PCDHA4	0.55156	0.00098343	0.0037868
TEAD2	0.55188	1.23E-16	2.23E-15
BACE2	0.55232	3.31E-10	3.36E-09
PIK3IP1	0.55241	1.59E-10	1.67E-09
MSRB3	0.55278	0.0014181	0.0052437
PELI1	0.55352	1.20E-08	1.02E-07
KIAA0355	0.55355	4.81E-11	5.34E-10
ZBTB34	0.55398	5.20E-07	3.56E-06
TGFB1	0.55424	1.13E-08	9.71E-08
RGAG4	0.55493	0.0021748	0.0076646
PLEKHH1	0.55516	2.91E-14	4.35E-13
E2F5	0.55536	1.55E-11	1.82E-10
ZNF615	0.55584	1.27E-08	1.08E-07
TSKU	0.55626	8.75E-15	1.38E-13
ZCCHC9	0.55637	1.23E-08	1.05E-07
BX322557.10	0.55745	4.61E-05	0.00023484
LPAR2	0.55757	0.0012398	0.0046614
PVRL2	0.55766	2.45E-11	2.82E-10
TNXB	0.55782	0.010735	0.031412
ZNF540	0.55851	0.010188	0.029993
SOCS5	0.55908	3.04E-08	2.48E-07
TMX4	0.55927	2.81E-25	8.62E-24
MAFF	0.55937	5.11E-09	4.57E-08
CCDC66	0.55984	9.71E-06	5.47E-05
TSPAN5	0.56049	4.42E-05	0.00022571

ELF5	0.56106	8.20E-12	9.93E-11
SNIP1	0.56145	2.47E-10	2.55E-09
RP11-298I3.4	0.56153	0.0051432	0.016466
HIST1H2BD	0.56346	0.0020985	0.0074155
ADPRM	0.5644	0.0020714	0.0073344
ZFYVE9	0.5644	2.86E-12	3.60E-11
MOSPD1	0.56444	2.11E-09	1.97E-08
SPINK5	0.56453	2.80E-06	1.72E-05
VPS54	0.56461	3.09E-18	6.33E-17
MMP24	0.56528	6.51E-07	4.39E-06
RPS6KA2	0.56562	2.67E-08	2.19E-07
IGF2R	0.56595	1.89E-14	2.88E-13
PHACTR2	0.5662	6.25E-23	1.71E-21
CDNF	0.56692	0.00025569	0.0011255
NR1D1	0.56831	0.0013019	0.0048682
CACUL1	0.56855	9.81E-26	3.12E-24
STX3	0.56856	1.23E-19	2.78E-18
AC083799.1	0.56883	1.07E-05	6.00E-05
GNPDA1	0.56904	1.14E-17	2.23E-16
PLXDC2	0.56919	0.00098223	0.0037837
C11orf54	0.56935	3.45E-18	7.03E-17
9-Sep	0.57042	3.82E-31	1.66E-29
RP11-680F20.1	0.57059	0.0013452	0.0050079
HSPA4L	0.57069	6.49E-22	1.66E-20
FRS2	0.57111	1.39E-25	4.36E-24
NEK5	0.57159	0.0084758	0.025567
PPIC	0.57162	6.68E-10	6.58E-09
LHFPL2	0.5728	5.82E-08	4.56E-07
AGO3	0.57361	9.62E-10	9.33E-09
KLHDC7A	0.57396	0.0018425	0.0066288
HIST1H2AI	0.57421	0.005276	0.016833
FBXO16	0.57457	6.87E-05	0.0003402
RP11-706O15.3	0.57512	6.09E-07	4.13E-06
MR1	0.57577	9.39E-14	1.35E-12
REEP3	0.5758	1.92E-25	5.94E-24
RND3	0.57611	5.23E-24	1.51E-22
DDHD1	0.57631	1.15E-10	1.23E-09
PRTG	0.57669	1.87E-06	1.17E-05
RBPMS	0.5769	1.64E-06	1.04E-05
AC018642.1	0.57694	0.00096086	0.0037099
LRPAP1	0.57765	1.47E-30	6.05E-29
CEP120	0.57785	2.70E-10	2.77E-09
CLIP4	0.5782	8.01E-10	7.82E-09
CERCAM	0.57853	1.89E-08	1.58E-07
PARP3	0.57861	3.06E-09	2.80E-08

FAM19A2	0.57922	6.08E-08	4.77E-07
DNAH6	0.57934	0.0001082	0.00051499
TMEM45A	0.57961	1.71E-14	2.63E-13
FBXL22	0.58021	0.0079789	0.024245
SLC4A7	0.58035	1.50E-11	1.77E-10
PCDHGA4	0.58053	0.012438	0.0357
STK38L	0.58087	2.34E-21	5.87E-20
NPAS2	0.58094	4.00E-05	0.00020611
ATF3	0.58295	1.29E-15	2.19E-14
ANKRD46	0.58307	8.02E-11	8.70E-10
SEMA6A	0.58363	1.88E-29	7.16E-28
TMEM234	0.58405	0.00062356	0.0025078
ACVR2B	0.5841	0.00010589	0.00050551
FAXDC2	0.58441	2.18E-19	4.86E-18
MOB1B	0.58498	1.10E-22	2.97E-21
ONECUT2	0.58546	4.50E-17	8.34E-16
RNF144B	0.5855	1.08E-26	3.60E-25
CTC-559E9.8	0.58573	0.0036768	0.012212
REV3L	0.58608	7.52E-17	1.38E-15
EDN1	0.58644	0.00021551	0.0009623
STAG3	0.58773	6.50E-08	5.07E-07
PXK	0.58773	9.34E-11	1.01E-09
ADAM10	0.58807	5.40E-33	2.54E-31
NRBP1	0.58817	3.95E-18	7.99E-17
CALCRL	0.58838	0.0085339	0.025718
ZNF286B	0.58889	0.00055157	0.0022518
RP11-297L17.1	0.58979	0.011889	0.034265
ZFAND5	0.59102	4.81E-45	4.11E-43
FER1L4	0.59111	8.31E-05	0.00040539
HIPK2	0.59119	1.45E-16	2.60E-15
ZPLD1	0.59171	2.37E-05	0.00012549
PLCXD2	0.59189	0.0078067	0.02378
ZNF654	0.59262	4.49E-16	7.84E-15
BID	0.59266	2.83E-06	1.73E-05
BDH2	0.59277	1.43E-10	1.51E-09
PPM1L	0.59317	3.62E-06	2.18E-05
PKD2	0.59333	1.22E-12	1.59E-11
RP11-732M18.	0.59339	0.010568	0.030975
HIC1	0.59374	0.01225	0.035197
GRHL2	0.59383	9.69E-35	5.07E-33
PCDHB10	0.59396	5.35E-05	0.0002701
CCDC71L	0.59403	0.0040249	0.013241
FAM200A	0.59454	4.44E-09	4.01E-08
IGFBP1	0.5947	0.017479	0.047995
ATP6V0E1	0.59478	3.29E-23	9.16E-22

ATMIN	0.59478	5.21E-32	2.37E-30
GDF11	0.59496	2.58E-21	6.46E-20
TP53INP2	0.59673	7.47E-19	1.60E-17
NKX2-8	0.59683	0.0014707	0.0054169
ADRB2	0.59735	0.0047135	0.015227
TPPP3	0.59765	1.73E-09	1.63E-08
ANG	0.5978	1.72E-07	1.27E-06
GS1-124K5.2	0.59783	0.012633	0.036191
LRP10	0.59815	1.86E-33	9.00E-32
IGF2BP3	0.5992	6.75E-15	1.08E-13
SREK1IP1	0.59978	1.08E-23	3.09E-22
KIAA0513	0.59989	1.23E-08	1.05E-07
RP11-585P4.5	0.59997	0.002355	0.0082327
PARD6B	0.60009	1.47E-07	1.09E-06
SEMA4B	0.6001	6.05E-09	5.37E-08
RNF43	0.60095	2.22E-13	3.09E-12
MALAT1	0.60156	4.68E-22	1.21E-20
SPDEF	0.60167	5.76E-28	2.05E-26
LINC00612	0.6022	0.011076	0.032267
HIVEP1	0.60227	1.11E-08	9.55E-08
GINM1	0.60262	3.16E-20	7.42E-19
ZSCAN31	0.60271	0.001678	0.0061032
RP11-525E9.1	0.60275	0.001956	0.0069753
GDPD1	0.60283	9.57E-08	7.30E-07
ITGAV	0.60284	6.18E-29	2.29E-27
BMP1	0.60294	2.64E-12	3.34E-11
AP001469.9	0.60311	0.0032345	0.010924
NAP1L2	0.60345	4.62E-11	5.14E-10
STXBP5	0.60443	4.25E-18	8.53E-17
TUSC2	0.60444	2.45E-20	5.80E-19
FTSJD1	0.6045	7.57E-15	1.20E-13
CDKN1B	0.60454	4.53E-47	4.20E-45
ID1	0.6046	7.64E-27	2.56E-25
RFK	0.60507	1.16E-25	3.67E-24
PLSCR4	0.60575	5.58E-05	0.00028023
PDIA5	0.60647	2.54E-15	4.20E-14
SYT5	0.60691	0.00073547	0.0029155
KIF5A	0.60868	2.38E-07	1.71E-06
RNF19A	0.60879	1.25E-12	1.62E-11
ETV7	0.60905	5.51E-05	0.00027718
ST3GAL5	0.60937	0.00046232	0.0019233
HIST1H3E	0.60952	0.0077734	0.023694
RP11-158K1.3	0.60954	0.0087358	0.02625
GSPT2	0.60964	0.009996	0.029531
LCN12	0.60986	0.01546	0.043059

EVI5	0.60994	4.45E-13	6.07E-12
PELI2	0.61007	0.00084495	0.0033048
LPXN	0.61078	1.28E-12	1.66E-11
ZNF596	0.61117	1.37E-08	1.16E-07
DRAXIN	0.61125	0.00203	0.0072118
PCDHA8	0.61134	0.0020808	0.0073626
UGT2B17	0.61174	0.00019872	0.00089286
MVB12B	0.61177	3.74E-15	6.13E-14
MCFD2	0.6118	2.75E-25	8.44E-24
PTCHD4	0.61182	0.0033326	0.011198
CHD9	0.61209	2.40E-18	4.98E-17
ST6GALNAC4P	0.61249	0.014659	0.041165
GPX7	0.61257	2.68E-07	1.91E-06
C14orf37	0.61279	2.05E-05	0.00010966
ZNF79	0.61334	2.85E-10	2.92E-09
KLF4	0.6141	8.10E-11	8.77E-10
ITM2B	0.61633	7.41E-35	3.90E-33
GFM1	0.61674	1.57E-37	9.58E-36
TM7SF3	0.61778	9.42E-50	9.44E-48
ANKEF1	0.61875	1.58E-10	1.66E-09
KB-1732A1.1	0.61888	0.00056933	0.0023157
RP11-15H20.5	0.61958	2.65E-08	2.17E-07
AC007308.6	0.62009	0.015882	0.044091
CREB3L1	0.62037	0.0052145	0.016666
GPRC5D	0.62053	0.003304	0.011121
CICP16	0.62164	0.007138	0.021973
DPYSL4	0.62189	0.00010537	0.00050357
ID2	0.62297	4.69E-30	1.86E-28
AGPAT9	0.62385	0.0078341	0.023851
RHOB	0.62388	1.34E-26	4.42E-25
LINC00086	0.62429	1.73E-07	1.27E-06
SPINK1	0.62453	1.78E-17	3.40E-16
NAV3	0.62485	5.26E-06	3.09E-05
NUDT4P1	0.62506	0.014902	0.041735
8-Mar	0.62519	1.59E-19	3.57E-18
XBP1	0.6261	1.12E-33	5.49E-32
ETNK1	0.62612	1.09E-32	5.10E-31
RAPGEF2	0.62625	2.03E-14	3.09E-13
C9orf3	0.62633	3.62E-13	4.98E-12
CTC-471F3.5	0.62763	0.014153	0.039936
PRKACB	0.62804	1.13E-18	2.40E-17
LPAR3	0.62822	1.04E-16	1.90E-15
C22orf34	0.6284	4.63E-11	5.15E-10
NUDT4	0.62871	5.76E-48	5.42E-46
ASZ1	0.62904	0.0033142	0.011145

SCML2	0.62948	1.04E-14	1.63E-13
MEX3A	0.62976	8.41E-31	3.55E-29
LAMB1	0.62994	6.36E-19	1.37E-17
PART1	0.63105	8.23E-09	7.18E-08
PCDH9	0.63162	7.09E-07	4.76E-06
SPEF1	0.63183	0.0064564	0.020087
C20orf112	0.63226	1.95E-09	1.83E-08
ZFC3H1	0.63258	3.16E-14	4.71E-13
FAM212B	0.63264	1.60E-06	1.02E-05
MAST4	0.6329	2.77E-10	2.84E-09
HECTD2	0.63324	1.96E-15	3.28E-14
RBP1	0.63458	0.014985	0.041912
MAPKAPK3	0.63492	3.69E-29	1.39E-27
UBN2	0.63512	4.18E-18	8.41E-17
SLC30A1	0.63544	1.22E-29	4.73E-28
MAP2K4	0.63575	1.51E-29	5.82E-28
STK38	0.63595	1.95E-17	3.71E-16
CHODL	0.63649	3.27E-11	3.71E-10
PCOLCE2	0.63654	5.81E-13	7.82E-12
CATSPERB	0.63661	0.0018804	0.0067419
PAG1	0.63665	7.89E-11	8.58E-10
RIBC1	0.63706	0.013102	0.037364
TOB2	0.63745	5.39E-36	2.96E-34
TMEM44	0.63818	0.00018616	0.00084213
TET2	0.63857	2.09E-20	4.96E-19
SRPR	0.63858	5.26E-52	5.73E-50
TTN	0.63948	0.0050158	0.016119
ATP1B1	0.63948	9.79E-38	6.02E-36
SYDE2	0.63957	3.30E-10	3.35E-09
TBC1D9	0.64022	1.17E-23	3.31E-22
SOX5	0.64032	7.73E-09	6.76E-08
PARP8	0.64044	3.77E-10	3.80E-09
ITGB8	0.64102	5.32E-13	7.19E-12
RP1-102E24.8	0.64142	0.010043	0.029647
RLN1	0.64168	0.0035828	0.01194
ARL5B	0.64183	8.43E-25	2.49E-23
EIF4BP7	0.6419	0.0039024	0.012883
TMTC1	0.64216	0.0010262	0.0039345
TPI1P2	0.64219	0.0050226	0.016138
RP3-523K23.2	0.64227	0.00066669	0.0026669
DLX2	0.64234	0.00010767	0.00051299
RP11-134G8.8	0.64254	4.16E-08	3.32E-07
MYB	0.6427	8.84E-07	5.84E-06
RP11-303E16.2	0.64284	7.00E-10	6.86E-09
RPS6KA3	0.64308	3.25E-31	1.42E-29

IL27RA	0.64371	0.0067794	0.020983
TSPAN11	0.64524	2.38E-14	3.58E-13
C9orf43	0.64594	0.003807	0.012591
RP11-10K16.1	0.64631	0.0052484	0.016763
ACADL	0.64631	0.00021984	0.00097995
MT-ND5	0.64649	2.05E-24	5.97E-23
EIF3FP3	0.64663	0.01153	0.033417
TEP1	0.64925	8.91E-13	1.17E-11
CCDC146	0.64987	0.0057191	0.018068
SLC35D2	0.65024	4.34E-15	7.07E-14
C1orf213	0.65071	0.0075271	0.023056
SDC4	0.65092	7.16E-11	7.82E-10
KCND2	0.65102	0.00086891	0.0033928
NDN	0.65106	2.04E-20	4.85E-19
ITGA4	0.65161	1.79E-08	1.50E-07
MAP9	0.65194	6.11E-25	1.83E-23
SLC25A18	0.65221	0.015555	0.043303
SLAIN1	0.65324	5.08E-37	2.96E-35
C11orf95	0.65341	4.85E-14	7.12E-13
IRX5	0.65351	4.08E-07	2.85E-06
PCDHB6	0.65391	1.58E-06	1.00E-05
TOX3	0.65404	4.89E-23	1.34E-21
ZFYVE26	0.65448	5.80E-14	8.47E-13
AC093162.5	0.65468	0.0052263	0.016698
GABRB2	0.65486	4.83E-19	1.05E-17
ZFYVE1	0.65531	2.97E-10	3.03E-09
DCP2	0.65563	1.55E-31	6.83E-30
ANKRD22	0.65701	3.40E-07	2.39E-06
NOVA1	0.65768	6.20E-19	1.34E-17
ANKRD19P	0.65772	0.011834	0.034133
SCNN1G	0.6578	0.018165	0.049542
FLJ27365	0.65825	0.00015634	0.0007184
ST8SIA4	0.65858	1.92E-14	2.92E-13
RHOBTB3	0.66004	3.32E-17	6.21E-16
A4GALT	0.66032	0.018168	0.049542
MFAP3L	0.66041	9.76E-05	0.00046951
EPAS1	0.66049	1.37E-07	1.02E-06
CTB-118P15.2	0.66118	0.011918	0.034345
FAM84A	0.66162	1.04E-38	6.70E-37
MED28	0.66249	3.02E-28	1.09E-26
CHRM2	0.66253	0.0018827	0.0067489
BLNK	0.66282	8.90E-07	5.87E-06
DCAF4L1	0.6646	0.0012966	0.0048503
ST3GAL1	0.66594	5.41E-54	6.54E-52
MPZL2	0.66696	3.31E-22	8.64E-21

VWA7	0.66701	2.64E-13	3.66E-12
PIKFYVE	0.66781	5.03E-28	1.80E-26
PBX2	0.66819	1.23E-23	3.47E-22
RP11-12M5.4	0.66863	0.017873	0.04888
TMEM133	0.66886	0.001349	0.0050211
RP11-294O2.2	0.66925	0.00049093	0.0020278
SOX2	0.66988	0.017064	0.04697
BTBD3	0.67021	2.07E-22	5.49E-21
FGF5	0.67047	0.014828	0.041573
RP11-50C13.1	0.67201	0.0045472	0.014751
TOB1	0.67219	1.04E-49	1.03E-47
PIH1D2	0.67266	0.0030514	0.010406
DNAJB4	0.67293	2.32E-11	2.68E-10
RP11-10J21.4	0.67467	0.014691	0.041239
AC004112.4	0.67523	0.0096228	0.028597
KANK3	0.6755	2.82E-09	2.59E-08
RP11-235E17.5	0.67574	0.0041943	0.013742
DOK6	0.67595	4.09E-27	1.39E-25
FOSL2	0.67607	5.75E-08	4.52E-07
PLA2G10	0.67622	0.016535	0.045684
RFX6	0.67645	0.012597	0.036097
PLEKHA3	0.67703	4.72E-15	7.65E-14
MFGE8	0.67744	1.77E-17	3.37E-16
HBP1	0.67848	1.33E-30	5.47E-29
SPATA2	0.67911	3.76E-14	5.56E-13
PTPRZ1	0.67916	1.00E-08	8.63E-08
DACH1	0.67925	3.50E-10	3.54E-09
SEC14L1	0.67927	1.93E-18	4.02E-17
RBMS2	0.67932	1.26E-10	1.33E-09
SESN3	0.68011	4.02E-33	1.90E-31
WSB1	0.68048	6.76E-31	2.88E-29
RP11-599B13.7	0.68072	0.015856	0.044044
SUMF1	0.68104	1.51E-30	6.17E-29
RP1-149A16.3	0.68167	0.01128	0.032768
WNT3	0.68179	0.0088901	0.02667
ENTPD4	0.68215	9.70E-31	4.06E-29
RP11-33B1.2	0.68229	0.0060536	0.01898
AP4S1	0.68241	0.0019031	0.0068154
PRKG1-AS1	0.68255	0.0041159	0.013504
ALCAM	0.6829	2.85E-53	3.23E-51
PIFO	0.68344	0.0010711	0.0040896
HADHB	0.68366	4.58E-48	4.36E-46
ATP8B1	0.68394	5.75E-22	1.48E-20
GFRA1	0.68426	7.53E-08	5.82E-07
MMP1	0.68455	0.0060621	0.019003

ST8SIA1	0.68496	0.0095615	0.028433
RPL9P18	0.68541	0.015027	0.042015
CEP19	0.68582	1.07E-05	5.97E-05
BMS1P5	0.68656	0.0077165	0.023552
ATF6B	0.68681	3.37E-38	2.13E-36
BMF	0.68682	9.29E-12	1.12E-10
VASH2	0.68748	2.81E-17	5.30E-16
IGBP1	0.68778	5.92E-17	1.09E-15
RP11-18J9.3	0.68795	0.014821	0.041559
M6PR	0.688	6.62E-30	2.62E-28
NEB	0.68838	0.0081011	0.02458
ANK1	0.68863	2.56E-19	5.67E-18
TGFA	0.68916	1.28E-06	8.24E-06
DCP1A	0.68972	1.06E-47	9.85E-46
TUBB8P8	0.6901	0.013842	0.03919
RP11-876N24.!	0.69055	0.0052629	0.016803
STRADB	0.69121	2.14E-23	6.00E-22
HES1	0.69146	2.97E-23	8.29E-22
IGF2BP2	0.69186	4.51E-21	1.12E-19
GLI3	0.69246	1.47E-07	1.09E-06
RP11-452H21.4	0.69294	0.013961	0.039486
F2R	0.69336	5.46E-05	0.00027491
PPP1R15A	0.69468	1.64E-27	5.70E-26
ARID3B	0.69506	6.77E-07	4.56E-06
RP11-178L8.9	0.69603	0.012715	0.036382
DLGAP1-AS1	0.69627	0.0081675	0.024738
TGFBR2	0.69639	6.08E-10	6.02E-09
RP1-140K8.5	0.6964	0.012812	0.036631
CNIH3	0.69645	0.0070095	0.02162
CTD-2561B21.!	0.69664	0.013121	0.037401
TRIB1	0.69677	4.19E-46	3.78E-44
NAP1L6	0.698	0.0014892	0.0054775
RP11-766N7.3	0.6988	0.011685	0.033788
GBP2	0.70132	0.0097093	0.028817
E2F3	0.70148	6.58E-27	2.22E-25
SLC22A23	0.70181	1.50E-27	5.24E-26
KIAA0922	0.70286	1.38E-14	2.14E-13
ZNF880	0.70357	7.34E-11	7.99E-10
RP11-432M8.1	0.70428	0.0091569	0.027387
LRRC66	0.70485	0.011468	0.033263
FILIP1L	0.7054	0.011825	0.034124
PLA2G7	0.70627	9.83E-34	4.83E-32
ITM2A	0.7063	0.0020624	0.0073057
PPAP2B	0.70633	2.92E-21	7.30E-20
GPC6	0.70816	6.39E-25	1.91E-23

TOX	0.70853	7.59E-07	5.07E-06
PLBD2	0.70899	2.27E-25	6.99E-24
RP11-31F19.1	0.70905	0.0099393	0.029406
CABYR	0.7095	0.00015714	0.00072171
GNS	0.70987	2.20E-50	2.30E-48
CSNK1G1	0.71007	3.46E-27	1.19E-25
RNF122	0.71061	2.51E-07	1.80E-06
BTG3	0.71079	1.15E-23	3.27E-22
RNF44	0.71173	3.82E-50	3.93E-48
HS3ST3B1	0.71202	0.00083375	0.0032671
PHLPP2	0.71266	4.46E-23	1.23E-21
NIPA1	0.71273	4.72E-25	1.42E-23
PNRC1	0.71308	3.52E-25	1.07E-23
P2RY1	0.71369	2.76E-08	2.25E-07
GS1-304P7.2	0.71402	0.0096642	0.028707
PCDH11X	0.71424	4.40E-09	3.97E-08
TNFSF8	0.71425	0.010991	0.032068
MMP16	0.71543	5.99E-32	2.71E-30
TP53I3	0.71547	7.41E-06	4.24E-05
APOE	0.71603	4.10E-06	2.45E-05
ETS2	0.71654	8.60E-31	3.62E-29
HS3ST3A1	0.71674	0.0034406	0.011516
HOXA7	0.71677	0.0086248	0.025958
RP11-21L23.2	0.71723	0.00013159	0.00061606
DYRK1B	0.71816	1.39E-16	2.50E-15
BCAS1	0.71854	0.010024	0.0296
CCDC68	0.71855	0.010835	0.031673
ZNF235	0.72004	2.23E-05	0.00011909
RP11-38P22.2	0.72209	0.00011395	0.00054111
RP11-33B1.4	0.72235	0.0027086	0.0093444
ODC1	0.72283	2.55E-66	4.10E-64
MBNL2	0.72286	4.04E-16	7.09E-15
PHC3	0.72314	2.06E-20	4.89E-19
YPEL3	0.72405	3.81E-14	5.62E-13
PLEKHA1	0.72419	1.19E-25	3.76E-24
GPR85	0.72556	1.54E-19	3.46E-18
SLC16A9	0.72606	0.00014947	0.00068936
AC007970.1	0.72743	0.0080142	0.024344
CEP85L	0.72898	1.89E-09	1.77E-08
NCOA3	0.72926	6.25E-28	2.21E-26
PRRG1	0.72927	2.75E-18	5.66E-17
RP11-642P15.1	0.72999	0.00032271	0.0013846
APLN	0.73008	7.94E-74	1.54E-71
RP11-540O11.[illegible]	0.73123	0.0034724	0.01161
DNAH7	0.73181	0.0031627	0.010722

SP110	0.73265	2.15E-11	2.49E-10
NKAIN1P1	0.73287	0.0019722	0.0070263
ARID5B	0.73317	1.74E-40	1.20E-38
RTN4	0.73325	4.12E-44	3.43E-42
PCDHB11	0.73363	2.44E-10	2.52E-09
CTD-2587M2.1	0.73379	0.0088871	0.026666
MTHFR	0.73399	2.33E-22	6.15E-21
LYG1	0.73449	0.0038322	0.012667
FAM63B	0.73488	1.34E-27	4.70E-26
FYCO1	0.73488	3.25E-38	2.06E-36
IL20RB	0.73497	0.0015392	0.0056456
RAB11FIP2	0.73669	3.49E-21	8.68E-20
EPHA5	0.73708	0.00013246	0.00061953
C2orf15	0.73864	8.11E-06	4.62E-05
UGT2B15	0.73875	2.05E-37	1.23E-35
CPED1	0.73971	6.55E-07	4.42E-06
SAP30	0.73991	1.85E-14	2.84E-13
CBLN4	0.74001	0.0059608	0.018739
OAT	0.74025	3.02E-45	2.61E-43
RANBP6	0.74037	2.38E-22	6.27E-21
BAZ2B	0.74099	2.54E-18	5.24E-17
LHFP	0.7412	4.08E-12	5.06E-11
LMO7	0.7412	6.39E-27	2.16E-25
RP11-229A12.2	0.74193	0.0038318	0.012667
SYTL2	0.74199	3.70E-34	1.87E-32
SOX21	0.7423	0.0040116	0.013206
CLU	0.74253	8.07E-73	1.53E-70
CTTNBP2	0.74261	1.78E-06	1.12E-05
CLCF1	0.74366	0.00017801	0.00080821
ID3	0.74463	3.62E-51	3.90E-49
SLC38A4	0.74493	6.54E-21	1.61E-19
SOBP	0.74499	9.72E-11	1.04E-09
GADD45A	0.74567	4.69E-29	1.76E-27
ELL2	0.74592	2.86E-65	4.33E-63
HES2	0.74609	5.04E-08	3.99E-07
WDR66	0.74687	4.11E-05	0.00021111
HSP90B2P	0.7475	0.0072084	0.022175
NCAM2	0.74751	1.22E-30	5.09E-29
TNPO1	0.74783	2.58E-53	3.01E-51
GABRA1	0.74784	3.51E-36	1.96E-34
PLXNB1	0.74896	1.42E-28	5.19E-27
BEND4	0.74919	1.08E-11	1.29E-10
RP11-701H24.4	0.74955	9.86E-05	0.00047379
FBXW7	0.75034	3.85E-22	1.00E-20
DBC1	0.75035	4.52E-05	0.00023028

CCDC96	0.75059	0.0060782	0.019047
RAB8B	0.75077	1.81E-22	4.81E-21
RP11-115D19.1	0.75087	8.53E-05	0.00041476
NKIRAS1	0.75157	1.94E-09	1.82E-08
CNNM2	0.7517	8.97E-16	1.53E-14
AP006222.2	0.75354	0.0068153	0.021059
DYNLT3	0.75466	6.00E-26	1.93E-24
CREBRF	0.75515	1.81E-15	3.04E-14
GRAMD3	0.75583	4.61E-11	5.14E-10
MAP3K5	0.75588	7.12E-29	2.63E-27
ATP8A1	0.75646	5.93E-15	9.53E-14
ELK4	0.75688	7.93E-46	7.03E-44
ITGB5	0.75879	1.08E-82	2.76E-80
IKZF2	0.75976	1.44E-12	1.86E-11
CEBPD	0.76046	5.27E-24	1.52E-22
DOCK4	0.76052	3.43E-08	2.78E-07
SNPH	0.76102	1.59E-05	8.64E-05
DSCAM	0.76208	0.002746	0.0094628
FGD6	0.76218	4.61E-13	6.27E-12
PGPEP1	0.76245	1.14E-16	2.06E-15
DFNA5	0.76298	3.70E-05	0.00019104
FRAT1	0.76332	2.04E-11	2.39E-10
GPR116	0.76473	8.03E-05	0.00039288
PHYHIP	0.76579	0.0052253	0.016697
RP11-77I22.3	0.76586	0.0064893	0.020173
ZNF711	0.76662	7.86E-15	1.24E-13
CAPRIN2	0.76673	1.47E-14	2.28E-13
TENM1	0.76745	5.20E-07	3.56E-06
RTEL1-TNFRSF	0.76767	0.00050863	0.0020935
ZG16B	0.76798	2.52E-18	5.21E-17
ARHGEF3	0.76805	1.69E-06	1.07E-05
EDNRB	0.7689	0.0047497	0.015328
LRRC37A11P	0.76906	0.0059735	0.018773
ARRDC3	0.7692	9.26E-41	6.46E-39
RP11-403I13.8	0.76973	9.51E-05	0.00045821
CRTAP	0.76988	1.46E-53	1.75E-51
CYR61	0.77047	6.45E-10	6.37E-09
GHR	0.77059	4.72E-17	8.74E-16
BCO2	0.77074	0.0056783	0.017963
ANGPTL3	0.77285	1.93E-07	1.41E-06
STXBP4	0.77291	1.05E-19	2.40E-18
NOS2P3	0.77335	0.0061579	0.019274
CBLN2	0.77356	6.25E-53	6.97E-51
ADAM23	0.77412	4.26E-07	2.96E-06
SNX25	0.77448	9.73E-11	1.05E-09

CPE	0.77508	6.17E-76	1.29E-73
PDGFC	0.77526	5.37E-09	4.80E-08
REG4	0.77616	4.60E-06	2.72E-05
ADCYAP1	0.77709	0.0034206	0.011461
KLF5	0.77811	2.94E-37	1.74E-35
LPL	0.77832	1.33E-08	1.13E-07
SOX9	0.77865	2.64E-53	3.05E-51
TVP23B	0.77896	8.11E-37	4.65E-35
EOGT	0.77973	9.45E-26	3.01E-24
MAPK6	0.78016	4.09E-40	2.77E-38
SEMA3E	0.7802	3.43E-11	3.89E-10
AMOTL1	0.78071	3.51E-24	1.02E-22
ANXA2	0.78112	0.0010587	0.0040454
RP11-214O1.2	0.78389	0.0047238	0.015252
FAM43A	0.78684	1.21E-10	1.29E-09
C12orf60	0.7875	1.78E-06	1.12E-05
CLDN1	0.78767	8.23E-09	7.18E-08
EYS	0.78774	0.0024518	0.0085403
FAM126B	0.78994	4.07E-23	1.12E-21
EPHA2	0.79198	6.61E-39	4.31E-37
PCTP	0.79209	3.60E-25	1.09E-23
LINC00173	0.79247	0.0032678	0.011019
ADAMTS19	0.79319	0.00034675	0.0014775
UGT2B11	0.79364	9.49E-11	1.02E-09
ENTPD7	0.79392	4.71E-22	1.22E-20
RIT1	0.79462	3.16E-36	1.77E-34
MTMR9	0.795	1.29E-20	3.12E-19
IKBIP	0.79511	1.65E-18	3.46E-17
NPY1R	0.79547	0.0040368	0.013273
AC010642.1	0.79674	0.00010081	0.00048338
FAM195B	0.79811	0.0032538	0.010979
KIAA1217	0.79812	7.45E-20	1.71E-18
ZKSCAN7	0.7982	0.00025878	0.0011375
PRDM13	0.79893	0.00062718	0.0025202
CDK8	0.79894	7.53E-28	2.65E-26
SIPA1L2	0.79963	8.15E-50	8.21E-48
LINC00869	0.80075	0.0038667	0.012772
RRAD	0.80187	0.00081623	0.0032069
AKAP12	0.80193	1.69E-20	4.05E-19
PALMD	0.803	1.45E-06	9.31E-06
VN1R83P	0.80393	0.0020408	0.0072432
TMEM64	0.80526	4.44E-51	4.73E-49
RP11-382A20.2	0.80538	0.003727	0.012355
PPP2R5A	0.8057	8.02E-39	5.17E-37
ANTXR2	0.80586	5.33E-18	1.06E-16

KCTD8	0.80776	0.00082902	0.00325
RDH10	0.80777	1.83E-25	5.67E-24
RP11-597D13.7	0.80854	0.0039726	0.01309
YOD1	0.80959	5.21E-48	4.93E-46
SLC9A7	0.80977	2.89E-25	8.82E-24
NREP	0.81039	1.32E-11	1.56E-10
TSPYL2	0.81089	9.71E-36	5.26E-34
ROBO2	0.81331	1.49E-10	1.57E-09
SCIN	0.81547	7.86E-13	1.04E-11
GPR161	0.81573	0.0019056	0.0068231
CCDC121	0.81623	2.96E-10	3.02E-09
SLC40A1	0.81624	3.42E-45	2.94E-43
LEPREL1	0.81654	6.07E-07	4.12E-06
LANCL3	0.81738	2.15E-14	3.26E-13
GPC2	0.81788	1.59E-08	1.34E-07
SHC4	0.81854	0.00084171	0.0032949
SPOCK1	0.81946	6.25E-05	0.00031197
NR1H2	0.82006	1.94E-35	1.05E-33
SEMA3D	0.82097	7.40E-43	5.65E-41
IHH	0.82127	0.0034493	0.011539
CTB-157D17.1	0.82142	0.00026131	0.0011475
LIPH	0.82173	2.25E-12	2.86E-11
PLEKHG1	0.82181	0.0030368	0.010361
ELF3	0.82182	8.73E-40	5.87E-38
FAM135A	0.82194	6.68E-27	2.25E-25
THSD4	0.82209	1.97E-07	1.44E-06
HIST1H2AG	0.82212	2.04E-08	1.69E-07
RFX5	0.82311	1.19E-61	1.68E-59
ENPP4	0.8237	5.95E-20	1.37E-18
FGA	0.82374	0.00092522	0.0035897
PTP4A1	0.82444	3.77E-55	4.65E-53
PRICKLE2	0.82455	3.89E-07	2.72E-06
RP5-968P14.2	0.82514	6.25E-06	3.64E-05
APCDD1	0.82526	0.0007305	0.0029004
CD55	0.82531	2.47E-39	1.64E-37
RP11-102F4.3	0.82541	4.77E-16	8.30E-15
KLHL15	0.82684	8.27E-29	3.04E-27
RNF148	0.83051	0.0023409	0.0081911
CTSA	0.83177	1.17E-65	1.84E-63
IGF1	0.83203	0.0030761	0.010471
SYNPO2	0.83203	1.57E-09	1.49E-08
DUSP1	0.83206	1.92E-13	2.69E-12
MAP7	0.83238	4.17E-67	6.94E-65
GAL3ST4	0.83267	3.08E-07	2.18E-06
KLHL24	0.83519	5.11E-36	2.82E-34

TWIST1	0.8352	1.91E-33	9.22E-32
ZNF528	0.83636	1.13E-12	1.48E-11
RP11-813N20.1	0.83679	4.56E-06	2.71E-05
AC084082.3	0.83703	0.002887	0.0098959
KLHL20	0.83805	2.64E-34	1.35E-32
RASGEF1B	0.83872	0.00077438	0.0030585
NTS	0.83935	4.64E-14	6.83E-13
INSM1	0.83966	0.0023634	0.0082573
SEMA3A	0.84029	0.0023505	0.0082218
MYLIP	0.84119	2.52E-15	4.18E-14
C1orf95	0.84159	5.24E-07	3.59E-06
CADPS2	0.84184	4.74E-36	2.62E-34
MTF2	0.84245	2.66E-33	1.28E-31
KDM6B	0.84275	5.67E-47	5.20E-45
UBASH3B	0.84286	0.00062203	0.0025033
CADM2	0.84513	7.87E-08	6.07E-07
DICER1	0.84579	7.62E-48	7.13E-46
TFCP2L1	0.8465	6.82E-17	1.25E-15
DUSP8	0.84873	7.82E-20	1.80E-18
C2CD4D	0.84881	1.58E-05	8.57E-05
GALNT10	0.84888	1.38E-48	1.34E-46
GRM3	0.84891	7.47E-07	5.00E-06
ASXL3	0.84932	2.99E-05	0.00015673
YPEL2	0.84942	1.13E-16	2.05E-15
DIXDC1	0.84947	1.11E-26	3.68E-25
CHST2	0.84971	7.12E-09	6.25E-08
USP6NL-IT1	0.8507	0.00098272	0.0037848
PMAIP1	0.85126	2.18E-22	5.76E-21
PLK2	0.85247	1.30E-22	3.48E-21
FNDC3B	0.85299	5.68E-35	3.00E-33
RORB	0.85384	1.05E-09	1.01E-08
PCDH15	0.85424	0.0019187	0.0068606
MUC4	0.85539	0.001038	0.0039733
KATNAL1	0.85543	2.90E-14	4.34E-13
RP3-412A9.10	0.8555	0.00072512	0.0028826
RASSF8	0.85566	3.52E-12	4.39E-11
ARHGAP29	0.85638	8.49E-15	1.34E-13
CLSTN3	0.85707	1.32E-12	1.72E-11
HS6ST2	0.85724	1.52E-29	5.86E-28
ENOX1	0.85891	1.05E-18	2.23E-17
UTRN	0.86118	1.80E-10	1.88E-09
PI15	0.86121	0.0018786	0.0067367
F5	0.86165	4.60E-11	5.12E-10
STAT4	0.86266	7.57E-07	5.06E-06
COL4A6	0.86391	2.39E-08	1.97E-07

RP11-597D13.9	0.86462	5.35E-11	5.91E-10
PLEKHA8	0.86492	2.33E-34	1.19E-32
LINC00106	0.86507	0.00049681	0.0020503
ITGA5	0.8653	4.00E-27	1.37E-25
ISG20	0.86534	5.57E-17	1.03E-15
DRAM1	0.86562	6.16E-28	2.19E-26
BCL9	0.86605	3.58E-59	4.77E-57
FAM177B	0.86701	0.00013814	0.00064237
NHLRC3	0.86707	5.63E-19	1.22E-17
OGFOD2	0.86787	0.0013508	0.0050258
AC010091.1	0.87058	7.16E-08	5.55E-07
MEX3B	0.87061	3.02E-09	2.77E-08
TNFRSF12A	0.87096	3.35E-15	5.52E-14
PHLDA1	0.8712	1.63E-17	3.12E-16
ATP8B5P	0.87527	0.0019229	0.0068743
CDKL5	0.8764	0.0002592	0.0011391
NBEA	0.87695	5.05E-29	1.89E-27
GPR110	0.87721	6.47E-05	0.00032194
CBLB	0.87825	3.72E-34	1.88E-32
PAPSS2	0.87961	5.37E-22	1.38E-20
POU3F1	0.87969	0.0018276	0.006585
POU4F2	0.88064	0.0017758	0.0064239
HS3ST5	0.88112	0.0017854	0.0064547
CYP39A1	0.883	1.99E-16	3.55E-15
ERO1LB	0.88456	4.45E-11	4.97E-10
PPP2R1B	0.88615	2.85E-53	3.23E-51
C9orf152	0.88713	5.67E-07	3.86E-06
RP11-158H5.7	0.88778	3.28E-06	1.98E-05
LARGE	0.88818	3.94E-13	5.40E-12
SQRDL	0.88895	0.00035902	0.001527
GRM7	0.89051	5.57E-05	0.00027978
LATS2	0.89222	8.35E-32	3.75E-30
SRPX	0.89232	2.80E-18	5.77E-17
XK	0.89355	1.05E-36	5.98E-35
NOV	0.89356	2.06E-33	9.94E-32
ZNF629	0.89477	2.90E-13	4.00E-12
SULF1	0.89538	7.13E-07	4.78E-06
DCC	0.8976	4.99E-07	3.43E-06
LUM	0.89958	5.70E-12	6.99E-11
CALCOCO1	0.90074	1.48E-53	1.77E-51
RP11-379K17.1	0.90094	3.04E-11	3.47E-10
ZSWIM6	0.90133	1.10E-25	3.48E-24
IL13RA2	0.90171	9.68E-06	5.46E-05
JUN	0.90292	9.64E-36	5.24E-34
ZNF608	0.90296	3.80E-18	7.71E-17

CCR1	0.90368	0.00013803	0.00064204
KIAA1211	0.90433	2.10E-31	9.18E-30
MKNK2	0.90685	4.50E-76	9.53E-74
GALNTL6	0.90921	0.00030691	0.0013241
SIK1	0.91003	1.52E-20	3.64E-19
SAA1	0.91056	0.0010053	0.0038589
GADD45G	0.91246	1.41E-15	2.39E-14
FREM3	0.91258	9.79E-07	6.42E-06
CCDC114	0.9137	0.00098098	0.0037797
FOXP1	0.9147	4.24E-36	2.36E-34
CDH12	0.91511	9.90E-35	5.17E-33
PRICKLE1	0.91584	0.00030773	0.0013266
ROR1	0.916	0.00046436	0.0019309
AMOTL2	0.91635	1.89E-23	5.32E-22
DNAJC27	0.91685	1.00E-14	1.58E-13
SLIT2	0.91691	3.51E-07	2.47E-06
ZNFX1	0.91698	1.17E-31	5.22E-30
MMP11	0.91746	1.96E-14	2.98E-13
KDELC1	0.91789	8.10E-19	1.73E-17
RP11-453F18_	0.92055	1.94E-05	0.000104
NFKBIZ	0.92071	9.50E-81	2.27E-78
ZNF385A	0.92302	5.45E-46	4.86E-44
NR6A1	0.92351	1.55E-09	1.48E-08
CCNJ	0.92361	7.71E-26	2.46E-24
RP11-594N15.3	0.92371	0.00049177	0.0020304
APOF	0.92388	0.00072116	0.0028682
SYTL5	0.92719	1.39E-26	4.59E-25
TGIF1	0.92972	2.84E-28	1.03E-26
SLCO5A1	0.93344	1.14E-31	5.10E-30
GLG1	0.93397	3.33E-70	6.08E-68
INPP5D	0.9349	0.00014147	0.00065654
ZNF671	0.9351	0.00028099	0.0012241
PLA2G4A	0.93618	1.86E-23	5.26E-22
NEDD9	0.93689	1.11E-22	2.98E-21
PAM	0.9375	4.65E-41	3.27E-39
AL050303.1	0.93788	3.60E-06	2.17E-05
CA2	0.93926	0.00021577	0.0009632
LIMD2	0.94131	4.03E-07	2.81E-06
FAM65B	0.94188	1.77E-37	1.07E-35
FTLP14	0.94393	1.11E-05	6.19E-05
OTX1	0.94735	6.27E-09	5.55E-08
RORC	0.94913	2.78E-11	3.19E-10
GRID2	0.94916	6.87E-15	1.09E-13
TUBA1A	0.94934	4.17E-64	6.06E-62
LPGAT1	0.95224	5.24E-84	1.38E-81

CYP7A1	0.95347	0.00051854	0.0021291
PCDH17	0.95391	1.45E-06	9.31E-06
RP11-24B21.1	0.9552	0.00045132	0.0018801
ZCCHC12	0.95658	0.00024119	0.0010662
ZMAT3	0.95696	4.88E-81	1.20E-78
NCKAP5L	0.96152	1.48E-08	1.24E-07
HIST1H2BG	0.9628	0.00037891	0.001605
UST	0.96477	1.67E-20	4.00E-19
SLC10A7	0.96605	9.89E-31	4.13E-29
WDR63	0.96716	1.90E-10	1.98E-09
ADAMTS5	0.96792	0.00060634	0.0024492
FLRT3	0.97127	6.14E-27	2.08E-25
LINC00327	0.97162	0.00026916	0.0011775
TNRC6A	0.97259	3.36E-71	6.20E-69
TMEM2	0.97298	1.46E-76	3.13E-74
ALB	0.97298	4.36E-110	1.73E-107
C12orf5	0.97445	1.98E-29	7.54E-28
TNFRSF21	0.97468	2.94E-134	1.57E-131
RBMS1	0.97779	0.00049994	0.0020618
GALNT7	0.97868	4.08E-60	5.55E-58
PTPN14	0.97883	8.15E-21	1.99E-19
SLC52A1	0.98026	2.09E-21	5.25E-20
RP11-119F7.5	0.98167	9.03E-05	0.00043685
SEMA3C	0.98355	2.04E-90	5.93E-88
FAS	0.98506	6.69E-51	7.04E-49
LAMC2	0.99006	1.41E-21	3.58E-20
WIF1	0.99289	5.38E-12	6.62E-11
FOXA3	0.99421	0.00023116	0.0010263
CDC37L1	0.99444	6.77E-37	3.92E-35
KHNYN	0.9966	2.00E-30	8.05E-29
CPEB2	0.99669	4.37E-19	9.56E-18
CRISPLD1	0.99734	7.44E-31	3.15E-29
WDR44	0.99739	1.99E-65	3.04E-63
CTC-429C10.2	0.99744	0.00041055	0.0017299
CD24P4	0.99806	2.64E-09	2.43E-08
DNAJC12	0.9983	1.68E-27	5.83E-26
MFAP2	0.99889	5.68E-18	1.13E-16
LRP1	1.0024	7.12E-20	1.64E-18
LRP1B	1.0041	3.16E-06	1.92E-05
COL19A1	1.0073	0.00018639	0.00084295
TNC	1.008	9.11E-72	1.70E-69
SLC38A2	1.0082	9.55E-112	3.95E-109
SPATA7	1.0085	1.76E-13	2.48E-12
LINC00511	1.0088	7.42E-25	2.19E-23
HTR7	1.0093	1.30E-05	7.17E-05

MAN1A1	1.0126	1.25E-80	2.95E-78
CCNG2	1.0138	9.32E-70	1.67E-67
MIA3	1.0153	8.70E-89	2.46E-86
RASSF6	1.0154	3.02E-06	1.84E-05
FAM214A	1.0163	4.86E-44	4.01E-42
INPP5A	1.0183	1.15E-40	8.02E-39
MCC	1.022	1.27E-06	8.24E-06
GPR137B	1.0227	4.09E-39	2.69E-37
IL6R	1.0237	8.78E-74	1.69E-71
ADAMTS1	1.0244	3.88E-69	6.82E-67
NABP1	1.0275	3.40E-13	4.68E-12
NR4A3	1.031	4.93E-06	2.90E-05
TMC7	1.0373	1.49E-14	2.31E-13
RHOBTB1	1.0387	6.60E-68	1.12E-65
IQCJ-SCHIP1	1.0399	7.11E-07	4.77E-06
IER3	1.0437	2.22E-16	3.95E-15
SERPINB5	1.0454	1.93E-15	3.23E-14
HMGCS2	1.047	1.52E-10	1.60E-09
SPOCK2	1.0488	2.22E-145	1.48E-142
FAM160B1	1.0513	1.23E-41	8.97E-40
A1CF	1.0523	4.65E-41	3.27E-39
USP54	1.0533	3.32E-108	1.26E-105
ARHGAP42	1.0543	6.95E-19	1.50E-17
UGT1A1	1.0545	0.00016529	0.00075469
FNDC3A	1.0556	6.85E-79	1.56E-76
TP73	1.056	1.14E-09	1.09E-08
RAP2C	1.0591	1.89E-65	2.94E-63
SMOC1	1.0595	1.75E-22	4.67E-21
LEF1	1.06	1.15E-35	6.20E-34
ARG2	1.0615	3.48E-23	9.68E-22
MATN2	1.0629	3.64E-09	3.31E-08
RP11-206M11.	1.0668	7.29E-13	9.67E-12
NAP1L3	1.0678	2.33E-57	3.02E-55
PITPNC1	1.0772	2.68E-13	3.71E-12
ECEL1	1.0779	1.70E-08	1.43E-07
LACC1	1.0794	5.32E-15	8.58E-14
SERPINE1	1.0821	0.000109	0.0005185
LCOR	1.0841	8.65E-62	1.23E-59
CEACAM1	1.085	2.08E-09	1.94E-08
AL021068.1	1.0868	2.56E-06	1.58E-05
RFTN1	1.0905	5.11E-08	4.04E-07
TSLP	1.091	6.92E-30	2.73E-28
PGM2L1	1.0923	7.63E-12	9.28E-11
FOXP2	1.0924	3.39E-37	1.99E-35
BCL3	1.0942	3.29E-28	1.18E-26

SNORD3A	1.0959	9.18E-05	0.00044354
GSTA1	1.0989	2.95E-13	4.07E-12
SLITRK6	1.1014	7.83E-62	1.12E-59
KIAA1199	1.1049	8.60E-12	1.04E-10
ARHGAP30	1.1091	1.85E-14	2.83E-13
CNTN1	1.1118	4.06E-08	3.25E-07
DOCK10	1.1119	4.38E-11	4.90E-10
GPRC5A	1.1119	9.60E-35	5.04E-33
PSD3	1.1154	1.70E-64	2.49E-62
PRSS23	1.1155	6.89E-138	4.14E-135
DDIT4L	1.1173	1.22E-68	2.10E-66
CP	1.122	4.57E-16	7.96E-15
HDHD1	1.1231	3.06E-51	3.31E-49
SAT1	1.1297	4.43E-35	2.36E-33
DDX3Y	1.1318	1.15E-108	4.48E-106
GSN	1.1321	5.19E-39	3.40E-37
GSTA2	1.1326	1.94E-11	2.28E-10
SCUBE2	1.133	8.00E-56	1.01E-53
PZP	1.1337	7.95E-07	5.30E-06
PDE4B	1.1384	4.27E-67	7.05E-65
CACNA1D	1.1401	7.63E-58	9.94E-56
TSPAN19	1.1457	4.70E-05	0.0002386
ABCA1	1.1462	2.98E-10	3.04E-09
RSPO3	1.1517	2.22E-07	1.61E-06
EGR2	1.1527	4.06E-06	2.43E-05
CNTD1	1.1543	2.05E-06	1.28E-05
CNTNAP3	1.1544	9.70E-17	1.77E-15
AHR	1.1566	4.85E-60	6.55E-58
CROT	1.1577	4.59E-116	2.14E-113
FAM196A	1.1644	7.64E-09	6.69E-08
LPHN2	1.1648	5.02E-31	2.15E-29
ADM	1.1679	9.20E-07	6.06E-06
MMP10	1.168	2.31E-05	0.00012274
IL8	1.1717	3.32E-05	0.00017279
FAM46A	1.1729	8.30E-152	5.95E-149
FOS	1.1745	5.79E-38	3.63E-36
CSMD3	1.1809	3.63E-06	2.18E-05
CTB-167B5.2	1.1887	1.81E-05	9.77E-05
CGA	1.1889	1.09E-07	8.28E-07
SDAD1P1	1.1933	9.99E-10	9.67E-09
EPHA7	1.2009	3.24E-130	1.68E-127
TP53INP1	1.2091	7.08E-153	5.27E-150
SOX4	1.2113	3.51E-145	2.26E-142
FOSL1	1.2144	8.08E-08	6.22E-07
ALDH1A1	1.2172	7.66E-163	7.14E-160

UGT2B28	1.2194	1.02E-09	9.82E-09
SPRY4	1.2205	6.37E-50	6.45E-48
NR4A1	1.2206	1.99E-37	1.20E-35
FNIP2	1.2238	6.26E-76	1.30E-73
RP11-402G3.3	1.2247	1.38E-05	7.61E-05
NETO1	1.2312	4.46E-37	2.61E-35
COL9A2	1.2365	2.62E-11	3.01E-10
CDKN1A	1.2377	5.91E-156	4.78E-153
PPT2	1.239	1.94E-36	1.10E-34
EGR1	1.2418	2.82E-84	7.50E-82
ANTXR1	1.2424	1.27E-31	5.64E-30
FJX1	1.246	4.08E-09	3.70E-08
RP5-1148A21.3	1.2477	6.60E-10	6.52E-09
CSGALNACT1	1.2547	4.79E-26	1.55E-24
UGT2B10	1.2571	1.31E-67	2.20E-65
DNAJB9	1.2631	2.00E-53	2.35E-51
GRHL3	1.2645	2.69E-11	3.08E-10
HCN4	1.2652	3.18E-06	1.93E-05
GPR137C	1.2693	1.78E-19	3.97E-18
SPP1	1.2709	1.65E-06	1.04E-05
DLX5	1.2715	6.26E-06	3.64E-05
NTN4	1.2892	7.42E-07	4.97E-06
RGL1	1.292	2.63E-11	3.02E-10
CHRM3	1.2942	7.61E-23	2.06E-21
ENPP5	1.2988	1.46E-49	1.44E-47
CHGB	1.3027	6.23E-19	1.35E-17
TNFRSF19	1.3055	2.93E-09	2.69E-08
CHIC1	1.3061	1.57E-41	1.13E-39
TLE4	1.3121	2.00E-19	4.46E-18
FSTL1	1.3167	3.92E-33	1.86E-31
TNFSF15	1.3171	4.27E-51	4.58E-49
IL12A	1.325	3.25E-09	2.98E-08
ZNF804A	1.3311	1.07E-07	8.14E-07
EGR4	1.348	1.06E-06	6.94E-06
SPTSSB	1.3503	2.82E-16	4.98E-15
TLE1	1.3512	5.78E-49	5.64E-47
LAMC1	1.3529	1.13E-191	1.41E-188
FN1	1.3536	4.88E-137	2.84E-134
PDE3A	1.3612	1.62E-10	1.70E-09
SLC12A2	1.3655	3.53E-116	1.69E-113
SULF2	1.3706	8.44E-24	2.41E-22
SATB1	1.3805	1.71E-38	1.09E-36
CDKN2B	1.3858	3.16E-20	7.42E-19
FOSB	1.3883	1.80E-18	3.75E-17
CCDC80	1.3908	6.39E-83	1.65E-80

ERBB4	1.4133	2.66E-36	1.50E-34
FAM13C	1.4156	2.26E-24	6.58E-23
LAMA3	1.4164	4.77E-54	5.81E-52
PCDH19	1.4316	4.65E-15	7.56E-14
EGLN3	1.4511	2.03E-10	2.11E-09
SCARA5	1.4584	9.82E-12	1.18E-10
HMGA2	1.4607	7.52E-39	4.87E-37
PRDM1	1.4647	1.34E-11	1.59E-10
LINC00552	1.4662	1.95E-07	1.42E-06
VCAN	1.4676	1.75E-78	3.94E-76
ANKRD29	1.4936	1.79E-39	1.19E-37
GDF15	1.4954	5.35E-112	2.27E-109
GUCY1A3	1.5017	6.04E-67	9.87E-65
EREG	1.502	2.22E-08	1.84E-07
GABARAPL1	1.5049	1.50E-60	2.06E-58
NR4A2	1.5133	5.99E-44	4.89E-42
VGLL3	1.5135	8.57E-12	1.04E-10
DIO2	1.5184	4.45E-10	4.44E-09
DSCAM-AS1	1.5216	7.03E-08	5.45E-07
UGT2B7	1.5431	1.68E-31	7.39E-30
BTG2	1.5643	4.07E-244	9.48E-241
RP11-588K22.2	1.5775	6.77E-29	2.50E-27
BMP2	1.578	6.63E-158	5.61E-155
AREG	1.5906	2.55E-20	6.03E-19
PTGS2	1.5996	8.65E-104	3.04E-101
ADAMTS7	1.6276	2.50E-29	9.47E-28
C8orf4	1.6918	6.28E-22	1.61E-20
EGR3	1.6961	2.14E-18	4.43E-17
GDA	1.6988	4.06E-10	4.08E-09
ARSJ	1.7096	1.49E-25	4.66E-24
PKIA	1.711	1.55E-27	5.39E-26
PDK4	1.7333	9.73E-248	3.02E-244
IGFBP3	1.7955	0	0
FST	1.8202	3.27E-99	1.07E-96
AGR2	1.8239	1.79E-103	6.19E-101
DUSP4	2.0201	2.26E-35	1.22E-33
LXN	2.0247	3.16E-232	4.90E-229
ATOH1	2.0938	4.40E-15	7.15E-14
BASP1	2.0991	4.57E-44	3.79E-42
F3	2.1611	3.99E-153	3.10E-150
A2M	2.2262	8.74E-81	2.12E-78
DKK1	2.3421	0	0
MAPK4	2.3501	1.24E-204	1.78E-201
STEAP4	2.382	1.83E-48	1.76E-46
TNFRSF11B	2.4077	1.42E-175	1.47E-172

FGB	2.415	1.19E-24	3.50E-23

Table S3. Downregulated and upregulated genes upon SMAD4 KD in Rv1 cells

GeneName	log2FoldChange	pvalue	padj
USMG5	-4.0571	0	0
NIPSNAP1	-3.7436	0	0
TMPRSS4	-3.4047	3.22E-65	1.42E-63
POMGNT1	-3.4032	0	0
APOO	-3.3215	2.63E-112	3.09E-110
MT1E	-3.1475	6.21E-252	3.68E-249
COX5A	-3.0239	0	0
GCA	-2.8977	9.47E-174	2.49E-171
ANKRD22	-2.871	1.14E-41	2.76E-40
IGSF8	-2.8689	2.04E-259	1.28E-256
MT2A	-2.865	0	0
MFSD3	-2.8373	7.68E-223	3.46E-220
NENF	-2.785	1.12E-280	9.31E-278
TM4SF20	-2.7567	4.30E-19	4.30E-18
C15orf38	-2.7486	1.24E-147	2.44E-145
CYB5R3	-2.7476	5.74E-303	5.96E-300
ANKS4B	-2.6969	1.92E-17	1.76E-16
GM2A	-2.6781	9.02E-146	1.70E-143
TMEM9B	-2.6781	3.66E-200	1.27E-197
SURF1	-2.6769	1.11E-213	4.72E-211
CGNL1	-2.6768	1.16E-23	1.43E-22
PHEX	-2.6381	7.84E-39	1.70E-37
FLII	-2.6353	0	0
PROM2	-2.6327	0	0
MYBL2	-2.6313	1.65E-268	1.18E-265
MT1F	-2.629	2.50E-16	2.14E-15
ALDH3B2	-2.6017	1.61E-46	4.54E-45
STX10	-2.593	3.10E-146	5.90E-144
FLG	-2.5522	1.12E-65	5.05E-64
ATG4D	-2.5501	4.61E-169	1.14E-166
MSN	-2.5336	5.28E-120	6.72E-118
SLC44A2	-2.5262	0	0
GMFB	-2.5219	0	0
RP11-568K15.1	-2.5116	2.08E-124	2.76E-122
PAK4	-2.4878	3.42E-250	1.87E-247
GNAI2	-2.4795	0	0
ZNF28	-2.4721	4.29E-95	3.74E-93
RASSF3	-2.4524	0	0
FSCN1	-2.419	1.56E-10	9.12E-10
METTL2A	-2.415	7.70E-123	1.00E-120
DPM2	-2.4136	1.23E-168	3.00E-166
PLA2G4F	-2.3967	3.67E-103	3.57E-101

OAF	-2.3966	3.75E-96	3.34E-94
PCTP	-2.3964	6.51E-108	6.92E-106
FGFRL1	-2.3745	2.49E-93	2.08E-91
LGALS1	-2.353	0	0
SH3BGRL2	-2.3509	1.54E-146	2.95E-144
EIF3C	-2.3412	2.95E-53	9.81E-52
WNT7B	-2.338	1.67E-24	2.13E-23
HHLA2	-2.3355	3.34E-21	3.67E-20
AAMP	-2.3278	0	0
WNT10B	-2.3181	1.18E-128	1.66E-126
KLK11	-2.3169	8.31E-141	1.45E-138
EXOSC6	-2.3119	2.85E-109	3.13E-107
FAM134B	-2.3085	1.51E-63	6.42E-62
RP11-551L14.1	-2.2906	1.10E-18	1.07E-17
ALOX15	-2.2806	5.11E-111	5.92E-109
MRPL3	-2.2806	0	0
COL5A2	-2.2804	1.61E-201	5.65E-199
C12orf76	-2.2684	1.61E-41	3.87E-40
RPIA	-2.2593	1.20E-149	2.47E-147
DAK	-2.2586	8.06E-270	5.97E-267
PPIL3	-2.2582	4.02E-53	1.33E-51
C19orf57	-2.2566	9.58E-19	9.38E-18
RP13-467H17.1	-2.2532	1.31E-08	6.67E-08
SORBS3	-2.232	2.49E-114	2.99E-112
CDH17	-2.2273	3.13E-108	3.39E-106
TP53I11	-2.2269	2.02E-169	5.04E-167
IL36RN	-2.223	9.11E-12	5.85E-11
CLRN3	-2.2173	9.73E-19	9.53E-18
MPZL2	-2.2148	6.51E-110	7.26E-108
TMOD2	-2.2106	8.48E-94	7.24E-92
LMNB2	-2.1847	0	0
EIF6	-2.1514	1.47E-151	3.04E-149
ZNF702P	-2.144	7.58E-70	3.85E-68
DCAF12	-2.1405	7.52E-275	5.78E-272
IFT43	-2.1248	1.37E-61	5.61E-60
R3HCC1	-2.1229	3.21E-170	8.21E-168
VDAC3	-2.1208	0	0
SLC26A3	-2.1185	7.16E-27	1.02E-25
SSU72	-2.111	0	0
NPC2	-2.1096	9.70E-161	2.26E-158
COASY	-2.0915	6.30E-251	3.63E-248
HID1	-2.0906	3.60E-88	2.68E-86
PPP1R7	-2.0885	5.84E-187	1.75E-184
COQ2	-2.0762	2.16E-71	1.15E-69
NARS	-2.0762	5.53E-277	4.41E-274

ZNF468	-2.0733	2.22E-83	1.50E-81
HIST1H3H	-2.0655	5.40E-16	4.54E-15
RP11-480I12.5	-2.0556	4.16E-23	4.99E-22
TRGC1	-2.0549	5.23E-54	1.78E-52
KCNQ2	-2.0514	1.61E-16	1.39E-15
NUP210	-2.0499	5.96E-246	3.17E-243
MED24	-2.0454	3.27E-221	1.41E-218
LMAN2L	-2.0447	2.49E-63	1.05E-61
SV2C	-2.0361	3.13E-07	1.41E-06
HIST1H2AI	-2.0345	1.91E-10	1.11E-09
CRYL1	-2.0295	2.40E-115	2.91E-113
ANAPC13	-2.0168	2.31E-130	3.40E-128
NICN1	-2.014	5.19E-57	1.87E-55
CDCA5	-2.0127	4.83E-160	1.11E-157
IFRD2	-2.0127	4.39E-225	2.02E-222
PKMYT1	-1.9951	5.95E-108	6.36E-106
RP11-167H9.6	-1.9873	6.56E-11	3.95E-10
FAM213A	-1.9871	2.79E-134	4.38E-132
AP000593.5	-1.9861	6.90E-07	2.99E-06
LRRC37A16P	-1.9762	2.42E-136	3.93E-134
CDC42SE1	-1.972	4.10E-296	3.86E-293
AOX1	-1.9712	1.11E-22	1.30E-21
CNIH2	-1.9704	7.03E-13	4.89E-12
USH1C	-1.9695	1.20E-08	6.13E-08
SERINC5	-1.9679	1.80E-39	4.05E-38
NOX5	-1.9657	1.31E-23	1.61E-22
NDUFB3	-1.9574	3.31E-130	4.83E-128
SLC16A1	-1.9488	1.05E-222	4.65E-220
HMOX1	-1.9474	6.54E-26	9.00E-25
PTP4A2	-1.9457	6.88E-264	4.46E-261
MESP1	-1.9426	1.04E-18	1.02E-17
CDK16	-1.9426	2.57E-250	1.44E-247
PDCL3	-1.9414	1.67E-126	2.33E-124
GRAMD1C	-1.9369	3.33E-39	7.37E-38
MIS18A	-1.9311	5.94E-92	4.83E-90
ANXA5	-1.9299	1.31E-156	2.89E-154
C3orf70	-1.9254	1.34E-32	2.37E-31
METTL2B	-1.9207	4.25E-148	8.47E-146
ASPRV1	-1.9162	2.56E-08	1.27E-07
KCNC2	-1.9153	3.87E-18	3.64E-17
PMEL	-1.9081	9.85E-11	5.85E-10
PCED1B	-1.9058	3.71E-48	1.09E-46
PLEK2	-1.9034	1.44E-26	2.02E-25
GS1-358P8.4	-1.8994	2.91E-35	5.59E-34
SLC25A23	-1.8987	1.12E-242	5.68E-240

ULK3	-1.8965	2.37E-135	3.81E-133
MAPK3	-1.8892	3.32E-107	3.46E-105
SPCS3	-1.8886	4.14E-206	1.59E-203
CTA-445C9.14	-1.8805	3.83E-17	3.44E-16
MAPRE1	-1.8797	7.54E-207	2.95E-204
ZNF723	-1.8785	8.72E-11	5.20E-10
CCDC160	-1.8716	5.85E-14	4.35E-13
MB	-1.8713	6.75E-101	6.42E-99
KREMEN1	-1.8675	1.46E-39	3.29E-38
LUM	-1.8631	8.62E-22	9.69E-21
CEP19	-1.8615	1.20E-15	9.91E-15
RP1-180E22.3	-1.8528	2.59E-18	2.47E-17
PIM1	-1.8524	6.83E-15	5.38E-14
UHMK1	-1.8421	3.97E-296	3.86E-293
NCMAP	-1.8334	8.20E-16	6.83E-15
CSK	-1.8286	1.31E-130	1.96E-128
RPH3AL	-1.8258	1.46E-06	6.13E-06
MAPRE3	-1.8216	3.86E-52	1.25E-50
RMI2	-1.8216	2.54E-70	1.30E-68
AC005537.2	-1.8023	2.53E-06	1.04E-05
SEMA4A	-1.8021	1.02E-285	8.83E-283
ATF5	-1.7988	2.22E-102	2.15E-100
MIR4453	-1.7974	3.88E-27	5.58E-26
CP	-1.7947	2.07E-16	1.77E-15
FKTN	-1.7943	1.10E-117	1.36E-115
ZNF678	-1.7913	6.88E-105	6.93E-103
LRRC8A	-1.7907	2.15E-226	1.04E-223
FAM189A1	-1.7823	3.76E-58	1.40E-56
LRRC59	-1.778	1.50E-205	5.65E-203
CAV1	-1.7768	4.17E-39	9.19E-38
CA12	-1.7727	1.41E-286	1.27E-283
C15orf41	-1.7678	2.67E-15	2.15E-14
TK2	-1.7673	2.43E-58	9.13E-57
SDC1	-1.7638	1.26E-164	3.05E-162
HAO1	-1.7619	8.94E-10	4.97E-09
E2F1	-1.7593	1.23E-110	1.39E-108
SYTL4	-1.7538	2.65E-15	2.14E-14
LIPM	-1.7534	1.64E-05	6.18E-05
EIF3CL	-1.7533	1.80E-08	9.05E-08
ABCC6P2	-1.7469	1.07E-06	4.55E-06
KAT2A	-1.7444	2.81E-212	1.14E-209
TNFRSF10D	-1.741	8.70E-94	7.37E-92
SRPRB	-1.7395	1.42E-267	9.84E-265
PCSK9	-1.7388	1.37E-06	5.76E-06
SYT7	-1.7383	5.97E-99	5.60E-97

MPP2	-1.738	2.94E-38	6.25E-37
CMC2	-1.7376	3.38E-73	1.86E-71
GSTM3	-1.7358	1.38E-34	2.61E-33
TMEM180	-1.7343	1.36E-65	6.07E-64
SMAD4	-1.7337	6.72E-84	4.58E-82
SECISBP2	-1.733	1.03E-152	2.17E-150
AGXT2L1	-1.7278	4.55E-08	2.21E-07
GRB2	-1.7273	2.07E-178	5.88E-176
STAT2	-1.7265	8.04E-71	4.18E-69
PAN2	-1.7257	2.65E-186	7.86E-184
RP11-122G18.5	-1.7243	1.10E-45	3.01E-44
CCDC135	-1.7237	1.87E-11	1.17E-10
EHHADH	-1.7235	3.93E-13	2.78E-12
CETN3	-1.7231	3.39E-91	2.70E-89
VARS2	-1.7229	6.18E-111	7.01E-109
ENO2	-1.7226	5.01E-185	1.46E-182
DDX21	-1.7211	6.15E-258	3.75E-255
C1orf192	-1.7179	2.87E-68	1.41E-66
FIBIN	-1.7178	1.82E-05	6.81E-05
HLA-E	-1.7177	5.90E-93	4.89E-91
RASGEF1C	-1.7174	1.16E-06	4.91E-06
GCLM	-1.7162	1.38E-84	9.55E-83
C19orf21	-1.7069	9.17E-40	2.08E-38
LINC00870	-1.7064	4.99E-06	1.99E-05
MRAS	-1.7057	3.53E-24	4.46E-23
RAB4B	-1.7023	2.59E-07	1.17E-06
VEPH1	-1.7012	4.67E-22	5.34E-21
ADH6	-1.7011	2.12E-09	1.14E-08
GLB1L2	-1.6997	1.10E-86	7.99E-85
ISCA2	-1.6988	1.14E-25	1.55E-24
PM20D2	-1.698	4.44E-67	2.07E-65
OPTN	-1.6974	9.14E-10	5.08E-09
MDFIC	-1.6946	7.66E-147	1.49E-144
RNF135	-1.6911	2.62E-67	1.24E-65
WDR83OS	-1.6894	2.95E-32	5.14E-31
RP1-223E5.4	-1.6847	1.85E-10	1.07E-09
TCF19	-1.6846	6.46E-44	1.68E-42
PAGR1	-1.6819	9.17E-66	4.15E-64
CTD-2008P7.8	-1.6817	9.91E-09	5.09E-08
VPS4A	-1.6792	4.86E-109	5.30E-107
STEAP1	-1.6765	8.65E-130	1.25E-127
EDEM1	-1.6723	5.50E-164	1.31E-161
AL050303.1	-1.6678	1.01E-07	4.77E-07
DNAJC27-AS1	-1.6665	2.04E-23	2.50E-22
SEMA4F	-1.6634	1.56E-24	2.00E-23

GSTZ1	-1.6634	4.67E-79	2.92E-77
LIMK1	-1.6564	1.04E-52	3.41E-51
CTSH	-1.6552	5.87E-92	4.80E-90
AIDA	-1.6548	7.62E-112	8.93E-110
RP11-43F13.3	-1.6544	9.22E-13	6.36E-12
SF3B4	-1.6527	4.40E-194	1.45E-191
SAMD14	-1.6517	2.93E-23	3.56E-22
TTLL1	-1.6486	3.08E-10	1.77E-09
FUT4	-1.6454	2.23E-17	2.03E-16
ZNF812	-1.6428	1.91E-17	1.75E-16
ZNF716	-1.6412	5.85E-18	5.45E-17
TMCC2	-1.6397	1.31E-08	6.66E-08
PIGW	-1.6396	1.12E-55	3.96E-54
EIF2D	-1.6393	4.95E-159	1.12E-156
PEG10	-1.6358	7.11E-266	4.76E-263
CRIP3	-1.6338	4.92E-05	0.00017543
TMCC3	-1.6334	4.07E-36	8.09E-35
GPN2	-1.6333	1.77E-67	8.44E-66
TMEM92	-1.6311	3.84E-05	0.00013858
C1orf106	-1.6287	2.19E-75	1.28E-73
FUK	-1.6282	4.26E-64	1.83E-62
FAM45A	-1.6257	1.96E-83	1.33E-81
ARRB2	-1.6251	1.28E-81	8.33E-80
SCAMP4	-1.6237	1.20E-78	7.42E-77
TARBP2	-1.6226	7.47E-74	4.18E-72
KIAA0195	-1.6191	1.28E-65	5.75E-64
PPP2R1B	-1.6185	3.94E-104	3.91E-102
RP11-160O5.1	-1.6182	6.74E-06	2.65E-05
MORN2	-1.6167	5.13E-22	5.85E-21
LCP1	-1.6164	4.82E-34	8.89E-33
CD99L2	-1.6136	1.22E-74	7.04E-73
ULK4P2	-1.6126	2.11E-05	7.85E-05
EPT1	-1.6122	4.53E-189	1.42E-186
TSPAN13	-1.612	9.08E-80	5.81E-78
MMRN2	-1.6117	2.85E-07	1.28E-06
M6PR	-1.6088	6.07E-111	6.92E-109
KLK1	-1.6076	1.92E-75	1.13E-73
AP003733.1	-1.6073	6.04E-06	2.39E-05
CTC-429P9.1	-1.6037	3.88E-09	2.06E-08
PTK2B	-1.6032	5.20E-182	1.50E-179
CCDC43	-1.603	2.17E-47	6.22E-46
SCIN	-1.6022	3.89E-22	4.45E-21
MAST3	-1.6008	1.93E-39	4.32E-38
CAPG	-1.5987	2.78E-39	6.19E-38
ZNF681	-1.5985	2.14E-71	1.14E-69

PDHB	-1.5982	2.89E-191	9.37E-189
ACTG1P1	-1.5949	2.13E-07	9.72E-07
PEX26	-1.5944	8.63E-139	1.46E-136
COPG1	-1.5943	1.17E-244	6.08E-242
ARHGEF1	-1.5938	2.85E-72	1.55E-70
ENTPD1	-1.5933	1.10E-53	3.73E-52
CHAC1	-1.5926	1.03E-10	6.09E-10
RWDD2B	-1.5909	2.63E-53	8.76E-52
ZNF721	-1.5886	6.66E-78	4.10E-76
DMC1	-1.5833	8.20E-16	6.83E-15
RSPO3	-1.582	2.13E-05	7.90E-05
FOLR1	-1.5797	1.85E-05	6.92E-05
TP53TG3D	-1.5785	9.10E-05	0.00031365
RPLP0P2	-1.5759	4.62E-05	0.00016507
LRRC45	-1.5736	9.13E-66	4.14E-64
PREX1	-1.5721	8.45E-08	4.00E-07
MRPL37	-1.5712	8.54E-212	3.41E-209
KATNB1	-1.5699	1.05E-78	6.53E-77
TRAM1	-1.5697	3.64E-129	5.17E-127
PPDPF	-1.569	1.69E-89	1.31E-87
F11R	-1.5665	5.21E-114	6.17E-112
MAP1A	-1.5647	3.91E-12	2.56E-11
PALM3	-1.5628	6.59E-06	2.60E-05
LTBP4	-1.5602	1.53E-54	5.29E-53
PCYOX1	-1.5522	4.54E-76	2.70E-74
NET1	-1.5522	8.02E-178	2.22E-175
IARS	-1.551	4.93E-196	1.65E-193
PRPF8	-1.5474	7.23E-202	2.63E-199
PPP1R37	-1.547	2.89E-73	1.59E-71
TCEAL1	-1.5467	1.07E-34	2.03E-33
IGFBPL1	-1.5453	3.64E-07	1.62E-06
TTL	-1.5427	2.22E-64	9.58E-63
CACFD1	-1.5419	5.06E-54	1.72E-52
ZNRD1	-1.5393	4.61E-18	4.32E-17
SLC17A4	-1.538	4.98E-14	3.73E-13
FLG-AS1	-1.5368	2.78E-18	2.64E-17
AIM1	-1.5362	2.02E-71	1.08E-69
TRIM21	-1.5338	4.11E-20	4.32E-19
ZNF93	-1.5305	2.35E-35	4.55E-34
ASRGL1	-1.5304	8.88E-18	8.22E-17
ACBD3	-1.5275	5.00E-175	1.36E-172
RP11-27I1.2	-1.526	2.07E-22	2.40E-21
PEMT	-1.5245	1.34E-23	1.65E-22
MLLT6	-1.5237	1.71E-213	7.10E-211
EPSTI1	-1.5227	2.73E-06	1.12E-05

PAPOLA	-1.5219	3.88E-189	1.24E-186
NES	-1.5192	3.70E-27	5.33E-26
WARS2	-1.5184	6.18E-53	2.04E-51
PICK1	-1.5167	9.64E-58	3.53E-56
PAFAH1B2	-1.5154	1.53E-110	1.72E-108
CLCN2	-1.5143	9.13E-62	3.74E-60
NOC4L	-1.5117	8.32E-68	4.02E-66
ESCO2	-1.5107	1.45E-69	7.37E-68
OAS3	-1.5085	3.24E-46	9.01E-45
MIR210HG	-1.5031	2.09E-07	9.51E-07
RP11-313J2.1	-1.503	2.30E-17	2.09E-16
RP11-977G19.12	-1.5019	2.62E-07	1.19E-06
RBP7	-1.5015	1.24E-11	7.88E-11
RMND5B	-1.501	8.57E-75	4.95E-73
AP2S1	-1.5006	1.67E-152	3.51E-150
MAGEA1	-1.5002	2.23E-130	3.31E-128
CLIC3	-1.4999	9.26E-15	7.25E-14
WNT8B	-1.4967	5.66E-17	5.01E-16
ANKRD52	-1.4959	6.26E-145	1.17E-142
ELN	-1.4951	1.10E-05	4.21E-05
COL3A1	-1.493	1.37E-09	7.47E-09
SLC52A3	-1.491	5.66E-36	1.12E-34
ZDHHC4	-1.4903	1.03E-64	4.51E-63
C5orf28	-1.4873	1.16E-37	2.39E-36
GPR133	-1.4862	0.00025107	0.00081488
FGFR3	-1.4855	1.31E-84	9.15E-83
ZCCHC24	-1.4846	1.01E-20	1.09E-19
UBXN6	-1.484	1.97E-90	1.54E-88
MPRIP	-1.4825	2.66E-142	4.71E-140
RP11-321F8.4	-1.4793	3.14E-09	1.68E-08
MYO1A	-1.4789	1.37E-56	4.92E-55
RP11-498C9.15	-1.4765	7.63E-06	2.98E-05
WDR34	-1.4758	8.52E-94	7.24E-92
ENHO	-1.4751	7.46E-11	4.48E-10
SEL1L3	-1.4751	4.10E-84	2.82E-82
C6orf25	-1.4744	1.54E-06	6.42E-06
YDJC	-1.4729	1.25E-70	6.47E-69
ATP6V0E2	-1.4729	1.01E-152	2.17E-150
LRP5	-1.4722	1.99E-89	1.52E-87
CCDC74A	-1.4721	5.98E-111	6.85E-109
RP11-1148L6.5	-1.4719	4.75E-12	3.10E-11
CCDC155	-1.47	0.00015022	0.00050331
STARD3NL	-1.4693	2.72E-100	2.57E-98
LAMB4	-1.4689	0.00020766	0.0006833
EPS8L3	-1.4668	1.09E-09	6.01E-09

CPNE5	-1.4654	0.0001928	0.00063722
KLHDC10	-1.4644	2.10E-89	1.60E-87
PEX7	-1.4643	1.09E-35	2.14E-34
FAM222A	-1.4633	2.46E-38	5.25E-37
TMEM192	-1.463	1.58E-109	1.74E-107
ABCF2	-1.4616	1.01E-199	3.43E-197
STC1	-1.4614	0.00015936	0.00053248
MORC4	-1.4609	1.20E-70	6.21E-69
PSD4	-1.4602	7.77E-108	8.21E-106
TMEM216	-1.4559	7.26E-27	1.03E-25
ZNF728	-1.4542	2.83E-22	3.25E-21
RHBDD1	-1.4524	1.49E-90	1.17E-88
CDS1	-1.4519	3.87E-80	2.48E-78
SLC16A3	-1.4504	6.02E-46	1.66E-44
GAL3ST1	-1.45	1.77E-21	1.96E-20
GTF2H3	-1.4498	6.51E-132	1.00E-129
ASCC2	-1.4493	4.10E-71	2.16E-69
PUSL1	-1.4481	5.81E-38	1.22E-36
GCC1	-1.4478	7.16E-76	4.25E-74
DTX4	-1.4398	1.30E-116	1.59E-114
COL1A1	-1.4379	8.34E-15	6.53E-14
ULK4P3	-1.4338	0.00041914	0.0013203
LDOC1	-1.4333	4.58E-55	1.60E-53
FREM1	-1.4331	2.45E-12	1.63E-11
FAM127A	-1.4302	5.18E-52	1.68E-50
RP11-64K12.2	-1.4289	3.28E-17	2.96E-16
PODXL	-1.4254	9.83E-139	1.64E-136
MTA1	-1.4239	1.64E-119	2.07E-117
ZFAND2B	-1.4234	6.98E-39	1.52E-37
RP11-44F14.8	-1.4186	1.18E-16	1.03E-15
TOMM40L	-1.4186	7.01E-92	5.66E-90
TM4SF5	-1.4178	1.93E-11	1.20E-10
ARHGAP17	-1.4173	4.13E-43	1.04E-41
CXorf38	-1.4155	2.26E-44	5.98E-43
RP11-72M17.1	-1.4153	0.00049705	0.0015493
SLC40A1	-1.4148	3.41E-74	1.95E-72
FAM126A	-1.4142	2.98E-61	1.21E-59
TTC19	-1.4137	9.47E-112	1.10E-109
UHRF1	-1.4118	8.55E-60	3.32E-58
TBXA2R	-1.41	4.79E-11	2.91E-10
GNB5	-1.4081	1.14E-15	9.40E-15
HNRNPAB	-1.4079	2.22E-178	6.23E-176
TSPAN11	-1.4053	2.25E-33	4.08E-32
UBE2S	-1.405	6.22E-135	9.85E-133
ARAP3	-1.4049	0.00055658	0.0017185

FPGS	-1.401	8.58E-61	3.43E-59
TEX261	-1.4003	1.53E-125	2.10E-123
LL22NC03-63E9.3	-1.3989	0.00054475	0.001685
DISP2	-1.398	1.99E-05	7.42E-05
DPH2	-1.3976	1.16E-86	8.41E-85
AC002064.4	-1.3972	0.00059079	0.0018161
CTC-451A6.4	-1.3958	0.00013644	0.00045922
ZNF141	-1.3957	1.05E-42	2.62E-41
ATP6V1C2	-1.3954	3.36E-38	7.09E-37
NXT2	-1.3934	3.76E-29	5.84E-28
MYD88	-1.3907	1.06E-85	7.55E-84
OXA1L	-1.3903	6.86E-139	1.18E-136
AMHR2	-1.3898	8.45E-11	5.04E-10
RP11-3N2.1	-1.3888	1.72E-08	8.65E-08
RNLS	-1.3888	1.89E-12	1.28E-11
ABCC6	-1.3873	5.97E-17	5.28E-16
ZBTB42	-1.3861	5.08E-42	1.24E-40
TGM2	-1.385	1.65E-05	6.21E-05
UNG	-1.3849	3.69E-158	8.24E-156
TBC1D13	-1.3845	1.42E-63	6.05E-62
IRF5	-1.3825	6.45E-43	1.62E-41
CLDN2	-1.382	0.00060693	0.0018607
ANXA1	-1.3814	1.73E-12	1.17E-11
ICMT	-1.3812	9.91E-160	2.26E-157
AC110619.2	-1.3808	8.25E-10	4.60E-09
VARS	-1.3799	4.78E-142	8.41E-140
SYK	-1.3791	4.85E-94	4.16E-92
ZNF729	-1.379	6.62E-13	4.61E-12
RP11-393M11.2	-1.3788	6.73E-06	2.65E-05
PRKRIR	-1.3783	4.91E-83	3.29E-81
STRIP2	-1.3782	1.73E-12	1.17E-11
MFHAS1	-1.3776	7.96E-78	4.89E-76
ANKRD36BP1	-1.3772	3.69E-38	7.79E-37
SLC45A3	-1.3772	1.52E-55	5.34E-54
GLDC	-1.3767	1.88E-50	5.93E-49
ZNF511	-1.376	6.01E-25	7.87E-24
RP11-622K12.1	-1.376	2.12E-60	8.36E-59
SDHC	-1.3756	1.34E-201	4.78E-199
C6orf120	-1.3752	7.45E-71	3.89E-69
ALG2	-1.3748	2.25E-64	9.70E-63
IL22RA1	-1.3734	0.00073837	0.0022349
PSMG3-AS1	-1.3709	2.25E-35	4.36E-34
KLHL12	-1.3696	2.29E-120	2.94E-118
NDUFB8	-1.3676	0.00033501	0.0010692
AL133493.2	-1.3671	1.06E-18	1.04E-17

FOXN1	-1.3639	0.00079414	0.002394
C1RL	-1.3637	2.35E-06	9.63E-06
LRRC20	-1.3633	3.09E-34	5.77E-33
LRRC26	-1.3622	2.15E-09	1.16E-08
ACSS1	-1.3616	3.28E-08	1.61E-07
TEX19	-1.361	4.61E-06	1.84E-05
VTA1	-1.361	1.09E-139	1.89E-137
AL449209.1	-1.3575	7.15E-08	3.41E-07
RP11-498E2.7	-1.357	1.24E-08	6.32E-08
ALOX15B	-1.3566	1.51E-06	6.31E-06
SLC19A2	-1.3563	1.41E-85	9.96E-84
CRISP3	-1.3559	0.00041888	0.0013199
SLFN11	-1.3553	3.56E-21	3.90E-20
RAB31	-1.3552	9.93E-17	8.67E-16
C5orf49	-1.3551	0.00079215	0.0023883
AC093323.3	-1.3549	1.49E-96	1.35E-94
CTD-2192J16.20	-1.3545	0.00013057	0.00044095
PCDH20	-1.3527	8.79E-87	6.42E-85
ADH1B	-1.3526	1.21E-08	6.18E-08
ABHD2	-1.3526	2.97E-125	4.02E-123
LINC00674	-1.3514	2.68E-25	3.57E-24
RP11-104L21.2	-1.3504	0.00013177	0.00044473
URM1	-1.3501	2.84E-75	1.66E-73
AC139100.2	-1.3498	2.78E-15	2.24E-14
KAZALD1	-1.3495	1.70E-53	5.71E-52
RP11-57H14.4	-1.3484	2.34E-12	1.56E-11
GPAM	-1.3478	1.02E-41	2.49E-40
IFIT3	-1.3451	0.00054283	0.0016798
ACTR2	-1.345	1.03E-123	1.35E-121
TBRG4	-1.3445	2.23E-132	3.48E-130
LCK	-1.344	0.00053408	0.0016558
MED15	-1.3437	4.65E-56	1.66E-54
BOK	-1.3433	1.68E-27	2.45E-26
USP5	-1.3431	4.55E-174	1.21E-171
BCAM	-1.342	3.01E-49	9.12E-48
FAM178B	-1.3419	2.31E-22	2.66E-21
TMPRSS2	-1.3419	4.28E-40	9.79E-39
NAT10	-1.3416	1.83E-125	2.50E-123
TPM3	-1.3405	9.50E-189	2.94E-186
TMEM52	-1.3395	5.64E-11	3.41E-10
VMA21	-1.3394	2.76E-59	1.06E-57
HBQ1	-1.3387	4.44E-10	2.53E-09
PLEKHN1	-1.3384	7.85E-16	6.55E-15
RP11-436A20.4	-1.3374	8.09E-06	3.15E-05
SYT15	-1.3367	1.92E-19	1.95E-18

AAAS	-1.3362	5.28E-96	4.68E-94
MAP2K4	-1.3355	7.23E-82	4.76E-80
TAF9B	-1.3352	3.01E-82	2.00E-80
FSD1L	-1.335	3.95E-26	5.47E-25
SLC37A4	-1.334	2.03E-47	5.85E-46
LRRC23	-1.3335	7.19E-15	5.65E-14
NCAPD2	-1.333	1.72E-131	2.62E-129
MAP3K14-AS1	-1.3329	6.09E-07	2.65E-06
HIST1H2AC	-1.3293	3.26E-25	4.31E-24
LRRC31	-1.327	2.05E-05	7.64E-05
TTC7B	-1.3252	1.69E-34	3.19E-33
RP11-255H23.2	-1.3248	5.75E-06	2.28E-05
MAGED1	-1.323	1.29E-143	2.37E-141
AK7	-1.3222	5.31E-17	4.72E-16
P2RX7	-1.3217	1.66E-06	6.92E-06
TSPAN15	-1.321	1.91E-17	1.75E-16
LINC00663	-1.3208	3.31E-09	1.76E-08
CENPH	-1.3202	6.56E-96	5.77E-94
POPDC3	-1.3192	7.06E-06	2.77E-05
CDIPT	-1.3185	2.68E-85	1.88E-83
ZNF138	-1.3182	9.81E-39	2.12E-37
PIGZ	-1.3166	5.00E-14	3.74E-13
RAB28	-1.3161	5.66E-31	9.37E-30
PCID2	-1.3143	1.47E-57	5.37E-56
ABHD17C	-1.314	1.85E-50	5.85E-49
CAPN5	-1.3137	2.14E-18	2.05E-17
IL23R	-1.3131	0.0010596	0.0031292
RP11-353B9.1	-1.3126	0.00059439	0.0018252
NEURL3	-1.3122	1.81E-06	7.51E-06
RP11-92G12.3	-1.3119	0.0001228	0.00041609
FBP2	-1.3118	0.0012583	0.0036665
CTA-292E10.6	-1.3115	0.00024367	0.00079173
SUV39H1	-1.3112	2.10E-07	9.56E-07
SVEP1	-1.311	1.07E-08	5.48E-08
MAP4K2	-1.3107	2.70E-65	1.19E-63
SYNGR4	-1.3098	0.0012825	0.0037306
CYP27B1	-1.3085	5.80E-06	2.30E-05
LGALS3BP	-1.3081	4.57E-08	2.21E-07
ZNF876P	-1.3078	0.0006441	0.0019665
RP11-1348G14.4	-1.3056	0.00078229	0.002361
TTYH2	-1.3056	5.89E-33	1.05E-31
CDC26	-1.3043	2.07E-12	1.39E-11
SLC6A8	-1.3039	1.87E-131	2.83E-129
HIST1H2AJ	-1.3034	0.0013414	0.0038817
MOB3A	-1.3032	5.26E-44	1.37E-42

SLC10A4	-1.3004	0.0009163	0.002734
TPMT	-1.2998	1.02E-63	4.37E-62
PRUNE2	-1.2996	2.34E-67	1.11E-65
DENND4B	-1.2995	3.68E-92	3.03E-90
KIAA0319L	-1.2993	6.39E-102	6.13E-100
KAT2B	-1.2992	6.69E-44	1.73E-42
COMT	-1.2978	1.76E-163	4.15E-161
ZNF624	-1.296	1.07E-10	6.34E-10
EPHX1	-1.2955	1.55E-100	1.47E-98
ARL6IP6	-1.2931	6.61E-23	7.82E-22
HIST1H2AE	-1.2927	0.0013184	0.0038215
ACSS3	-1.2926	0.00073178	0.0022182
ZNF616	-1.2915	4.49E-28	6.70E-27
RP11-848P1.2	-1.2914	5.45E-08	2.63E-07
NDRG3	-1.2908	3.78E-50	1.18E-48
CDK18	-1.2905	3.99E-45	1.08E-43
RP11-307C12.11	-1.2898	0.00063575	0.0019425
RAB7L1	-1.2895	1.21E-90	9.50E-89
NTRK2	-1.2874	0.0012885	0.0037427
TK1	-1.2855	4.51E-91	3.56E-89
SLC6A17	-1.2823	8.45E-06	3.29E-05
TUBA3FP	-1.2814	0.00025485	0.00082599
LRP4	-1.2803	0.001595	0.0045654
GSTO2	-1.2802	4.21E-48	1.23E-46
B3GAT3	-1.2792	9.74E-63	4.06E-61
ZNF736	-1.2789	1.78E-25	2.39E-24
MON1B	-1.2778	3.80E-74	2.16E-72
H2AFX	-1.2752	6.71E-74	3.77E-72
TMEM169	-1.2746	0.0010873	0.0032037
KDELR2	-1.2742	3.45E-143	6.21E-141
AC132872.2	-1.2741	2.61E-05	9.59E-05
COQ5	-1.2734	6.94E-74	3.89E-72
NIF3L1	-1.2725	2.26E-49	6.88E-48
RP3-322G13.5	-1.2721	0.00092397	0.0027557
CDCA7	-1.2721	5.80E-49	1.74E-47
C1orf115	-1.2708	7.09E-204	2.63E-201
NDE1	-1.2694	1.51E-36	3.03E-35
ALDH3B1	-1.2679	3.11E-29	4.86E-28
ELOVL5	-1.2678	6.54E-125	8.75E-123
PDK3	-1.2675	8.23E-19	8.10E-18
SDC4	-1.2674	2.25E-26	3.14E-25
COL21A1	-1.2673	1.74E-07	7.97E-07
SLC43A3	-1.2661	1.60E-33	2.92E-32
LGR5	-1.2626	5.16E-40	1.18E-38
ATG2A	-1.2611	3.04E-45	8.22E-44

HIRIP3	-1.2584	1.97E-37	4.05E-36
CTD-2196E14.4	-1.2576	5.04E-07	2.21E-06
TMED4	-1.2575	3.54E-188	1.08E-185
FAM189B	-1.257	2.49E-79	1.57E-77
RPF2	-1.2558	1.78E-67	8.45E-66
RP11-46H11.12	-1.2549	0.0014722	0.0042308
SAMD5	-1.2544	0.0020239	0.0056963
IDH1	-1.2536	8.07E-149	1.63E-146
KLHL9	-1.252	1.61E-85	1.14E-83
CLUH	-1.2508	8.25E-106	8.35E-104
UBAC2-AS1	-1.2497	6.98E-08	3.33E-07
LSM12	-1.2493	5.82E-63	2.45E-61
PLSCR1	-1.2488	2.60E-37	5.34E-36
CYP2C9	-1.2451	0.0019516	0.0055054
LINC00630	-1.245	7.44E-09	3.86E-08
DCTN4	-1.2427	1.72E-93	1.45E-91
AC110619.1	-1.2407	0.00022995	0.0007501
GEMIN4	-1.2406	8.53E-76	5.04E-74
HTR3A	-1.2405	0.002271	0.0063435
TMEM246	-1.24	0.002267	0.0063349
SMIM19	-1.2397	3.66E-46	1.02E-44
CORO2A	-1.2381	7.04E-21	7.62E-20
CTA-217C2.1	-1.2349	3.24E-12	2.14E-11
RP11-366M4.3	-1.2341	0.0019329	0.0054588
THAP6	-1.2337	3.64E-28	5.45E-27
UBA3	-1.2322	3.20E-58	1.20E-56
AC098614.2	-1.2317	0.002457	0.0068235
RP11-1109M24.5	-1.2314	1.40E-05	5.33E-05
COL4A6	-1.2313	2.49E-08	1.23E-07
DNAJC3-AS1	-1.2313	8.30E-12	5.35E-11
GYLTL1B	-1.2313	5.00E-31	8.30E-30
SLC30A4	-1.2302	4.03E-06	1.62E-05
CELF1	-1.2302	1.99E-136	3.25E-134
RPL22L1	-1.2295	2.40E-107	2.51E-105
TNFRSF11A	-1.2287	0.00012501	0.00042316
SMC6	-1.2273	5.01E-27	7.15E-26
ATP1A1	-1.2273	7.30E-122	9.46E-120
RP11-699L21.1	-1.2262	0.0025311	0.0070079
ABCC11	-1.2256	1.64E-05	6.17E-05
PDE1A	-1.2234	0.00046766	0.0014638
C1orf170	-1.2233	1.61E-05	6.09E-05
FAM45B	-1.2231	0.002655	0.0073214
OST4	-1.2226	2.31E-45	6.29E-44
APOA5	-1.2221	4.22E-05	0.00015153
RUSC1	-1.2218	1.60E-68	7.92E-67

OTOGL	-1.2211	3.11E-05	0.00011311
WDR16	-1.2209	0.00025703	0.00083241
RP1-80N2.3	-1.2202	1.21E-06	5.10E-06
AL353898.3	-1.2195	0.001325	0.0038382
KCTD12	-1.2193	0.0009812	0.002913
DUSP12	-1.2184	7.12E-45	1.91E-43
DALRD3	-1.2172	3.80E-61	1.54E-59
SFTPA2	-1.2171	6.75E-05	0.0002365
RHOV	-1.2155	6.94E-09	3.61E-08
ADAT1	-1.2141	1.16E-60	4.61E-59
SULT2B1	-1.214	0.0012272	0.0035829
POLE4	-1.2126	2.89E-33	5.22E-32
PTPLB	-1.2123	3.59E-108	3.86E-106
FAM174B	-1.2121	8.88E-07	3.80E-06
SLC25A44	-1.2108	4.09E-87	3.01E-85
ZNF849P	-1.2104	0.0029069	0.0079664
SLC25A24	-1.2091	1.41E-28	2.15E-27
FAM66B	-1.208	1.37E-05	5.23E-05
SH2B3	-1.2074	1.60E-09	8.70E-09
MT1X	-1.2066	0.0014385	0.0041443
B4GALNT1	-1.2063	2.28E-14	1.74E-13
C6orf203	-1.2058	9.77E-28	1.44E-26
ELOVL7	-1.204	3.45E-35	6.60E-34
CREB3L1	-1.2028	0.00031242	0.0010013
LRRC16B	-1.2016	0.00039565	0.0012499
C16orf74	-1.2013	7.65E-05	0.00026593
NIPA2	-1.2004	2.36E-69	1.19E-67
TMC6	-1.2002	2.72E-24	3.45E-23
ITPKB	-1.1986	2.33E-50	7.29E-49
AP3S2	-1.1984	1.54E-26	2.16E-25
FBXO48	-1.197	1.30E-06	5.44E-06
RP11-677M14.3	-1.1959	0.00034093	0.0010866
LRRC8B	-1.1959	5.65E-53	1.87E-51
RADIL	-1.195	4.67E-07	2.06E-06
SLCO2B1	-1.195	3.73E-07	1.66E-06
SLC35E1P1	-1.1948	0.00058657	0.0018041
PMVK	-1.1944	1.08E-82	7.18E-81
ST7-AS1	-1.1935	0.0013001	0.0037729
GMPR	-1.1927	3.71E-08	1.82E-07
HLA-DMB	-1.1922	2.17E-14	1.66E-13
PPM1N	-1.1916	1.20E-06	5.05E-06
SWAP70	-1.1908	2.99E-39	6.62E-38
C15orf52	-1.1906	7.19E-06	2.82E-05
LOXL1-AS1	-1.1893	5.94E-18	5.54E-17
AC129929.5	-1.1892	0.0030782	0.008396

AREL1	-1.1891	5.75E-63	2.43E-61
BMP8B	-1.1864	7.54E-14	5.58E-13
LPP-AS2	-1.185	0.00029232	0.00094097
ICAM2	-1.1843	0.001124	0.0032991
CTD-2008P7.6	-1.1834	0.0017242	0.0049062
RCCD1	-1.1828	4.77E-19	4.76E-18
SLC39A14	-1.1826	1.25E-125	1.72E-123
IP6K1	-1.1817	6.93E-89	5.27E-87
TRAPPC2	-1.1816	2.79E-29	4.36E-28
RP11-547I7.2	-1.1812	0.00033363	0.0010652
STPG1	-1.1804	1.73E-18	1.66E-17
RCAN3	-1.1799	4.56E-44	1.19E-42
SOD2	-1.1798	6.43E-137	1.07E-134
OLFM3	-1.1796	4.71E-05	0.00016813
PORCN	-1.1794	2.02E-09	1.09E-08
HPRT1	-1.1794	1.42E-67	6.77E-66
CBLN2	-1.1786	8.44E-90	6.55E-88
AHCY	-1.1784	3.13E-170	8.12E-168
METTL21A	-1.1783	2.91E-21	3.20E-20
ACRBP	-1.1771	1.68E-10	9.78E-10
NR1H4	-1.1761	8.30E-07	3.56E-06
TALDO1	-1.1755	4.89E-115	5.89E-113
GNGT1	-1.1746	0.00074178	0.0022446
CCBP2	-1.1738	0.00073102	0.0022166
C11orf21	-1.1735	0.00096438	0.0028655
RP11-517B11.7	-1.1728	2.84E-13	2.03E-12
CTD-2008P7.5	-1.1721	0.0030565	0.0083445
TPM4	-1.1713	1.47E-121	1.89E-119
GPNMB	-1.1707	5.22E-07	2.29E-06
HSPB8	-1.1699	1.08E-22	1.27E-21
TMC1	-1.1692	0.0036055	0.0097103
HOXB-AS5	-1.1689	4.10E-19	4.11E-18
TXLNA	-1.1667	3.82E-98	3.55E-96
RP4-639F20.1	-1.166	0.0012619	0.0036764
RP5-1085F17.3	-1.1659	7.85E-05	0.00027228
HOXA-AS2	-1.1654	0.00028197	0.00090835
RP13-1032I1.7	-1.1628	1.45E-05	5.49E-05
CREB3L2	-1.1624	2.76E-69	1.38E-67
DDR1	-1.1621	2.97E-84	2.05E-82
PA2G4	-1.162	5.53E-107	5.71E-105
AC009133.12	-1.1619	2.70E-06	1.10E-05
RIMS1	-1.1605	2.10E-10	1.22E-09
FANCF	-1.1601	6.70E-38	1.40E-36
RP11-196G18.3	-1.1591	0.0043912	0.011641
AKAP3	-1.1585	0.0034398	0.009305

CSPG4	-1.1578	0.0030935	0.0084365
MAP3K11	-1.1573	2.78E-54	9.51E-53
DZANK1	-1.1566	6.89E-08	3.29E-07
RAB39A	-1.1565	3.00E-19	3.04E-18
PARVA	-1.1565	4.23E-49	1.28E-47
ANKRD54	-1.155	3.57E-34	6.63E-33
CTD-2555O16.2	-1.1546	0.0020358	0.0057282
RP11-275I14.4	-1.1545	0.0041912	0.01114
RP11-142L1.1	-1.154	0.0022533	0.0062992
RP11-169K16.6	-1.1528	4.33E-06	1.74E-05
CTD-2561J22.2	-1.1525	0.001278	0.0037187
RP11-96K19.4	-1.1521	0.0031302	0.0085289
CBR3-AS1	-1.1519	0.0015222	0.0043698
THNSL1	-1.151	7.63E-58	2.81E-56
TMEM217	-1.1507	0.003629	0.0097686
PCSK7	-1.1499	1.02E-49	3.15E-48
CTD-3064H18.4	-1.1487	0.0005552	0.0017145
SNAP47	-1.1487	6.94E-87	5.08E-85
PPP1R13L	-1.1483	4.06E-32	7.05E-31
TLL1	-1.1476	3.01E-17	2.72E-16
TESK1	-1.1467	4.63E-23	5.54E-22
MUC13	-1.1467	9.76E-82	6.38E-80
BATF2	-1.1454	9.55E-05	0.00032793
KLHL1	-1.1449	0.0029447	0.0080604
CAPSL	-1.1448	0.0004356	0.0013684
RP1-59D14.6	-1.1444	0.000265	0.00085647
RP11-468E2.9	-1.142	0.00058599	0.0018026
PRPF40A	-1.142	3.16E-79	1.98E-77
NAT6	-1.1417	1.78E-16	1.53E-15
CCL28	-1.1411	0.00049875	0.0015536
XPO7	-1.1406	1.90E-96	1.70E-94
CCDC166	-1.1402	0.0050884	0.013312
KIF13B	-1.1402	7.29E-33	1.30E-31
PRLR	-1.139	2.21E-41	5.29E-40
RPRML	-1.1387	0.00018775	0.00062173
VASH1	-1.1387	1.88E-11	1.18E-10
RXFP3	-1.1384	0.0013004	0.0037731
GDF9	-1.1377	0.0030714	0.0083828
EXTL1	-1.1366	0.0017749	0.0050357
ANO1	-1.1348	0.0049134	0.012893
SYN1	-1.1348	9.03E-08	4.26E-07
PLOD1	-1.1342	7.75E-77	4.65E-75
TMEM41A	-1.1338	6.87E-38	1.43E-36
C2orf68	-1.1338	4.29E-49	1.29E-47
TRIM69	-1.1332	5.74E-38	1.21E-36

CA11	-1.1332	7.70E-44	1.98E-42
CFHR5	-1.1329	0.0048069	0.012647
AC018642.1	-1.1329	4.57E-06	1.83E-05
PRNP	-1.1327	5.78E-63	2.43E-61
SLC37A1	-1.1325	7.81E-19	7.70E-18
RP11-407N17.5	-1.1318	0.0010419	0.003081
WDR37	-1.1299	1.70E-41	4.09E-40
KLHDC3	-1.1286	4.79E-106	4.89E-104
LMLN	-1.1283	5.61E-50	1.74E-48
RP1-151F17.1	-1.1273	0.0029455	0.0080604
ZNF208	-1.1268	8.20E-14	6.05E-13
NUPL1	-1.126	4.87E-104	4.81E-102
RP11-690D19.3	-1.1259	8.11E-07	3.49E-06
RAB36	-1.1247	1.12E-23	1.39E-22
CTD-2245F17.2	-1.1239	0.005672	0.01472
WIPI2	-1.1224	6.98E-59	2.63E-57
BLOC1S6	-1.1222	1.85E-67	8.75E-66
MDH1B	-1.1221	0.00021495	0.00070493
KRT19	-1.1221	1.47E-12	1.00E-11
ZNF107	-1.1217	1.88E-46	5.30E-45
AC133680.1	-1.1209	0.0034666	0.0093715
CTC-429P9.3	-1.1201	7.34E-07	3.17E-06
AP000439.3	-1.1187	0.0037452	0.010054
METAP2	-1.1186	3.85E-91	3.06E-89
PAOX	-1.1183	3.81E-08	1.86E-07
TRIM60P18	-1.1181	8.52E-06	3.31E-05
SLA2	-1.1178	0.0060281	0.01556
LRRN1	-1.1161	1.02E-08	5.22E-08
RXRA	-1.1149	5.95E-37	1.21E-35
BTBD2	-1.1147	4.13E-55	1.44E-53
HIF1A	-1.1145	4.96E-63	2.10E-61
LRFN2	-1.1143	0.0061393	0.015816
FOXRED2	-1.114	2.29E-85	1.61E-83
AC007255.8	-1.1137	0.0051569	0.013477
TTC39B	-1.113	1.19E-07	5.56E-07
DHX33	-1.1126	5.13E-74	2.89E-72
DIRAS2	-1.1122	1.48E-16	1.28E-15
TLR5	-1.1117	0.0040804	0.010862
ARNTL2	-1.1116	7.86E-31	1.29E-29
KIAA1704	-1.1115	2.64E-35	5.09E-34
HTR1F	-1.1104	0.0012843	0.0037332
CTD-2521M24.9	-1.1097	0.0033729	0.0091373
MT1L	-1.1085	0.0046961	0.012382
CNTNAP2	-1.1073	1.10E-10	6.53E-10
SPATA31C1	-1.1067	0.0064498	0.016548

BCAR3	-1.105	5.61E-17	4.97E-16
PMF1	-1.1046	4.69E-22	5.36E-21
PRR15L	-1.1046	4.57E-23	5.47E-22
1-Mar	-1.1042	6.31E-58	2.34E-56
BAI2	-1.1038	1.03E-13	7.56E-13
RP11-389K14.3	-1.1037	0.0034138	0.0092372
ZNF888	-1.1036	0.0011075	0.003258
TRIQK	-1.1034	3.70E-41	8.78E-40
PRR16	-1.1025	6.04E-33	1.08E-31
PPP3CA	-1.1023	2.24E-147	4.37E-145
MRPL41	-1.1022	6.13E-46	1.69E-44
RP11-392A22.2	-1.1016	1.11E-09	6.11E-09
RP4-594I10.3	-1.101	9.30E-19	9.13E-18
ENDOG	-1.0998	1.06E-08	5.45E-08
P4HB	-1.0995	6.89E-137	1.13E-134
RLN1	-1.098	0.00073067	0.0022158
ANXA9	-1.0977	3.91E-20	4.11E-19
RP11-299G20.3	-1.0971	0.003766	0.010105
TUBE1	-1.0971	1.66E-23	2.03E-22
TIMM10	-1.0964	3.84E-34	7.10E-33
RP11-753B14.1	-1.0962	0.00062818	0.001921
TTPA	-1.096	6.91E-05	0.0002416
PTTG1	-1.0955	1.46E-60	5.78E-59
BRWD1-IT2	-1.0946	0.0014418	0.0041531
C9orf116	-1.0943	6.71E-07	2.91E-06
ADAP2	-1.0928	2.31E-11	1.44E-10
COL5A1	-1.092	1.59E-19	1.62E-18
ZNF506	-1.0912	2.46E-22	2.83E-21
SMIM12	-1.0906	1.44E-42	3.57E-41
SMPD3	-1.0897	3.08E-13	2.19E-12
RP4-646N3.1	-1.0895	0.0014074	0.0040614
CRB2	-1.0892	0.003474	0.0093892
NT5C3A	-1.089	1.03E-47	2.97E-46
CDH10	-1.0877	1.12E-17	1.04E-16
EPN1	-1.0874	1.87E-81	1.21E-79
C14orf142	-1.0872	1.01E-15	8.38E-15
C7orf63	-1.0864	6.96E-15	5.48E-14
ARHGEF2	-1.0856	6.78E-43	1.70E-41
PITPNA	-1.0849	2.38E-94	2.06E-92
DUS1L	-1.0847	2.89E-86	2.08E-84
RP11-135J2.4	-1.0843	0.0036493	0.0098153
SFT2D2	-1.0841	3.87E-12	2.54E-11
LINC00116	-1.084	3.88E-17	3.48E-16
P2RX6	-1.0836	5.58E-14	4.16E-13
HNF1A-AS1	-1.0828	4.25E-13	3.00E-12

TMEM107	-1.0825	1.17E-27	1.72E-26
PHKA1	-1.0815	6.66E-51	2.13E-49
GNAZ	-1.081	8.36E-21	9.02E-20
NSMAF	-1.0808	3.08E-59	1.18E-57
RP13-379L11.2	-1.0804	0.0054936	0.0143
ZNF493	-1.0799	1.33E-13	9.67E-13
CIZ1	-1.0798	2.85E-57	1.03E-55
ECHDC1	-1.0789	4.70E-24	5.91E-23
CTD-2350C19.1	-1.0775	0.00020788	0.00068379
DNAL1	-1.0772	1.15E-18	1.12E-17
AC005237.4	-1.0767	0.0076233	0.01928
RDH12	-1.0754	0.0072956	0.018505
WDR64	-1.0754	0.0070798	0.018013
B4GALT2	-1.0749	3.39E-91	2.70E-89
TMEM154	-1.0745	3.44E-05	0.00012433
ACOT4	-1.0739	4.62E-06	1.85E-05
CTC-458I2.2	-1.0723	0.0018257	0.0051678
XBP1	-1.072	4.15E-91	3.28E-89
HSF1	-1.0715	6.63E-55	2.31E-53
TTTY15	-1.0712	3.91E-31	6.51E-30
XXbac-B476C20.10	-1.0704	0.0083825	0.021015
DDX31	-1.0698	2.83E-34	5.29E-33
MANF	-1.0688	2.04E-104	2.05E-102
RNF157	-1.0685	5.56E-23	6.60E-22
MAGEA4	-1.0684	9.54E-131	1.43E-128
TRGC2	-1.0672	0.0086679	0.02166
PFKFB3	-1.0663	2.10E-48	6.20E-47
AC005077.14	-1.0662	0.00025202	0.00081771
PLCH1	-1.0656	1.72E-29	2.71E-28
RP13-36G14.4	-1.0655	0.00038124	0.0012072
GLRX	-1.0655	1.06E-07	4.95E-07
ADRA1A	-1.0653	2.76E-08	1.36E-07
RFFL	-1.0653	1.69E-13	1.22E-12
KCNG3	-1.0649	0.0058181	0.015072
TM4SF1	-1.0644	0.0053926	0.014047
RNH1	-1.0644	6.68E-72	3.59E-70
RC3H2	-1.0638	3.17E-65	1.40E-63
LRRC4C	-1.0634	3.39E-05	0.00012277
CYCS	-1.0633	8.73E-85	6.10E-83
ZNF22	-1.0617	2.93E-15	2.36E-14
WDR89	-1.0615	1.34E-18	1.30E-17
TMEM79	-1.0611	7.04E-25	9.20E-24
TOX	-1.0607	8.04E-08	3.82E-07
PEAK1	-1.0602	0.00086885	0.0026018
SIVA1	-1.0601	1.32E-41	3.18E-40

BANK1	-1.0599	0.00019184	0.00063446
RGS4	-1.0592	0.001183	0.0034635
TUBA4A	-1.0591	9.46E-39	2.04E-37
NCEH1	-1.0587	2.39E-27	3.47E-26
EMILIN3	-1.0582	0.0071627	0.018203
KIF21A	-1.058	8.14E-68	3.95E-66
THRA	-1.0573	1.36E-26	1.91E-25
UBAC1	-1.0553	7.24E-60	2.82E-58
RNPEP	-1.0552	2.14E-119	2.69E-117
LSM5	-1.055	4.92E-59	1.87E-57
UGDH-AS1	-1.0544	0.00085117	0.0025529
TBC1D10B	-1.0541	4.01E-60	1.57E-58
CTB-178M22.2	-1.0539	0.0063497	0.016309
VPS37C	-1.0531	2.00E-44	5.30E-43
AP001816.1	-1.0525	5.74E-27	8.18E-26
PFDN1	-1.0518	2.45E-27	3.56E-26
IL1R2	-1.0516	2.67E-07	1.21E-06
ZNF98	-1.0514	6.69E-12	4.33E-11
AC145343.2	-1.0507	0.0045697	0.012072
ADCY3	-1.0486	2.76E-47	7.92E-46
NOL10	-1.0484	1.31E-38	2.81E-37
IRAK1	-1.0484	3.73E-96	3.34E-94
DBP	-1.047	6.53E-10	3.67E-09
HIST2H2BE	-1.0469	3.20E-34	5.97E-33
SLC35A4	-1.0465	5.65E-86	4.04E-84
EPN3	-1.0447	1.25E-28	1.91E-27
ITGA2B	-1.0444	0.010118	0.024952
MBP	-1.0441	4.46E-19	4.45E-18
PLA1A	-1.0441	1.90E-19	1.93E-18
BZRAP1	-1.0439	2.64E-34	4.95E-33
HSBP1	-1.0431	1.46E-88	1.10E-86
BCL2L13	-1.0427	2.34E-79	1.48E-77
RP4-562J12.2	-1.0421	0.0070444	0.01794
C4orf47	-1.0418	0.010117	0.024952
ABCA2	-1.0417	2.16E-53	7.25E-52
CAAP1	-1.0408	2.31E-40	5.34E-39
AGGF1P3	-1.0406	0.0099182	0.024507
NKPD1	-1.0406	2.16E-07	9.83E-07
STRN4	-1.0402	7.89E-63	3.30E-61
GRM3	-1.0394	2.25E-05	8.32E-05
RP11-406A9.2	-1.0393	0.0028789	0.0079001
CYP4F23P	-1.0388	0.0084596	0.02118
WFS1	-1.0388	8.55E-31	1.41E-29
IDNK	-1.0385	4.05E-09	2.15E-08
CAPNS1	-1.0377	7.80E-108	8.21E-106

RP11-74E24.2	-1.0374	0.0083827	0.021015
ALDOC	-1.0363	1.42E-36	2.86E-35
WDR61	-1.0357	2.52E-49	7.66E-48
UCHL1	-1.0347	0.0089627	0.02234
MAT1A	-1.0347	5.75E-06	2.28E-05
KPNB1	-1.034	3.91E-118	4.83E-116
DUS3L	-1.0337	2.24E-35	4.36E-34
DPF2	-1.0335	3.58E-43	9.03E-42
BRDT	-1.033	0.011143	0.027249
RP11-440L14.1	-1.0328	1.74E-06	7.21E-06
HTR1D	-1.0327	0.0063563	0.016324
ZNF490	-1.0322	8.85E-06	3.43E-05
GLT8D1	-1.0319	6.75E-36	1.33E-34
ABCA13	-1.0317	0.0081581	0.020522
PPP1R16A	-1.0313	2.90E-34	5.42E-33
MYRF	-1.0307	4.29E-35	8.19E-34
IREB2	-1.0296	8.24E-66	3.74E-64
CDON	-1.0294	2.38E-10	1.38E-09
ULBP2	-1.029	0.0058163	0.01507
CCBL2	-1.0283	4.14E-17	3.71E-16
OGDH	-1.0282	5.62E-92	4.61E-90
PIM2	-1.0276	7.23E-27	1.03E-25
PHGR1	-1.0273	0.010719	0.026309
NAA40	-1.0272	2.97E-44	7.82E-43
PRKCI	-1.026	1.39E-62	5.75E-61
SUSD2	-1.0255	4.00E-08	1.95E-07
ADPRHL1	-1.0252	1.73E-23	2.12E-22
DGCR2	-1.0242	2.60E-57	9.45E-56
NAT14	-1.0239	4.55E-16	3.84E-15
FAM66A	-1.0238	0.011765	0.028626
C1QL4	-1.0237	1.98E-15	1.61E-14
SLC38A7	-1.0233	1.14E-28	1.75E-27
RP11-110I1.12	-1.023	2.33E-10	1.35E-09
WDR76	-1.0229	2.76E-26	3.85E-25
TTC7A	-1.0225	4.12E-10	2.35E-09
ABCC1	-1.0224	4.54E-41	1.07E-39
CTC-203F4.1	-1.0221	0.0085482	0.021381
FBXW11	-1.0217	1.74E-70	8.91E-69
AC026202.3	-1.0215	0.009254	0.022997
LMOD1	-1.0211	6.43E-05	0.00022597
UFD1L	-1.0207	1.54E-52	5.05E-51
MPV17	-1.0205	1.35E-30	2.20E-29
RYR1	-1.0204	1.15E-15	9.48E-15
FAM5C	-1.0199	0.010207	0.025151
SLAMF9	-1.0198	0.0072154	0.018319

RP11-464F9.9	-1.0193	0.007334	0.018593
AC097724.3	-1.0192	0.0023995	0.0066809
RABL5	-1.0192	2.84E-20	3.00E-19
CCDC134	-1.019	2.90E-10	1.67E-09
R3HDM4	-1.0188	1.45E-46	4.11E-45
TMEM150A	-1.0181	1.24E-14	9.65E-14
ZNF43	-1.0179	4.22E-48	1.23E-46
RGS19	-1.0168	0.00057806	0.0017804
SCARB1	-1.0163	6.08E-102	5.86E-100
ZNF541	-1.0162	0.0017576	0.0049887
PLEKHB2	-1.0161	2.29E-88	1.72E-86
ZNF845	-1.0147	1.25E-13	9.10E-13
RP11-318M2.2	-1.0142	0.008366	0.020989
VIM-AS1	-1.0132	3.16E-05	0.00011509
RP11-262H14.7	-1.0129	0.0062744	0.016134
MAGIX	-1.0128	0.0026177	0.0072291
MYH15	-1.0118	4.66E-10	2.65E-09
UNC5A	-1.0114	0.0096554	0.02392
GJB1	-1.011	4.56E-17	4.07E-16
SLC6A4	-1.0101	0.0083632	0.020984
C9orf91	-1.0098	8.27E-28	1.22E-26
NPR1	-1.0092	5.74E-06	2.28E-05
MAP2K3	-1.0091	9.51E-71	4.93E-69
ZBED3-AS1	-1.0081	5.25E-05	0.00018642
IRAK2	-1.0077	1.07E-12	7.36E-12
PPP2R3A	-1.0074	7.26E-16	6.07E-15
LONP1	-1.0052	7.71E-74	4.30E-72
RP11-549B18.1	-1.0049	0.00088363	0.0026422
RP11-195E2.4	-1.0049	1.80E-08	9.05E-08
PDGFRB	-1.0048	0.0032914	0.0089364
ZBTB5	-1.0045	3.17E-39	7.02E-38
RIPK2	-1.0044	2.00E-32	3.50E-31
ATP8B3	-1.0038	0.013582	0.03255
TCEA1	-1.0037	3.02E-88	2.26E-86
E2F8	-1.0029	1.60E-16	1.38E-15
TRIM72	-1.0028	0.0099508	0.024584
H6PD	-1.0027	6.60E-57	2.38E-55
AC015849.16	-1.002	9.84E-06	3.80E-05
RPL3L	-1.0018	0.010854	0.026615
JAK3	-1.0018	3.24E-05	0.00011756
EMC10	-1.0016	2.48E-53	8.31E-52
AC108488.3	-1.001	7.06E-07	3.06E-06
SCCPDH	-1.0007	4.13E-68	2.01E-66
PRODH	-1.0004	1.06E-05	4.09E-05
ZNF534	-1.0003	0.013221	0.031788

HIST1H2AG	-0.99993	3.92E-06	1.58E-05
TTLL7	-0.99986	4.39E-16	3.71E-15
FTLP12	-0.99911	0.0081753	0.020563
SERPINA10	-0.99882	0.013249	0.031852
CWH43	-0.99711	0.012146	0.029467
ASCL2	-0.99693	0.013755	0.032927
CFC1	-0.99687	7.97E-25	1.04E-23
KRAS	-0.9961	1.46E-61	5.96E-60
UGT3A1	-0.99576	0.00026699	0.00086223
PDE2A	-0.99517	0.0067215	0.017183
FER1L5	-0.99509	0.011224	0.027416
CTD-3074O7.12	-0.99502	6.52E-13	4.55E-12
APLP1	-0.99458	6.29E-31	1.04E-29
CPSF4	-0.99436	4.04E-46	1.12E-44
MAPK10	-0.99377	0.013286	0.03193
FTSJ3	-0.99358	1.23E-64	5.41E-63
C22orf46	-0.99284	3.67E-18	3.46E-17
RAMP2	-0.99239	0.011787	0.028673
TLCD2	-0.99227	8.56E-05	0.0002957
METTL12	-0.99178	4.32E-13	3.05E-12
MAPK12	-0.99123	4.05E-51	1.30E-49
BCAS1	-0.99059	0.01454	0.034571
PNMT	-0.98991	0.01241	0.030047
KHK	-0.98964	1.59E-07	7.32E-07
TBCE	-0.98904	4.03E-45	1.09E-43
ANO5	-0.98886	0.00018706	0.00061966
AL672294.1	-0.98777	0.012126	0.029423
C2orf27A	-0.98755	3.70E-10	2.11E-09
RMDN3	-0.98722	3.18E-46	8.86E-45
ERI2	-0.98706	8.13E-36	1.60E-34
PRKD3	-0.98681	6.91E-23	8.17E-22
PITPNB	-0.98636	1.10E-62	4.57E-61
LCMT1	-0.9862	3.56E-23	4.30E-22
RP11-157G21.2	-0.98543	0.010138	0.024994
GCNT1	-0.98511	2.39E-42	5.87E-41
SLC5A12	-0.98439	0.0078245	0.019754
RIMKLA	-0.98411	7.54E-05	0.00026244
PIK3R5	-0.98395	0.00014018	0.00047127
ISCA1P4	-0.98308	0.015454	0.036549
PADI2	-0.9825	0.014549	0.034589
RP11-796E2.4	-0.98217	0.0078647	0.019846
FAM172A	-0.98187	1.00E-25	1.36E-24
CTD-2647L4.3	-0.98151	0.00056165	0.0017327
CTC-429P9.5	-0.98144	5.74E-05	0.00020308
RAC3	-0.98124	3.85E-15	3.09E-14

ZNHIT6	-0.98054	3.10E-39	6.87E-38
RP11-483C6.1	-0.98041	0.015947	0.037585
POU2AF1	-0.97963	0.0031231	0.0085116
DIAPH1	-0.9787	1.17E-85	8.30E-84
RP11-564C4.6	-0.97848	6.53E-10	3.67E-09
DCHS1	-0.97809	1.02E-05	3.92E-05
MRO	-0.97778	1.55E-09	8.46E-09
RP11-94B19.7	-0.97746	0.0071893	0.01826
GALM	-0.97737	1.58E-28	2.40E-27
SLC38A6	-0.97724	4.54E-05	0.00016248
SCD	-0.97722	3.83E-68	1.87E-66
AFAP1L1	-0.97696	9.68E-07	4.12E-06
THEM5	-0.97673	0.0039589	0.010565
RP11-336A10.5	-0.97391	0.01338	0.032133
CYP2C19	-0.97329	0.0067159	0.017173
RPAP1	-0.97326	2.50E-44	6.61E-43
SLC41A1	-0.97299	1.18E-42	2.95E-41
APOBEC3F	-0.97263	1.17E-13	8.56E-13
ZNF100	-0.97251	6.98E-20	7.24E-19
PXN	-0.97205	5.11E-45	1.37E-43
RP11-342K6.4	-0.97142	0.015379	0.036391
IFIT2	-0.97124	0.0011826	0.0034629
TMEM81	-0.97118	2.10E-07	9.56E-07
ALOXE3	-0.97041	4.33E-12	2.83E-11
BCR	-0.96976	5.65E-39	1.24E-37
LOX	-0.96958	1.76E-06	7.31E-06
TCERG1L	-0.96948	3.11E-05	0.00011336
ZBED6	-0.9694	2.76E-13	1.97E-12
SIGLEC10	-0.96927	0.0097597	0.024146
AC005307.3	-0.96909	0.0080339	0.020239
RP11-1033A18.1	-0.9688	0.012841	0.030965
DHCR24	-0.96846	3.16E-97	2.90E-95
HSD3B7	-0.96827	1.02E-35	2.01E-34
RASGRP2	-0.96806	0.0025544	0.0070675
EMC8	-0.96803	2.17E-34	4.09E-33
RP11-22C11.2	-0.96698	5.44E-07	2.38E-06
IQCG	-0.96683	9.80E-15	7.66E-14
UAP1L1	-0.9668	8.60E-24	1.07E-22
HSPB1	-0.96675	3.71E-46	1.03E-44
NLRP9	-0.96641	0.014117	0.033679
SGK2	-0.96604	4.94E-32	8.55E-31
SOGA2	-0.96578	4.61E-06	1.84E-05
MXRA8	-0.96573	1.33E-25	1.81E-24
TRIM15	-0.96562	0.0051471	0.013453
INSC	-0.96544	0.012848	0.030975

FZD1	-0.96499	3.20E-35	6.13E-34
RP5-983L19.2	-0.9648	0.016471	0.038653
EXOC8	-0.96477	1.42E-34	2.69E-33
NQO1	-0.96475	7.80E-22	8.80E-21
YARS	-0.96436	1.62E-60	6.42E-59
VN1R81P	-0.96435	0.0049282	0.012925
B3GNT3	-0.96434	2.93E-08	1.44E-07
ATRAID	-0.96391	3.67E-47	1.05E-45
TNK2	-0.96193	4.49E-27	6.45E-26
LINC00595	-0.96173	0.017065	0.039922
RP11-483I13.5	-0.96141	0.018153	0.042219
CDAN1	-0.96141	5.03E-25	6.60E-24
CTD-2311B13.7	-0.96096	0.0035817	0.0096538
FAM109B	-0.96094	1.53E-09	8.31E-09
TFCP2	-0.96077	0.017635	0.041096
SLC2A1-AS1	-0.96051	0.0045629	0.012057
NUP93	-0.95998	1.79E-96	1.61E-94
MGAT4A	-0.95852	3.92E-11	2.40E-10
NFKB1	-0.95818	4.22E-41	9.98E-40
PDCD11	-0.95723	2.98E-58	1.12E-56
FBLN7	-0.9572	9.42E-05	0.00032372
CTC-559E9.5	-0.95714	0.0064494	0.016548
CTC-205M6.5	-0.95706	0.00098981	0.0029373
MAP2	-0.957	1.42E-63	6.05E-62
PLGRKT	-0.95697	8.67E-28	1.28E-26
AIM1L	-0.95665	0.0018803	0.0053167
MZB1	-0.95662	0.0037072	0.0099572
ZMYND10	-0.95637	0.0039432	0.010527
C18orf54	-0.9563	2.54E-18	2.42E-17
ZFAND1	-0.95613	2.08E-46	5.84E-45
RP11-111M22.2	-0.95548	0.00021548	0.00070645
MRPS27	-0.95538	1.18E-65	5.30E-64
PDZD3	-0.95536	5.39E-06	2.14E-05
ZNF676	-0.95529	3.99E-14	3.01E-13
C1orf51	-0.95502	5.12E-84	3.51E-82
AC004837.5	-0.95462	0.018231	0.042377
FRMD4B	-0.95461	2.01E-09	1.09E-08
LL22NC03-86G7.1	-0.95454	0.00015824	0.00052906
PLA2G3	-0.95448	8.14E-17	7.16E-16
FAM213B	-0.95412	2.95E-13	2.10E-12
KIF21B	-0.95331	6.13E-06	2.42E-05
SPATA4	-0.95313	0.019193	0.044399
ZNF778	-0.95296	1.62E-13	1.17E-12
C21orf119	-0.95262	8.37E-06	3.26E-05
CRIP1	-0.95257	0.017514	0.040846

TMEM128	-0.95225	3.12E-34	5.81E-33
INF2	-0.95183	4.69E-26	6.47E-25
ZNF320	-0.95143	7.06E-39	1.54E-37
FRMPD1	-0.9514	0.00047599	0.0014879
ZBTB9	-0.95043	5.12E-20	5.35E-19
GIGYF2	-0.94949	3.76E-67	1.76E-65
CCDC74B-AS1	-0.94787	0.002355	0.0065649
AF238380.5	-0.94757	0.0021947	0.0061469
C12orf68	-0.94722	0.018591	0.043137
PLA2G1B	-0.94713	0.014745	0.035023
TAPT1	-0.94686	1.28E-27	1.88E-26
CHST13	-0.94652	0.0001917	0.0006341
HILS1	-0.94647	0.018501	0.04296
RP11-807H7.2	-0.94617	0.008766	0.021886
DHRS4	-0.94569	6.77E-25	8.84E-24
RP11-85M11.2	-0.94482	0.016541	0.038808
LRRC4B	-0.94473	0.0083975	0.021048
CD38	-0.94424	0.01445	0.034402
MSR1	-0.94398	0.00044457	0.0013945
RP11-638I2.2	-0.94368	0.01606	0.0378
FAM63B	-0.9436	4.49E-42	1.09E-40
AC116407.2	-0.94345	0.020049	0.046172
TEX35	-0.9432	0.020259	0.046579
SHISA5	-0.94316	5.84E-70	2.97E-68
AKR1C1	-0.94249	1.16E-36	2.34E-35
RLN2	-0.94209	4.60E-06	1.84E-05
AC093388.3	-0.94194	0.019744	0.04552
C5orf30	-0.94171	4.79E-27	6.86E-26
RP11-61A14.2	-0.94164	2.16E-07	9.84E-07
GALNT6	-0.93994	0.00090744	0.0027087
TSPAN7	-0.9398	0.0043034	0.011424
RFT1	-0.93973	4.94E-47	1.41E-45
PLD2	-0.93963	3.33E-13	2.37E-12
OR6E1P	-0.93915	0.012002	0.029148
PTGES	-0.9385	1.95E-07	8.92E-07
FRRS1	-0.93767	1.33E-18	1.29E-17
TMC8	-0.9369	0.014189	0.033826
TMSB15A	-0.9366	0.00047112	0.0014742
RP11-846F4.11	-0.93657	0.019285	0.044566
TRIM36	-0.93654	5.26E-73	2.89E-71
RP11-532F12.5	-0.93598	0.0085726	0.02144
ECM2	-0.93524	0.011422	0.027854
GBA3	-0.93508	0.020736	0.04756
ABCC9	-0.93493	0.0011089	0.0032613
ENAM	-0.93443	0.021666	0.049468

GLB1	-0.93437	9.48E-56	3.36E-54
TEX15	-0.93422	9.84E-12	6.31E-11
PLEKHJ1	-0.9342	3.35E-34	6.23E-33
QSOX1	-0.93323	6.60E-66	3.00E-64
HRSP12	-0.93288	1.24E-18	1.20E-17
WNT2	-0.93238	0.0021901	0.0061348
CCRN4L	-0.93221	3.55E-12	2.34E-11
B4GALT3	-0.93193	1.74E-62	7.20E-61
CYP2T2P	-0.93176	0.020811	0.047716
AC069282.6	-0.93158	5.19E-06	2.07E-05
ACPP	-0.9315	3.25E-19	3.27E-18
RP11-617F23.1	-0.93117	0.0039164	0.010466
SNHG7	-0.93034	3.54E-36	7.05E-35
MCAT	-0.93006	8.88E-31	1.46E-29
CERS6	-0.92929	1.23E-36	2.49E-35
ZNF99	-0.92881	0.0052624	0.013735
ETV5	-0.92853	7.36E-32	1.26E-30
RBM23	-0.92837	2.05E-53	6.88E-52
AC046143.7	-0.92835	0.0044557	0.011799
UPK2	-0.92799	0.013947	0.033322
MMEL1	-0.92789	0.021811	0.049755
C14orf182	-0.92788	0.0047932	0.012616
RELL1	-0.92752	5.98E-07	2.60E-06
USP2	-0.92718	2.46E-05	9.07E-05
IPO13	-0.92615	2.27E-38	4.85E-37
DHRS12	-0.92608	7.21E-10	4.04E-09
SMC1B	-0.92562	1.07E-14	8.33E-14
PKN3	-0.92551	4.07E-23	4.89E-22
HYAL2	-0.92508	7.25E-40	1.65E-38
LINC00339	-0.92361	7.73E-13	5.36E-12
HSP90AA1	-0.92359	4.83E-66	2.21E-64
ODAM	-0.92352	0.0011726	0.0034355
LRRC17	-0.92284	0.019635	0.045285
RP11-159F24.3	-0.92278	0.017286	0.040356
CHPF	-0.92257	1.63E-32	2.86E-31
FCGR1A	-0.92231	0.013355	0.032081
C10orf11	-0.92224	0.00010319	0.00035314
TSKU	-0.92215	3.65E-26	5.07E-25
ASAH2C	-0.92162	0.021175	0.048443
ENOX2	-0.92159	1.40E-23	1.73E-22
CKAP5	-0.92152	4.11E-75	2.39E-73
ZNF730	-0.92062	5.12E-06	2.04E-05
OR51B5	-0.92048	0.017097	0.039987
COQ9	-0.91965	6.34E-52	2.05E-50
MYBPC1	-0.91944	9.23E-14	6.79E-13

ITPKC	-0.9194	1.42E-28	2.16E-27
IL5	-0.91887	0.016797	0.039352
CTD-2514K5.2	-0.91878	0.016646	0.039026
ETNK1	-0.91872	5.10E-59	1.93E-57
TOP1	-0.91841	2.56E-53	8.56E-52
ERAL1	-0.91798	1.39E-50	4.41E-49
AC091167.3	-0.91732	0.010852	0.026614
MGAT4B	-0.9165	1.81E-54	6.25E-53
RP11-509J21.1	-0.91418	0.012122	0.029416
IDH2	-0.91349	5.31E-44	1.38E-42
PTPRB	-0.91331	1.25E-54	4.34E-53
A2M-AS1	-0.91298	1.57E-06	6.53E-06
RP11-166P13.3	-0.9118	0.0011185	0.0032854
TRIM73	-0.91172	0.015938	0.037569
SLC31A1	-0.91136	1.00E-46	2.85E-45
C9orf152	-0.91078	0.00030538	0.00098013
SDF2L1	-0.91067	3.38E-21	3.71E-20
AC109309.4	-0.91004	0.010936	0.026794
TRMT6	-0.9097	2.87E-35	5.52E-34
WDR46	-0.90958	6.00E-46	1.66E-44
MUC3A	-0.90927	0.02114	0.04837
AGAP11	-0.90836	0.00070026	0.0021292
FAM188B	-0.90832	0.012559	0.030374
RP1-90J20.7	-0.90796	0.0016945	0.0048294
GSAP	-0.90788	4.95E-23	5.90E-22
FEZF1-AS1	-0.90702	0.00024385	0.00079211
HYDIN	-0.90682	0.00011596	0.00039381
ST6GALNAC5	-0.90624	5.23E-07	2.29E-06
RP11-81N13.1	-0.90475	0.00032061	0.0010261
EIF3M	-0.90473	4.34E-44	1.14E-42
LINC00341	-0.90377	0.0060605	0.015634
AC012456.4	-0.90267	0.021752	0.049648
SLC36A1	-0.90263	2.56E-24	3.25E-23
HIST2H2BF	-0.9026	0.0071106	0.018082
ELMO3	-0.90217	1.14E-39	2.57E-38
MARVELD1	-0.90162	1.23E-49	3.78E-48
RP11-262H14.4	-0.90099	0.0031361	0.0085425
TRIM31	-0.90043	0.020183	0.046429
ZNF572	-0.9002	0.0029718	0.0081255
TMEM136	-0.90018	3.21E-08	1.58E-07
PRMT1	-0.90007	5.47E-75	3.17E-73
POLD1	-0.8998	1.52E-36	3.04E-35
RP11-429J17.7	-0.89914	1.71E-05	6.43E-05
RNASEH2A	-0.89913	3.13E-38	6.64E-37
PIR	-0.89912	3.01E-24	3.81E-23

POLR3GL	-0.89887	4.88E-23	5.83E-22
LYPD5	-0.89811	0.015501	0.036639
RWDD2A	-0.89807	7.67E-11	4.60E-10
ALAD	-0.89783	1.79E-31	3.03E-30
LIG1	-0.8973	1.38E-43	3.51E-42
MROH6	-0.89723	1.28E-30	2.09E-29
RNF11	-0.89661	5.55E-44	1.44E-42
NDRG2	-0.89515	1.42E-18	1.37E-17
CTD-2196E14.9	-0.89455	5.40E-10	3.05E-09
RFC1	-0.89432	2.30E-45	6.26E-44
LA16c-83F12.6	-0.89409	0.0052304	0.013657
SLC35G2	-0.89308	0.0011003	0.0032388
IFIH1	-0.89244	3.91E-44	1.03E-42
RAPSN	-0.89227	0.015012	0.035607
CDK6	-0.892	2.06E-28	3.13E-27
ZMYM3	-0.89198	1.20E-45	3.29E-44
EHF	-0.89097	6.27E-40	1.43E-38
TTC18	-0.89095	2.90E-05	0.00010578
HAPLN2	-0.89064	0.0046114	0.012171
RPUSD3	-0.89058	4.21E-30	6.79E-29
TTI2	-0.8903	5.53E-25	7.25E-24
PRDM11	-0.89017	0.00033648	0.0010735
BCL2L14	-0.88936	0.002586	0.0071484
AC132872.1	-0.88906	0.02178	0.049695
VSIG10L	-0.88875	6.32E-13	4.41E-12
ZNF525	-0.88845	5.57E-15	4.41E-14
FAM89B	-0.88831	0.018932	0.043825
TECPR1	-0.88663	2.29E-26	3.20E-25
KLB	-0.8857	1.41E-10	8.29E-10
RP11-760H22.2	-0.88566	8.01E-05	0.00027762
HIST1H2AK	-0.88518	0.01508	0.035757
SLC10A5	-0.88484	8.49E-09	4.38E-08
ASAP2	-0.88435	6.45E-22	7.31E-21
ARHGEF26-AS1	-0.88434	7.20E-08	3.43E-07
MAF1	-0.88431	1.03E-52	3.39E-51
LARP1	-0.88381	6.77E-99	6.32E-97
TMEM41B	-0.88292	1.08E-36	2.18E-35
EBP	-0.88212	7.05E-58	2.61E-56
TUG1	-0.88209	1.24E-50	3.94E-49
RP5-894A10.5	-0.88194	5.41E-06	2.15E-05
ZNF117	-0.88176	5.06E-11	3.07E-10
MFSD4	-0.88144	7.86E-26	1.08E-24
ABCC6P1	-0.88005	9.66E-17	8.44E-16
RNF167	-0.8794	1.97E-64	8.55E-63
CEP250	-0.87855	2.04E-27	2.98E-26

CYB5R4	-0.87812	1.68E-11	1.06E-10
POLB	-0.87808	2.38E-23	2.90E-22
RP11-61A14.3	-0.87767	4.07E-11	2.49E-10
ZNF75D	-0.87766	2.89E-14	2.20E-13
DGKB	-0.87691	0.011312	0.027608
P2RY2	-0.87648	0.017553	0.040923
ZC3H7B	-0.87645	1.57E-41	3.77E-40
ARHGAP4	-0.87629	5.16E-05	0.00018346
SNX22	-0.87619	5.84E-08	2.81E-07
ANO7	-0.87607	2.46E-05	9.06E-05
SMYD5	-0.87597	7.39E-30	1.18E-28
TMEM185B	-0.87588	5.30E-16	4.46E-15
FXYD4	-0.87585	0.012271	0.02975
E2F4	-0.87513	2.85E-39	6.33E-38
LGALS4	-0.87485	5.91E-07	2.58E-06
TXNDC12	-0.87477	6.26E-42	1.52E-40
FAT2	-0.8744	0.019465	0.044942
CAMTA2	-0.8744	2.17E-32	3.80E-31
PPP2R4	-0.87423	1.43E-96	1.30E-94
IPMK	-0.87405	1.03E-14	8.04E-14
AC097662.2	-0.87386	0.010183	0.025096
TOB2	-0.87358	8.28E-50	2.55E-48
PRAME	-0.87325	6.79E-28	1.01E-26
UCP1	-0.87323	0.0031808	0.0086518
LAS1L	-0.87219	1.21E-41	2.93E-40
REEP1	-0.87209	0.0011975	0.0035021
ZNF674-AS1	-0.87209	6.32E-05	0.00022252
CTB-55O6.8	-0.87183	2.85E-07	1.28E-06
OGFOD1	-0.87157	1.07E-30	1.76E-29
POLD3	-0.87156	3.56E-18	3.36E-17
TTC16	-0.87113	0.017045	0.039887
PCK2	-0.87088	2.88E-37	5.90E-36
DUSP11	-0.8706	2.87E-18	2.73E-17
PPP6R2	-0.87047	5.72E-42	1.39E-40
USE1	-0.87038	5.64E-20	5.88E-19
SENP1	-0.87037	4.98E-33	8.94E-32
ZNF253	-0.86989	8.82E-17	7.73E-16
PTP4A1	-0.86989	4.27E-49	1.29E-47
KLRG1	-0.8698	0.0039048	0.010439
FBXW8	-0.86971	8.11E-25	1.06E-23
RP11-872J21.3	-0.86902	0.0034852	0.0094169
CANT1	-0.86881	2.07E-41	4.97E-40
CTD-3093M3.1	-0.86875	0.015822	0.037324
INTS4	-0.86836	2.55E-24	3.24E-23
ELP2	-0.86762	6.51E-39	1.42E-37

TUBB8P2	-0.86748	0.01399	0.033403
RBM47	-0.86719	1.96E-59	7.53E-58
TRIM28	-0.86712	1.09E-59	4.23E-58
CTC-429P9.2	-0.8669	0.0025104	0.0069579
MTMR6	-0.86665	2.46E-36	4.90E-35
C9orf173	-0.86625	3.18E-08	1.56E-07
PRR3	-0.8656	5.92E-19	5.86E-18
METTL21B	-0.86526	1.91E-11	1.19E-10
PGM2L1	-0.86461	0.00031461	0.0010078
ROM1	-0.8646	0.012893	0.031069
EPB41L4A-AS1	-0.86448	1.76E-18	1.70E-17
PSPH	-0.86447	6.04E-41	1.42E-39
MED6	-0.86402	9.52E-12	6.11E-11
NINL	-0.86265	2.44E-18	2.34E-17
MIR3180-1	-0.86238	0.018695	0.043338
ADCY5	-0.86226	0.01514	0.035886
SLC2A1	-0.86197	3.45E-26	4.80E-25
MEGF8	-0.86161	3.13E-30	5.07E-29
CARD14	-0.86152	2.17E-10	1.26E-09
RNPEPL1	-0.86139	3.62E-31	6.02E-30
RP4-545C24.1	-0.86126	0.012687	0.030647
DLD	-0.8607	1.26E-65	5.67E-64
ENTPD5	-0.8605	3.03E-22	3.49E-21
HCG15	-0.86013	0.0044927	0.011885
LCE1E	-0.86004	0.001699	0.004841
GAS6	-0.85989	8.05E-16	6.71E-15
RP11-187C18.4	-0.85946	0.00036667	0.0011631
BACE2	-0.85883	8.02E-16	6.69E-15
DDC	-0.85834	1.76E-29	2.77E-28
CTD-2410N18.3	-0.85812	0.00073738	0.0022331
RP11-96H19.1	-0.8581	0.0002288	0.00074668
FES	-0.85808	7.78E-07	3.35E-06
AMPD2	-0.85798	2.44E-23	2.97E-22
INSIG1	-0.85733	8.25E-51	2.64E-49
CD6	-0.85714	2.14E-08	1.07E-07
EFNA2	-0.85685	1.30E-10	7.65E-10
DNPEP	-0.85676	1.36E-21	1.52E-20
TCEA2	-0.85628	1.29E-19	1.32E-18
RGP1	-0.85619	3.46E-44	9.10E-43
HDAC9	-0.85575	0.0025253	0.0069954
SH2D2A	-0.85573	0.0026689	0.0073521
ALOX12B	-0.85565	0.0052379	0.013675
RP11-23E19.2	-0.85555	0.020085	0.046229
ZNF101	-0.85539	5.74E-14	4.28E-13
RP11-241F15.10	-0.85447	0.020577	0.047225

TONSL	-0.8544	2.89E-32	5.05E-31
AKR1C3	-0.85271	2.77E-15	2.24E-14
LINC00263	-0.85247	5.56E-10	3.14E-09
CTC-503J8.6	-0.85241	1.70E-33	3.09E-32
CCT8	-0.85235	1.64E-48	4.86E-47
RAB27B	-0.85206	3.74E-10	2.13E-09
AC011298.2	-0.85189	0.015265	0.03615
NUDCD1	-0.85188	2.38E-24	3.03E-23
RP11-660L16.2	-0.85152	5.99E-05	0.00021161
LRWD1	-0.8514	7.40E-26	1.02E-24
HAUS4	-0.85133	8.13E-11	4.86E-10
LDHD	-0.85109	6.99E-15	5.50E-14
CASP10	-0.85068	0.010172	0.025071
GLIS3	-0.85064	1.08E-06	4.57E-06
PLEKHA2	-0.84884	7.69E-12	4.97E-11
TNFRSF13C	-0.84869	0.013805	0.033032
PROB1	-0.84866	1.09E-08	5.58E-08
CYB5R2	-0.84863	4.07E-06	1.64E-05
RBBP8NL	-0.84796	1.18E-10	6.94E-10
RBM15B	-0.8477	8.07E-58	2.97E-56
CYP2C8	-0.8472	2.32E-07	1.05E-06
MGAT1	-0.84669	2.26E-45	6.16E-44
KLK15	-0.84655	0.0029004	0.0079506
TMPO-AS1	-0.84643	0.00055841	0.0017237
ATG10	-0.84594	1.56E-21	1.73E-20
RCBTB1	-0.84461	2.51E-31	4.22E-30
CCDC85A	-0.84415	0.0068915	0.017579
GPS1	-0.84389	1.06E-35	2.07E-34
HAUS2	-0.84383	9.06E-22	1.02E-20
DHRS4L2	-0.84338	4.57E-15	3.64E-14
RP11-600F24.7	-0.84319	6.81E-07	2.95E-06
ALDH1L2	-0.84308	8.30E-61	3.32E-59
C1orf226	-0.84268	1.20E-24	1.54E-23
TIMM8AP1	-0.84245	0.0031588	0.0085977
BCL2L2	-0.84244	5.07E-28	7.55E-27
DHX15	-0.84242	4.18E-82	2.76E-80
GPER	-0.8419	0.0060059	0.015505
PHF2	-0.84169	5.33E-32	9.24E-31
RP11-96K19.2	-0.84118	0.017777	0.041404
C2orf76	-0.84116	3.72E-07	1.66E-06
EXTL3	-0.84115	8.28E-53	2.73E-51
AK4	-0.8409	2.83E-46	7.91E-45
CTB-161K23.1	-0.84044	0.014113	0.033672
NDOR1	-0.84016	1.33E-15	1.09E-14
MRPS26	-0.84013	1.06E-29	1.68E-28

MVB12B	-0.83966	4.72E-15	3.75E-14
CD19	-0.83916	0.019205	0.044416
FBXO45	-0.83906	6.03E-39	1.32E-37
SFXN5	-0.83899	1.48E-18	1.43E-17
CPN1	-0.83884	0.0044615	0.011813
ZNF813	-0.83824	2.15E-11	1.34E-10
KLHL3	-0.83821	5.63E-05	0.00019942
ZNF257	-0.83798	1.15E-10	6.80E-10
RP11-529K1.2	-0.83757	0.011459	0.02793
SHMT1	-0.837	7.61E-34	1.40E-32
C11orf54	-0.8368	1.35E-30	2.20E-29
PKP1	-0.83659	1.01E-15	8.35E-15
WT1	-0.83639	9.63E-14	7.07E-13
GAS2L1	-0.8361	9.26E-26	1.26E-24
PPIF	-0.83606	1.28E-74	7.36E-73
MUTYH	-0.83565	3.09E-15	2.48E-14
RP11-424C20.2	-0.83533	0.00032883	0.0010507
ANTXR1	-0.83457	1.55E-08	7.81E-08
LRTOMT	-0.83431	2.08E-21	2.30E-20
CSDE1	-0.83393	4.58E-68	2.23E-66
CPM	-0.83241	0.00026693	0.00086217
VPS52	-0.83206	7.07E-43	1.77E-41
GPRASP2	-0.83181	5.36E-19	5.33E-18
PLAUR	-0.83174	0.00089681	0.0026801
FBXL14	-0.83162	1.06E-11	6.76E-11
PIGV	-0.83158	1.26E-24	1.63E-23
SPC24	-0.83144	6.47E-26	8.91E-25
MDGA2	-0.83121	0.00012536	0.00042419
S100P	-0.83027	0.0064397	0.016526
DOK7	-0.82951	0.0049471	0.012968
G3BP1	-0.82926	2.18E-68	1.08E-66
NGDN	-0.82909	1.49E-22	1.73E-21
SNAPIN	-0.82899	1.77E-38	3.80E-37
CA5B	-0.82887	8.09E-06	3.16E-05
AC007551.3	-0.82847	0.011425	0.027854
UGT2B17	-0.82835	7.05E-05	0.00024608
AMACR	-0.82832	0.0018121	0.0051339
RABEP1	-0.8281	7.51E-44	1.93E-42
RP11-610P16.1	-0.82806	0.0037868	0.010156
ACSF2	-0.82794	4.85E-11	2.95E-10
AGPAT5	-0.8279	1.16E-39	2.62E-38
AKAP12	-0.82753	1.90E-11	1.19E-10
FAM208A	-0.82749	4.71E-32	8.17E-31
MFAP3L	-0.82743	0.00018386	0.00060972
ABCA3	-0.82701	5.74E-41	1.35E-39

IQCH	-0.82664	6.28E-07	2.73E-06
HES4	-0.82662	5.41E-12	3.53E-11
ECI1	-0.8262	2.03E-32	3.56E-31
RBMXL2	-0.82582	0.017694	0.041222
LINC00649	-0.82552	3.28E-07	1.47E-06
RP5-1125A11.1	-0.82547	0.0032589	0.008854
ZNF718	-0.82542	5.39E-11	3.27E-10
FKBP5	-0.82465	2.64E-66	1.22E-64
AC104651.1	-0.82438	0.020129	0.04632
USP46-AS1	-0.82428	5.03E-06	2.00E-05
RP11-359B12.1	-0.82414	0.0029869	0.0081618
POFUT1	-0.82404	1.29E-48	3.84E-47
DOK1	-0.82348	2.92E-08	1.44E-07
RASAL1	-0.82327	0.0074213	0.018805
SPHK2	-0.82324	6.37E-19	6.29E-18
BRSK2	-0.82322	6.85E-05	0.00023971
FHOD1	-0.82271	6.21E-09	3.25E-08
AUH	-0.82233	5.97E-18	5.56E-17
LRBA	-0.82219	2.55E-45	6.93E-44
RP11-671C19.1	-0.82079	3.60E-49	1.09E-47
CHD1L	-0.82037	2.51E-42	6.17E-41
RP11-692D12.1	-0.8199	0.0138	0.033025
RP3-422G23.4	-0.81931	8.81E-11	5.25E-10
UCP2	-0.819	8.28E-08	3.92E-07
GDPD1	-0.81882	7.22E-09	3.75E-08
RP11-671C19.2	-0.81815	8.42E-11	5.03E-10
RP6-65G23.3	-0.81761	0.00072898	0.0022117
RNF166	-0.81691	1.11E-13	8.13E-13
OR1F1	-0.81663	0.0069252	0.017658
AL355312.1	-0.81633	0.0082824	0.020799
LRSAM1	-0.81581	8.51E-26	1.16E-24
ATP12A	-0.81488	0.0015603	0.0044728
GPD1L	-0.81479	8.89E-38	1.84E-36
GRID2IP	-0.81455	0.009989	0.024667
PON1	-0.81451	3.19E-39	7.05E-38
RP4-657E11.10	-0.81442	3.39E-05	0.00012289
RP11-177C12.1	-0.81439	0.0088145	0.021994
AC130352.1	-0.81439	0.00043597	0.0013694
SVOP	-0.8142	0.0048523	0.012749
RP11-628E19.4	-0.8137	0.018902	0.043769
ZSWIM5	-0.81281	5.86E-08	2.81E-07
SPR	-0.81271	1.63E-44	4.35E-43
CAB39L	-0.81263	3.95E-19	3.96E-18
FANCE	-0.81238	6.84E-08	3.27E-07
KRT18	-0.81157	1.35E-47	3.89E-46

CAB39	-0.81037	8.68E-37	1.76E-35
DTNB	-0.81032	3.13E-12	2.07E-11
CCDC88B	-0.81028	0.0011034	0.0032471
NCALD	-0.81008	4.86E-06	1.94E-05
CMTM8	-0.80956	4.99E-09	2.63E-08
CTD-2291D10.4	-0.80898	0.002303	0.0064277
HPSE	-0.80895	1.76E-09	9.52E-09
HK2	-0.80877	7.33E-44	1.89E-42
FAM86C1	-0.80865	4.86E-07	2.14E-06
SUDS3	-0.80787	4.17E-61	1.68E-59
RP11-73M7.1	-0.80668	1.02E-06	4.35E-06
POM121	-0.80656	7.58E-32	1.30E-30
SF3B3	-0.80614	7.99E-64	3.43E-62
OLFM2	-0.80587	6.51E-07	2.82E-06
SYCP3	-0.8056	0.00070679	0.0021478
BAIAP2-AS1	-0.80559	9.41E-19	9.22E-18
TRIM46	-0.80478	0.001426	0.0041112
PFAS	-0.80462	2.54E-30	4.14E-29
UROS	-0.80339	3.25E-41	7.74E-40
RP11-644F5.10	-0.80334	0.0013595	0.0039319
RP5-1061H20.4	-0.80183	0.00022233	0.00072683
RP4-740C4.6	-0.8018	7.40E-07	3.19E-06
TOE1	-0.80176	1.86E-12	1.25E-11
RP11-488C13.5	-0.80158	0.00042431	0.0013354
MLXIPL	-0.80066	0.00044082	0.001384
FAM214B	-0.80066	1.02E-24	1.32E-23
FAM50B	-0.80064	8.31E-22	9.36E-21
NOL11	-0.80058	5.60E-35	1.07E-33
THAP8	-0.80048	0.0011334	0.0033258
C11orf31	-0.79953	2.53E-28	3.82E-27
CA14	-0.79867	0.0060321	0.015567
ASAH2B	-0.7984	1.42E-10	8.32E-10
ATP2B2	-0.79793	0.020591	0.047253
IPO8	-0.79723	1.75E-52	5.70E-51
TMEM214	-0.79528	8.67E-31	1.43E-29
PSMB7	-0.79493	1.34E-46	3.80E-45
FSTL4	-0.79465	0.013448	0.032275
KHDRBS2	-0.79455	9.31E-08	4.39E-07
GIT1	-0.79388	7.37E-38	1.54E-36
MLLT1	-0.79374	3.75E-23	4.51E-22
MBOAT7	-0.79357	1.49E-42	3.69E-41
FZR1	-0.79355	8.32E-26	1.14E-24
GTF2F2	-0.79352	2.01E-27	2.93E-26
PTGR2	-0.79324	1.25E-12	8.57E-12
ARHGAP32	-0.79287	2.20E-10	1.27E-09

DSN1	-0.79264	5.63E-22	6.41E-21
PIM3	-0.79252	1.12E-38	2.40E-37
FAM58A	-0.79244	1.34E-13	9.77E-13
MCIDAS	-0.79223	0.016152	0.037977
ACBD5	-0.79219	3.16E-42	7.73E-41
ABHD15	-0.7915	8.24E-11	4.93E-10
INMT	-0.79148	0.0039614	0.01057
FEZF1	-0.79108	0.012776	0.030828
NR1I3	-0.79077	0.0014033	0.0040508
KL	-0.79034	0.010348	0.025465
CHUK	-0.79034	2.72E-27	3.94E-26
TTLL12	-0.79027	4.83E-39	1.06E-37
TRPC4AP	-0.79005	7.18E-36	1.42E-34
APH1A	-0.78954	6.42E-72	3.46E-70
SLC25A43	-0.7888	2.40E-19	2.43E-18
TMEM98	-0.78816	6.70E-26	9.21E-25
RP11-1070N10.7	-0.78799	0.016018	0.037722
NLRX1	-0.78728	1.42E-20	1.52E-19
ZNF714	-0.78717	3.73E-24	4.71E-23
ATRN	-0.78712	1.50E-37	3.10E-36
PLXNA2	-0.78687	1.03E-16	8.97E-16
VAC14	-0.78685	8.83E-20	9.13E-19
NDRG4	-0.78634	3.90E-17	3.49E-16
RP11-229E13.2	-0.78527	0.003644	0.009805
RRM2	-0.78477	1.05E-52	3.44E-51
FZD6	-0.78474	9.48E-41	2.21E-39
QDPR	-0.78462	1.34E-12	9.17E-12
MRRF	-0.78448	1.70E-25	2.29E-24
MCRS1	-0.78353	2.32E-45	6.30E-44
TEAD2	-0.78319	1.03E-23	1.28E-22
PSME2	-0.78315	8.84E-38	1.84E-36
CTC-559E9.8	-0.78294	0.0022445	0.0062762
INTS1	-0.78256	4.20E-30	6.78E-29
TMEM100	-0.78247	0.010771	0.026427
CCHCR1	-0.7824	6.88E-27	9.79E-26
SUOX	-0.78215	6.50E-67	3.02E-65
TRAPPC12-AS1	-0.78196	0.016215	0.03811
SCAP	-0.78147	5.59E-44	1.45E-42
ATP13A2	-0.78059	5.31E-33	9.51E-32
ARHGAP21	-0.78032	1.11E-31	1.89E-30
DMBX1	-0.78007	0.021742	0.049632
AL807752.1	-0.77994	0.00081268	0.0024456
RARB	-0.77962	6.82E-06	2.68E-05
TSACC	-0.77927	0.0047547	0.012524
SPATA5	-0.77885	5.93E-09	3.10E-08

ZNF599	-0.77867	0.00039878	0.0012592
DOCK11	-0.7786	0.0033698	0.0091312
HAGHL	-0.77783	1.74E-17	1.60E-16
IDH1-AS1	-0.77778	0.0025377	0.0070251
ABHD1	-0.77752	0.00012668	0.00042845
PPP4R2	-0.77614	2.54E-30	4.14E-29
NAGA	-0.77552	9.04E-18	8.36E-17
HP1BP3	-0.77465	4.66E-60	1.82E-58
UNC5C	-0.77463	6.86E-05	0.00023997
ZNF570	-0.77463	1.03E-06	4.38E-06
ASIP	-0.7741	0.016404	0.038504
AC073236.3	-0.77391	0.0024005	0.0066828
SLC30A3	-0.77384	0.01319	0.031717
FEM1AP2	-0.77383	0.012583	0.03042
HEXIM2	-0.77378	0.0084498	0.021158
NDRG1	-0.77374	5.22E-30	8.40E-29
ZNF699	-0.77313	0.0024345	0.0067685
TMEM104	-0.77206	9.82E-11	5.83E-10
LAMB3	-0.77184	3.90E-07	1.73E-06
CHAF1B	-0.77158	3.30E-15	2.65E-14
EBNA1BP2	-0.77107	2.35E-39	5.24E-38
TMSB4Y	-0.77097	0.00018715	0.00061985
DDIT4L	-0.77071	1.49E-26	2.09E-25
C7orf13	-0.7706	0.015497	0.036632
PKHD1	-0.77031	0.0060996	0.015723
ADPRH	-0.77016	0.0053515	0.013951
CRKL	-0.77001	7.51E-59	2.83E-57
RIBC2	-0.76999	3.88E-07	1.72E-06
BORA	-0.76955	1.34E-10	7.88E-10
SMPD4	-0.76953	1.31E-17	1.20E-16
FBXL19-AS1	-0.76947	4.79E-15	3.80E-14
C8orf33	-0.76901	3.45E-60	1.35E-58
TIGD4	-0.76895	0.017924	0.041732
PLEKHG2	-0.7689	4.36E-11	2.66E-10
RP11-296O14.3	-0.76886	0.01505	0.035691
RP11-712L6.5	-0.76871	0.0032498	0.0088303
SUPT5H	-0.76798	7.37E-50	2.28E-48
COPZ1	-0.76791	1.72E-69	8.70E-68
RP4-693M11.3	-0.76772	4.60E-05	0.00016444
RAD9A	-0.76691	2.33E-09	1.25E-08
PRR5	-0.76676	3.14E-07	1.41E-06
GALK1	-0.76644	1.35E-14	1.05E-13
TMED10P2	-0.76625	0.0040222	0.010718
RP1-50O24.6	-0.76603	0.00071781	0.0021797
RP11-51F16.9	-0.76551	0.010558	0.025945

MYL12A	-0.76548	6.01E-31	9.95E-30
KLHDC9	-0.76531	1.74E-18	1.68E-17
CCDC92	-0.76444	7.34E-18	6.82E-17
LRFN4	-0.76345	1.01E-07	4.76E-07
MYL5	-0.76284	2.55E-12	1.70E-11
C1orf122	-0.76234	2.39E-21	2.64E-20
CLDN4	-0.76203	1.60E-32	2.83E-31
HSPA5	-0.76192	2.44E-73	1.35E-71
SLC22A31	-0.7611	0.012085	0.029333
AP5B1	-0.76062	1.32E-11	8.40E-11
C16orf13	-0.76053	1.16E-23	1.43E-22
TMED1	-0.76012	6.06E-06	2.39E-05
JAGN1	-0.7601	2.20E-29	3.45E-28
FCHO1	-0.75851	1.03E-08	5.26E-08
TNFRSF1A	-0.7585	1.11E-38	2.39E-37
KDELR3	-0.75837	7.25E-08	3.45E-07
RP11-1C8.4	-0.75785	6.39E-06	2.52E-05
SMU1	-0.75743	6.70E-44	1.73E-42
LINC00242	-0.75686	0.0053124	0.013856
RPS6KL1	-0.75673	1.53E-11	9.66E-11
HDAC11	-0.75633	1.01E-05	3.90E-05
C11orf80	-0.75597	2.26E-05	8.36E-05
THBS4	-0.75556	0.02042	0.046918
CORO1A	-0.75501	0.0019653	0.0055405
ELP4	-0.75481	3.36E-11	2.07E-10
CSRNP1	-0.75447	2.08E-21	2.30E-20
RP11-694I15.7	-0.75396	0.0046088	0.012166
RPS15A	-0.7537	1.33E-13	9.67E-13
ZNF273	-0.75367	1.38E-15	1.13E-14
RP11-509E16.1	-0.75339	0.016337	0.038364
RP11-696N14.1	-0.75317	0.018788	0.04354
PCCB	-0.75311	1.85E-65	8.27E-64
AC009237.11	-0.75297	0.020281	0.046624
TRIM47	-0.7528	3.00E-12	1.99E-11
DGKE	-0.75276	2.09E-12	1.40E-11
DENND4A	-0.75234	2.43E-19	2.46E-18
ZNF573	-0.75215	8.78E-06	3.41E-05
FAM63A	-0.75127	5.11E-20	5.35E-19
DENND1C	-0.75119	1.84E-22	2.14E-21
LIMK2	-0.75092	2.23E-22	2.58E-21
B4GALNT3	-0.75051	3.84E-12	2.52E-11
AP5S1	-0.75047	2.43E-10	1.40E-09
AL161915.1	-0.75037	0.010621	0.026092
FAM222B	-0.74993	2.62E-26	3.66E-25
FMNL1	-0.74964	8.39E-06	3.26E-05

SEC13	-0.74948	4.14E-41	9.82E-40
TCEANC2	-0.74902	1.09E-09	5.99E-09
ADAL	-0.74901	1.72E-14	1.32E-13
CCDC28B	-0.74889	2.44E-16	2.09E-15
ITGB1BP1	-0.74889	8.56E-21	9.22E-20
STAG3L3	-0.7488	1.03E-05	3.97E-05
C4orf19	-0.7485	0.0058839	0.015213
MCM6	-0.74849	3.79E-29	5.89E-28
ZNF492	-0.74791	1.49E-08	7.51E-08
KCNH6	-0.74786	3.68E-11	2.26E-10
TPX2	-0.74782	2.53E-41	6.06E-40
NOTUM	-0.74716	0.020177	0.046421
BAG4	-0.7468	5.72E-25	7.49E-24
ZNF81	-0.74628	6.17E-09	3.22E-08
MFSD9	-0.74559	7.85E-11	4.70E-10
ZNF701	-0.74544	2.95E-07	1.33E-06
PRDM4	-0.74534	5.30E-24	6.65E-23
PCED1A	-0.74516	7.34E-17	6.46E-16
ZNF66	-0.74499	6.58E-05	0.00023068
DERA	-0.74481	1.23E-16	1.07E-15
TBC1D17	-0.74451	2.08E-16	1.79E-15
GINS4	-0.74448	1.04E-19	1.07E-18
CLSPN	-0.7444	1.57E-13	1.14E-12
TMEM209	-0.74406	1.10E-29	1.75E-28
VIM	-0.74386	1.12E-06	4.76E-06
MT3	-0.74366	0.008372	0.021001
CCDC47	-0.74308	2.19E-43	5.58E-42
DLGAP1	-0.74307	2.01E-10	1.17E-09
HAPLN3	-0.74297	4.67E-14	3.50E-13
HADH	-0.74269	2.59E-43	6.57E-42
TSPAN10	-0.74259	0.0047222	0.012445
C9orf114	-0.74192	7.09E-34	1.30E-32
HEG1	-0.74181	1.18E-05	4.50E-05
BRMS1	-0.74169	7.65E-22	8.64E-21
ASS1P12	-0.74163	0.003279	0.0089051
KIAA0922	-0.7416	2.22E-10	1.28E-09
GGH	-0.74123	9.37E-41	2.19E-39
CBY1	-0.7412	2.75E-11	1.70E-10
CARD10	-0.74101	1.10E-19	1.13E-18
RMND1	-0.74072	8.73E-11	5.20E-10
RP11-497H16.9	-0.7406	0.019273	0.044544
ORC1	-0.74045	1.69E-14	1.31E-13
HIST3H2A	-0.74021	6.06E-12	3.94E-11
PDIA3P	-0.7402	1.02E-05	3.93E-05
AMZ2	-0.7402	2.14E-29	3.36E-28

PRPSAP1	-0.74013	3.84E-18	3.62E-17
TRAM1L1	-0.74007	1.66E-09	8.99E-09
TMED6	-0.73976	0.0069727	0.017771
SFXN3	-0.73975	5.88E-29	9.09E-28
ABCE1	-0.73939	3.15E-35	6.05E-34
SNAI3	-0.73925	0.0065108	0.016696
ARID3C	-0.73748	0.01885	0.043674
CNEP1R1	-0.73698	5.04E-09	2.65E-08
DDX23	-0.73611	1.19E-50	3.80E-49
RP11-464F9.20	-0.73592	0.011877	0.028872
PPM1H	-0.73571	2.11E-25	2.82E-24
RP11-254F7.2	-0.73497	0.0016069	0.0045961
NSUN4	-0.73484	1.40E-20	1.49E-19
SLC39A13	-0.73461	1.50E-06	6.25E-06
SPDYC	-0.73435	0.017394	0.040591
GTF3C1	-0.73424	4.96E-27	7.09E-26
SNHG9	-0.73421	0.016212	0.03811
EI24	-0.73348	4.41E-54	1.51E-52
TTC30A	-0.73326	0.0008219	0.0024708
SART1	-0.733	2.45E-34	4.60E-33
AC006538.1	-0.73286	0.010274	0.025301
EMILIN1	-0.73274	0.0047098	0.012415
ASB16	-0.73272	5.83E-06	2.31E-05
TTLL6	-0.73204	0.013932	0.033295
RP11-66B24.2	-0.73143	0.021414	0.048947
DDX41	-0.73137	5.33E-34	9.82E-33
CTC-228N24.3	-0.73087	4.40E-08	2.13E-07
TRPC3	-0.73052	0.0080863	0.020358
TIMM13	-0.73043	1.24E-19	1.27E-18
RAB3D	-0.72972	2.59E-21	2.86E-20
SLC27A5	-0.72957	6.86E-11	4.13E-10
GALK2	-0.72928	4.33E-14	3.26E-13
SLC25A20	-0.72886	3.51E-19	3.53E-18
AL357673.1	-0.72873	7.39E-07	3.19E-06
ZNF426	-0.72856	5.37E-09	2.82E-08
AKR1C2	-0.72833	2.58E-31	4.35E-30
ANGEL1	-0.72761	3.43E-18	3.25E-17
MCMDC2	-0.72639	6.07E-08	2.91E-07
C10orf107	-0.72623	0.02034	0.046749
GYS1	-0.7258	2.97E-32	5.16E-31
ACOT1	-0.72571	0.0068939	0.01758
CCDC11	-0.72504	0.019365	0.044729
DNAH10OS	-0.72471	0.013656	0.032713
KLKP1	-0.72443	0.00019147	0.00063344
ZNF708	-0.72435	7.92E-13	5.49E-12

PAG1	-0.72398	1.11E-11	7.06E-11
AC010642.1	-0.72363	0.0091341	0.022728
CPT1C	-0.72357	1.50E-10	8.79E-10
KLK3	-0.72352	0.002438	0.0067772
WDFY2	-0.72274	3.27E-08	1.60E-07
ZNF776	-0.72254	2.92E-11	1.81E-10
MOCS2	-0.7223	8.25E-23	9.73E-22
MID1IP1-AS1	-0.72203	0.0049629	0.013008
KB-1460A1.1	-0.72174	0.015169	0.035949
AC092431.1	-0.72103	0.011248	0.027465
BLVRB	-0.72089	2.43E-20	2.58E-19
CATSPER2P1	-0.7207	0.0026436	0.0072921
Z83851.4	-0.71997	0.0038175	0.010231
LEPREL4	-0.71989	2.73E-29	4.27E-28
BCMO1	-0.71835	0.0017839	0.0050591
HSD11B1L	-0.71799	2.33E-05	8.62E-05
IGSF11	-0.71794	0.0024417	0.0067848
UBBP4	-0.71766	6.89E-05	0.00024105
SLC24A1	-0.71747	5.75E-10	3.24E-09
MRPL53	-0.71735	0.00061355	0.0018799
PTPN23	-0.71729	6.42E-27	9.15E-26
NDST2	-0.71704	6.84E-13	4.77E-12
DLG2	-0.71638	2.43E-05	8.95E-05
WIPF3	-0.71637	0.00080485	0.0024238
GAS6-AS1	-0.7162	0.00094421	0.0028104
MAGT1	-0.71535	1.43E-41	3.45E-40
RTKN	-0.71529	3.07E-30	4.99E-29
AARS2	-0.71468	7.23E-20	7.50E-19
KANK2	-0.7146	1.24E-23	1.53E-22
RP11-181C3.1	-0.71383	0.0031162	0.0084951
SLC25A34	-0.71366	0.013367	0.032107
SPHK1	-0.71351	0.00023655	0.0007704
KIFAP3	-0.7134	1.72E-29	2.71E-28
TIRAP	-0.71322	2.56E-07	1.16E-06
PRRC2C	-0.71313	1.57E-36	3.15E-35
FIBP	-0.71303	6.34E-33	1.13E-31
TM9SF1	-0.71259	0.0025979	0.0071785
ARL1	-0.71247	1.64E-36	3.27E-35
RP11-983P16.4	-0.71127	8.20E-15	6.43E-14
SMC1A	-0.71114	6.00E-40	1.37E-38
ZNF512	-0.71101	6.15E-13	4.31E-12
CTD-2017C7.2	-0.71092	0.019141	0.044298
RP11-38L15.3	-0.71072	0.0067629	0.017276
PHF21B	-0.7103	4.60E-05	0.00016427
SORBS2	-0.70993	1.25E-09	6.84E-09

TMEM126A	-0.70993	2.18E-12	1.47E-11
ZNF259	-0.7099	2.41E-20	2.55E-19
ARHGAP39	-0.70972	3.59E-16	3.05E-15
RP11-438N16.1	-0.70937	1.31E-19	1.34E-18
TRIM13	-0.70934	1.54E-13	1.12E-12
RP1-261D10.2	-0.70888	0.0097632	0.02415
ZNF665	-0.70876	0.00083166	0.0024976
HYOU1	-0.70858	1.66E-43	4.23E-42
DPYSL2	-0.70848	8.17E-50	2.52E-48
SNAPC3	-0.70834	7.80E-22	8.79E-21
RASGRP1	-0.70781	1.66E-05	6.25E-05
AC098973.2	-0.70774	8.60E-06	3.34E-05
VPS18	-0.70761	5.94E-16	4.99E-15
HPGD	-0.70759	9.50E-30	1.51E-28
GRPEL1	-0.70751	6.55E-35	1.25E-33
TNNC1	-0.70731	0.0026365	0.0072753
NELFB	-0.7073	2.52E-28	3.80E-27
LRFN3	-0.70711	3.91E-08	1.91E-07
CDC25A	-0.70692	8.76E-25	1.14E-23
SRRM2-AS1	-0.70639	0.00043286	0.0013604
AKAP9	-0.70615	1.16E-48	3.45E-47
BCS1L	-0.70551	6.51E-26	8.96E-25
SLC11A1	-0.70538	0.0042897	0.011393
ZNF438	-0.70529	0.00026366	0.00085242
PSRC1	-0.70519	9.06E-12	5.82E-11
ELP5	-0.70506	3.43E-28	5.15E-27
KIAA1161	-0.705	4.72E-27	6.76E-26
ZSCAN22	-0.70487	2.02E-08	1.01E-07
TSPAN6	-0.70482	4.99E-10	2.82E-09
PPP1R15B	-0.70481	1.63E-45	4.45E-44
PSD	-0.70465	7.73E-10	4.32E-09
GALNT7	-0.70412	1.58E-24	2.02E-23
CASP7	-0.70401	1.41E-15	1.15E-14
NLRP1	-0.70395	9.78E-14	7.18E-13
WBSCR27	-0.70342	0.0012184	0.0035587
CCDC167	-0.70337	1.45E-12	9.90E-12
SNRPC	-0.70314	2.31E-31	3.90E-30
PRKX	-0.70284	4.61E-33	8.29E-32
PHRF1	-0.70265	8.51E-20	8.81E-19
PCYT1A	-0.70203	4.09E-29	6.34E-28
PROSER1	-0.70199	4.31E-36	8.56E-35
CTA-29F11.1	-0.70196	0.0041475	0.011028
CTC-523E23.11	-0.70177	0.010833	0.026574
FGFR1	-0.70069	0.00018426	0.00061075
PNPLA6	-0.70043	1.16E-19	1.19E-18

GRIK5	-0.70013	0.0031426	0.0085592
RTN4	-0.6995	2.63E-28	3.96E-27
RSL24D1	-0.69937	4.70E-27	6.75E-26
NFE2L1	-0.69921	5.28E-50	1.64E-48
AP2A1	-0.69919	1.04E-28	1.60E-27
NBEAL2	-0.69901	1.47E-23	1.81E-22
KCTD5	-0.69881	1.56E-28	2.37E-27
CSRNP3	-0.6984	6.46E-15	5.10E-14
PACSIN1	-0.69807	2.68E-21	2.96E-20
AC004540.5	-0.69792	0.010348	0.025465
PSMG3	-0.6977	1.12E-16	9.74E-16
GBA	-0.69715	3.45E-33	6.21E-32
BIN1	-0.69713	0.020071	0.046215
RDH5	-0.69672	0.0023339	0.0065097
ZNF197	-0.6967	7.49E-13	5.20E-12
TMEM183A	-0.6959	8.75E-51	2.80E-49
RIMS3	-0.69589	1.68E-06	6.99E-06
KB-1507C5.2	-0.69588	0.00056694	0.0017479
NRTN	-0.69548	0.0098344	0.024314
MFSD1	-0.69518	1.37E-26	1.93E-25
SNF8	-0.69463	8.35E-09	4.31E-08
COL4A5	-0.6942	2.28E-23	2.79E-22
LINC00271	-0.69402	0.020975	0.048038
RP11-522I20.3	-0.69378	0.00015138	0.00050705
AC091801.1	-0.69357	0.012808	0.030897
C11orf65	-0.69356	0.0020955	0.0058868
RP11-61J19.4	-0.69295	4.54E-06	1.82E-05
SETD9	-0.69271	2.06E-06	8.49E-06
GPAT2	-0.69108	1.45E-19	1.48E-18
ESPL1	-0.69095	6.55E-23	7.75E-22
MYEOV	-0.69041	1.39E-06	5.83E-06
SYNJ2BP	-0.69033	5.83E-14	4.34E-13
HSP90AB1	-0.68993	4.63E-53	1.53E-51
MAP2K6	-0.68959	1.64E-09	8.90E-09
RORC	-0.68954	0.00078689	0.0023735
AASDH	-0.68951	8.20E-12	5.28E-11
TP53INP1	-0.68905	1.08E-44	2.89E-43
PDZD7	-0.689	0.008721	0.021785
BRI3BP	-0.68855	2.03E-46	5.71E-45
UQCC	-0.68853	3.85E-25	5.07E-24
ATP7A	-0.6885	1.49E-15	1.22E-14
PLCXD1	-0.68841	8.60E-29	1.33E-27
FAM27A	-0.68833	0.012347	0.029924
TUBGCP4	-0.68823	5.60E-18	5.23E-17
C6orf136	-0.68747	5.82E-11	3.52E-10

ALKBH3	-0.68667	2.97E-10	1.71E-09
NDFIP1	-0.6865	2.35E-33	4.24E-32
ANKRD13C	-0.6863	1.14E-18	1.11E-17
SMCO4	-0.68626	2.51E-25	3.34E-24
FAM86B3P	-0.68592	0.0049898	0.013069
NUDT17	-0.68543	8.35E-11	4.99E-10
SLC39A3	-0.6851	1.86E-11	1.17E-10
FPGT	-0.68494	2.44E-08	1.21E-07
CBX8	-0.68464	2.50E-12	1.66E-11
RDH11	-0.68435	3.30E-34	6.14E-33
14-Sep	-0.68417	0.015796	0.037267
SMIM7	-0.68389	3.79E-13	2.68E-12
MYRIP	-0.68386	1.75E-18	1.69E-17
USP9Y	-0.68364	1.12E-14	8.75E-14
CECR6	-0.68356	9.23E-07	3.94E-06
CAMKK2	-0.68351	6.14E-37	1.25E-35
SRGAP2C	-0.68347	6.28E-05	0.000221
DERL3	-0.68331	3.99E-10	2.27E-09
B3GALT4	-0.6829	0.00034808	0.0011082
TBCA	-0.68285	3.74E-34	6.92E-33
DPYD	-0.68209	0.00036476	0.0011576
UPP1	-0.68197	5.34E-05	0.00018961
CLEC11A	-0.68182	0.0021799	0.006108
C5orf58	-0.68151	0.00081764	0.0024587
ZFYVE19	-0.68141	8.12E-16	6.76E-15
IKZF2	-0.68059	5.31E-07	2.33E-06
RAB11FIP4	-0.68018	1.50E-28	2.29E-27
PPP1R18	-0.67957	1.24E-07	5.78E-07
RP11-357C3.3	-0.67925	0.00031773	0.0010172
ANAPC16	-0.67891	2.30E-35	4.45E-34
APPL2	-0.67879	3.39E-22	3.89E-21
RPS6KB1	-0.67866	1.76E-19	1.79E-18
MAPK8IP2	-0.67848	7.76E-27	1.10E-25
BZW1	-0.67831	3.35E-30	5.42E-29
RNF123	-0.67811	9.54E-28	1.41E-26
RP11-343N15.5	-0.67733	1.42E-10	8.31E-10
WDR11	-0.67551	2.26E-24	2.89E-23
RP5-1157M23.2	-0.67501	0.002961	0.0081007
CNNM4	-0.67468	1.28E-21	1.43E-20
ZNF726	-0.67447	1.16E-06	4.91E-06
SLC47A2	-0.67403	2.91E-05	0.00010637
NME3	-0.6738	1.97E-05	7.35E-05
RGS11	-0.67299	4.14E-08	2.01E-07
KIAA0100	-0.67245	7.21E-33	1.28E-31
WDR17	-0.67204	9.60E-14	7.05E-13

TCEB1	-0.67195	9.63E-30	1.53E-28
GAA	-0.67136	1.04E-28	1.60E-27
DLEU1	-0.67133	6.78E-10	3.80E-09
PAXBP1-AS1	-0.67121	0.0038975	0.010424
SLC35F6	-0.67105	7.29E-17	6.42E-16
VIPAS39	-0.67088	3.43E-15	2.75E-14
DCTN3	-0.67082	7.41E-31	1.22E-29
POGLUT1	-0.67049	7.15E-11	4.30E-10
RP11-1094M14.11	-0.67045	0.0012351	0.0036022
GALNT10	-0.6704	1.72E-22	2.00E-21
ARHGEF28	-0.67018	4.12E-05	0.00014795
NUMA1	-0.67014	1.96E-39	4.38E-38
SEC14L4	-0.67004	2.30E-05	8.50E-05
OSGIN1	-0.66987	2.64E-05	9.69E-05
MAPK11	-0.66873	3.25E-22	3.73E-21
PLCH2	-0.66803	0.00582	0.015074
CSNK1E	-0.66788	1.68E-38	3.60E-37
DTWD1	-0.66755	1.15E-08	5.87E-08
VPS39	-0.66737	1.66E-28	2.53E-27
KIF3A	-0.66732	1.05E-17	9.72E-17
MTO1	-0.66729	3.84E-12	2.52E-11
GPR156	-0.66723	0.012874	0.031029
SPDEF	-0.66715	5.39E-27	7.68E-26
LRP12	-0.66701	1.28E-18	1.24E-17
RP11-452L6.5	-0.66657	0.0015889	0.0045497
RP11-417J8.3	-0.66653	0.00010749	0.0003672
CENPQ	-0.66603	4.20E-07	1.86E-06
DNAJC4	-0.66551	1.54E-13	1.12E-12
PDP2	-0.6654	2.33E-14	1.78E-13
MED30	-0.66505	1.39E-13	1.01E-12
FASTK	-0.66457	2.22E-25	2.97E-24
CACNA2D2	-0.66408	2.05E-08	1.03E-07
GLA	-0.66407	3.81E-13	2.69E-12
HSPA1A	-0.66401	0.0025259	0.0069958
TNRC6B	-0.66343	1.53E-23	1.88E-22
WIF1	-0.66336	0.00057515	0.0017717
KDM4A-AS1	-0.66317	0.016954	0.039687
AACS	-0.66314	1.94E-31	3.28E-30
FBXW9	-0.66265	1.32E-08	6.70E-08
SDSL	-0.66261	1.38E-26	1.95E-25
PHF1	-0.6621	2.55E-18	2.43E-17
KLHL31	-0.66172	0.014137	0.033717
DNHD1	-0.66169	0.0002088	0.00068659
SLC7A8	-0.66169	8.17E-17	7.18E-16
SNX29	-0.66167	1.01E-08	5.20E-08

CYFIP2	-0.66158	2.07E-30	3.37E-29
MED27	-0.66152	2.00E-11	1.25E-10
KIAA0556	-0.66144	1.26E-12	8.64E-12
DENND6B	-0.66103	4.25E-05	0.0001525
PXN-AS1	-0.66096	0.00084673	0.0025407
ZNF765	-0.66091	7.23E-08	3.44E-07
CENPM	-0.66046	1.59E-17	1.46E-16
MBD2	-0.66034	1.60E-20	1.71E-19
FAM65A	-0.66025	1.26E-08	6.42E-08
AD000090.2	-0.66012	3.62E-05	0.00013068
PLEKHG6	-0.6599	1.49E-06	6.23E-06
ZNF185	-0.65931	2.90E-08	1.43E-07
RP11-705O3.1	-0.65894	0.012376	0.029982
ZNF737	-0.65781	5.94E-11	3.59E-10
RP11-488L18.10	-0.65767	7.40E-05	0.00025771
CTD-3099C6.9	-0.65752	0.00016068	0.00053646
VRK3	-0.657	2.61E-23	3.18E-22
AC010524.2	-0.65669	3.60E-07	1.60E-06
KIAA0895	-0.65649	1.58E-14	1.22E-13
RAB3GAP2	-0.6564	3.26E-25	4.31E-24
WDR88	-0.65598	0.0028386	0.0077948
DACH1	-0.6558	2.09E-05	7.77E-05
SLC51A	-0.65548	0.0069856	0.017797
NECAB3	-0.65547	8.92E-14	6.57E-13
ENTPD1-AS1	-0.65546	0.0020971	0.0058904
SYT5	-0.655	0.0037145	0.0099753
LPIN1	-0.65495	3.35E-23	4.05E-22
RP11-44F14.2	-0.65484	5.99E-06	2.37E-05
ARPC3	-0.65453	3.49E-25	4.61E-24
PLCD3	-0.65449	5.15E-10	2.91E-09
RAP1GAP2	-0.65397	5.09E-33	9.11E-32
NUDT8	-0.65311	2.07E-07	9.46E-07
PDZD4	-0.65215	0.016918	0.039623
CHIC1	-0.65152	6.35E-07	2.76E-06
SLC33A1	-0.65084	3.66E-23	4.41E-22
BIRC2	-0.65064	1.67E-18	1.61E-17
ZNF429	-0.65042	3.64E-12	2.39E-11
RP11-312O7.2	-0.65028	0.0097561	0.02414
CBR3	-0.64984	0.0018013	0.0051051
AMIGO1	-0.64965	1.22E-09	6.67E-09
TOM1L2	-0.6492	4.56E-23	5.46E-22
TTC38	-0.64878	1.51E-17	1.39E-16
RFXAP	-0.64854	5.58E-06	2.21E-05
ILF3-AS1	-0.64852	1.20E-20	1.29E-19
ZNF583	-0.64805	0.00065535	0.002

KLHL36	-0.64784	6.09E-18	5.67E-17
CDC14B	-0.6476	1.88E-07	8.60E-07
GNRHR2	-0.64736	0.0062815	0.01615
KIAA1407	-0.64733	9.49E-05	0.000326
C21orf67	-0.64623	0.00047236	0.0014773
RP11-395B7.7	-0.6461	1.17E-10	6.90E-10
AAED1	-0.64609	0.00030457	0.00097783
THAP7-AS1	-0.64552	0.0064505	0.016548
PTRF	-0.64513	0.00022333	0.00072978
TAF2	-0.64504	4.25E-27	6.11E-26
ZNF83	-0.64478	4.49E-11	2.74E-10
GSTA2	-0.64449	0.00449	0.011881
ISY1	-0.64424	1.38E-09	7.52E-09
CHD5	-0.64399	0.01202	0.029183
C19orf68	-0.64376	0.001416	0.0040841
AIF1L	-0.64357	3.58E-34	6.64E-33
COL4A4	-0.64298	0.016371	0.038435
RP5-1103G7.4	-0.6417	1.76E-07	8.07E-07
HMBS	-0.64149	5.09E-19	5.06E-18
C19orf12	-0.64137	3.27E-12	2.16E-11
EFCAB14	-0.64118	8.79E-33	1.56E-31
TIMP2	-0.6404	0.0030744	0.008389
STAT5B	-0.64029	4.41E-19	4.41E-18
PACSIN2	-0.64018	3.53E-26	4.91E-25
TOPBP1	-0.6399	3.43E-28	5.15E-27
TGFB3	-0.63952	0.00012716	0.00042979
RP4-798C17.6	-0.6393	0.0036658	0.0098561
LINC00471	-0.63928	0.017591	0.041004
FUCA2	-0.63924	3.51E-19	3.52E-18
DEGS2	-0.63913	0.01133	0.027645
DHFR	-0.63911	1.08E-30	1.77E-29
GAK	-0.63813	3.25E-21	3.58E-20
BOP1	-0.63771	1.09E-09	5.99E-09
THNSL2	-0.63764	4.59E-12	3.00E-11
CLTCL1	-0.63724	9.38E-05	0.00032251
RP11-21L23.2	-0.63719	0.006943	0.017699
CIAPIN1	-0.63701	5.07E-30	8.15E-29
CTA-204B4.2	-0.63636	0.0032339	0.0087905
IRAK4	-0.63614	5.74E-14	4.28E-13
RRNAD1	-0.63601	3.95E-15	3.16E-14
FAM115B	-0.63574	1.75E-06	7.27E-06
PTGES3	-0.63557	7.80E-34	1.43E-32
HOMER2	-0.63553	4.03E-27	5.79E-26
OPA3	-0.63519	1.14E-17	1.05E-16
IFT140	-0.63503	3.18E-07	1.43E-06

OGG1	-0.6349	2.96E-11	1.83E-10
BIRC3	-0.6345	0.00228	0.0063671
ZNF552	-0.63414	4.30E-07	1.90E-06
RP11-163N6.2	-0.63404	0.0072943	0.018504
UROD	-0.63395	3.40E-21	3.73E-20
ASUN	-0.63391	6.76E-25	8.84E-24
IMPACT	-0.63384	6.06E-23	7.18E-22
RP11-304L19.11	-0.63383	0.0013703	0.0039611
DDI2	-0.6338	3.66E-30	5.92E-29
WT1-AS	-0.63351	0.013278	0.031916
SBF2-AS1	-0.63332	0.017841	0.041543
ANAPC2	-0.63313	1.79E-13	1.29E-12
RAD51B	-0.63308	0.0012819	0.0037293
DKFZP761J1410	-0.63294	5.06E-18	4.74E-17
TRPM4	-0.63289	1.24E-20	1.33E-19
TRMT1	-0.63199	4.85E-19	4.83E-18
RNF8	-0.63198	2.21E-10	1.28E-09
NGEF	-0.63161	1.26E-09	6.89E-09
RASGRF2	-0.63143	0.017236	0.040258
ZNF85	-0.63109	2.02E-07	9.21E-07
NT5DC1	-0.63104	1.51E-19	1.54E-18
RP4-756H11.3	-0.63066	0.00035491	0.0011281
KCNC3	-0.63056	3.99E-05	0.00014361
AC092295.7	-0.63048	0.015232	0.036088
ZNF41	-0.63027	8.86E-10	4.93E-09
DPH5	-0.63004	8.04E-10	4.49E-09
FAM115A	-0.62958	3.73E-34	6.90E-33
RBM25	-0.62929	3.80E-33	6.83E-32
PPP1R9A	-0.62906	4.56E-18	4.28E-17
TPK1	-0.62831	0.00032439	0.0010373
TSC22D4	-0.62809	2.56E-17	2.33E-16
C3orf33	-0.62748	5.25E-10	2.97E-09
RP3-462D8.2	-0.62698	0.010025	0.024754
ENPEP	-0.62688	0.0094701	0.023495
HMGCL	-0.62644	8.10E-23	9.56E-22
DCDC2	-0.62583	0.019147	0.044306
KLHDC2	-0.62578	1.14E-24	1.48E-23
IQSEC2	-0.62558	8.13E-24	1.01E-22
DICER1-AS1	-0.62537	0.0016187	0.0046281
NDUFB1	-0.62504	4.75E-15	3.77E-14
SLC35F2	-0.62501	3.62E-19	3.64E-18
HINT2	-0.62447	2.78E-11	1.72E-10
PRPF40B	-0.62319	4.70E-08	2.27E-07
ZNF471	-0.62271	1.24E-06	5.24E-06
TBRG1	-0.62248	2.30E-12	1.54E-11

GTPBP10	-0.62234	4.91E-19	4.89E-18
ZC3H6	-0.62216	2.68E-12	1.78E-11
MAPK8IP1	-0.62167	1.12E-16	9.79E-16
RP11-649A18.12	-0.62155	0.0054503	0.014192
LARP6	-0.62133	3.35E-05	0.00012148
MIOS	-0.62122	1.43E-13	1.04E-12
BAG6	-0.62114	5.00E-33	8.96E-32
PDP1	-0.62072	3.26E-13	2.32E-12
LPIN2	-0.62058	4.25E-09	2.25E-08
RBKS	-0.62055	0.0029454	0.0080604
HIST1H2BD	-0.6197	0.0072229	0.018334
TTC21A	-0.61961	0.0042915	0.011397
SLC13A3	-0.61949	3.86E-12	2.54E-11
CRNKL1	-0.61932	2.04E-15	1.66E-14
DGKZ	-0.61891	8.20E-21	8.85E-20
PABPC4	-0.61874	4.58E-38	9.64E-37
RP11-677M14.7	-0.61828	0.0036832	0.009899
RBM3	-0.61807	8.32E-28	1.23E-26
PEX1	-0.6178	2.56E-16	2.18E-15
LETM1	-0.61714	9.08E-23	1.07E-21
TSC2	-0.61687	8.11E-21	8.76E-20
ARHGAP12	-0.61684	5.54E-10	3.13E-09
CLCN5	-0.61633	4.43E-15	3.53E-14
ITCH	-0.61631	6.76E-22	7.65E-21
PPP6C	-0.6157	8.37E-21	9.03E-20
ZNF514	-0.61547	0.00038547	0.0012196
YTHDF1	-0.61547	1.53E-25	2.07E-24
SH3RF1	-0.61522	1.25E-19	1.28E-18
TBC1D2B	-0.61503	2.70E-10	1.56E-09
TSTD3	-0.61477	0.0061107	0.015746
SCPEP1	-0.61462	1.73E-20	1.84E-19
ZNF480	-0.61446	2.72E-15	2.20E-14
DUT	-0.61442	2.41E-28	3.64E-27
FAM221A	-0.61436	0.00044929	0.0014082
PTPRE	-0.61432	2.54E-05	9.34E-05
SPTBN5	-0.61423	0.0050705	0.013268
HYAL3	-0.61383	2.01E-06	8.29E-06
ERLIN1	-0.6137	1.62E-20	1.73E-19
BRE	-0.61339	1.88E-12	1.27E-11
ACTN2	-0.61275	0.0048205	0.01268
VWA5B2	-0.61258	2.46E-11	1.53E-10
NBAS	-0.61237	2.72E-14	2.07E-13
COQ3	-0.61233	1.20E-12	8.23E-12
TMEM218	-0.6122	2.23E-09	1.20E-08
PPP1R3E	-0.61219	0.00093867	0.0027951

FBXW2	-0.61207	1.60E-14	1.23E-13
KLF12	-0.61206	0.0017384	0.004941
CCDC142	-0.61203	1.08E-08	5.54E-08
TMEM170A	-0.61196	3.91E-09	2.08E-08
ZNF501	-0.61174	0.0001112	0.000379
DNTTIP1	-0.61152	6.09E-13	4.26E-12
RFX2	-0.61144	0.0024595	0.0068278
ALPK3	-0.61136	1.04E-06	4.42E-06
GPC1	-0.61091	1.04E-06	4.43E-06
FCGBP	-0.61082	0.01109	0.027131
ZNF140	-0.61076	1.42E-11	8.98E-11
HEYL	-0.61013	0.011864	0.028843
CTD-2619J13.14	-0.61003	7.57E-14	5.60E-13
KRT8P12	-0.60988	3.21E-06	1.30E-05
TRERF1	-0.60977	3.97E-06	1.60E-05
KIAA1841	-0.60965	0.00025838	0.00083639
COLEC11	-0.60906	0.00025696	0.0008323
PUS3	-0.60881	6.93E-10	3.89E-09
KIF13A	-0.60869	1.20E-08	6.11E-08
RP11-566E18.3	-0.60835	2.95E-05	0.00010752
CNTD2	-0.6079	0.00099603	0.0029532
MTFMT	-0.60765	1.19E-10	7.03E-10
NHLRC2	-0.6076	1.97E-13	1.42E-12
SSPO	-0.607	8.93E-07	3.82E-06
PSMB10	-0.60675	0.012783	0.03084
ARF4	-0.60644	1.50E-24	1.93E-23
RSAD2	-0.60639	0.011168	0.027297
ZDBF2	-0.6063	7.80E-20	8.08E-19
CHRNA2	-0.60577	3.87E-11	2.37E-10
CLCN3	-0.60523	1.98E-21	2.20E-20
TFPI	-0.60507	2.96E-22	3.40E-21
ZFAT	-0.60469	1.41E-08	7.15E-08
PSMC3	-0.60447	4.64E-37	9.46E-36
TTC5	-0.60432	3.69E-13	2.61E-12
ID3	-0.60429	6.03E-32	1.04E-30
CAPS2	-0.60389	1.93E-08	9.67E-08
CYB561D2	-0.60382	1.55E-12	1.06E-11
NETO2	-0.60354	5.59E-17	4.96E-16
RPE	-0.60351	2.81E-16	2.40E-15
ZNF91	-0.6031	1.52E-18	1.47E-17
RP11-300J18.3	-0.60283	0.0014597	0.0041979
DNAJA1	-0.60204	8.30E-29	1.28E-27
LINGO1	-0.60194	6.00E-07	2.61E-06
ASPSCR1	-0.60191	1.89E-10	1.10E-09
TSPAN12	-0.60184	0.0057778	0.014978

RP11-280F2.2	-0.60135	0.0084924	0.021255
SLC9A1	-0.60107	5.89E-21	6.40E-20
TRMT10A	-0.60073	2.34E-12	1.56E-11
CCDC113	-0.60003	9.76E-10	5.41E-09
SLC2A6	-0.59959	0.00062077	0.0019006
CHST3	-0.59955	7.21E-19	7.11E-18
JOSD2	-0.59939	1.16E-05	4.43E-05
RAD51	-0.59927	1.55E-10	9.04E-10
COPE	-0.59901	6.70E-17	5.92E-16
SSH2	-0.59843	1.82E-13	1.32E-12
GPX8	-0.59778	5.45E-07	2.38E-06
CHM	-0.59696	2.04E-16	1.76E-15
ARHGEF37	-0.59692	1.77E-27	2.59E-26
TSTD2	-0.59649	6.64E-08	3.17E-07
STX2	-0.59648	4.05E-07	1.80E-06
TMEM184B	-0.59616	7.24E-12	4.68E-11
CHMP1A	-0.59611	7.07E-25	9.23E-24
NRBP2	-0.59598	2.75E-17	2.50E-16
MAF	-0.59525	0.0014422	0.0041537
RPP40	-0.59487	4.72E-08	2.28E-07
VASH2	-0.59476	7.84E-11	4.70E-10
C11orf71	-0.59458	7.00E-05	0.00024471
AC009403.2	-0.59449	0.0025563	0.0070719
KLC2	-0.59449	1.51E-22	1.76E-21
PSPN	-0.59446	0.01607	0.037806
FTL	-0.59402	5.84E-38	1.22E-36
USP54	-0.59386	1.83E-33	3.32E-32
NIT1	-0.59383	3.21E-14	2.44E-13
TLDC2	-0.59371	6.58E-07	2.85E-06
EBF2	-0.59351	0.0081152	0.020426
VPS53	-0.59323	8.43E-14	6.22E-13
CTDNEP1	-0.59323	2.03E-15	1.65E-14
TMEM116	-0.59292	2.07E-05	7.71E-05
SETD1A	-0.59249	3.06E-16	2.60E-15
DNAJB12	-0.59237	1.55E-17	1.43E-16
RPS6KA4	-0.59233	6.03E-14	4.48E-13
GLYCTK	-0.59207	3.10E-05	0.00011297
CRELD2	-0.59149	7.55E-24	9.42E-23
CDKN1A	-0.59148	1.50E-39	3.36E-38
GPD2	-0.59093	5.44E-19	5.40E-18
JRK	-0.59084	4.31E-19	4.31E-18
MTHFR	-0.59012	6.18E-11	3.73E-10
ZDHHC12	-0.59011	1.42E-13	1.03E-12
KLK2	-0.58993	2.23E-09	1.20E-08
ZNF789	-0.58938	9.74E-17	8.51E-16

RPP25	-0.58911	1.61E-05	6.06E-05
GGA1	-0.58861	6.33E-22	7.18E-21
PASK	-0.58843	5.61E-17	4.97E-16
PSMD9	-0.58827	0.002972	0.0081255
ZNF717	-0.58826	1.67E-09	9.06E-09
GRHL2	-0.58769	1.29E-33	2.35E-32
SKA1	-0.58753	1.67E-12	1.13E-11
DNAJC17	-0.58729	1.33E-05	5.06E-05
LINGO3	-0.58666	0.0097069	0.024027
LCLAT1	-0.58652	3.35E-10	1.92E-09
HSPA2	-0.58636	3.68E-16	3.12E-15
GNB1L	-0.58569	0.00049486	0.0015431
CDK7	-0.58533	1.78E-12	1.20E-11
ZNF653	-0.5853	0.00067487	0.0020568
RP11-793H13.11	-0.58523	0.0044167	0.011702
C12orf23	-0.58521	3.22E-16	2.73E-15
SLC16A8	-0.58507	0.00022885	0.00074674
PCYT2	-0.58497	4.91E-19	4.89E-18
QTRTD1	-0.5845	5.14E-17	4.56E-16
CCDC15	-0.58448	0.00099186	0.0029429
TEX264	-0.5844	4.87E-23	5.82E-22
CCND3	-0.58385	2.36E-13	1.69E-12
F2RL3	-0.58338	0.0013597	0.0039321
RP5-837J1.2	-0.58332	0.0012324	0.0035964
HSPH1	-0.58269	3.04E-24	3.85E-23
DOCK5	-0.58241	1.13E-17	1.04E-16
WWP2	-0.58219	3.14E-19	3.17E-18
KIAA0247	-0.58152	8.73E-14	6.43E-13
RAB17	-0.5815	3.29E-23	3.98E-22
PUS10	-0.58141	2.83E-07	1.28E-06
MAL2	-0.5813	6.22E-38	1.30E-36
CRLS1	-0.58127	3.57E-18	3.37E-17
RP11-305N23.1	-0.58049	0.00055692	0.0017193
RPS6KA3	-0.58014	7.05E-21	7.62E-20
INPPL1	-0.57994	4.85E-22	5.54E-21
MXD3	-0.57951	7.93E-05	0.00027509
AMD1	-0.57913	1.93E-42	4.76E-41
ZFP28	-0.5781	0.00015924	0.00053223
KRBA1	-0.57801	1.86E-08	9.32E-08
ZNF816	-0.57772	4.56E-05	0.00016306
FUZ	-0.57754	0.0014498	0.0041733
DGCR6	-0.5774	0.00017146	0.00057062
NT5DC3	-0.57697	1.10E-15	9.11E-15
ADPGK	-0.57678	1.61E-11	1.01E-10
RP11-497G19.1	-0.57666	0.015245	0.036116

C14orf164	-0.57657	0.014768	0.035058
RENBP	-0.57638	3.70E-05	0.00013333
GCSH	-0.57626	2.33E-07	1.06E-06
C3orf67	-0.57604	0.00014468	0.0004853
IL17RE	-0.57577	2.27E-10	1.31E-09
PVRL2	-0.57574	3.27E-09	1.74E-08
CTD-2240H23.2	-0.57573	0.0095439	0.023661
AC018755.16	-0.57543	0.021102	0.048309
DFFB	-0.57528	2.97E-09	1.59E-08
CENPI	-0.57487	8.16E-06	3.18E-05
ZNF675	-0.57482	4.68E-11	2.85E-10
CLIP2	-0.57434	2.23E-10	1.29E-09
SMG7-AS1	-0.57412	0.0081269	0.020453
GORASP1	-0.57389	4.82E-20	5.05E-19
RP11-545E17.3	-0.57363	0.0068726	0.017533
ACOT9	-0.57289	0.008011	0.020196
ZNF697	-0.57274	0.00039941	0.001261
TFG	-0.57234	3.30E-31	5.51E-30
SLC26A11	-0.57214	1.69E-07	7.76E-07
BNIP3	-0.57115	2.72E-21	3.00E-20
ELMO1	-0.57029	3.95E-06	1.59E-05
TARS	-0.56983	2.91E-27	4.22E-26
FXR2	-0.56969	5.29E-21	5.75E-20
CTC-428G20.3	-0.56957	0.0011223	0.0032956
HTATIP2	-0.56953	1.63E-06	6.78E-06
ASB8	-0.5689	5.61E-18	5.23E-17
SI	-0.56866	6.16E-12	4.00E-11
ASB1	-0.5686	1.28E-15	1.05E-14
ATF4	-0.56809	4.00E-29	6.21E-28
UBA5	-0.56755	4.39E-19	4.39E-18
SNHG8	-0.56705	6.55E-15	5.16E-14
PDE9A	-0.56703	3.70E-08	1.81E-07
NCKIPSD	-0.5667	2.05E-13	1.48E-12
MTFR1L	-0.56647	9.77E-08	4.60E-07
XKR8	-0.56623	0.001376	0.0039759
CDC45	-0.56612	5.68E-15	4.49E-14
PIK3IP1	-0.56609	2.44E-08	1.21E-07
SIRT5	-0.56589	1.98E-07	9.07E-07
TBC1D9B	-0.56579	3.13E-24	3.95E-23
BAIAP3	-0.56573	1.87E-06	7.72E-06
TMEM254	-0.56561	6.57E-16	5.50E-15
DQX1	-0.5656	0.0062344	0.016039
SRGAP2	-0.56526	2.73E-18	2.60E-17
ACP6	-0.56514	3.94E-08	1.92E-07
KDM4A	-0.56508	8.75E-17	7.67E-16

GPR160	-0.56498	3.26E-09	1.74E-08
UBE2V2	-0.56482	1.26E-21	1.40E-20
TRAF7	-0.56462	2.11E-26	2.96E-25
ZNF207	-0.56451	9.00E-25	1.17E-23
TRIM6	-0.56409	0.014237	0.033929
ADAM15	-0.56399	1.02E-32	1.80E-31
TFR2	-0.5636	5.28E-08	2.54E-07
USP31	-0.56359	3.24E-10	1.85E-09
RUNDC3B	-0.56338	1.15E-09	6.34E-09
NPLOC4	-0.56292	9.92E-29	1.53E-27
ZNF695	-0.56252	1.39E-05	5.30E-05
DENND5B	-0.56199	3.52E-08	1.73E-07
C1orf85	-0.56196	4.02E-16	3.40E-15
TLE6	-0.56154	0.00086724	0.0025973
DDB1	-0.56152	4.46E-24	5.61E-23
ATXN7L3B	-0.56144	1.87E-33	3.41E-32
AP1G1	-0.56131	2.81E-32	4.92E-31
MERTK	-0.56111	0.00052052	0.0016159
CCNK	-0.561	1.57E-20	1.68E-19
CDYL2	-0.56097	2.13E-08	1.06E-07
ATG4A	-0.56088	2.23E-10	1.29E-09
SKP2	-0.56087	9.52E-22	1.07E-20
KIF7	-0.56035	0.020748	0.047581
RANBP10	-0.56001	5.14E-17	4.57E-16
MRFAP1	-0.55976	1.96E-52	6.40E-51
RINT1	-0.55955	8.59E-13	5.94E-12
C12orf10	-0.55948	2.10E-07	9.56E-07
LPCAT4	-0.55874	1.02E-07	4.78E-07
FRS3	-0.55859	0.0006257	0.0019143
ARPC1B	-0.55849	9.95E-14	7.29E-13
PARP11	-0.55823	0.0031259	0.0085181
KATNAL1	-0.55803	6.89E-05	0.00024098
CROCCP4	-0.55793	0.0078146	0.019732
SOLH	-0.55753	2.06E-12	1.38E-11
GABRB3	-0.55729	9.46E-18	8.74E-17
MINOS1	-0.55728	1.29E-05	4.91E-05
ELAVL2	-0.55708	5.85E-05	0.00020685
CNP	-0.5568	4.59E-20	4.82E-19
CTC-504A5.1	-0.55629	0.0060341	0.01557
ABCD1	-0.55608	6.17E-05	0.00021752
HPS6	-0.55569	1.56E-09	8.51E-09
ZNF431	-0.55533	1.21E-08	6.16E-08
PDE4DIP	-0.55493	1.04E-22	1.23E-21
SEC22C	-0.5548	8.80E-23	1.04E-21
LCA5L	-0.55432	0.019493	0.045003

ATRIP	-0.55365	6.55E-09	3.41E-08
AC015849.15	-0.5532	0.0094376	0.023428
MAGEA10	-0.55306	0.00023933	0.00077872
KLF16	-0.553	1.73E-12	1.17E-11
AC093724.2	-0.55297	0.0015767	0.004518
TRAF3IP3	-0.55291	0.00095426	0.0028391
PIF1	-0.55206	0.00046652	0.0014605
SENP8	-0.55204	0.011073	0.027102
FBXW4	-0.55178	2.24E-14	1.71E-13
C9orf64	-0.55177	9.34E-15	7.31E-14
NSFL1C	-0.55174	1.58E-15	1.29E-14
ZNF724P	-0.55151	2.09E-06	8.59E-06
TTPAL	-0.55149	8.48E-11	5.06E-10
MPHOSPH9	-0.55114	9.50E-12	6.10E-11
RP5-1033H22.2	-0.55111	0.00040181	0.0012682
TPPP	-0.55105	0.00031495	0.0010086
FAM151B	-0.55104	4.29E-05	0.00015384
LPCAT1	-0.55076	8.41E-18	7.79E-17
BLOC1S2	-0.55035	6.56E-18	6.10E-17
BTC	-0.55006	1.85E-05	6.91E-05
TSPAN1	-0.54984	1.19E-07	5.54E-07
ABCF3	-0.54979	5.47E-20	5.71E-19
TBC1D16	-0.54909	3.30E-12	2.18E-11
TAPT1-AS1	-0.54839	0.0020643	0.0058006
SBNO1	-0.54826	1.71E-25	2.30E-24
RP11-114H21.2	-0.54748	0.016008	0.037708
RSBN1L-AS1	-0.547	2.60E-07	1.18E-06
AC092171.4	-0.54689	1.32E-09	7.20E-09
AC004166.7	-0.54687	2.84E-07	1.28E-06
ZNF829	-0.54594	1.50E-06	6.27E-06
FBXL6	-0.54565	6.41E-08	3.07E-07
PPARA	-0.54562	2.40E-09	1.29E-08
ATG4C	-0.54538	3.61E-08	1.76E-07
TNS4	-0.54521	0.010544	0.025915
ERAP1	-0.54509	3.32E-09	1.77E-08
ZNF586	-0.54478	4.42E-07	1.95E-06
ZNF286A	-0.54435	4.43E-06	1.78E-05
GDPD3	-0.54431	0.019518	0.045044
EAF1	-0.54407	3.25E-17	2.93E-16
GLIPR1	-0.54343	0.0031601	0.0086001
IL17RC	-0.5434	5.20E-15	4.12E-14
SLC25A22	-0.54295	5.88E-19	5.82E-18
ACOT2	-0.54291	1.81E-11	1.14E-10
HIST1H1C	-0.5429	0.0012374	0.0036085
DGKA	-0.54277	1.19E-07	5.56E-07

F5	-0.54225	0.0014033	0.0040508
UFSP1	-0.54115	0.00089874	0.0026851
CAV2	-0.54114	0.0010743	0.0031686
CNPY2	-0.54078	0.0068077	0.017384
LRRC8C	-0.54069	1.85E-05	6.92E-05
TRIM56	-0.5403	1.53E-08	7.75E-08
ENTPD3-AS1	-0.54013	0.00069482	0.0021142
ADD3	-0.54012	2.64E-15	2.13E-14
RASL11A	-0.53981	0.00038948	0.0012314
PVRL4	-0.53977	7.09E-06	2.78E-05
CASC3	-0.53943	6.75E-31	1.11E-29
TMEM30B	-0.53929	1.58E-12	1.07E-11
ERCC1	-0.53923	1.33E-08	6.75E-08
DPP10	-0.53922	0.013731	0.032878
MTHFD1	-0.53911	5.59E-29	8.65E-28
RBM27	-0.53872	6.22E-16	5.22E-15
MPZL3	-0.53867	3.43E-11	2.11E-10
TFEB	-0.53821	6.74E-05	0.00023619
ABP1	-0.53818	0.00016044	0.00053576
MED29	-0.53736	5.64E-20	5.88E-19
TBL1X	-0.53727	2.27E-11	1.41E-10
DNAJC14	-0.53702	2.62E-13	1.87E-12
CHEK1	-0.5369	2.12E-16	1.81E-15
KIAA1191	-0.53659	3.24E-23	3.92E-22
PDIA4	-0.53534	7.24E-41	1.70E-39
TMEM254-AS1	-0.53514	0.0053581	0.013964
SLFN5	-0.53477	0.00022284	0.00072828
ZNF860	-0.53474	7.35E-05	0.00025605
PRIM1	-0.53464	1.56E-07	7.20E-07
RAP1A	-0.53459	5.96E-12	3.87E-11
BTN2A2	-0.53381	3.26E-07	1.46E-06
BSPRY	-0.53332	1.17E-09	6.45E-09
CDC6	-0.53321	3.05E-18	2.89E-17
MAVS	-0.53299	8.87E-15	6.95E-14
PEG3	-0.53217	0.00025288	0.00082024
EXOC3	-0.53202	1.68E-08	8.46E-08
C19orf45	-0.5317	0.0040806	0.010862
ALS2	-0.53133	9.70E-10	5.38E-09
MROH1	-0.53122	4.15E-08	2.02E-07
CTC-338M12.3	-0.53098	0.00053431	0.0016562
PSMD12	-0.52981	5.22E-19	5.18E-18
GOLGA7	-0.5297	1.11E-17	1.02E-16
WARS	-0.52966	1.28E-22	1.50E-21
SPSB2	-0.52947	1.44E-08	7.29E-08
AK4P1	-0.52937	0.007109	0.01808

ST6GALNAC4	-0.5293	6.29E-05	0.00022135
DAXX	-0.52922	1.13E-17	1.04E-16
OCEL1	-0.52908	0.0010927	0.0032192
SYNGR2	-0.52897	4.32E-23	5.17E-22
PPP1R15A	-0.52896	4.62E-15	3.67E-14
AC007246.3	-0.52844	0.00035347	0.0011238
TNFSF15	-0.528	1.86E-06	7.69E-06
HIST1H2BK	-0.52796	2.93E-08	1.44E-07
CTDSPL	-0.52755	3.73E-32	6.48E-31
DCLRE1A	-0.52739	1.29E-12	8.78E-12
PHYHIPL	-0.52733	0.0010301	0.0030495
C9orf37	-0.52727	3.45E-06	1.40E-05
SRCAP	-0.52721	4.66E-15	3.70E-14
POLR2M	-0.52715	5.66E-13	3.97E-12
C1orf21	-0.52701	1.35E-08	6.87E-08
DOLK	-0.52673	1.17E-12	8.02E-12
PARGP1	-0.52619	5.00E-05	0.00017808
STIP1	-0.52586	7.52E-35	1.43E-33
SPATA24	-0.52572	0.0054721	0.014247
ZNF497	-0.52531	0.0021363	0.0059923
COA3	-0.525	1.35E-18	1.31E-17
ZBTB25	-0.52452	0.0010004	0.0029654
SIX2	-0.52449	2.75E-14	2.09E-13
LRRC61	-0.52397	4.11E-11	2.51E-10
THOP1	-0.52344	1.40E-13	1.02E-12
DBI	-0.52264	2.73E-10	1.57E-09
DGCR5	-0.52255	0.0014805	0.0042528
CORO6	-0.52225	0.00011886	0.00040332
DCUN1D5	-0.52221	2.72E-15	2.20E-14
TCEB3	-0.52206	1.21E-24	1.56E-23
ARHGAP31	-0.52199	2.92E-09	1.56E-08
NHSL1	-0.52186	6.10E-18	5.68E-17
ZFP90	-0.52168	0.016299	0.038285
CAPZA2	-0.52137	3.12E-23	3.77E-22
HSPBP1	-0.51998	7.82E-12	5.05E-11
FAM122C	-0.51997	0.014656	0.034836
ELFN1	-0.51997	0.00013267	0.00044754
CHAMP1	-0.51957	3.57E-14	2.70E-13
DPM3	-0.51886	8.92E-10	4.96E-09
ATP6V0D1	-0.51847	3.53E-14	2.67E-13
CRYZL1	-0.51844	5.02E-08	2.42E-07
MRPL18	-0.518	7.42E-17	6.53E-16
RHOBTB3	-0.51794	3.22E-08	1.58E-07
ARL13B	-0.51741	4.55E-07	2.01E-06
HK1	-0.51714	9.26E-20	9.55E-19

TINF2	-0.5171	1.83E-08	9.19E-08
TMEM62	-0.51697	2.66E-08	1.31E-07
RAB23	-0.51669	2.50E-06	1.02E-05
WDR6	-0.51642	2.92E-25	3.87E-24
CYB561A3	-0.51633	7.57E-09	3.93E-08
ARSG	-0.51597	0.0002049	0.00067444
GRTP1	-0.51541	7.00E-08	3.34E-07
SLC35A5	-0.5154	5.27E-13	3.70E-12
TMEM198B	-0.51501	7.59E-05	0.00026393
CSTF2	-0.51494	4.12E-10	2.34E-09
EFHC1	-0.51488	0.007605	0.019243
CTPS2	-0.51483	8.63E-08	4.08E-07
ZDHHC8P1	-0.51471	8.85E-07	3.79E-06
PAFAH2	-0.51448	6.43E-15	5.08E-14
RHOU	-0.51397	7.34E-32	1.26E-30
UBB	-0.51396	1.13E-13	8.27E-13
NDUFAF5	-0.5127	9.16E-07	3.92E-06
IPO11	-0.51261	2.24E-08	1.11E-07
MRPL15	-0.51221	4.24E-22	4.85E-21
CHFR	-0.51172	2.44E-07	1.10E-06
STAG3L1	-0.51164	0.017235	0.040258
CLGN	-0.51141	6.88E-08	3.29E-07
C3orf80	-0.51107	0.00067426	0.0020553
PSMA3	-0.51105	3.06E-17	2.77E-16
GRB10	-0.51076	2.11E-14	1.62E-13
OCRL	-0.51073	3.13E-19	3.16E-18
MBD1	-0.51055	1.32E-17	1.22E-16
ERCC2	-0.51051	2.17E-14	1.67E-13
FBXO2	-0.51041	0.016493	0.0387
RP1-152L7.5	-0.51035	7.48E-07	3.23E-06
CHCHD5	-0.51006	5.84E-08	2.81E-07
RASA4B	-0.51001	0.0033135	0.0089905
NEK1	-0.50996	5.36E-05	0.00019014
ZNF611	-0.5099	6.09E-05	0.00021501
ARFGEF2	-0.50957	9.75E-16	8.09E-15
VKORC1L1	-0.50924	6.42E-19	6.34E-18
CC2D1B	-0.509	7.64E-14	5.65E-13
CSMD1	-0.50878	2.45E-12	1.63E-11
C19orf70	-0.50875	1.72E-12	1.16E-11
IFT172	-0.5086	9.75E-07	4.16E-06
NCAPH2	-0.5085	3.67E-14	2.77E-13
ZBTB33	-0.50815	5.94E-11	3.59E-10
ZNF254	-0.50809	3.82E-11	2.34E-10
RAB12	-0.50805	1.56E-08	7.90E-08
WWOX	-0.50803	0.0026627	0.0073397

MMAB	-0.50803	1.04E-19	1.07E-18
MCM10	-0.50785	7.54E-14	5.58E-13
FAM32A	-0.50782	1.36E-15	1.11E-14
KCNMB4	-0.50759	2.89E-08	1.42E-07
NSMCE4A	-0.50687	1.65E-15	1.35E-14
PARG	-0.50668	3.33E-08	1.63E-07
ZNF606	-0.50664	0.00033181	0.0010595
RP11-18F14.2	-0.50662	1.64E-10	9.57E-10
CCNH	-0.50661	1.02E-12	7.04E-12
TMEM129	-0.50658	3.37E-12	2.23E-11
UNC119B	-0.50641	3.80E-30	6.13E-29
TSPYL4	-0.50634	3.40E-19	3.43E-18
FAM149A	-0.50604	0.00014059	0.0004725
SEC14L1P1	-0.50597	0.0057822	0.014987
FAM73B	-0.50596	6.06E-11	3.66E-10
SLC25A32	-0.50577	3.97E-11	2.43E-10
SOCS7	-0.5057	8.93E-07	3.82E-06
HAX1	-0.50548	1.91E-31	3.22E-30
ADIPOR1	-0.50527	9.30E-31	1.52E-29
FBXL2	-0.50523	0.00021629	0.00070887
FANCG	-0.50516	1.22E-13	8.88E-13
HELQ	-0.50512	8.89E-05	0.00030655
HBE1	-0.5048	4.44E-07	1.96E-06
FANCC	-0.5046	4.24E-10	2.41E-09
CDK10	-0.50425	1.54E-12	1.04E-11
C1orf233	-0.50417	1.58E-05	5.98E-05
ATP13A1	-0.50415	1.02E-14	7.94E-14
THAP4	-0.50374	4.10E-15	3.27E-14
SRGAP2B	-0.50361	1.53E-05	5.81E-05
CEP290	-0.50321	2.34E-09	1.26E-08
CACNA2D1	-0.50299	3.65E-22	4.18E-21
RING1	-0.50286	2.72E-12	1.80E-11
GTF2H2C	-0.50261	4.54E-06	1.82E-05
ZSWIM1	-0.50235	6.39E-07	2.77E-06
HERPUD1	-0.50208	3.07E-20	3.24E-19
PIGS	-0.50199	4.27E-19	4.28E-18
SPRYD7	-0.50196	1.12E-07	5.24E-07
APC2	-0.50182	5.91E-06	2.34E-05
MCEE	-0.50165	0.00051064	0.0015876
ABCC10	-0.50124	2.86E-05	0.00010455
ADCK5	-0.50109	2.42E-05	8.91E-05
RP11-474P12.5	-0.50108	0.014659	0.03484
UBE4A	-0.50103	2.59E-14	1.97E-13
AC005003.1	-0.50098	0.00019928	0.00065727
SNW1	-0.50043	2.60E-12	1.73E-11

KCNIP4		-0.50041	0.01734	0.040474
CSE1L		0.50012	3.11E-23	3.77E-22
RPL15P3		0.50034	0.0003806	0.0012053
CCNL2		0.50041	1.42E-12	9.70E-12
JAK1		0.50057	0.0012853	0.0037356
C16orf72		0.50059	1.72E-13	1.24E-12
TMEM121		0.5006	2.29E-05	8.47E-05
RAP1B		0.50072	1.29E-19	1.32E-18
GLTPD1		0.50079	3.56E-11	2.19E-10
VAMP2		0.50082	5.11E-17	4.55E-16
DIXDC1		0.50132	3.48E-10	1.99E-09
DDX39B		0.50135	4.80E-13	3.38E-12
SLIT2		0.50154	0.005664	0.014703
LTBP3		0.50158	1.78E-10	1.04E-09
ZFAND6		0.50158	4.69E-15	3.73E-14
DYNC2H1		0.50164	6.42E-09	3.35E-08
DSTYK		0.50168	2.05E-16	1.76E-15
MOAP1		0.50171	4.20E-19	4.21E-18
RP11-475C16.1		0.50189	0.00042908	0.0013494
CCDC137		0.50194	1.46E-17	1.34E-16
FCHO2		0.50201	2.25E-14	1.72E-13
ASH1L		0.50202	2.88E-15	2.32E-14
RSF1		0.50229	1.52E-12	1.04E-11
NREP		0.50247	4.59E-05	0.00016392
	9-Sep	0.50252	3.30E-19	3.32E-18
CTGLF12P		0.50316	0.012496	0.030232
ZNF362		0.50326	1.05E-05	4.05E-05
RBPJ		0.50343	5.61E-24	7.03E-23
IGBP1		0.5035	9.47E-10	5.26E-09
OTUD3		0.50353	1.45E-10	8.53E-10
POLE3		0.50364	5.08E-21	5.53E-20
SCAMP5		0.50387	1.12E-10	6.59E-10
ATPBD4		0.50393	1.62E-09	8.82E-09
RNF216P1		0.50394	1.78E-06	7.37E-06
NBPF14		0.5043	8.43E-07	3.61E-06
COMMD5		0.50447	1.05E-06	4.44E-06
RP11-51O6.1		0.50451	0.014752	0.035028
TUBBP5		0.5047	1.97E-05	7.37E-05
TRIOBP		0.50487	1.32E-06	5.55E-06
PLXNC1		0.50502	0.017961	0.04181
KIF2C		0.50549	2.24E-14	1.71E-13
PDE12		0.5057	2.33E-26	3.25E-25
TOMM20		0.5057	6.56E-32	1.13E-30
BCL3		0.50576	3.96E-06	1.60E-05
FNBP1L		0.50582	7.41E-23	8.76E-22

TYW1B	0.50606	1.18E-06	4.98E-06
ADAM9	0.50639	9.62E-27	1.36E-25
PVR	0.50641	2.23E-22	2.57E-21
PLEKHO1	0.50659	1.29E-07	5.97E-07
RABL2A	0.50698	0.00010877	0.00037135
MAN2A2	0.50742	1.16E-12	7.96E-12
NEFL	0.50774	0.0063488	0.016309
R3HCC1L	0.50798	4.76E-08	2.30E-07
LITAF	0.50809	0.0070734	0.018003
PTBP2	0.50833	2.07E-11	1.29E-10
PLEC	0.50839	0.00042244	0.0013301
EEF1A1P6	0.50847	8.53E-07	3.65E-06
FTSJD1	0.50848	6.58E-12	4.27E-11
STC2	0.5085	1.10E-10	6.51E-10
COMMD10	0.50851	9.80E-10	5.43E-09
PTMAP5	0.50858	2.34E-06	9.61E-06
SHOC2	0.50861	7.07E-15	5.56E-14
KBTBD11	0.50862	8.51E-21	9.18E-20
DCTN6	0.50876	6.61E-08	3.16E-07
CCM2	0.50889	3.76E-14	2.84E-13
TMEM8B	0.50905	5.70E-10	3.21E-09
ABI2	0.50925	1.49E-21	1.66E-20
PILRB	0.50945	8.69E-11	5.18E-10
RABL2B	0.50947	2.04E-11	1.28E-10
TTC13	0.50974	4.90E-12	3.20E-11
CDC42BPA	0.50998	8.23E-24	1.03E-22
STAG2	0.51003	1.70E-25	2.29E-24
TET2	0.51035	4.18E-13	2.95E-12
CXorf57	0.51038	8.95E-06	3.47E-05
VPS13D	0.51046	1.70E-12	1.15E-11
EXT1	0.51071	1.41E-09	7.69E-09
TANC2	0.51074	0.00012759	0.0004312
GPRC5C	0.51122	3.08E-07	1.38E-06
KLHL11	0.51133	3.29E-08	1.61E-07
GOLT1B	0.51151	5.94E-15	4.69E-14
NPTX1	0.51161	7.67E-05	0.00026651
MYO1E	0.51171	2.95E-13	2.10E-12
LRPPRC	0.51205	7.55E-27	1.07E-25
MT-ND3	0.51208	0.0039081	0.010447
AATK	0.51208	0.00030934	0.00099207
ZCCHC10	0.5121	7.46E-08	3.55E-07
KALRN	0.51211	0.010078	0.024866
RAB40C	0.51263	3.85E-12	2.53E-11
HYLS1	0.51274	2.98E-07	1.34E-06
CENPB	0.51283	1.21E-20	1.29E-19

MT-CYB	0.51285	1.60E-37	3.30E-36
SPTBN2	0.5129	7.05E-14	5.22E-13
DPY19L4	0.51315	5.28E-17	4.69E-16
GATA6	0.51334	0.00052676	0.0016345
ANKRD40	0.51363	1.99E-20	2.12E-19
GTF2B	0.5137	3.31E-10	1.89E-09
NHP2L1	0.51372	5.94E-30	9.53E-29
SLC2A8	0.51378	1.96E-09	1.06E-08
GLS	0.51382	4.98E-22	5.68E-21
KCTD15	0.51397	1.67E-14	1.29E-13
HOXC9	0.51399	0.0032758	0.0088986
TMEM56	0.51402	9.51E-08	4.48E-07
GRIP1	0.51438	0.0024075	0.0066997
SPSB1	0.51449	0.00010412	0.00035616
NUB1	0.51473	3.27E-18	3.10E-17
ZNF740	0.51477	2.29E-25	3.05E-24
MT-ND6	0.51495	0.0020553	0.0057769
TEX10	0.51525	6.44E-16	5.40E-15
SUV420H1	0.51525	2.30E-19	2.34E-18
FAM72C	0.5153	0.0020605	0.0057908
CXXC5	0.51531	0.00031579	0.0010112
AK1	0.51541	9.47E-05	0.00032545
TTLL4	0.51551	8.24E-13	5.70E-12
RPL39P3	0.51572	0.011101	0.027154
ALG8	0.5158	8.22E-17	7.22E-16
GAR1	0.51594	6.70E-15	5.28E-14
NUF2	0.51597	1.20E-18	1.17E-17
CENPJ	0.51627	7.95E-09	4.11E-08
NDUFB2	0.51633	4.94E-17	4.40E-16
RAB15	0.51634	6.95E-06	2.73E-05
DSG2	0.51668	4.88E-27	6.98E-26
PTCH1	0.51684	2.38E-13	1.70E-12
RGPD8	0.51694	1.18E-05	4.52E-05
AKAP10	0.51757	1.75E-12	1.18E-11
UBTD2	0.51763	1.18E-11	7.53E-11
LRRC8D	0.5177	1.24E-13	9.00E-13
CRAMP1L	0.51774	8.73E-09	4.50E-08
BTG2	0.51778	3.94E-23	4.74E-22
BAI1	0.518	5.53E-06	2.20E-05
CNOT8	0.51815	1.93E-14	1.49E-13
ATP6V1C1	0.51828	6.05E-23	7.17E-22
BNIP2	0.51845	2.32E-11	1.44E-10
PMP22	0.51871	0.0019436	0.005487
RPL15	0.51889	2.15E-48	6.34E-47
OXSR1	0.51904	3.94E-29	6.11E-28

NRP1	0.51934	8.28E-05	0.00028682
CYTH2	0.51934	1.78E-14	1.37E-13
ISYNA1	0.51947	1.05E-16	9.17E-16
PPP2R2A	0.51951	1.33E-29	2.12E-28
SENP6	0.51964	3.25E-27	4.70E-26
SPESP1	0.52005	2.50E-06	1.02E-05
ZBED5	0.52009	5.68E-16	4.77E-15
TNFAIP1	0.5201	1.68E-14	1.30E-13
PDE8B	0.52047	0.00016299	0.00054374
PCNP	0.52062	2.13E-21	2.36E-20
CUX1	0.52066	1.36E-17	1.25E-16
P2RY11	0.52108	0.0039893	0.010639
UBE2D4	0.52122	0.00059767	0.001834
EIF4G2	0.52132	8.97E-32	1.53E-30
LCA5	0.52147	0.0034816	0.0094085
SRF	0.5219	1.51E-20	1.61E-19
NEK7	0.52211	2.23E-28	3.38E-27
FAM193A	0.52229	5.05E-14	3.78E-13
F2RL1	0.52287	3.96E-16	3.35E-15
GABRA1	0.52289	1.93E-20	2.05E-19
NFIA	0.52298	4.56E-11	2.78E-10
PTPN2	0.52303	7.79E-11	4.67E-10
SLC4A8	0.52305	0.0019935	0.0056137
UBE2K	0.52333	1.59E-23	1.94E-22
CCDC40	0.52356	0.0010694	0.0031564
STK24	0.52376	1.14E-22	1.34E-21
UBN2	0.52382	2.09E-16	1.80E-15
SH3GLB1	0.5239	6.53E-17	5.77E-16
SAT1	0.52409	1.98E-07	9.04E-07
SREBF2	0.52412	1.16E-21	1.30E-20
RFWD3	0.52437	3.00E-23	3.63E-22
MEMO1	0.5245	0.014438	0.034377
ZBTB49	0.52466	0.003904	0.010438
CDK9	0.52466	3.10E-12	2.05E-11
PHACTR2	0.52466	1.67E-19	1.70E-18
SPPL2B	0.52519	2.59E-12	1.72E-11
GRIN1	0.52553	3.59E-11	2.21E-10
AC010091.1	0.52554	0.00059524	0.0018273
RNA28S5	0.5256	0.00011295	0.00038439
KRIT1	0.52567	1.23E-14	9.55E-14
NOTCH1	0.52575	0.00036586	0.0011608
NELL2	0.52576	1.26E-15	1.04E-14
AC005943.5	0.52612	4.23E-07	1.87E-06
SAV1	0.52662	7.26E-20	7.52E-19
RIMS2	0.52696	3.72E-11	2.29E-10

HIVEP2		0.52697	3.53E-12	2.32E-11
CACNB1		0.52704	0.0010647	0.0031433
STOM		0.52757	4.04E-29	6.27E-28
REXO2		0.52758	1.06E-10	6.25E-10
THOC7		0.5277	6.23E-13	4.36E-12
BTN2A1		0.52786	1.68E-10	9.81E-10
PPP2R2D		0.52818	1.73E-12	1.17E-11
TMX3		0.52849	5.02E-15	3.98E-14
LAMTOR1		0.52876	1.99E-22	2.31E-21
HNRNPU		0.52907	4.69E-33	8.41E-32
UPF1		0.52988	1.35E-18	1.31E-17
ADORA2B		0.53092	0.00023814	0.00077536
NMNAT2		0.53097	8.28E-15	6.49E-14
ARID5B		0.53118	9.73E-20	1.00E-18
KCTD21		0.53127	0.00013225	0.00044628
CKAP2		0.53131	5.14E-19	5.11E-18
METTL21D		0.53172	2.29E-05	8.46E-05
ADAMTS1		0.53173	9.63E-16	8.00E-15
ELL2		0.5321	1.38E-31	2.34E-30
RNF146		0.53223	2.33E-15	1.89E-14
LAT2		0.53233	6.21E-13	4.34E-12
SIN3A		0.53242	1.31E-21	1.46E-20
RP11-701H24.7		0.53262	0.012827	0.030936
TTC6		0.5327	1.93E-09	1.04E-08
PCNT		0.53307	2.26E-12	1.52E-11
ZC3H12C		0.53308	1.17E-09	6.45E-09
	8-Mar	0.53322	1.85E-14	1.42E-13
AC006465.3		0.53323	2.59E-08	1.28E-07
NUP62CL		0.53347	9.64E-08	4.54E-07
RB1		0.53372	1.70E-18	1.64E-17
TIMP1		0.534	1.53E-35	2.99E-34
VPS33A		0.53419	9.47E-11	5.63E-10
MLL3		0.53421	2.65E-13	1.89E-12
DNAJB4		0.53424	2.71E-07	1.23E-06
RFWD2		0.53443	2.31E-20	2.44E-19
EFEMP1		0.53466	1.60E-10	9.37E-10
POLR3B		0.5347	2.95E-15	2.37E-14
BTBD1		0.53489	1.78E-29	2.80E-28
GPRC5D		0.5351	0.013984	0.033399
CRISPLD2		0.53514	0.00066052	0.0020146
HSDL1		0.53549	1.04E-14	8.10E-14
TMX1		0.53553	6.43E-16	5.39E-15
NUP50		0.53565	3.82E-29	5.93E-28
STK35		0.53588	2.12E-26	2.97E-25
KCTD20		0.53599	1.09E-24	1.41E-23

YBX1P1	0.53673	0.012932	0.031148
ZNF124	0.53734	9.92E-13	6.83E-12
SLC38A4	0.53751	6.11E-12	3.97E-11
TBC1D12	0.53803	1.14E-13	8.29E-13
PABPC5	0.53874	0.0080124	0.020197
FLJ22184	0.53893	0.0051363	0.013429
UBE2G1	0.53901	4.83E-27	6.92E-26
KRBOX4	0.53944	2.38E-07	1.08E-06
MAP4K3	0.53944	2.69E-17	2.44E-16
PITPNM2	0.53962	2.57E-10	1.48E-09
FAXC	0.53968	4.82E-05	0.00017171
CCNI	0.53968	1.71E-29	2.70E-28
GEN1	0.53969	9.41E-13	6.49E-12
WDR82	0.53976	9.63E-36	1.90E-34
ARID4B	0.53989	3.81E-22	4.37E-21
NBPF10	0.53994	1.11E-09	6.10E-09
SLC7A2	0.54053	2.81E-25	3.74E-24
MLL	0.54054	1.61E-12	1.09E-11
LYPD6	0.54078	0.0090732	0.022596
HPN	0.54132	1.30E-15	1.07E-14
XXbac-B135H6.15	0.54148	0.00392	0.010472
C4orf29	0.54148	1.66E-11	1.05E-10
HES2	0.54156	0.00012798	0.00043236
ESYT1	0.54156	1.49E-31	2.53E-30
RP11-20O24.4	0.54178	0.011615	0.028285
KHNYN	0.54214	1.08E-09	5.95E-09
RBPMS	0.54265	9.52E-06	3.68E-05
TRIP13	0.54272	3.20E-19	3.23E-18
SMAD3	0.54311	3.79E-11	2.33E-10
FKBP7	0.54319	5.50E-05	0.0001949
KDM2A	0.54344	3.40E-29	5.30E-28
CAMK2B	0.54366	1.46E-05	5.52E-05
ANKH	0.54385	1.10E-20	1.18E-19
RBBP9	0.54437	2.73E-17	2.48E-16
CEP85	0.54462	1.07E-11	6.86E-11
GOLGA2B	0.54476	1.66E-06	6.92E-06
TIGD3	0.5448	0.0023763	0.006619
SORT1	0.54518	1.55E-25	2.10E-24
STXBP5L	0.54521	5.84E-07	2.55E-06
SNHG6	0.54536	8.72E-19	8.58E-18
AL627309.1	0.54543	0.00078983	0.0023817
LRIG1	0.54596	6.84E-07	2.96E-06
RBM12	0.54596	4.91E-29	7.60E-28
DNAJC6	0.54649	1.53E-10	8.98E-10
ZNF503-AS2	0.54655	4.18E-12	2.74E-11

IGFBP2	0.54697	3.05E-16	2.59E-15
LINC00324	0.54705	0.00069398	0.002112
ZFPM1	0.54709	1.08E-07	5.07E-07
PTPN21	0.54721	2.49E-05	9.15E-05
MAPK1	0.54736	1.77E-40	4.10E-39
ATL1	0.54739	1.63E-05	6.14E-05
SNHG1	0.54745	5.46E-15	4.32E-14
STK4	0.54785	3.18E-17	2.87E-16
KAZN	0.54786	0.0002532	0.00082102
ZNF549	0.548	1.35E-06	5.68E-06
MBIP	0.54814	3.00E-09	1.60E-08
CD46	0.54829	1.72E-26	2.41E-25
POC1A	0.54844	2.99E-14	2.27E-13
KLF15	0.54866	8.89E-13	6.14E-12
STMN1	0.54877	2.53E-25	3.36E-24
TMEM185A	0.5489	0.0018123	0.0051339
TNFAIP8L1	0.54898	7.81E-07	3.36E-06
PCSK1N	0.54903	0.0024581	0.0068258
TERT	0.54924	0.00016444	0.00054796
NCBP2	0.54936	1.58E-26	2.22E-25
DENND2D	0.54943	3.72E-09	1.98E-08
RICTOR	0.54977	4.82E-13	3.39E-12
TMEM45B	0.54983	1.70E-08	8.58E-08
AHCTF1	0.54998	4.39E-34	8.10E-33
SOS1	0.5506	1.05E-16	9.16E-16
ARHGEF10	0.55064	2.49E-15	2.01E-14
ZFHX4	0.55104	6.46E-05	0.00022685
FAM89A	0.55115	8.87E-06	3.44E-05
DLGAP4	0.55135	6.05E-15	4.78E-14
RP11-742N3.1	0.55142	1.80E-06	7.46E-06
SIGMAR1	0.55223	3.85E-36	7.67E-35
PELI1	0.55228	6.62E-09	3.45E-08
FDXR	0.55238	5.02E-23	5.98E-22
AKAP5	0.55253	0.00046003	0.0014406
FAM3C	0.55309	5.28E-28	7.85E-27
SMAGP	0.5535	4.33E-10	2.46E-09
MED21	0.55358	1.37E-11	8.67E-11
KBTBD8	0.55371	7.66E-06	2.99E-05
CTD-2119F7.2	0.55414	0.00011302	0.0003845
PLXDC2	0.5546	0.0017451	0.0049567
SLC9B2	0.55466	1.50E-13	1.09E-12
RASA1	0.55487	3.50E-18	3.31E-17
JMJD1C	0.55527	8.86E-19	8.70E-18
CAPZA1	0.55556	2.03E-24	2.59E-23
CNST	0.55601	1.60E-21	1.78E-20

PPIC	0.5563	5.44E-11	3.30E-10
KCND2	0.55641	0.0082608	0.020758
ZBED1	0.55658	2.50E-16	2.14E-15
CCPG1	0.55667	0.0021283	0.0059732
NAP1L4	0.55671	2.46E-39	5.48E-38
ORAI3	0.55672	6.62E-06	2.61E-05
SS18L1	0.5568	8.58E-13	5.94E-12
OXR1	0.55715	7.46E-20	7.73E-19
ACAT1	0.55739	1.30E-24	1.67E-23
CALCOCO1	0.55798	4.87E-21	5.31E-20
AASDHPPT	0.55812	1.05E-14	8.22E-14
SYNRG	0.55815	1.74E-16	1.50E-15
LURAP1L	0.55818	0.015506	0.036645
APH1B	0.55823	0.00013082	0.00044173
INPP4A	0.55855	2.24E-14	1.72E-13
DDOST	0.55858	6.80E-38	1.42E-36
PTPRN2	0.55871	0.0014815	0.0042552
BCORL1	0.55917	5.50E-17	4.88E-16
CTD-2006C1.2	0.55923	2.10E-06	8.64E-06
ZSWIM3	0.55956	5.88E-05	0.00020788
TGIF2	0.55982	1.06E-17	9.78E-17
STAT1	0.55989	1.53E-20	1.63E-19
FAM199X	0.55994	4.74E-23	5.67E-22
RRAS2	0.56004	6.62E-18	6.16E-17
PDGFC	0.5607	6.64E-05	0.00023278
TMSB4X	0.5607	1.40E-09	7.63E-09
PIGU	0.56084	1.14E-16	9.89E-16
NBEA	0.56131	6.79E-12	4.40E-11
ZNF670	0.56156	8.28E-09	4.28E-08
DPY19L1	0.5616	1.00E-08	5.15E-08
ITM2C	0.56169	1.10E-32	1.95E-31
KDM6B	0.56179	7.47E-19	7.37E-18
PSTPIP2	0.56193	1.05E-11	6.74E-11
ARPP19	0.56193	2.99E-38	6.34E-37
SYDE2	0.56236	1.12E-08	5.73E-08
SFXN1	0.56236	9.51E-31	1.56E-29
PRR14L	0.5625	1.56E-21	1.74E-20
BTBD3	0.56281	4.84E-17	4.31E-16
SLC29A3	0.56285	8.26E-07	3.55E-06
CTIF	0.5638	5.87E-07	2.56E-06
RP11-151N17.2	0.56399	1.25E-06	5.24E-06
CDV3	0.56399	2.25E-35	4.36E-34
HECTD4	0.56405	2.35E-10	1.36E-09
PCDHB7	0.56463	0.0043283	0.011482
NR1D2	0.56468	4.49E-17	4.01E-16

PDE5A	0.5647	1.38E-08	6.98E-08
CEP135	0.565	9.20E-13	6.35E-12
PWWP2B	0.5651	1.44E-09	7.83E-09
ARHGAP1	0.5651	1.79E-24	2.29E-23
INPP1	0.56516	0.00015769	0.0005274
FAM69A	0.56518	0.00010154	0.00034773
C17orf58	0.56532	1.45E-06	6.07E-06
GIPC1	0.56557	1.17E-25	1.59E-24
SLC47A1	0.56568	1.24E-14	9.65E-14
WNK1	0.56589	1.59E-24	2.04E-23
MPP6	0.56598	6.91E-13	4.81E-12
PRSS12	0.56601	0.00077801	0.0023488
MIER1	0.56638	2.08E-13	1.50E-12
CALD1	0.56656	3.67E-36	7.31E-35
FOXK1	0.56679	3.02E-22	3.47E-21
IFNAR2	0.56722	9.25E-08	4.36E-07
SLC25A6	0.56725	5.30E-28	7.88E-27
ATG9A	0.56726	3.98E-18	3.75E-17
TRIP10	0.5673	1.91E-05	7.15E-05
CRIPT	0.56738	5.51E-07	2.41E-06
PAPD5	0.56753	5.56E-19	5.51E-18
NOV	0.56765	1.76E-11	1.11E-10
APBB3	0.56827	5.99E-07	2.61E-06
ZC4H2	0.5683	2.11E-14	1.62E-13
DNAJC12	0.56835	2.09E-08	1.04E-07
MT-ND5	0.56838	0.0001209	0.00040983
TMEM64	0.56838	1.54E-24	1.97E-23
PRICKLE3	0.56861	1.58E-12	1.07E-11
FAM3C2	0.56866	3.30E-05	0.00011983
PAPD7	0.56875	6.06E-17	5.36E-16
PALMD	0.56914	0.0012924	0.0037524
SLC41A2	0.56946	8.84E-10	4.92E-09
RBM14	0.56979	7.23E-24	9.03E-23
PINK1	0.57016	1.68E-08	8.47E-08
FANCM	0.57019	4.29E-09	2.27E-08
PSD3	0.57033	1.91E-18	1.83E-17
GATS	0.5704	8.81E-05	0.0003041
RP11-35G9.3	0.57104	0.001817	0.0051453
AFF4	0.57107	2.82E-25	3.74E-24
RBM33	0.57126	2.37E-26	3.31E-25
MYH7B	0.5713	0.010874	0.026658
FERMT2	0.57147	1.25E-11	7.95E-11
PXDC1	0.57172	0.00029619	0.00095269
PIK3C2B	0.57176	9.05E-17	7.92E-16
TMEM167A	0.57177	1.22E-19	1.25E-18

RP5-1148A21.3	0.57219	0.020214	0.046487
DNMT3B	0.57232	7.52E-09	3.90E-08
RPL18AP3	0.57261	7.64E-05	0.00026554
ZFYVE20	0.57261	1.26E-27	1.85E-26
ZBTB44	0.57274	7.77E-22	8.77E-21
TRIB1	0.57278	6.13E-39	1.34E-37
TAOK1	0.57317	2.94E-23	3.57E-22
SPIN1	0.5732	1.12E-32	1.98E-31
LINC00265	0.57356	4.71E-06	1.88E-05
BLM	0.57368	3.95E-11	2.42E-10
DDHD2	0.57376	6.66E-32	1.15E-30
WIZ	0.57432	1.86E-16	1.60E-15
SNX33	0.57433	0.00032859	0.00105
GID4	0.57433	1.37E-11	8.66E-11
CD2AP	0.57489	1.10E-25	1.49E-24
CD151	0.57557	3.76E-20	3.96E-19
SMCR7L	0.57579	2.08E-27	3.03E-26
ERLIN2	0.57596	7.98E-38	1.66E-36
PGRMC2	0.57629	1.29E-18	1.26E-17
CCDC88A	0.57656	3.46E-08	1.70E-07
GABRA3	0.57665	3.48E-05	0.00012594
ZDHHC2	0.57672	3.40E-28	5.10E-27
RPL7AP6	0.57702	0.00016376	0.00054594
OGFR	0.57727	1.17E-18	1.13E-17
TEAD1	0.57734	1.04E-18	1.02E-17
RP11-24B19.3	0.57758	0.00023918	0.00077836
61E3.4	0.57775	0.00059374	0.0018235
RECK	0.57778	4.72E-05	0.00016826
RP11-334C17.5	0.5781	0.0014186	0.0040909
CHMP6	0.57813	9.03E-09	4.65E-08
DBT	0.57828	7.05E-24	8.82E-23
STIL	0.57834	1.41E-15	1.16E-14
MAPKAPK3	0.57864	2.13E-25	2.84E-24
SH3BGRL	0.57873	2.98E-17	2.69E-16
ANKRA2	0.5789	4.54E-16	3.84E-15
NAE1	0.57918	1.05E-24	1.36E-23
RBMXL1	0.57939	4.81E-14	3.61E-13
CMTM6	0.57944	2.79E-23	3.39E-22
CPOX	0.58025	3.06E-25	4.05E-24
PAQR3	0.58036	6.34E-11	3.82E-10
IFRD1	0.58037	8.41E-26	1.15E-24
KAT6B	0.58052	8.54E-16	7.11E-15
PARP16	0.58065	3.15E-11	1.95E-10
IL18BP	0.58128	0.00062511	0.0019128
ZDHHC23	0.58133	2.91E-21	3.20E-20

CDK2AP1	0.58141	3.99E-21	4.36E-20
FBXO17	0.58149	1.85E-08	9.28E-08
MIS18BP1	0.58177	1.05E-17	9.69E-17
AASS	0.58255	1.51E-11	9.55E-11
MINPP1	0.58261	2.03E-22	2.35E-21
IGSF9	0.58264	6.11E-12	3.97E-11
TCF12	0.58284	7.32E-25	9.55E-24
CCNG1	0.58309	6.80E-20	7.06E-19
GGA2	0.58352	9.11E-32	1.55E-30
SLC43A2	0.58366	4.89E-17	4.36E-16
CARS2	0.58377	8.52E-28	1.26E-26
NT5C2	0.58431	1.83E-22	2.12E-21
LYPLA1	0.58484	1.08E-24	1.40E-23
BAI3	0.58486	0.011973	0.029084
GATA3	0.58502	1.77E-12	1.19E-11
ZNF112	0.58597	0.00011204	0.00038168
H3F3AP6	0.58601	4.96E-07	2.18E-06
SCOC	0.58601	8.32E-23	9.81E-22
TBC1D9	0.58657	6.66E-19	6.58E-18
CTNNB1	0.5867	1.71E-49	5.22E-48
FAM19A2	0.58692	1.95E-09	1.06E-08
PKD1	0.58696	0.00093895	0.0027956
PIAS2	0.58696	5.26E-23	6.26E-22
ADRBK2	0.587	2.09E-12	1.40E-11
DR1	0.58702	7.69E-33	1.37E-31
MORF4L1P1	0.58781	1.32E-08	6.70E-08
NCOA3	0.58781	1.74E-21	1.93E-20
DCP1B	0.58785	4.83E-20	5.06E-19
ICK	0.58794	8.14E-27	1.15E-25
LBR	0.58804	1.04E-29	1.66E-28
MOB1B	0.5884	1.69E-23	2.06E-22
SGOL1	0.58864	3.87E-08	1.89E-07
MMP24	0.58864	2.96E-08	1.45E-07
TMEM164	0.58881	1.65E-22	1.92E-21
SNX2	0.58896	3.75E-23	4.51E-22
DDX5	0.58921	3.59E-41	8.54E-40
PLEKHB1	0.58958	7.47E-28	1.11E-26
RCE1	0.58963	2.31E-11	1.44E-10
BZW2	0.59009	4.82E-26	6.66E-25
BUB3	0.59061	6.89E-41	1.62E-39
CLCN7	0.59108	1.08E-13	7.88E-13
GABARAPL1	0.59113	1.08E-07	5.07E-07
ATP6AP1	0.59113	2.44E-33	4.41E-32
IGDCC4	0.59116	0.00067602	0.00206
UPF3B	0.59128	3.35E-15	2.69E-14

ALG9	0.59145	2.40E-08	1.19E-07
EPS8L1	0.59171	8.18E-13	5.67E-12
SH3BP5	0.59219	0.00050218	0.0015636
AIFM3	0.5924	9.05E-08	4.27E-07
SNRK	0.59253	3.60E-14	2.72E-13
ISPD	0.59276	6.51E-05	0.00022851
BID	0.59286	1.45E-06	6.08E-06
ACVR2A	0.59289	2.28E-07	1.04E-06
PAN3	0.59321	5.55E-22	6.32E-21
B4GALNT4	0.59331	7.89E-12	5.09E-11
TMED3	0.59373	4.81E-39	1.06E-37
SELT	0.5938	3.66E-28	5.48E-27
DUSP7	0.59412	1.14E-21	1.28E-20
KIF1B	0.59475	7.73E-25	1.01E-23
GREB1	0.59476	1.60E-07	7.36E-07
CELF5	0.59481	0.00016607	0.00055321
YWHAZ	0.59483	2.90E-31	4.86E-30
LPAR2	0.59507	0.000907	0.0027078
LONRF3	0.59533	2.07E-07	9.43E-07
BAK1	0.59553	1.97E-22	2.28E-21
GRIN2D	0.5958	1.68E-12	1.14E-11
MED12L	0.59589	0.00048237	0.0015067
HTRA1	0.59591	5.92E-10	3.34E-09
RP11-53O19.1	0.59627	0.012293	0.029799
SEMA3C	0.59631	2.61E-31	4.39E-30
GFRA1	0.59648	1.05E-05	4.05E-05
PITX1	0.59652	1.26E-13	9.21E-13
CD83	0.59678	0.015271	0.036158
ERBB2IP	0.59691	4.21E-28	6.29E-27
SRM	0.59699	2.77E-24	3.52E-23
AHDC1	0.59702	5.30E-09	2.79E-08
PTDSS1	0.59726	2.73E-35	5.26E-34
GLDN	0.59773	0.00036738	0.0011652
ITGA9	0.59797	0.00019909	0.00065677
EEA1	0.59798	7.78E-28	1.15E-26
HADHA	0.59807	1.90E-42	4.69E-41
GCC2	0.5983	2.53E-16	2.16E-15
FGFR1OP2	0.59853	2.15E-15	1.75E-14
TADA2B	0.59877	1.90E-15	1.55E-14
ESCO1	0.59893	1.87E-14	1.44E-13
ZFAS1	0.59904	2.93E-18	2.78E-17
UBAP1	0.59979	2.56E-29	4.01E-28
IPO5	0.60001	8.45E-37	1.72E-35
SGK1	0.60053	0.013339	0.032047
ARL4D	0.60071	0.0076441	0.019323

C20orf111	0.60126	9.68E-12	6.21E-11
FAM91A1	0.60131	2.61E-32	4.57E-31
NR2F1	0.60133	4.09E-26	5.65E-25
SP8	0.60136	1.30E-05	4.97E-05
RFX7	0.60163	8.77E-19	8.62E-18
RACGAP1	0.60177	5.02E-36	9.95E-35
DDHD1	0.6018	2.93E-13	2.09E-12
GSN	0.60218	2.47E-10	1.43E-09
MED28	0.60227	1.57E-25	2.11E-24
SRPX	0.60231	6.93E-08	3.31E-07
GINM1	0.60241	1.85E-19	1.87E-18
CLSTN3	0.6025	3.62E-07	1.62E-06
CREB1	0.60252	2.85E-20	3.01E-19
PSEN1	0.60253	6.86E-24	8.58E-23
NT5DC2	0.6031	9.85E-24	1.22E-22
CCNB2	0.6034	2.05E-27	2.98E-26
COX10-AS1	0.60369	7.96E-08	3.78E-07
MRS2	0.60372	1.46E-19	1.49E-18
ARMC1	0.60386	3.71E-24	4.68E-23
TMEM234	0.60406	0.00051063	0.0015876
UXS1	0.60431	3.48E-19	3.50E-18
MAFK	0.60433	2.98E-15	2.39E-14
SMC4	0.6044	1.68E-29	2.65E-28
GDNF	0.60464	0.00083512	0.0025073
IRF2BP2	0.60514	1.38E-35	2.71E-34
POLR2D	0.60518	7.19E-15	5.65E-14
PRKY	0.60547	2.69E-12	1.79E-11
RP11-22B23.2	0.60573	0.020974	0.048038
ASB13	0.60653	1.45E-19	1.48E-18
MED17	0.6066	1.27E-22	1.49E-21
AMMECR1L	0.60746	3.44E-12	2.27E-11
SFPQ	0.60748	1.75E-40	4.06E-39
TMEM245	0.6078	1.00E-33	1.83E-32
SNX16	0.60789	0.00014811	0.00049639
UBE2D1	0.60827	1.20E-11	7.61E-11
BMPR1B	0.60888	9.76E-09	5.02E-08
RP11-632C17__A.1	0.60896	4.72E-07	2.08E-06
NPR2	0.60955	4.24E-06	1.70E-05
KANK3	0.60999	2.78E-07	1.25E-06
P2RX5	0.61046	3.38E-07	1.51E-06
MAT2B	0.61123	7.92E-33	1.41E-31
KIF5C	0.61195	1.55E-12	1.06E-11
MORF4L1	0.61217	1.25E-36	2.52E-35
LUZP2	0.61218	2.28E-06	9.38E-06
H3F3A	0.6136	1.03E-44	2.76E-43

OBSCN	0.61362	8.27E-05	0.00028622
POLR3D	0.61368	1.24E-23	1.53E-22
DBF4B	0.6137	5.29E-10	2.99E-09
REV1	0.61376	6.48E-22	7.33E-21
NME1	0.61391	1.87E-13	1.35E-12
ARRDC2	0.61403	2.14E-13	1.53E-12
SNX25	0.61537	1.24E-06	5.22E-06
H1FX	0.61601	2.14E-05	7.95E-05
DZIP1	0.61614	8.44E-08	3.99E-07
PCDHGA1	0.6164	0.015251	0.036125
TPR	0.6164	1.39E-36	2.80E-35
SNIP1	0.61645	9.35E-13	6.45E-12
CENPF	0.61646	1.62E-24	2.08E-23
TMEM194A	0.61677	8.65E-32	1.48E-30
PERP	0.61692	2.86E-39	6.34E-38
DCAF12L2	0.61767	2.95E-06	1.20E-05
RAB5B	0.61772	3.86E-21	4.23E-20
RP11-556K13.1	0.6178	0.014935	0.035434
BTBD8	0.61838	0.0091902	0.022855
APOL2	0.6184	9.19E-11	5.47E-10
WDR59	0.61859	7.77E-27	1.10E-25
ADRA2A	0.61922	5.81E-12	3.78E-11
FOXJ3	0.61943	4.59E-36	9.11E-35
CYP4F29P	0.61947	0.0026382	0.0072791
PRRG4	0.61955	3.07E-12	2.03E-11
KIFC1	0.61955	3.01E-29	4.71E-28
NEMF	0.61995	3.17E-15	2.55E-14
CSNK1G1	0.62021	4.27E-21	4.67E-20
STAT4	0.62032	0.0014001	0.0040427
MBTD1	0.62087	1.40E-22	1.63E-21
AC011747.7	0.62129	0.0055245	0.014375
SALL2	0.62179	1.34E-09	7.33E-09
CALM2	0.62187	2.93E-32	5.12E-31
CHGB	0.62194	0.00011208	0.0003817
SLC7A1	0.62201	5.69E-35	1.08E-33
ERC1	0.62203	1.20E-22	1.41E-21
SRD5A1	0.62237	2.33E-15	1.89E-14
ELOVL6	0.62243	3.90E-37	7.97E-36
SNX21	0.6226	4.57E-09	2.41E-08
LINC00657	0.62272	1.55E-37	3.21E-36
PHLDB1	0.62351	3.61E-14	2.73E-13
VAV3	0.62355	3.68E-12	2.42E-11
DMPK	0.62357	3.72E-10	2.12E-09
ZDHHC21	0.62432	7.29E-18	6.77E-17
SLC39A10	0.62447	3.26E-31	5.45E-30

L3MBTL3	0.6247	3.30E-07	1.48E-06
RBM7	0.62478	4.08E-11	2.50E-10
LHFP	0.62508	2.45E-09	1.31E-08
KIAA1211	0.62525	5.71E-15	4.51E-14
GSPT2	0.62535	0.016015	0.037721
FBLN1	0.62553	3.62E-16	3.07E-15
TSC22D3	0.62568	1.23E-08	6.25E-08
FNBP1	0.62629	1.43E-24	1.83E-23
ATPAF1	0.62729	9.94E-32	1.69E-30
ARL9	0.62754	0.0015964	0.0045688
ZNRF2	0.62754	9.79E-06	3.78E-05
STXBP5	0.62761	3.92E-18	3.69E-17
SDHAF2	0.62807	9.06E-05	0.00031223
PRKAR1A	0.62821	3.41E-43	8.62E-42
PCGF3	0.62831	8.02E-31	1.32E-29
TMEM170B	0.62865	3.17E-17	2.86E-16
CCDC18	0.62869	9.82E-10	5.44E-09
GSG2	0.62871	4.89E-10	2.77E-09
MIA3	0.6288	8.02E-39	1.74E-37
RAB11FIP2	0.62887	4.19E-17	3.75E-16
C17orf62	0.62927	2.90E-15	2.33E-14
ALDH6A1	0.62934	3.67E-46	1.02E-44
PKP4	0.62967	2.28E-24	2.90E-23
MXI1	0.62969	4.77E-25	6.27E-24
DLEU2	0.62996	2.15E-10	1.24E-09
GSDMB	0.63002	9.85E-05	0.00033783
MT-ND2	0.63113	4.49E-38	9.44E-37
PLEKHH1	0.63127	6.75E-18	6.27E-17
GPRC5A	0.63147	1.76E-11	1.11E-10
SLC23A2	0.63163	1.57E-18	1.51E-17
TENM1	0.6318	4.68E-05	0.00016698
IQGAP1	0.63242	7.42E-30	1.19E-28
LNX2	0.63253	1.47E-21	1.64E-20
BUB1	0.63255	1.31E-23	1.62E-22
TERF2IP	0.63257	1.31E-36	2.63E-35
SRPK1	0.63261	7.10E-32	1.22E-30
PCDHB15	0.63278	0.00010021	0.0003434
RSPRY1	0.63353	2.44E-23	2.98E-22
CDC42	0.63373	4.84E-41	1.14E-39
AMBRA1	0.63382	1.42E-22	1.66E-21
ABHD13	0.63443	8.98E-13	6.20E-12
GALNT2	0.63505	1.27E-27	1.86E-26
APP	0.63624	2.32E-50	7.28E-49
PCDHGB1	0.63637	0.00011926	0.00040453
RYBP	0.63637	2.68E-25	3.56E-24

CTD-2287O16.1	0.63653	0.0017929	0.0050826
TMEM238	0.63687	9.48E-09	4.88E-08
DACH2	0.63705	8.29E-05	0.00028709
CLN5	0.6371	9.61E-13	6.62E-12
TRPS1	0.63717	8.30E-17	7.28E-16
URGCP	0.63745	2.24E-33	4.07E-32
NEK9	0.63775	1.49E-37	3.08E-36
LAMP1	0.63796	2.75E-41	6.55E-40
TOB1	0.63871	2.76E-44	7.27E-43
CTDSP2	0.63873	3.03E-40	6.95E-39
RNF149	0.63875	3.86E-20	4.06E-19
RNFT1	0.63883	1.25E-07	5.79E-07
SLC5A3	0.63886	1.38E-12	9.44E-12
RP11-848P1.9	0.63911	0.00011082	0.00037783
UBP1	0.63928	1.02E-47	2.95E-46
6-Mar	0.63944	4.33E-42	1.06E-40
KIDINS220	0.63946	2.60E-31	4.38E-30
SYPL1	0.64056	5.53E-25	7.25E-24
FAM72D	0.64075	4.23E-15	3.37E-14
ZNF677	0.64082	7.68E-13	5.33E-12
CNOT11	0.64109	3.42E-38	7.23E-37
CDYL	0.64115	3.48E-19	3.50E-18
RP11-1212A22.1	0.64134	0.0017291	0.0049173
RP11-159H10.3	0.64134	0.00050737	0.0015784
C21orf2	0.64135	0.0042972	0.011409
CDR2	0.64138	1.49E-15	1.22E-14
ARHGEF17	0.64142	1.11E-10	6.56E-10
RP11-46D6.1	0.64156	0.0090787	0.022607
ALDH1A1	0.64159	8.10E-44	2.08E-42
PPP1R11	0.64162	2.24E-18	2.15E-17
GPCPD1	0.64168	9.20E-14	6.77E-13
TBKBP1	0.64188	1.53E-11	9.65E-11
E2F3	0.64245	2.51E-22	2.90E-21
B3GNT2	0.64253	3.88E-15	3.11E-14
ZFHX2	0.64279	2.84E-08	1.40E-07
ABHD5	0.64286	4.58E-16	3.86E-15
AC074117.10	0.64308	7.56E-08	3.59E-07
SENP5	0.64327	1.57E-29	2.48E-28
ITGA2	0.64333	9.03E-20	9.33E-19
HECA	0.64335	2.28E-15	1.85E-14
FGFR1OP	0.64432	2.91E-14	2.22E-13
SPIRE1	0.64433	9.65E-19	9.45E-18
VWCE	0.64496	7.26E-05	0.00025304
STK17B	0.64496	8.08E-12	5.21E-11
MAMDC4	0.64593	1.24E-09	6.81E-09

SLC17A7	0.64682	3.66E-13	2.60E-12
FAM171A2	0.64705	1.11E-07	5.17E-07
UNC93B1	0.64736	2.85E-17	2.58E-16
SIM2	0.64753	5.90E-21	6.41E-20
FAM110B	0.64763	7.32E-29	1.13E-27
SMG1P1	0.64791	0.012978	0.031254
FAM104A	0.64793	3.34E-14	2.53E-13
MCL1	0.64798	4.01E-59	1.53E-57
LAPTM4B	0.64845	4.22E-61	1.70E-59
PARD3	0.64863	3.45E-23	4.17E-22
MPHOSPH6	0.64907	1.44E-11	9.11E-11
BSG	0.6492	2.49E-40	5.74E-39
RRAGC	0.64932	1.04E-15	8.58E-15
C20orf194	0.64997	0.00016192	0.00054033
ADCK1	0.65003	2.57E-12	1.71E-11
HTT	0.65017	6.48E-22	7.33E-21
CTD-2561J22.5	0.65022	4.22E-07	1.87E-06
EVA1B	0.65058	2.94E-07	1.32E-06
KCTD1	0.65104	3.19E-18	3.02E-17
ZNF827	0.65105	0.006712	0.017165
MAP4K4	0.65136	2.21E-35	4.30E-34
ATF3	0.65147	2.43E-22	2.80E-21
ECT2	0.65203	7.50E-39	1.63E-37
RNF2	0.65207	5.77E-19	5.72E-18
WWTR1	0.65234	9.48E-16	7.87E-15
ITGA6	0.6527	3.98E-09	2.11E-08
XPC	0.65287	1.93E-44	5.14E-43
BCL2L12	0.65295	3.81E-22	4.37E-21
HIC2	0.6538	6.82E-06	2.68E-05
RAB1B	0.654	1.05E-35	2.06E-34
CENPP	0.65404	1.63E-06	6.80E-06
UBQLN4	0.65434	2.04E-45	5.58E-44
RBFOX2	0.65447	5.01E-43	1.26E-41
MYO1B	0.65449	1.10E-24	1.43E-23
CSNK1G3	0.65458	1.07E-24	1.38E-23
KANK1	0.65467	2.33E-40	5.38E-39
SUSD5	0.6547	0.0061652	0.015874
MTAP	0.6548	1.02E-22	1.20E-21
ZC2HC1A	0.65494	7.10E-07	3.07E-06
CRYBG3	0.65538	6.34E-06	2.50E-05
DDX60L	0.65562	0.013984	0.033399
HDHD1	0.65627	2.33E-15	1.89E-14
RP11-54O7.1	0.6564	5.41E-09	2.84E-08
BMPR1A	0.65697	2.16E-25	2.88E-24
CYP2S1	0.65698	8.28E-07	3.55E-06

TRIM23	0.65708	2.35E-21	2.59E-20
UBE2Q2	0.65709	1.25E-20	1.34E-19
GPSM1	0.65717	1.38E-23	1.69E-22
USP25	0.65751	3.09E-23	3.74E-22
BLMH	0.65771	1.57E-39	3.53E-38
ATP6V1G1	0.65774	5.31E-37	1.08E-35
H3F3AP4	0.65852	3.25E-24	4.10E-23
RP11-599J14.2	0.65889	0.0034927	0.0094346
SPATA7	0.65904	1.90E-05	7.10E-05
FAT3	0.65964	0.012216	0.029619
SEC23A	0.66009	8.41E-44	2.16E-42
KLHL20	0.66021	2.68E-18	2.55E-17
PPTC7	0.66048	7.45E-34	1.37E-32
GLRB	0.66085	5.75E-07	2.51E-06
ZNF529	0.66091	2.82E-13	2.01E-12
CST3	0.66103	7.00E-32	1.20E-30
LTBR	0.66105	6.80E-49	2.03E-47
REC8	0.66197	0.021778	0.049695
GNA12	0.66212	3.54E-29	5.51E-28
ZNF644	0.66232	5.52E-20	5.76E-19
CASC5	0.66273	9.37E-19	9.19E-18
ERRFI1	0.66278	3.79E-24	4.78E-23
RP11-244J10.1	0.66282	0.016613	0.03896
SLC15A4	0.66289	5.14E-14	3.84E-13
CDH1	0.66325	1.04E-39	2.35E-38
EFR3A	0.66334	2.79E-27	4.04E-26
GRN	0.66343	4.17E-26	5.76E-25
CDADC1	0.66368	4.33E-08	2.10E-07
DNAJC5	0.66458	3.36E-35	6.44E-34
GOLGA1	0.66542	2.30E-23	2.81E-22
VPS37B	0.66544	2.34E-20	2.48E-19
PREPL	0.6657	3.32E-28	4.99E-27
PARP14	0.66576	6.86E-24	8.58E-23
CBX1	0.66632	3.26E-38	6.91E-37
GPHN	0.6667	1.37E-29	2.17E-28
ZC3HAV1L	0.66692	1.87E-09	1.01E-08
CCDC136	0.66692	1.21E-12	8.27E-12
CHMP3	0.66772	3.08E-33	5.56E-32
SAMD4A	0.6683	6.44E-13	4.50E-12
FBXL8	0.6685	2.60E-05	9.55E-05
FAS	0.66854	8.87E-22	9.98E-21
POLR3G	0.6689	1.06E-07	4.96E-07
ZCCHC11	0.66913	8.99E-29	1.38E-27
TNFRSF25	0.66916	1.07E-07	5.02E-07
CLP1	0.66928	1.28E-12	8.74E-12

PPM1L	0.66975	1.57E-07	7.22E-07
TET1	0.66975	1.76E-09	9.52E-09
ATL2	0.66984	1.11E-25	1.51E-24
OSBPL1A	0.67046	1.14E-19	1.17E-18
ELMOD1	0.6706	2.25E-18	2.16E-17
MXD1	0.67091	3.28E-14	2.49E-13
FRAS1	0.67136	1.37E-14	1.07E-13
ZFYVE21	0.67158	2.05E-25	2.75E-24
LPL	0.67181	2.15E-06	8.83E-06
MIR17HG	0.67229	0.0037367	0.010032
KLHL26	0.67247	4.10E-13	2.90E-12
CES2	0.6725	9.00E-32	1.54E-30
RBM4B	0.67259	1.51E-23	1.86E-22
JUP	0.67294	1.15E-32	2.04E-31
ZFP36	0.67322	9.51E-11	5.65E-10
DHRSX	0.67381	6.05E-09	3.16E-08
FAM69B	0.67403	5.87E-12	3.82E-11
PGBD5	0.67406	2.65E-10	1.52E-09
SPRED1	0.67408	1.02E-30	1.67E-29
61E3.4	0.67411	2.44E-05	8.98E-05
MAP3K2	0.67428	7.40E-26	1.02E-24
OTUD5	0.67455	3.62E-29	5.63E-28
JARID2	0.67456	1.63E-19	1.66E-18
EFHD1	0.67505	0.001675	0.0047792
RP11-680F8.3	0.67527	0.016243	0.038161
HIPK3	0.67535	2.09E-29	3.28E-28
ZNF385C	0.67556	0.016945	0.039676
COL7A1	0.67586	4.59E-10	2.60E-09
MAZ	0.67589	3.58E-06	1.45E-05
DNAJB9	0.67619	5.81E-14	4.33E-13
KLF3	0.67631	2.54E-28	3.83E-27
AGPAT9	0.67649	0.014177	0.033802
FZD8	0.67653	1.12E-10	6.63E-10
IRF1	0.67693	8.56E-06	3.33E-05
KIAA1107	0.67715	1.81E-10	1.06E-09
KITLG	0.67741	7.27E-16	6.08E-15
ZBED4	0.67771	1.27E-27	1.87E-26
CASK	0.67779	1.85E-26	2.59E-25
OSR2	0.6779	2.68E-05	9.83E-05
LIMD2	0.67808	0.00077523	0.002341
H2AFY2	0.67814	1.94E-20	2.06E-19
G2E3	0.67837	5.65E-18	5.28E-17
FEM1C	0.67847	9.31E-27	1.32E-25
VAMP3	0.67847	3.84E-32	6.67E-31
EMB	0.6786	1.53E-29	2.42E-28

ERCC6L	0.67862	2.12E-14	1.62E-13
GBE1	0.67865	1.69E-39	3.79E-38
CADM4	0.67893	3.38E-14	2.56E-13
UGT2B7	0.67902	7.94E-06	3.10E-05
FKBP1A	0.67912	2.79E-56	9.97E-55
RLF	0.67913	1.50E-17	1.38E-16
CBX2	0.68006	6.21E-10	3.49E-09
CLDN12	0.68052	1.00E-40	2.33E-39
RNF4	0.68063	1.18E-37	2.45E-36
CALU	0.68084	5.33E-40	1.22E-38
PCDHB6	0.68096	2.00E-07	9.12E-07
STK19	0.68145	1.38E-13	1.00E-12
MIER2	0.68212	8.73E-10	4.86E-09
PPARGC1B	0.68264	3.58E-17	3.22E-16
DEPDC1	0.68295	2.50E-28	3.78E-27
RAVER2	0.68297	2.01E-17	1.84E-16
FNDC3A	0.68298	6.34E-32	1.09E-30
PLCB4	0.68306	4.93E-20	5.16E-19
PRKAB1	0.68328	5.84E-23	6.94E-22
CHSY3	0.68338	0.00021672	0.00070996
ZNF850	0.68342	5.88E-12	3.83E-11
NKX2-5	0.68356	9.68E-14	7.10E-13
RNF111	0.68358	1.76E-24	2.25E-23
PCOLCE2	0.68433	1.62E-16	1.40E-15
ZNF350	0.6845	2.07E-07	9.47E-07
CAMSAP2	0.68462	2.26E-44	5.98E-43
AADAT	0.68517	7.72E-12	4.98E-11
LEPROT	0.68519	3.32E-31	5.54E-30
AGAP3	0.6854	2.65E-31	4.44E-30
ARRDC3	0.68568	1.80E-28	2.73E-27
SUGT1P	0.68608	0.00035978	0.0011425
CPD	0.68608	4.91E-40	1.12E-38
CDC25C	0.68655	1.03E-13	7.53E-13
RP11-248J18.2	0.68697	0.0017201	0.0048956
RPS6KA2	0.68741	9.11E-12	5.85E-11
AXIN1	0.6877	3.78E-30	6.12E-29
DUSP14	0.68794	3.98E-20	4.19E-19
C11orf82	0.68795	5.18E-14	3.87E-13
AC073046.25	0.68848	0.0029125	0.0079797
CD59	0.68876	2.74E-38	5.83E-37
TMEM44	0.68953	4.27E-05	0.00015315
EEF1A1P13	0.68976	0.0017147	0.0048829
ZDHHC1	0.68987	3.73E-12	2.45E-11
MLYCD	0.68991	1.06E-07	4.97E-07
TMEM8A	0.69023	5.90E-22	6.71E-21

XPO4	0.69086	3.51E-28	5.26E-27
RP11-15N24.4	0.69112	0.0090135	0.022459
MICU3	0.69142	2.07E-05	7.68E-05
ABL1	0.69194	9.98E-35	1.89E-33
MLTK	0.69241	7.73E-16	6.45E-15
KIF20B	0.69259	3.07E-07	1.38E-06
DIRC2	0.69269	7.27E-17	6.41E-16
PPP3R1	0.69359	3.29E-30	5.33E-29
GJA1	0.69382	1.13E-42	2.82E-41
UBAC2	0.6939	1.17E-32	2.07E-31
LPGAT1	0.694	2.05E-44	5.44E-43
CAMSAP1	0.69416	2.21E-38	4.71E-37
ABHD8	0.69427	0.0002092	0.00068771
ASTN2	0.69549	2.57E-17	2.33E-16
SLC38A1	0.69662	1.93E-50	6.05E-49
ORMDL3	0.69687	9.86E-27	1.39E-25
CYB561D1	0.69741	2.66E-12	1.77E-11
ARMC3	0.69759	0.015329	0.036282
MFSD2A	0.69782	4.27E-06	1.71E-05
DNAJB6	0.69786	1.46E-48	4.32E-47
UBALD1	0.6984	4.02E-15	3.21E-14
CERKL	0.69941	0.00013883	0.00046703
YBX3	0.7004	8.41E-55	2.92E-53
LIPH	0.70089	6.54E-10	3.67E-09
FOXJ2	0.70147	2.18E-33	3.96E-32
RCOR1	0.7018	5.64E-32	9.73E-31
RP11-159G9.5	0.70197	6.64E-12	4.31E-11
NIPAL2	0.70243	3.55E-27	5.12E-26
GIGYF1	0.70247	1.27E-31	2.16E-30
NBPF16	0.70268	0.020892	0.047885
PNRC1	0.70287	2.40E-24	3.05E-23
ITPR1	0.70325	0.018	0.04189
RP11-755F10.1	0.70376	0.006301	0.016192
WNT3	0.70379	0.019671	0.045362
DSP	0.7045	1.71E-25	2.30E-24
RANBP6	0.70469	1.00E-19	1.03E-18
CDH12	0.70489	5.70E-20	5.94E-19
CTSF	0.70522	1.31E-35	2.57E-34
CERS2	0.70536	6.97E-41	1.63E-39
CYP1A2	0.70554	1.84E-08	9.23E-08
CLCF1	0.70624	0.00075565	0.0022849
FLRT2	0.7067	1.14E-10	6.75E-10
LIPA	0.70673	2.76E-25	3.67E-24
NAV3	0.7074	4.38E-07	1.94E-06
MCU	0.7076	4.82E-24	6.06E-23

ZNF711	0.70788	1.33E-12	9.07E-12
HIC1	0.70822	0.0073021	0.018519
PDLIM5	0.70841	2.87E-43	7.26E-42
IL17D	0.70882	7.40E-18	6.87E-17
LMBRD1	0.70936	1.23E-23	1.52E-22
NLGN3	0.70955	0.0060843	0.015686
SERINC1	0.70977	3.68E-48	1.08E-46
FAM160A1	0.70991	8.15E-14	6.02E-13
TUSC3	0.70996	1.01E-61	4.14E-60
TWIST1	0.71	6.81E-21	7.37E-20
DYRK1A	0.71022	1.17E-38	2.51E-37
APCDD1	0.71027	0.00954	0.023655
VOPP1	0.71053	3.70E-28	5.53E-27
ADIPOR2	0.71102	2.23E-46	6.24E-45
EPDR1	0.71156	4.28E-38	9.01E-37
CLK1	0.7117	2.19E-20	2.32E-19
WWC2	0.71196	1.05E-17	9.66E-17
TNRC18	0.71281	4.98E-08	2.40E-07
TMEM138	0.71289	3.45E-17	3.11E-16
ZCCHC14	0.71348	8.36E-24	1.04E-22
EIF5A2	0.71386	2.98E-17	2.70E-16
ST3GAL1	0.71419	2.24E-62	9.26E-61
TP53BP2	0.71421	3.73E-35	7.13E-34
GPC2	0.71496	7.35E-06	2.88E-05
TGFBR2	0.7154	3.60E-09	1.92E-08
SYTL5	0.71549	5.59E-16	4.69E-15
KIF23	0.71592	1.46E-23	1.80E-22
DEDD	0.71608	3.38E-25	4.46E-24
OXTR	0.71633	7.62E-05	0.00026476
RFK	0.71641	7.02E-36	1.39E-34
PWWP2A	0.71657	3.06E-18	2.91E-17
INO80D	0.71686	3.88E-21	4.24E-20
C10orf54	0.71705	0.019009	0.043998
ZBTB41	0.71707	1.31E-28	2.00E-27
HMGB3	0.71741	7.32E-43	1.83E-41
FGF22	0.71763	0.014718	0.034964
ZIC2	0.71776	7.47E-17	6.58E-16
NECAP2	0.71777	1.21E-24	1.57E-23
SPRYD3	0.71782	2.20E-31	3.72E-30
ATM	0.71783	1.98E-23	2.43E-22
DDX3X	0.71847	2.95E-61	1.20E-59
CASKIN1	0.71865	1.91E-12	1.29E-11
SSH1	0.7189	6.81E-40	1.55E-38
DOCK3	0.71907	1.84E-27	2.69E-26
SIRT7	0.71948	3.72E-17	3.34E-16

HOXA1	0.71954	0.0034109	0.0092307
EIF4E3	0.72033	3.94E-10	2.25E-09
MKRN1	0.7206	3.65E-58	1.36E-56
PARP12	0.72099	5.71E-20	5.95E-19
PACS1	0.72104	2.08E-35	4.06E-34
TTYH3	0.72114	3.94E-26	5.46E-25
FBXL5	0.72118	1.54E-25	2.08E-24
FAM72B	0.72137	2.69E-16	2.29E-15
BAZ2B	0.72163	4.60E-19	4.60E-18
CDCA2	0.72164	1.51E-29	2.39E-28
ANKRD62	0.72187	5.34E-09	2.81E-08
CDH3	0.72192	4.13E-38	8.70E-37
RP11-379K17.12	0.7222	1.25E-06	5.25E-06
SEC14L1	0.72243	6.65E-19	6.57E-18
RAP2C	0.72243	3.47E-28	5.20E-27
TET3	0.7225	1.18E-32	2.08E-31
ATP2B3	0.72263	0.0049858	0.013061
SPRY2	0.72279	7.34E-35	1.39E-33
PIFO	0.72295	0.0013233	0.0038336
CDC42EP4	0.72337	1.42E-11	8.99E-11
ZDHHC7	0.72372	2.63E-40	6.05E-39
CTTNBP2NL	0.72388	3.19E-14	2.42E-13
SECISBP2L	0.7241	9.45E-39	2.04E-37
ASXL1	0.72421	9.14E-39	1.98E-37
DDX19A	0.72509	2.43E-23	2.96E-22
PCDHA8	0.72513	0.0013183	0.0038215
SCML2	0.72514	1.51E-21	1.68E-20
JAZF1	0.7256	3.90E-06	1.57E-05
PARD6B	0.72601	4.78E-11	2.91E-10
MYCBP2	0.72601	9.98E-16	8.27E-15
ANKRD50	0.72628	1.25E-29	1.99E-28
FOXD4	0.72664	9.24E-11	5.50E-10
RAB22A	0.72692	9.04E-41	2.11E-39
NINJ1	0.72716	1.12E-41	2.71E-40
ARID1B	0.72721	2.00E-28	3.04E-27
ADAM23	0.72788	9.96E-07	4.24E-06
MREG	0.72869	9.25E-06	3.58E-05
LSR	0.72894	1.24E-24	1.60E-23
HABP2	0.7292	1.56E-18	1.51E-17
FAM109A	0.72926	8.11E-24	1.01E-22
TMEM135	0.73027	6.26E-21	6.79E-20
FAM60A	0.731	2.52E-43	6.41E-42
PODXL2	0.73121	3.56E-47	1.02E-45
ADAM10	0.7313	3.70E-50	1.16E-48
HTRA4	0.73133	0.0084432	0.021147

C9orf41	0.73205	1.43E-44	3.80E-43
SLCO2A1	0.7322	0.0013753	0.0039743
DTX2	0.73263	1.80E-07	8.25E-07
ADCK3	0.73289	2.93E-58	1.10E-56
MAP3K4	0.73334	1.39E-28	2.13E-27
SH3GL2	0.73336	0.01132	0.027626
GPR125	0.73347	8.79E-44	2.25E-42
ADCY9	0.73363	2.91E-17	2.64E-16
PBX3	0.73403	8.78E-17	7.69E-16
NIPAL1	0.73418	1.31E-11	8.32E-11
CERS1	0.73443	4.08E-06	1.64E-05
PRKAR2A	0.73448	2.39E-57	8.69E-56
PLXNB2	0.73489	4.59E-30	7.40E-29
CCNYL1	0.73495	8.60E-27	1.22E-25
IDS	0.73522	1.33E-34	2.51E-33
GPR176	0.73529	0.011549	0.028137
TMEM123	0.73575	2.60E-46	7.26E-45
ICOSLG	0.73604	1.44E-21	1.60E-20
UGT8	0.7364	2.72E-41	6.50E-40
MOSPD3	0.7366	7.16E-15	5.63E-14
RHNO1	0.7368	3.83E-27	5.52E-26
SLAIN1	0.73717	4.22E-41	9.98E-40
MEF2A	0.73808	5.35E-32	9.26E-31
AC018696.4	0.73811	0.0095941	0.023774
PELI3	0.73897	1.50E-23	1.84E-22
POLH	0.74127	9.11E-34	1.67E-32
FRMD8	0.74135	8.14E-26	1.11E-24
ALB	0.74135	3.45E-67	1.62E-65
AGRN	0.74139	1.08E-08	5.52E-08
RP4-742C19.8	0.74215	0.0045659	0.012064
GLS2	0.74307	1.07E-29	1.70E-28
KDM5B	0.74318	8.00E-38	1.66E-36
TXNDC5	0.74345	3.51E-06	1.42E-05
FJX1	0.74378	0.0026637	0.0073398
LEPREL1	0.74405	7.83E-06	3.06E-05
GLIS2	0.74463	1.93E-08	9.67E-08
ORAI1	0.74506	2.49E-16	2.13E-15
ME1	0.74641	1.64E-19	1.67E-18
C19orf26	0.74732	9.23E-12	5.92E-11
GRIA2	0.74751	0.00061698	0.0018898
SLC9A6	0.74753	1.10E-16	9.62E-16
ABCC4	0.74786	5.99E-20	6.23E-19
NPIPL3	0.74845	0.00072838	0.0022102
HSPA4L	0.74846	2.91E-31	4.88E-30
LRP1B	0.74967	0.0036261	0.009762

SIPA1L2	0.75031	5.92E-45	1.59E-43
DBF4	0.75045	3.63E-23	4.38E-22
LMO7	0.75136	2.26E-27	3.28E-26
RP11-403I13.8	0.75158	0.0005995	0.0018393
FBXL19	0.75165	1.46E-16	1.26E-15
SLC44A5	0.75282	2.42E-06	9.93E-06
ASPM	0.75302	6.08E-51	1.95E-49
SLC25A21	0.75403	1.56E-05	5.90E-05
ZNF281	0.75518	1.26E-24	1.62E-23
MDM2	0.75551	1.06E-50	3.40E-49
TNFRSF14	0.75586	0.0044122	0.011693
CXXC4	0.7569	1.65E-08	8.30E-08
TSPAN14	0.75711	3.02E-40	6.93E-39
SZRD1	0.75733	1.68E-64	7.29E-63
LEF1	0.75833	1.47E-18	1.42E-17
KIAA1549	0.75845	6.14E-22	6.96E-21
DENND6A	0.75962	5.44E-46	1.50E-44
RP11-395P17.3	0.75977	4.06E-08	1.98E-07
CACNA1D	0.75988	6.54E-33	1.17E-31
RTKN2	0.76038	2.52E-22	2.90E-21
KLHL15	0.76058	1.20E-22	1.40E-21
FBXO46	0.76065	6.09E-21	6.60E-20
HBP1	0.76095	1.65E-46	4.67E-45
HNRPDL	0.76153	4.37E-51	1.41E-49
NPNT	0.76173	1.28E-64	5.58E-63
DCAF6	0.76187	7.57E-26	1.04E-24
CNIH3	0.76206	0.012718	0.030712
RNF44	0.76229	1.36E-49	4.16E-48
SPG20	0.76244	0.00072947	0.0022128
REG4	0.76301	6.53E-06	2.57E-05
RP11-597D13.9	0.76316	4.53E-08	2.20E-07
SACS	0.76362	1.66E-23	2.03E-22
CHRDL1	0.76368	0.0085371	0.021356
RP11-512M8.3	0.76412	1.38E-14	1.07E-13
UNC5B	0.76424	3.07E-08	1.51E-07
ADAM7	0.76483	0.013581	0.03255
SCAMP1	0.76511	4.47E-48	1.30E-46
PLXNB1	0.76532	5.22E-28	7.77E-27
CNIH	0.76608	3.56E-42	8.70E-41
TNFSF9	0.76658	0.00014279	0.00047928
S100A11	0.76671	4.97E-69	2.48E-67
RDH10	0.76673	5.54E-23	6.59E-22
LRCH1	0.76689	5.79E-11	3.50E-10
C14orf37	0.76763	2.34E-08	1.16E-07
TUSC2	0.76777	3.27E-41	7.77E-40

PON2	0.7687	1.19E-49	3.66E-48
ARID3B	0.76917	1.27E-08	6.47E-08
NEK2	0.76934	1.56E-50	4.95E-49
CNN2	0.76966	2.82E-31	4.73E-30
KCTD2	0.76974	8.31E-37	1.69E-35
TSC22D2	0.76993	2.26E-29	3.54E-28
AFF1	0.77079	2.95E-32	5.14E-31
ISG20	0.77095	2.41E-12	1.61E-11
TNIP2	0.771	1.61E-28	2.45E-27
GNPTAB	0.7712	6.21E-69	3.09E-67
SPATA25	0.77125	0.018869	0.043712
PRKCE	0.77137	4.80E-07	2.11E-06
CTD-2145A24.3	0.77266	0.02092	0.047938
TAF5	0.77322	1.06E-13	7.75E-13
YAF2	0.77489	1.08E-19	1.12E-18
RNF24	0.77496	5.40E-12	3.52E-11
ARID2	0.77524	1.84E-46	5.20E-45
RELN	0.77573	0.0013139	0.0038098
LIMCH1	0.77621	1.30E-55	4.57E-54
CDC37L1	0.77635	1.10E-20	1.18E-19
HSD17B11	0.77651	2.50E-31	4.22E-30
CABLES1	0.77711	1.13E-07	5.30E-07
AC003088.1	0.77736	0.0072887	0.018494
AC125232.1	0.77755	3.01E-07	1.35E-06
CTNND2	0.7777	8.74E-16	7.27E-15
RP11-173M1.8	0.7778	0.015579	0.036805
CTD-2228K2.7	0.77808	0.0015405	0.0044205
CTB-36H16.2	0.77811	1.86E-06	7.69E-06
BRAF	0.77834	6.63E-41	1.56E-39
F2R	0.77904	4.16E-06	1.67E-05
ENPP4	0.77968	5.15E-18	4.82E-17
GC	0.77995	1.37E-11	8.66E-11
XPR1	0.78009	6.36E-50	1.97E-48
RBPMS2	0.78021	2.11E-17	1.92E-16
PIANP	0.7804	0.00019305	0.00063786
GLUL	0.78054	1.47E-71	7.87E-70
EFCAB2	0.78133	1.29E-13	9.41E-13
TMEM43	0.78173	5.27E-39	1.16E-37
RNF139	0.78176	5.87E-36	1.16E-34
AC112229.1	0.78247	0.020554	0.047177
NAP1L6	0.78291	0.00060674	0.0018604
CORO1C	0.78336	1.06E-55	3.75E-54
ZNF649	0.78342	1.31E-09	7.15E-09
DUSP6	0.78381	5.08E-39	1.12E-37
SLC22A3	0.78446	3.50E-15	2.80E-14

VANGL1	0.78474	8.89E-27	1.26E-25
ETV1	0.78553	1.65E-36	3.31E-35
DNAJB5	0.78583	5.47E-12	3.56E-11
JMY	0.78807	1.75E-42	4.33E-41
MTND2P28	0.78808	1.39E-24	1.79E-23
MAN1A1	0.78809	5.83E-48	1.70E-46
DBN1	0.78829	6.72E-44	1.74E-42
RYR3	0.7886	0.0022573	0.0063096
CDK1	0.78927	1.89E-39	4.23E-38
SLC25A26	0.78939	1.08E-21	1.22E-20
KISS1	0.78967	6.96E-05	0.00024314
AC092171.1	0.79063	0.013423	0.032226
RP1-59D14.3	0.79087	0.014519	0.034534
DIAPH2	0.79104	3.10E-32	5.40E-31
TMX4	0.79106	4.13E-50	1.29E-48
TAB2	0.79131	2.10E-33	3.81E-32
DHRS3	0.79149	1.19E-31	2.02E-30
LACC1	0.79154	1.00E-07	4.71E-07
GLCCI1	0.7916	6.44E-21	6.97E-20
RBP4	0.79165	0.003939	0.010517
MAFF	0.79194	6.72E-19	6.63E-18
SGOL2	0.79269	7.90E-18	7.32E-17
RP11-119F7.5	0.79326	0.016355	0.038404
PIK3R3	0.79335	5.34E-44	1.39E-42
CDC42BPG	0.79357	1.21E-36	2.45E-35
EPC2	0.79403	7.55E-23	8.91E-22
GPR180	0.79416	8.39E-27	1.19E-25
GPR143	0.79536	0.00021919	0.00071746
FRAT1	0.79565	9.00E-14	6.63E-13
TNXB	0.79618	0.00044863	0.0014064
SYNE1	0.79623	4.29E-20	4.50E-19
BMF	0.79657	1.29E-16	1.12E-15
SKIDA1	0.79724	1.26E-10	7.44E-10
ROBO2	0.79765	1.76E-09	9.54E-09
FAM72A	0.79784	3.53E-20	3.71E-19
ATP11C	0.7992	1.95E-34	3.67E-33
PNRC2	0.79961	1.67E-53	5.64E-52
BRICD5	0.79977	0.0015536	0.0044547
BBX	0.80001	2.97E-55	1.04E-53
COLGALT1	0.8001	1.35E-60	5.37E-59
NACC1	0.80016	3.42E-40	7.85E-39
ZNF385A	0.80023	3.71E-31	6.17E-30
GOLIM4	0.8007	1.85E-73	1.03E-71
RP11-164P12.4	0.80091	2.98E-05	0.00010857
CAPRIN2	0.80148	4.78E-17	4.26E-16

MEF2C	0.80159	1.40E-14	1.09E-13
ATP6V0E1	0.80189	1.04E-41	2.52E-40
FAM13C	0.80222	8.27E-07	3.55E-06
TMEM55B	0.80227	9.76E-19	9.55E-18
DCAF15	0.80294	1.76E-25	2.36E-24
COBLL1	0.80365	2.64E-34	4.94E-33
ATF7	0.80426	4.98E-27	7.11E-26
NPR3	0.80432	3.23E-10	1.85E-09
PER1	0.80438	8.75E-29	1.35E-27
HERC3	0.80565	4.95E-18	4.64E-17
CHD9	0.80623	5.95E-32	1.03E-30
SIX1	0.80786	2.46E-08	1.22E-07
IL6R	0.80792	1.34E-45	3.68E-44
C2orf42	0.80866	8.88E-10	4.94E-09
PARM1	0.80886	1.33E-42	3.29E-41
ITM2A	0.80946	0.0014368	0.0041401
USP3	0.80973	1.01E-39	2.29E-38
ELK1	0.80986	8.83E-45	2.36E-43
RNF19B	0.80999	4.51E-21	4.92E-20
ZFP36L1	0.81004	9.44E-57	3.39E-55
PDE4C	0.8101	0.015452	0.036547
RNF150	0.81014	5.52E-11	3.34E-10
MAN2A1	0.81106	2.45E-49	7.45E-48
PHF16	0.81183	1.60E-28	2.44E-27
BCL6	0.81206	3.53E-31	5.87E-30
JHDM1D	0.81256	1.29E-42	3.22E-41
ATG14	0.81283	1.69E-20	1.80E-19
AC114776.1	0.81325	0.016936	0.039659
MT-ATP8	0.8134	0.018389	0.042714
PRICKLE1	0.81637	0.0060731	0.015663
SREK1IP1	0.81694	1.70E-42	4.20E-41
SUMF1	0.81705	1.41E-46	4.00E-45
RP11-65F13.2	0.81712	2.05E-20	2.17E-19
FBXO25	0.81744	6.60E-43	1.66E-41
CDCA8	0.81883	4.86E-49	1.46E-47
RRN3	0.81886	2.67E-68	1.32E-66
MSRB3	0.81946	1.85E-06	7.64E-06
PCNPP1	0.81957	0.015936	0.037568
SESN1	0.81962	3.90E-45	1.05E-43
GUCY1A3	0.82287	1.28E-17	1.18E-16
SEMA7A	0.82295	0.00017553	0.00058351
FGB	0.82301	0.019509	0.045032
RNF103	0.82429	2.73E-17	2.48E-16
FAM102A	0.82445	2.35E-27	3.42E-26
NPM1P9	0.8249	0.008511	0.021296

EPAS1	0.82525	1.23E-11	7.83E-11
ZNF654	0.82544	1.32E-25	1.79E-24
NRBP1	0.82554	3.67E-37	7.51E-36
GAD1	0.82682	0.014171	0.033793
KIAA0355	0.82722	1.03E-24	1.33E-23
C1QTNF3	0.82831	0.0045289	0.011972
FLRT3	0.82844	5.17E-22	5.88E-21
FAM46C	0.82893	1.61E-07	7.43E-07
MPDZ	0.829	4.94E-28	7.36E-27
ANKS1A	0.82912	3.04E-42	7.46E-41
LINC00086	0.82914	9.02E-12	5.80E-11
RP11-458I7.4	0.82954	0.015993	0.037676
RP11-661A12.9	0.82977	0.01768	0.041198
PTPRU	0.83047	5.50E-28	8.17E-27
WIPF1	0.83062	0.00081353	0.0024474
TCF4	0.83181	2.43E-25	3.24E-24
NTN4	0.83192	0.014463	0.034428
OSTM1	0.83232	8.27E-29	1.28E-27
TUBB6	0.83237	2.38E-46	6.66E-45
ENTPD4	0.83278	2.46E-51	7.95E-50
BMP1	0.83321	6.47E-28	9.60E-27
ITGB1	0.83324	4.66E-74	2.63E-72
IL6ST	0.83405	1.13E-36	2.29E-35
SCARA3	0.83437	3.85E-37	7.87E-36
ARHGAP20	0.83454	2.17E-38	4.63E-37
ELMOD2	0.8351	1.87E-36	3.73E-35
FANCB	0.8353	2.95E-07	1.33E-06
CXADR	0.83576	1.70E-35	3.32E-34
DLX1	0.83796	5.02E-10	2.84E-09
MRAP2	0.83813	0.014237	0.033929
ANKRD29	0.83835	8.04E-11	4.81E-10
CEP85L	0.8394	2.25E-12	1.51E-11
SLC38A2	0.83983	5.94E-71	3.13E-69
CNOT6L	0.84009	1.99E-38	4.26E-37
PRICKLE2	0.8403	3.82E-07	1.70E-06
OTUD1	0.84098	2.53E-10	1.46E-09
FAM65B	0.84172	7.20E-33	1.28E-31
NCS1	0.84257	5.38E-58	2.00E-56
SPRY4	0.84283	5.97E-23	7.08E-22
MCC	0.84425	0.00045963	0.0014396
LL0XNC01-7P3.1	0.84431	0.016747	0.039242
RB1CC1	0.84567	3.89E-66	1.79E-64
CEP120	0.84632	6.06E-21	6.58E-20
C12orf49	0.84651	2.70E-59	1.03E-57
LSM14B	0.84712	1.12E-59	4.33E-58

MT-ND1	0.84747	6.25E-98	5.79E-96
PCDHGA11	0.8479	5.42E-05	0.00019241
FBXW7	0.84884	2.03E-28	3.07E-27
TCF7L2	0.84959	1.54E-22	1.80E-21
TEP1	0.84999	1.58E-23	1.94E-22
MFNG	0.85048	0.00088876	0.0026572
C12orf5	0.85181	3.19E-24	4.03E-23
NCDN	0.8521	7.18E-54	2.43E-52
RRN3P3	0.85324	7.54E-07	3.25E-06
KLHDC8A	0.85335	0.0019521	0.0055061
HS6ST2	0.85359	5.50E-28	8.17E-27
ULK2	0.85361	5.17E-39	1.14E-37
FAM13B	0.85432	3.00E-30	4.87E-29
GPR126	0.85468	1.99E-83	1.34E-81
GHR	0.85519	2.67E-29	4.18E-28
ELL	0.8556	2.24E-16	1.92E-15
ENPP5	0.85631	3.18E-18	3.01E-17
RNF217	0.85657	0.00036281	0.0011518
PARP8	0.85668	7.94E-19	7.82E-18
MAP3K14	0.85784	1.68E-25	2.27E-24
B3GNT1	0.85807	7.70E-34	1.41E-32
LAMA3	0.8586	1.79E-17	1.64E-16
CNTFR	0.8588	1.19E-09	6.54E-09
TBC1D1	0.85895	2.16E-48	6.36E-47
UBE2E1	0.86183	2.66E-43	6.73E-42
ADSS	0.86228	2.13E-37	4.37E-36
GRAMD3	0.86231	4.00E-12	2.62E-11
SEMA3A	0.86296	0.015479	0.036594
PARD6G	0.86301	7.18E-24	8.98E-23
AMH	0.86346	1.09E-07	5.09E-07
CABYR	0.8635	3.24E-06	1.31E-05
RP11-420A23.1	0.86378	0.011785	0.028671
PRDM2	0.8653	1.12E-38	2.41E-37
LCORL	0.86542	3.02E-27	4.37E-26
SLC25A12	0.86546	1.89E-25	2.53E-24
HOTAIR	0.86561	0.0037913	0.010166
E2F5	0.86596	3.08E-27	4.46E-26
SMURF2	0.86597	7.88E-39	1.71E-37
MMP16	0.86612	5.52E-56	1.96E-54
FAM133DP	0.867	0.0066728	0.01708
LDLR	0.86811	1.79E-21	1.98E-20
AVL9	0.86823	1.23E-75	7.24E-74
IMPDH1P4	0.8689	0.018436	0.042818
NHS	0.86899	4.19E-15	3.35E-14
ACVR1	0.8716	2.10E-10	1.22E-09

GFPT1	0.87168	2.13E-53	7.15E-52
UGT2B28	0.87224	0.00017736	0.00058922
TVP23B	0.87399	2.15E-41	5.14E-40
EPB41L2	0.874	7.59E-32	1.30E-30
FAM43A	0.87427	5.09E-12	3.32E-11
PPM1F	0.8745	3.72E-57	1.34E-55
IGF2BP1	0.87471	2.73E-17	2.48E-16
ELOVL4	0.87687	3.50E-49	1.06E-47
SKI	0.87692	3.01E-37	6.17E-36
MFGE8	0.87698	7.49E-30	1.20E-28
MYL9	0.87734	5.22E-09	2.74E-08
FLJ27365	0.87797	3.03E-07	1.36E-06
FAM57A	0.8782	2.15E-40	4.96E-39
HDGFRP3	0.87861	2.88E-42	7.07E-41
MDGA1	0.88331	0.00037424	0.0011861
AC093673.5	0.88338	0.00024335	0.00079082
SPATA2	0.88623	3.63E-25	4.78E-24
GADD45A	0.88628	1.24E-43	3.17E-42
SESN3	0.88647	1.97E-54	6.79E-53
FOXL2	0.88732	1.64E-06	6.81E-06
E2F6	0.8882	3.94E-25	5.19E-24
MAP3K5	0.89043	3.54E-45	9.57E-44
PHTF2	0.89059	6.75E-77	4.06E-75
ERO1L	0.89068	9.27E-68	4.46E-66
CEACAM1	0.89089	1.02E-05	3.92E-05
SESN2	0.89107	7.50E-58	2.77E-56
EPPK1	0.89178	3.00E-12	1.99E-11
KCNB1	0.89258	0.00035703	0.0011341
ZNF367	0.8928	4.36E-34	8.05E-33
CD164	0.89379	1.20E-67	5.77E-66
WBP1L	0.89498	6.01E-50	1.86E-48
LONRF1	0.89557	2.51E-56	8.97E-55
FAM181B	0.89594	0.014467	0.034434
CX3CL1	0.89647	3.14E-50	9.81E-49
LINC00869	0.89649	0.011581	0.028212
YPEL2	0.89772	2.55E-19	2.58E-18
RP11-252A24.7	0.89837	1.70E-28	2.58E-27
10-Sep	0.89879	5.15E-24	6.46E-23
AC005258.3	0.89947	0.019932	0.045918
RPS6KA5	0.90023	1.46E-13	1.06E-12
RNMT	0.9009	8.90E-48	2.58E-46
PCDHB18	0.90129	6.15E-10	3.46E-09
TNKS2	0.90149	1.72E-74	9.86E-73
CCDC80	0.90167	1.63E-31	2.76E-30
FLNA	0.90173	2.66E-40	6.12E-39

TRNP1	0.90192	4.79E-63	2.03E-61
BRWD1	0.90249	6.64E-55	2.31E-53
GDF15	0.90278	1.92E-43	4.90E-42
RPL23AP53	0.90338	1.83E-08	9.16E-08
RP11-676M6.1	0.90458	0.00066622	0.0020317
FAM117B	0.9049	2.19E-50	6.89E-49
UBXN10	0.90563	0.0021893	0.0061335
CCNT2	0.90573	8.34E-34	1.53E-32
YPEL1	0.90582	1.70E-06	7.06E-06
GOLGA8A	0.906	4.75E-09	2.51E-08
RP11-588K22.2	0.90648	7.15E-08	3.41E-07
TTK	0.90725	9.95E-43	2.49E-41
ITGB6	0.90747	0.00021379	0.00070168
WASL	0.90761	2.49E-79	1.57E-77
RFTN1	0.90958	4.35E-05	0.00015588
RASSF8	0.90986	2.30E-12	1.54E-11
SOX4	0.90988	6.33E-83	4.23E-81
RASA2	0.91016	1.06E-21	1.19E-20
ZBTB47	0.91104	1.28E-27	1.88E-26
DPYSL4	0.91205	1.45E-08	7.31E-08
DGKH	0.91221	3.43E-07	1.53E-06
BCL7A	0.91245	2.30E-66	1.06E-64
KPNA2	0.91403	7.52E-71	3.92E-69
ING1	0.91419	2.30E-28	3.48E-27
B4GALT6	0.91496	1.39E-14	1.08E-13
PDE1B	0.91562	0.00099362	0.0029473
TUBD1	0.91582	1.46E-15	1.20E-14
MIR3188	0.91583	0.0171	0.03999
B4GALT5	0.91731	3.47E-68	1.70E-66
APBA2	0.91767	1.98E-57	7.24E-56
FNIP1	0.91793	1.20E-39	2.70E-38
CACNA1H	0.91877	0.0026059	0.0071986
RNF19A	0.91882	4.95E-31	8.21E-30
DYRK2	0.91882	6.36E-49	1.91E-47
TP73	0.92022	1.16E-06	4.89E-06
HADHB	0.92032	6.95E-88	5.15E-86
ZBTB4	0.92097	1.13E-59	4.37E-58
MTSS1L	0.92141	1.63E-32	2.87E-31
TMEM109	0.92324	1.31E-70	6.72E-69
BBC3	0.92368	8.61E-44	2.21E-42
ST8SIA4	0.92413	6.31E-31	1.04E-29
SEMA6D	0.92419	0.0070293	0.017904
AGO2	0.92573	1.47E-25	1.99E-24
THAP2	0.92685	1.96E-11	1.22E-10
AL020996.1	0.92722	0.021826	0.049784

IMPDH1	0.92756	2.46E-63	1.04E-61
TREX2	0.92826	0.013758	0.032932
SOBP	0.92895	4.20E-16	3.56E-15
ARMC8	0.93163	8.30E-48	2.41E-46
FCHSD2	0.93203	3.90E-30	6.30E-29
OTUD4	0.9322	4.99E-61	2.01E-59
TCFL5	0.93226	1.97E-35	3.84E-34
SEMA4C	0.93434	6.19E-30	9.91E-29
SYTL2	0.93544	7.24E-71	3.79E-69
KDM3A	0.93566	7.50E-48	2.18E-46
RP11-372K14.2	0.93599	0.011082	0.027119
SNRPN	0.93607	0.005313	0.013856
YAP1	0.93706	1.22E-86	8.85E-85
TMEM86A	0.93773	5.35E-08	2.58E-07
NACC2	0.93782	9.62E-48	2.78E-46
ARHGAP28	0.93789	9.58E-06	3.70E-05
SNN	0.93851	1.47E-21	1.64E-20
MARCKS	0.93982	3.19E-124	4.22E-122
TMEM127	0.94034	6.51E-73	3.56E-71
SATB2	0.94065	2.98E-24	3.78E-23
LUC7L	0.94116	5.54E-44	1.44E-42
CGN	0.94294	6.09E-96	5.37E-94
HCN2	0.94319	6.89E-17	6.08E-16
BICD2	0.94327	4.20E-59	1.60E-57
RNF38	0.94439	1.42E-48	4.22E-47
RP11-122A3.2	0.94523	0.01936	0.044726
RALGDS	0.94575	7.38E-63	3.09E-61
ATP8B5P	0.94579	0.015466	0.036568
HGSNAT	0.94584	1.40E-77	8.56E-76
EREG	0.94614	0.010838	0.026584
TAS2R14	0.94818	0.0069835	0.017794
EEF1A1P12	0.95011	0.00077312	0.0023353
KPNA3	0.95036	5.40E-54	1.83E-52
PTGER4	0.95124	3.79E-17	3.40E-16
NEURL1B	0.9517	5.89E-59	2.23E-57
CDKN1C	0.95263	6.43E-08	3.08E-07
IL17RD	0.95431	3.42E-55	1.19E-53
C21orf91	0.95443	1.84E-39	4.12E-38
MYADM	0.95485	4.98E-44	1.30E-42
CERS5	0.95511	2.19E-54	7.53E-53
SCML1	0.95514	4.64E-62	1.91E-60
RP11-295D4.1	0.9555	0.0050026	0.013099
ALCAM	0.95561	2.76E-104	2.75E-102
TCP11L2	0.95749	8.65E-20	8.95E-19
RP11-701H24.2	0.95818	5.15E-19	5.12E-18

AP4S1	0.9587	3.31E-05	0.00012014
TMEM25	0.96016	2.69E-61	1.10E-59
GNB3	0.96142	7.74E-10	4.32E-09
SOWAHC	0.96395	4.42E-14	3.32E-13
C3orf58	0.96541	2.00E-79	1.28E-77
KPNA5	0.96564	6.27E-13	4.38E-12
AHCTF1P1	0.96569	0.003054	0.0083387
EEF1A1P29	0.96588	0.011384	0.027771
KDSR	0.96667	1.49E-55	5.25E-54
ZNF275	0.96715	2.58E-45	7.01E-44
CDK8	0.9678	8.16E-39	1.77E-37
FAM212B	0.96788	4.10E-15	3.27E-14
ITIH5	0.96819	0.017147	0.040094
ACBD7	0.96861	1.07E-15	8.82E-15
WWP1	0.96877	1.35E-60	5.38E-59
SLC30A7	0.96934	3.59E-53	1.19E-51
C2orf69	0.97021	8.91E-40	2.02E-38
ABTB1	0.97188	1.79E-34	3.38E-33
FLJ00418	0.97281	0.010929	0.026781
RASGEF1B	0.97288	0.00050123	0.0015609
RPS10P3	0.97306	0.016664	0.039063
DCP2	0.97306	1.84E-66	8.53E-65
COLGALT2	0.97356	1.71E-12	1.15E-11
CHODL	0.97357	2.71E-28	4.08E-27
PBXIP1	0.97402	5.62E-81	3.62E-79
FAM217B	0.97492	1.15E-48	3.45E-47
CTBP2	0.97563	2.72E-34	5.09E-33
COX5BP6	0.97594	0.0068119	0.017393
SOX2	0.97723	0.007573	0.019164
HSPG2	0.97774	0.00030412	0.00097683
ANKIB1	0.97794	1.70E-81	1.10E-79
SKIL	0.97849	1.33E-53	4.49E-52
ADAMTS6	0.97939	1.49E-24	1.91E-23
DNAJC27	0.97959	8.67E-17	7.60E-16
ABCC5	0.97997	2.84E-77	1.73E-75
ANLN	0.98021	3.62E-75	2.11E-73
IER2	0.98113	1.42E-49	4.32E-48
GXYLT1	0.98139	4.05E-74	2.30E-72
MEX3D	0.98144	3.08E-26	4.28E-25
CERCAM	0.98262	5.97E-30	9.57E-29
NETO1	0.98263	1.45E-21	1.62E-20
RP11-30P6.6	0.98328	0.014017	0.033459
GPC4	0.98353	8.01E-60	3.11E-58
ZNF667	0.98455	3.46E-31	5.77E-30
SNAI1	0.98501	3.38E-05	0.00012247

PRDM1	0.98584	0.00019382	0.00064018
CSGALNACT1	0.98623	2.62E-14	2.00E-13
CILP2	0.98652	6.91E-38	1.44E-36
FBXO32	0.98774	3.52E-14	2.66E-13
CTD-2666L21.2	0.98932	0.013986	0.033399
LRP3	0.98949	3.24E-34	6.03E-33
AGPAT6	0.98951	6.54E-83	4.36E-81
STX3	0.99275	2.93E-67	1.37E-65
RBMS1	0.99342	0.0093581	0.023234
CADM1	0.99368	1.03E-11	6.61E-11
RNF125	0.99534	4.97E-17	4.42E-16
RP11-535A19.1	0.99611	0.007367	0.018675
RP5-884C9.2	0.99774	0.0088444	0.022061
TGFB2	0.99776	0.0025103	0.0069579
GLG1	0.99813	3.29E-62	1.36E-60
LPPR3	0.99874	0.012448	0.03013
WBP1LP2	0.99919	0.0033724	0.0091372
PRSS23	1.0008	9.21E-109	1.00E-106
ITGA4	1.0012	4.37E-23	5.23E-22
TRHDE	1.0014	2.32E-34	4.37E-33
SLC38A9	1.0022	5.03E-20	5.26E-19
MAST4	1.0029	1.12E-21	1.25E-20
RND3	1.0034	6.16E-66	2.81E-64
AMER1	1.0056	7.41E-28	1.10E-26
RP3-337H4.8	1.0064	2.93E-16	2.50E-15
PLEKHA3	1.0078	4.17E-35	7.97E-34
EPHA7	1.0078	2.24E-92	1.85E-90
LRP1	1.0086	7.15E-17	6.30E-16
WASF3	1.0093	6.95E-71	3.65E-69
SEMA4B	1.0095	2.57E-20	2.72E-19
FAXDC2	1.0113	3.26E-72	1.77E-70
LAPTM4A	1.0122	4.46E-77	2.70E-75
RP11-777F6.3	1.0132	0.0034281	0.0092747
RASGEF1A	1.0151	1.18E-67	5.66E-66
ODC1	1.0164	2.05E-149	4.16E-147
IQCJ-SCHIP1	1.0166	2.98E-06	1.21E-05
C19orf40	1.0167	2.57E-11	1.60E-10
KIAA1432	1.0169	5.78E-45	1.55E-43
YPEL3	1.019	3.17E-37	6.48E-36
BRD3	1.0216	2.70E-54	9.27E-53
GPR157	1.0222	3.84E-23	4.62E-22
ST3GAL5	1.0234	3.19E-09	1.70E-08
GLI3	1.0242	4.76E-20	4.98E-19
TMEM87A	1.0242	6.16E-66	2.81E-64
RP11-713C5.1	1.0254	4.22E-07	1.87E-06

ABCA1	1.026	1.95E-08	9.76E-08
PCDHB11	1.0269	3.45E-23	4.16E-22
CNNM2	1.0278	6.14E-31	1.02E-29
OR8G5	1.0282	0.00063967	0.0019536
FAM71F2	1.0297	0.0028853	0.0079167
MMP11	1.0307	6.59E-16	5.52E-15
SERTAD1	1.0315	6.27E-26	8.64E-25
AC018633.4	1.0316	0.010297	0.025353
PGAP1	1.0321	8.32E-58	3.05E-56
UNC5B-AS1	1.0355	0.00073226	0.0022194
G3BP2	1.0357	2.43E-97	2.24E-95
CSMD3	1.039	0.00081175	0.0024435
HOXC13	1.0396	2.70E-12	1.79E-11
TSPAN9	1.0401	2.39E-34	4.49E-33
NDN	1.0402	1.39E-50	4.40E-49
ZNFX1	1.041	4.59E-54	1.57E-52
UBE2E2	1.0417	2.89E-57	1.05E-55
PDIA2	1.042	3.62E-11	2.23E-10
TNRC6C	1.0427	8.71E-26	1.19E-24
MEX3C	1.0429	6.78E-84	4.61E-82
ID2	1.0433	1.45E-88	1.10E-86
PBX2	1.0441	2.80E-69	1.40E-67
PIK3AP1	1.0458	1.38E-09	7.55E-09
CCDC50	1.0461	4.03E-66	1.85E-64
BASP1	1.0474	8.02E-09	4.15E-08
RP11-603B24.1	1.0479	0.0047658	0.01255
DICER1	1.0479	3.34E-94	2.87E-92
RASD1	1.0487	0.0013796	0.0039856
FOXO1	1.0492	1.05E-18	1.03E-17
FOXP1	1.0497	2.44E-48	7.17E-47
PCDH9	1.0502	1.67E-17	1.53E-16
AC097721.2	1.0517	0.0025859	0.0071484
SCRT1	1.0523	0.0056386	0.01464
SCGN	1.0528	7.53E-06	2.94E-05
FBXO33	1.0541	2.07E-57	7.55E-56
PITPNC1	1.0545	8.59E-11	5.13E-10
RP11-175K6.1	1.0556	0.0067456	0.017234
CTA-204B4.6	1.0558	1.03E-89	7.97E-88
GDA	1.0563	0.006271	0.016129
KCNK15	1.0571	0.0092417	0.022972
AC005329.7	1.0584	0.002311	0.0064483
HMGB1P41	1.0592	0.0083602	0.020982
TNPO1	1.0602	2.59E-114	3.09E-112
ATP1B1	1.0605	9.28E-96	8.13E-94
VLDLR	1.0611	1.07E-13	7.86E-13

MYLIP	1.0612	2.67E-30	4.35E-29
BAMBI	1.0623	4.05E-33	7.28E-32
AHR	1.0623	7.96E-56	2.83E-54
RP11-361F15.2	1.0629	3.79E-07	1.68E-06
SULF1	1.065	1.85E-08	9.26E-08
PPAP2B	1.0659	1.92E-50	6.04E-49
BMP6	1.0661	4.73E-17	4.21E-16
CLSTN2	1.0669	0.002826	0.0077653
RHOB	1.0693	1.37E-76	8.20E-75
GNAI1	1.0697	1.43E-64	6.22E-63
SEMA3E	1.0709	5.10E-21	5.54E-20
NRG3	1.071	0.0048414	0.012725
TRIM71	1.0719	0.0084289	0.021116
TPM2	1.0721	7.94E-08	3.77E-07
LINC00552	1.0733	0.0082652	0.020764
AC006978.6	1.0742	1.00E-05	3.88E-05
IGFBP3	1.0785	4.06E-143	7.25E-141
ANKRD13B	1.0789	1.56E-64	6.77E-63
FOXA2	1.0801	0.0070529	0.017957
WEE1	1.0802	1.01E-72	5.51E-71
SUN2	1.0812	3.37E-71	1.78E-69
RP3-412A9.10	1.0827	0.00011601	0.0003939
RP11-134G8.8	1.0833	3.53E-27	5.10E-26
SBK1	1.0843	1.67E-32	2.93E-31
RBM38	1.0852	3.43E-60	1.35E-58
TGM1	1.0864	1.56E-10	9.13E-10
FYCO1	1.0865	1.12E-94	9.73E-93
RNF148	1.087	0.00091953	0.0027433
MTMR9	1.087	3.65E-42	8.91E-41
DRD5P1	1.0879	0.0070792	0.018013
UGCG	1.0888	1.00E-55	3.56E-54
NOVA1	1.089	1.21E-54	4.18E-53
NTN1	1.0899	0.002945	0.0080604
DRAM1	1.09	4.20E-44	1.10E-42
SLC23A3	1.0901	4.57E-05	0.00016329
NPC1	1.0901	3.74E-47	1.07E-45
BACH1	1.0904	3.99E-51	1.29E-49
BCL6B	1.0917	9.00E-26	1.23E-24
SLC35D2	1.0926	1.75E-47	5.03E-46
PHLDA2	1.0955	9.18E-17	8.03E-16
SATB1	1.0966	4.95E-25	6.50E-24
FAT1	1.099	8.13E-39	1.76E-37
PAM	1.1011	3.59E-58	1.34E-56
ETV7	1.1025	2.27E-15	1.84E-14
EOMES	1.1062	2.82E-05	0.00010319

KLF10	1.1092	3.99E-59	1.52E-57
BRMS1L	1.1095	8.54E-41	2.00E-39
SLC45A4	1.1095	4.40E-46	1.22E-44
DCUN1D4	1.1129	3.69E-69	1.85E-67
CXCR2	1.1179	0.0001962	0.00064764
PLEKHO2	1.1185	3.25E-13	2.31E-12
DCAF4	1.1197	4.50E-22	5.15E-21
RP11-296I10.6	1.1202	3.59E-05	0.0001297
ARNT2	1.122	2.60E-43	6.61E-42
NABP1	1.124	1.86E-18	1.79E-17
ICAM1	1.1319	3.65E-07	1.63E-06
KDELC1	1.1329	9.06E-26	1.23E-24
FUT9	1.1345	0.00023746	0.00077325
UTRN	1.1356	1.80E-22	2.09E-21
RP3-523K23.2	1.1359	4.87E-10	2.76E-09
PANX1	1.1386	1.12E-62	4.66E-61
ARHGAP42	1.1401	4.87E-24	6.12E-23
GPR27	1.1401	1.59E-40	3.69E-39
PAPSS2	1.1425	3.22E-38	6.83E-37
TNC	1.1439	4.58E-74	2.60E-72
ITGB1P1	1.145	0.0011794	0.0034543
CDC34	1.1474	4.09E-76	2.44E-74
SDC2	1.1474	5.66E-130	8.21E-128
RALGPS2	1.1479	1.13E-91	9.08E-90
FGF2	1.1509	1.68E-06	6.99E-06
TNIK	1.151	1.60E-60	6.32E-59
ARSJ	1.1512	1.31E-10	7.68E-10
CENPE	1.1514	9.56E-107	9.81E-105
IL8	1.152	0.0038877	0.010402
SDAD1P1	1.1521	4.15E-08	2.02E-07
RPA4	1.1522	0.0010477	0.0030966
KLHL28	1.1531	1.26E-31	2.15E-30
TMEM117	1.1536	5.77E-39	1.26E-37
DDX26B	1.1587	7.49E-23	8.84E-22
E2F7	1.1622	1.39E-68	6.91E-67
CADPS2	1.1651	9.91E-80	6.33E-78
IHH	1.1687	0.002892	0.0079317
FST	1.1696	1.52E-35	2.97E-34
IGF2BP2	1.1719	1.26E-59	4.86E-58
ZMAT3	1.1722	1.42E-129	2.03E-127
CHD7	1.1751	1.05E-78	6.54E-77
RNF133	1.1827	0.0011621	0.0034061
EOGT	1.1854	7.65E-61	3.07E-59
ACTA1	1.1904	0.0028335	0.0077818
REPS2	1.1914	1.92E-09	1.04E-08

FNDC3B	1.1916	1.36E-84	9.41E-83
IL13RA2	1.1952	1.11E-08	5.68E-08
NSG1	1.1965	6.33E-36	1.25E-34
SLC30A1	1.1974	4.74E-119	5.93E-117
ANKRD19P	1.199	2.50E-05	9.21E-05
MAPK6	1.1992	9.80E-88	7.24E-86
SLC52A1	1.1995	3.32E-34	6.17E-33
PLEKHA1	1.2005	7.83E-82	5.14E-80
KLF2	1.2026	1.94E-19	1.97E-18
MTF2	1.2087	6.05E-77	3.65E-75
TGFB1	1.2107	1.09E-50	3.47E-49
SCN1B	1.2112	5.98E-05	0.00021126
ASXL3	1.2127	4.22E-09	2.24E-08
CHRM3	1.2131	9.61E-20	9.91E-19
PPP2R5A	1.2154	1.72E-101	1.64E-99
MGP	1.2191	0.0025858	0.0071484
TMEM38A	1.2192	1.15E-05	4.42E-05
ELK3	1.2201	1.42E-34	2.69E-33
ACER2	1.2224	5.83E-34	1.07E-32
SHF	1.2243	2.72E-11	1.69E-10
FAM196A	1.2269	1.90E-08	9.48E-08
RP11-448G15.3	1.2358	0.00017095	0.000569
DSCAM	1.2398	5.13E-06	2.04E-05
RP4-631H13.6	1.2419	0.00042306	0.0013319
BEND4	1.2489	2.99E-35	5.75E-34
TRABD2B	1.2535	6.36E-05	0.00022381
PMAIP1	1.2548	2.98E-48	8.75E-47
FAM83B	1.2554	3.58E-26	4.97E-25
JAG1	1.2609	0.0018939	0.0053543
IGF2R	1.2622	8.75E-68	4.22E-66
GRHL3	1.2636	8.58E-12	5.53E-11
ZNF608	1.2664	7.30E-41	1.71E-39
TGIF1	1.2672	1.23E-56	4.41E-55
ADM	1.2682	2.40E-06	9.85E-06
RP11-363E7.4	1.2703	7.03E-34	1.29E-32
ADRB1	1.2704	0.0016837	0.0048014
KCNS2	1.2712	6.10E-06	2.41E-05
TLR2	1.272	0.0017381	0.0049407
UBASH3B	1.2732	1.63E-07	7.52E-07
SEMA3D	1.275	1.05E-109	1.16E-107
NKX1-2	1.2751	0.0017074	0.0048629
A2M	1.2781	4.64E-16	3.92E-15
MT1G	1.2792	2.84E-67	1.34E-65
IGF2BP3	1.2814	1.46E-73	8.09E-72
FAM160B1	1.2817	1.43E-78	8.86E-77

GPR137B	1.2837	5.28E-72	2.86E-70
DUSP5	1.2841	5.26E-15	4.17E-14
IRX5	1.291	3.08E-35	5.92E-34
PELI2	1.2919	2.33E-15	1.89E-14
PDE4A	1.2924	5.78E-60	2.25E-58
AMOTL2	1.2948	2.04E-46	5.73E-45
FRAT2	1.2995	2.62E-65	1.16E-63
C1orf95	1.3033	1.98E-16	1.70E-15
MAP3K3	1.3066	1.50E-40	3.50E-39
PLK2	1.3092	2.59E-65	1.15E-63
CROT	1.3093	7.73E-139	1.31E-136
MEX3B	1.3122	1.31E-21	1.46E-20
STEAP4	1.3138	2.17E-10	1.26E-09
RP13-238F13.5	1.3166	0.00031831	0.0010189
CTC-429C10.2	1.3175	0.00050263	0.0015645
FAT4	1.3205	2.93E-11	1.81E-10
DUSP2	1.3219	1.33E-70	6.84E-69
ZSWIM6	1.3233	1.80E-67	8.55E-66
DLX5	1.3263	0.0010098	0.0029915
HES1	1.3266	1.88E-89	1.45E-87
PHLDA3	1.3268	3.32E-88	2.47E-86
CDH2	1.3273	5.39E-72	2.91E-70
TMEM133	1.3287	4.71E-11	2.87E-10
MAFB	1.3299	4.18E-77	2.54E-75
NAB1	1.3324	2.12E-71	1.13E-69
CACNB4	1.3335	0.00038514	0.0012187
PKD2	1.3403	5.62E-86	4.03E-84
LAMC1	1.3427	2.77E-143	5.05E-141
ANKRD46	1.3456	1.53E-64	6.65E-63
RPL13AP20	1.3493	0.00020607	0.00067817
SMIM13	1.3501	6.39E-92	5.18E-90
KLHL24	1.3581	8.01E-106	8.14E-104
SNORD3B-2	1.3594	0.00056889	0.0017537
CCDC89	1.3634	0.00051173	0.0015905
RAB8B	1.3717	5.28E-93	4.40E-91
RP11-330M19.1	1.373	0.00060249	0.0018482
TSLP	1.3769	3.00E-51	9.69E-50
REEP3	1.3782	1.15E-143	2.12E-141
CTD-3247F14.2	1.3809	0.00063637	0.0019441
NR4A2	1.3823	2.49E-35	4.80E-34
C11orf95	1.4014	5.13E-81	3.32E-79
PKIA	1.4035	6.67E-17	5.89E-16
PRTG	1.4082	1.10E-40	2.56E-39
HOXC8	1.4107	4.33E-06	1.74E-05
CYR61	1.4141	2.02E-35	3.93E-34

PLAG1	1.4282	0.00043426	0.0013644
HTR7	1.4374	1.85E-09	1.00E-08
METRNL	1.4455	2.31E-11	1.43E-10
LFNG	1.4474	9.43E-16	7.84E-15
IER5L	1.4545	2.36E-35	4.56E-34
NEDD9	1.467	2.53E-65	1.13E-63
HRK	1.4689	1.87E-11	1.17E-10
BMP2	1.4721	1.84E-127	2.59E-125
HS3ST5	1.4736	0.00010071	0.00034502
CMTM3	1.4765	7.57E-10	4.23E-09
FOXP2	1.4823	4.15E-79	2.60E-77
LATS2	1.4846	4.09E-107	4.24E-105
RNF145	1.4889	3.18E-52	1.03E-50
FSTL1	1.4955	3.95E-48	1.16E-46
CPEB2	1.5047	1.21E-48	3.59E-47
CCDC68	1.5108	5.44E-06	2.16E-05
ZBTB34	1.5129	7.08E-68	3.44E-66
CDKL5	1.5186	7.52E-10	4.21E-09
JUNB	1.522	5.11E-132	7.91E-130
TLE4	1.5334	6.16E-30	9.87E-29
ZNF532	1.5416	5.27E-125	7.10E-123
GPR137C	1.5467	1.51E-31	2.56E-30
TGFBR1	1.5473	1.53E-93	1.29E-91
ADAMTS7	1.5483	2.82E-28	4.25E-27
ECEL1	1.5538	4.12E-14	3.10E-13
SLCO5A1	1.5649	1.22E-103	1.19E-101
SOX21	1.5872	2.08E-08	1.04E-07
RP11-102F4.3	1.5945	2.27E-60	8.92E-59
KIAA1199	1.6016	5.56E-23	6.61E-22
MB21D2	1.6039	1.14E-05	4.37E-05
FOS	1.6076	1.65E-69	8.34E-68
FNIP2	1.6093	2.92E-153	6.32E-151
LPHN2	1.6112	7.86E-62	3.23E-60
ACVR2B	1.6174	3.13E-31	5.23E-30
LSM11	1.6232	7.98E-104	7.85E-102
ARHGEF3	1.6517	1.48E-33	2.69E-32
PDCD1	1.6523	9.52E-11	5.66E-10
BCL2L11	1.683	2.95E-135	4.71E-133
PCDH19	1.6858	1.10E-18	1.08E-17
LCOR	1.6876	2.59E-174	6.97E-172
PCDH17	1.6879	1.53E-18	1.48E-17
SERPINB5	1.6891	1.13E-48	3.38E-47
AC009133.15	1.6938	1.89E-05	7.09E-05
NR5A2	1.7022	1.18E-15	9.67E-15
DRAXIN	1.7083	3.70E-25	4.87E-24

PLEKHG1	1.7127	8.75E-08	4.13E-07
FOSL1	1.725	3.31E-13	2.35E-12
TNRC6A	1.7291	2.71E-226	1.28E-223
S1PR3	1.7428	8.67E-06	3.37E-05
PPT2	1.7562	2.37E-77	1.45E-75
RP11-216F19.2	1.7604	3.01E-66	1.39E-64
SERPINE1	1.7648	3.35E-07	1.50E-06
NDNF	1.7735	2.39E-16	2.04E-15
SULF2	1.7881	1.47E-42	3.65E-41
VGLL3	1.7891	1.39E-13	1.01E-12
DUSP4	1.7962	7.29E-22	8.24E-21
RRAD	1.8011	3.99E-15	3.19E-14
DOCK10	1.8096	1.62E-32	2.85E-31
ADAMTS5	1.8175	3.26E-07	1.46E-06
POU3F1	1.83	1.42E-07	6.56E-07
TNFRSF11B	1.8707	5.78E-111	6.66E-109
ARC	1.8738	1.04E-28	1.60E-27
VCAN	1.9126	1.49E-169	3.77E-167
SYNPO	1.9136	1.42E-09	7.77E-09
EGR4	1.9194	1.54E-08	7.77E-08
DDX3Y	1.9372	0	0
TNFRSF12A	2.0677	2.10E-118	2.61E-116
CREBRF	2.0764	3.78E-156	8.26E-154
WTIP	2.1296	1.79E-27	2.61E-26
HMGA2	2.1477	5.19E-97	4.74E-95
FOSB	2.1959	6.78E-53	2.24E-51
MMP10	2.3775	1.40E-09	7.66E-09
EGR1	2.6299	0	0
EGR2	2.6557	5.42E-32	9.36E-31
EGR3	4.441	2.49E-229	1.23E-226
